# Synthesis of 2-benzyl *N*-substituted anilines via imine condensation–isoaromatization of (*E*)-2-arylidene-3-cyclohexenones and primary amines

**DOI:** 10.3762/bjoc.20.130

**Published:** 2024-07-02

**Authors:** Lu Li, Na Li, Xiao-Tian Mo, Ming-Wei Yuan, Lin Jiang, Ming-Long Yuan

**Affiliations:** 1 National and Local Joint Engineering Research Center for Green Preparation Technology of Biobased Materials; School of Chemistry and Environment, Yunnan Minzu University, Kunming, China

**Keywords:** aniline, (*E*)-2-arylidene-3-cyclohexenone, imine condensation, isoaromatization, primary amine

## Abstract

A catalyst- and additive-free synthesis of 2-benzyl *N*-substituted anilines from (*E*)-2-arylidene-3-cyclohexenones and primary amines has been reported. The reaction proceeds smoothly through a sequential imine condensation–isoaromatization pathway, affording a series of synthetically useful aniline derivatives in acceptable to high yields. Mild reaction conditions, no requirement of metal catalysts, operational simplicity and the potential for scale-up production are some of the highlighted advantages of this transformation.

## Introduction

Aniline derivatives possessing arylmethyl substituents at the *ortho* position are an important class of amines. They have a wide variety of practical applications, ranging from anti-depression [1], being δ receptor stimulants in analgesic pharmaceuticals [2], to antioxidant additives in the petrochemical industry [3]. Besides, 2-benzylanilines also serve as valuable building blocks in synthetic chemistry [4]. The classical route to this kind of aniline derivatives usually starts from parent anilines, which undergo Friedel–Crafts reaction with acyl halides followed by carbonyl reduction. This electrophilic aromatic substitution usually needs harsh reaction conditions, tedious synthetic procedures and sometimes encounters the trouble of separating positional isomers caused by orientation or steric effects of the pre-existed amino group on the aryl moiety. Nevertheless, anilines are not always readily accessible. Typically, the preparation methods involve S_N_Ar reactions with *N*-centered nucleophiles [5], nitroarene reduction [6] and transition metal (e.g., Pd, Cu)-catalyzed C–N cross coupling of aryl halides, aryl sulfonates or arylboronic acid reagents with ammonia or NH substrates [7–8]. Pre-functionalized arenes are essential precusors in all of these general approaches.

**Scheme 1:**
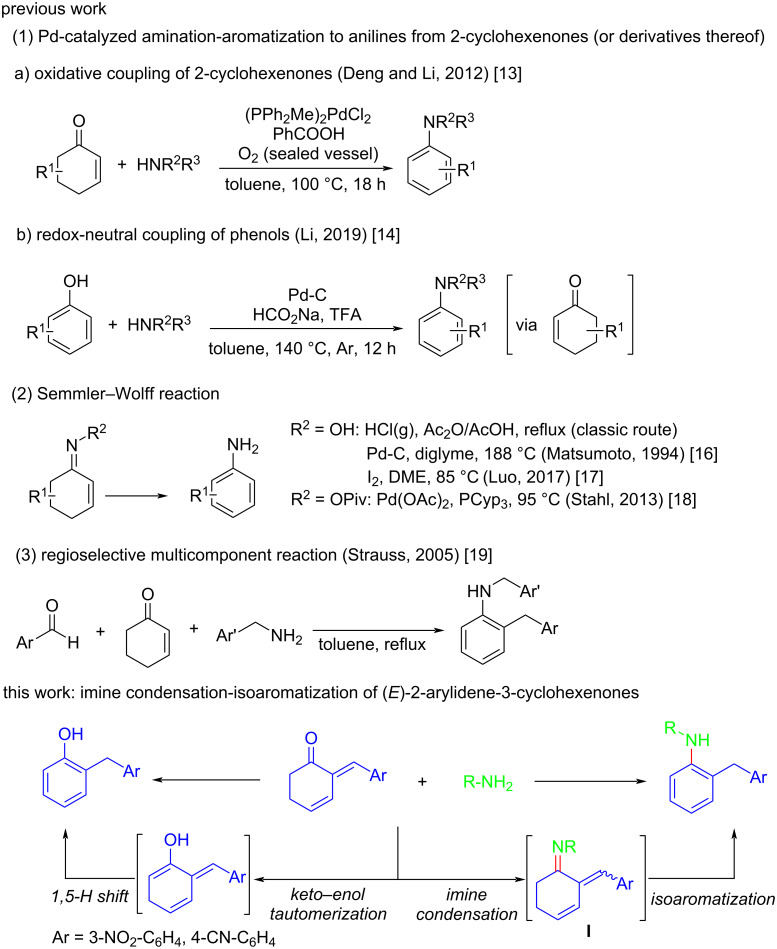
Synthesis of aniline derivatives from 2-cyclohexenones or derivatives thereof.

**Scheme 2:**
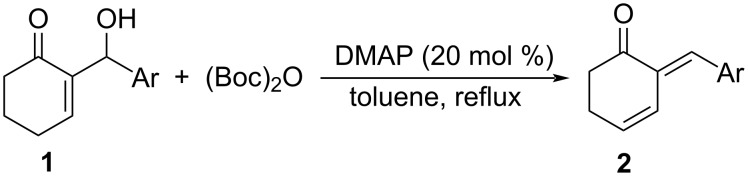
Synthesis of (*E*)-2-arylidene-3-cyclohexenones **2**.

**Scheme 3:**
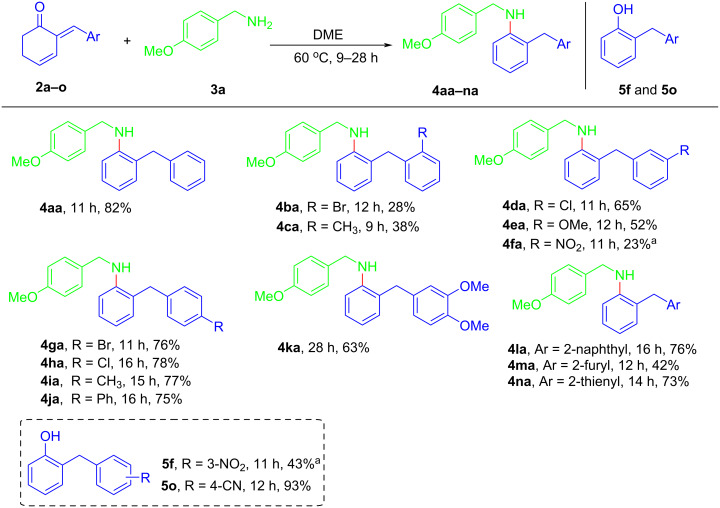
Substrate scope of (*E*)-2-arylidene-3-cyclohexenones **2**. Conditions: reactions were conducted with **2a–o** (0.2 mmol) and **3a** (2.0 mmol) were stirred in DME (2 mL) at 60 °C; Isolated yields; ^a^2-Benzylaniline **4fa** and 2-benzylphenol **5f** were delivered synchronously.

2-Cyclohexenones are fundamental and versatile organic synthetic materials [9–10]. They have been applied as an ideal arylation platform to construct functionalized anilines via an amination–dehydrogenative aromatization strategy with amines as nucleophiles [11–12]. For instance, the groups of Deng and Li reported the Pd catalyzed oxidative coupling of 2-cyclohexenones with amines [13]. Later, the same group demonstrated the direct amination of phenols by reductive coupling of in situ generated 2-cyclohexenones with nucleophilic nitrogen sources like ammonia, amines and hydrazine [14]. The reactions were regarded as via simple nucleophilic addition along with Pd-catalyzed dehydrogenative aromatization in these elegant works (Scheme 1, (1)). The Semmler–Wolff reaction is often implemented in the synthesis of anilines through Brønsted acid or transition-metal-promoted conversion of 2-cyclohexanone oximes [15–18] (Scheme 1, (2)). Moreover, Strauss and co-workers described a green, multicomponent reaction of aromatic aldehydes, 2-cyclohexenone and amines to afford 2-arylmethyl *N*-substituted anilines [19] (Scheme 1, (3)). To date, although plentiful amination–aromatization approaches for the preparation of anilines have been well-established, to develop novel and efficient synthetic methods still remains highly desirable. In continuation of our recent studies on synthetic applications of Morita–Baylis–Hillman (MBH) adducts [20–21], we were interested in further utilizing (*E*)-2-arylidene-3-cyclohexenones that can be facilely synthesized from MBH alcohols to build functionalized molecules. Herein, we wish to report our preliminary study on a catalyst- and additive-free synthesis of 2-benzyl-*N*-substituted anilines. In this work, (*E*)-2-arylidene-3-cyclohexenones firstly react with primary amines to form cyclohexenylimine intermediates **I**. Afterward, isoaromatization resulting from imine–enamine tautomerization and exocyclic double bond shift occurs to give rise to stable aniline products. Interestingly, when the 2-arylidene-3-cyclohexenones bearing a strong electron-withdrawing group take part in the reaction, a base-promoted phenol formation via self-tautomerization of cyclohexenones emerges as a competing reaction pathway (Scheme 1, this work).

## Results and Discussion

The present study began with the preparation of the (*E*)-2-arylidene-3-cyclohexenones **2** via DMAP-catalyzed elimination reaction of 2-cyclohexenone-MBH alcohols **1** and di-*tert*-butyl dicarbonate [22] as depicted in Scheme 2. Starting materials **2** were prepared in moderate to high yields.

Next, we chose (*E*)-2-benzylidenecyclohex-3-en-1-one (**2a**) and 4-methoxybenzylamine (**3a**) as starting materials to investigate the feasibility and efficiency of the reaction (Table 1). Initially, the reaction was conducted in the presence of 2.0 equiv of **3a** in toluene at 60 °C without any addition of catalysts or additives. To our delight, **2a** could be fully converted and the 2-benzylaniline **4aa** was obtained in 52% yield after 46 h (Table 1, entry 1). Some commonly used acid catalysts were tested, however, neither Brønsted acids such as AcOH and TsOH, nor Lewis acids such as FeCl_3_ and BF_3_·Et_2_O, showed a promoting effect on the aimed transformation (Table 1, entries 2–5). Thiourea, which is regarded as a classic H-bond donor in carbonyl activation, also could not boost the yield (Table 1, entry 6). Fortunately, it was found that the yield of **4aa** was gradually increased to 64% upon adding **3a** from 2.0 to 10.0 equiv (Table 1, entries 7 and 8). However, carrying out the reaction under neat conditions rendered to dramatically dropped yield (Table 1, entry 9). It indicates that the involvement of conventional organic solvents into the reaction system seems to be critical for target transformation. Though the exact reason is not apparent now, we speculate that the solvation effect might be beneficial for stabilizing the condensation intermediate **I** and avoiding further unwanted conversions, e.g., nucleophilic attack of excessive benzylamine to the intermediate **I**. The following examination on solvents demonstrated that using ether solvents as the reaction media led to higher yields. More precisely, dimethoxyethane (DME) gave a superior result to THF, affording **4aa** in 82% yield (Table 1, entries 10–16). Subsequently, modification of the reaction temperature or concentration turned out to be unsatisfactory (Table 1, entries 17–19). We also added 4 Å molecular sieves as water scavengers, but it showed no positive influence on the reaction efficiency (Table 1, entry 20). It should be noted that no competing aza-Michael adduct was monitored in all of the evaluated reaction conditions.

**Table 1:** Optimization studies.^a^

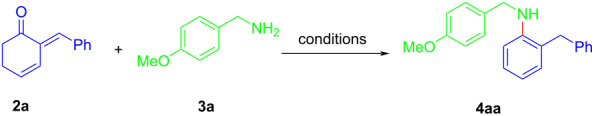					

Entry	**3a** (equiv)	Solvent	Additives (amount)	Time (h)	Yield (%)^b^

1	2.0	toluene	–	46	52
2	2.0	toluene	FeCl_3_ (0.3 equiv)	48	30
3	2.0	toluene	BF_3_·Et_2_O (0.3 equiv)	30	34
4	2.0	toluene	AcOH (0.3 equiv)	38	21
5	2.0	toluene	TsOH (0.3 equiv)	35	20
6	2.0	toluene	thiourea (0.3 equiv)	42	45
7	5.0	toluene	–	33	58
8	10.0	toluene	–	14	64
9	10.0	–	–	12	27
10	10.0	PhCF_3_	–	14	65
11	10.0	CH_3_CN	–	21	57
12	10.0	CHCl_3_	–	18	35
13	10.0	THF	–	25	73
**14**	**10.0**	**DME**	**–**	**11**	**82**
15	10.0	EtOH	–	18	42
16	10.0	DMF	–	18	20
17^c^	10.0	DME	–	16	66
18^d^	10.0	DME	–	11	71
19^e^	10.0	DME	–	13	81
20	10.0	DME	4 Å MS (50 mg)	12	74

^a^Unless otherwise noted, the reactions were performed with **2a** (0.20 mmol) and **3a** (2.0 mmol) in solvent (2 mL) at 60 °C. ^b^Yields of isolated products. ^c^At 50 °C. ^d^1.0 mL of solvent. ^e^4.0 mL of solvent.

According to the above screening results, the generality of the reaction was examined under the optimal reaction conditions as outlined in Table 1, entry 14. Firstly, the substrate range of (*E*)-2-arylidene-3-cyclohexenones **2** were investigated while keeping 4-methoxybenzylamine (**3a**) as the nucleophile (Scheme 3). Generally speaking, the method exhibited good tolerance to various aryl moieties except for those containing strong electron-withdrawing substituent (i.e., -CN, -NO_2_). 3-Cyclohexenones **2** bearing a halogen group (i.e., -Cl, -Br) as well as an electron-donating group (i.e., -Me, -OMe, -Ph) worked well under the optimized reaction conditions, delivering the expected products **4ba**–**ea** and **4ga**–**ka** in 28–78% yields. 3-Cyclohexenones possessing a bulky 2-naphthyl group or heteroaryl group (i.e., 2-furyl, 2-thienyl) also smoothly took part in the reaction to afford **4la**–**na** in 42–76% yields. In the case of 3-cyclohexenone possessing a 3-NO_2_ group, the main reaction pathway appeared to proceed via self-tautomerization, since 2-benzylphenol **5f** was separated in 43% yield along with only 23% yield of normal product **4fa**. When 4*-*CN substituted 3-cyclohexanone was investigated, phenol **5o** was isolated exclusively. This was probably due to the significantly enhanced acidity of 3-cyclohexanones caused by the strong electron-withdrawing effect, which made **3a** as a base rather than a nucleophile under such conditions. The location of substituents was found to affect the product yield greatly, considering that the reactions worked better with 3-cyclohexenones bearing a *para*-substituent as compared to their *ortho*- or *meta*-substituted counterparts. It might be explained by the less obvious steric resistance of the former in the process of Schiff base formation. In addition, an alkylidenyl-equipped 3-cyclohexanone was found to be incompatible with the current reaction system, only generating a mixture of unidentifiable byproducts.

**Scheme 4:**
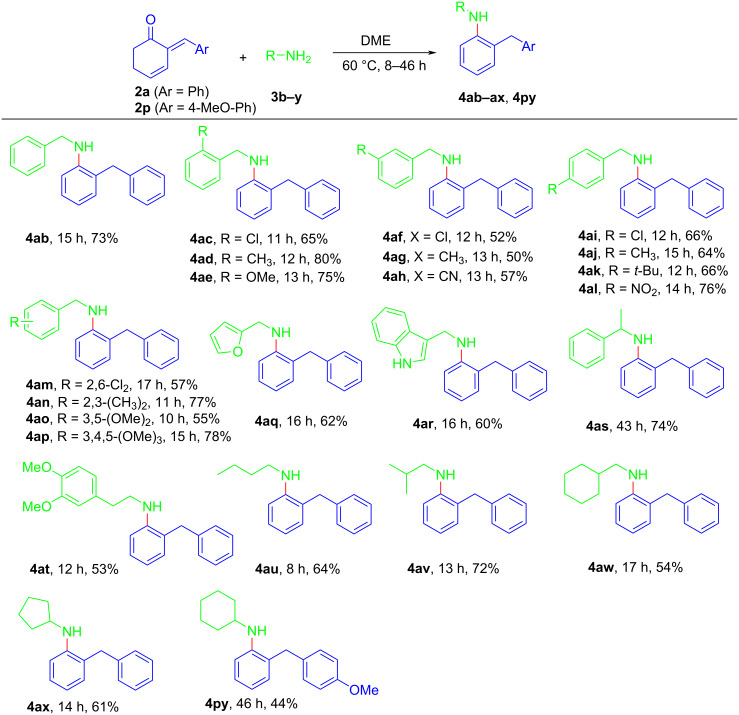
Substrate scope of primary amines **3**. Conditions: reactions conducted with **2** (0.2 mmol) and **3b**–**y** (2.0 mmol) were stirred in DME (2 mL) at 60 °C; isolated yields.

Next, we explored the scope of various primary amines under the optimal conditions (Scheme 4). With 3-cyclohexenones **2a** and **2p** used, a variety of primary amines **3** successfully participated in the reaction to produce *N*-substituted anilines **4** in moderate to good yields. The electronic properties of the substituents, irrespective of their positions on benzylamines, displayed no substantial disparity on the reaction outcomes, leading to the formation of **4ab**–**ap** in 50–80% yields. Both the heteroarylmethylamines and sterically hindered α-methylbenzylamine reacted nicely to afford **4aq**–**as** in 60–74% yields. This method was equally valid for β-phenylethylamine to provide **4at** in 53% yield. Notably, not only linear but also cyclic primary amines were applicable for the established transformation, and targeted products **4au**–**ax** and **4py** were synthesized in 44–72% yields. Finally, when we switched our attention to the generality of primary aromatic amines such as aniline or secondary amines such as dibenzylamine, it was found that only the starting materials were recovered after work-up of the reaction mixture.

The structure of 2-benzyl *N*-substituted anilines **4** were determined by detailed analysis of their NMR spectral data. In particular, ^1^H NMR spectrum of the representative compound **4aa** shows two characteristic signals at δ = 4.16 (singlet) and 3.89 (singlet) that correspond to the two groups of benzylic protons, respectively. Two peaks at δ = 3.77 (singlet) and 3.81 (broad singlet) are attributed to the -OMe group and active hydrogen of -NH group, respectively. The aromatic protons, shown as multi- or doublet signals at δ = 7.21, 7.07, 6.71 and 6.63 indicate the newly formed aromatic protons derived from the isoaromatization of the fragment of 3-cyclohexenone. This is further supported by the ^13^C NMR spectrum, which contains two peaks at δ = 38.4 and 47.7 indicating the two types of benzylic carbons. The NMR data of known compound **4ab** were also in good correlation with previously reported data [19].

**Scheme 5:**
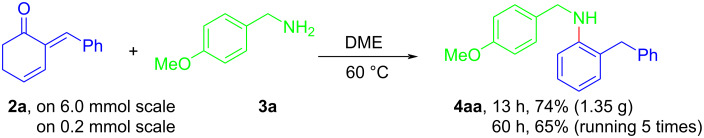
Gram-scale reaction and successive one-pot synthesis.

The synthetic practicability of the protocol was further demonstrated. As depicted in Scheme 5, we first attempted scale-up synthesis of product **4aa**. Pleasingly, when starting from **2a** on a 6.0 mmol scale, the product **4aa** was afforded in 74% yield. We also conducted the successive synthesis of **4aa** in a manner of one-pot procedure. On the condition of full conversion of **2a** under the standard reactions, 1.0 equiv of each substrate was added synchronously. After running 5 times, 65% yield was obtained within a total reaction time of 60 h.

**Scheme 6:**
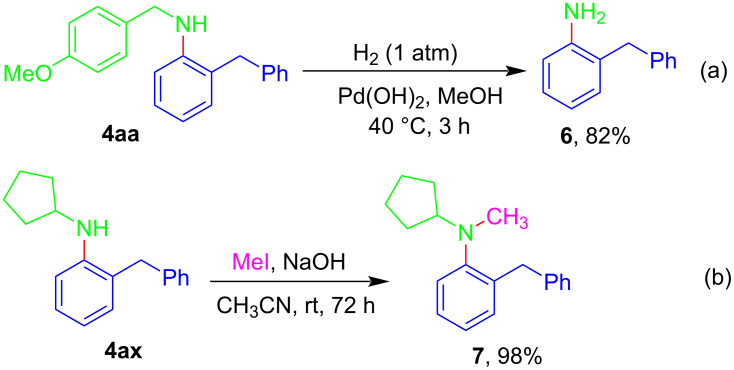
Synthetic manipulation.

Finally, we explored the synthetic versatility of the products in this methodology. Debenzylation of product **4aa** could be easily carried out by catalytic hydrogenation to produce **6** (Scheme 6a). On the other hand, **4ax** could smoothly undergo *N*-methylation with MeI to give product **7** in quantitative yield (Scheme 6b).

## Conclusion

In conclusion, we have developed an efficient method to rapidly synthesize 2-benzyl-*N*-substituted anilines from (*E*)-2-arylidene-3-cyclohexenones and primary aliphatic amines. The reaction proceeds through an imine condensation–isoaromatization approach under catalyst- and additive-free conditions, allowing the generation of synthetically useful aniline derivatives in 23–82% yields. This method provides an alternative to the construction of anilines via an amination–aromatization strategy. Furthermore, it is also characterized by simple operation, mild reaction conditions, broad substrate scope and efficiency on a gram-scale preparation, thus allowing a new and convenient process to assemble synthetically valuable industrial or fine chemicals. Further exploration into more synthetic application of (*E*)-2-arylidene-3-cyclohexenones is in progress.

## Experimental

### General procedure for the preparation of 2-benzyl-*N*-substituted anilines **4** and 2-benzylphenols **5f**/**5o**

In a vial containing a magnetic stirrer was placed (*E*)-2-arylidene-3-cyclohexenone **2** (0.2 mmol), primary aliphatic amine **3** (2.0 mmol) and DME (2 mL). The reaction mixture was stirred at 60 °C and the reaction process was monitored by TLC analysis. After completion, the solvent was concentrated under reduced pressure and the residue was purified by column chromatography on silica gel to give product **4**. In the case of reacting with 3-NO_2_ bearing **2f**, 2-benzylphenol **5f** was partially obtained together with normal product **4fa**. 4-CN substituted **2o** generated 2-benzylphenol **5o** exclusively.

## Supporting Information

File 1 Experimental procedures, characterization data, and copies of NMR spectra of all new compounds.

## Data Availability

All data that supports the findings of this study is available in the published article and/or the supporting information to this article.
