# Supplementary material for: Synthesis of 2-benzyl N-substituted anilines via imine condensation–isoaromatization of (E)-2-arylidene-3-cyclohexenones and primary amines
Source: Beilstein J Org Chem. 2024 Jul 2;20:1468–75. doi: 10.3762/bjoc.20.130 (PMC11228828; doi:10.3762/bjoc.20.130)
Supplement: File 1 — Experimental procedures, characterization data, and copies of NMR spectra of all new compounds. [file Beilstein_J_Org_Chem-20-1468-s001.pdf]

BEILSTEIN JOURNAL OF ORGANIC CHEMISTRY

Supporting Information

for

Synthesis of 2-benzyl *N*-substituted anilines via imine condensation–isoaromatization of (*E*)-2-arylidene-3-cyclohexenones and primary amines

Lu Li, Na Li, Xiao-Tian Mo, Ming-Wei Yuan, Lin Jiang and Ming-Long Yuan

Beilstein J. Org. Chem. **2024,** *20,* 1468–1475. doi:10.3762/bjoc.20.130

Experimental procedures, characterization data, and copies of NMR spectra of all new compounds

License and Terms: This is a supporting information file under the terms of the Creative Commons Attribution License (https://creativecommons.org/licenses/by/4.0). Please note that the reuse, redistribution and reproduction in particular requires that the author(s) and source are credited and that individual graphics may be subject to special legal provisions.
The license is subject to the *Beilstein Journal of Organic Chemistry* terms and conditions: (https://www.beilstein-journals.org/bjoc/terms)

Table of Contents

I. General information S1

II. Synthesis and characterization data of (*E*)-2-methylene-3-cyclohexenones **2** S1–S2

III. Synthesis and characterization data of product **4** and **5f**/**5o**. .... S2−S13

IV. Gram-scale synthesis of **4aa** S13

V. Successive one-pot synthesis of **4aa** S13

VI. Derivation of the products .... S13−S14

VII. References S14

VIII. Copies of ^{1}H and ^{13}C NMR spectra of new compounds .... S15−S61

I. General information

Melting points were measured using a micro-melting point tester (Model FX-RD-X5E). ^{1}H NMR and ^{13}C NMR spectra were recorded on a Bruker Advance III 400 MHz spectrometers with tetramethylsilane (TMS) as an internal standard and $CDCl_3$ or DMSO-d_6 as a solvent. The chemical shifts (δ) were expressed in parts per million (ppm) and the coupling constants (*J*) were in Hz. Reactions were detected by thin-layer chromatography (TLC) with pre-coated G254 silica gel. All solvents and chemical reagents unless otherwise noted were used as commercially available without further purification. Starting materials of (*E*)-2-methylene-3-cyclohexenones **2a**–**p** were synthesized according to the literature procedures[1].

II. Synthesis and characterization data of (*E*)-2-methylene-3-cyclohexenones 2

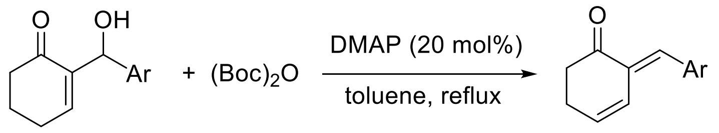

1 **2**

According to the reported literature[1], to a solution of MBH alcohol **1** (13.2 mmol) in toluene (50 mL) was added $(Boc)_2O$ (3.16 g, 14.5 mmol) and DMAP (0.32 g, 2.6 mmol), and the resulting solution was stirred at reflux temperature for 12 h. After completion of the reaction monitored by TLC analysis, the solvent was concentrated under reduced pressure and the residue was purified by column chromatography on silica gel (eluent: petroleum ether/ethyl acetate = 20:1) to afford the pure (*E*)-2-methylene-3-cyclohexenones **2**. (*E*)-2-methylene-3-cyclohexenones **2** bearing a C_6H_5 (**2a**)[1], 2-BrC_6H_4 (**2b**)[2], 2-MeC_6H_4 (**2c**)[2], 3-ClC_6H_4 (**2d**)[2], 3-MeOC_6H_4 (**2e**)[1], 4-BrC_6H_4 (**2g**)[2], 4-ClC_6H_4 (**2h**)[1], 4-MeC_6H_4 (**2i**)[1], 2-furyl (**2m**)[1], 2-thienyl (**2n**)[2], 4-MeOC_6H_4 (**2p**)[1] are known compounds.

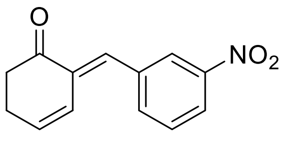

2-(3-Nitrobenzylidene)cyclohex-3-en-1-one (2f)

Yellow solid; 44% yield; m. p. 52-56 °C; ^{1}H NMR (400 MHz, $CDCl_3$) δ 8.19 (s, 1H), 8.09 (d, *J* = 8.0 Hz, 1H), 7.64 (d, *J* = 8.0 Hz, 1H), 7.52-7.48 (m, 1H), 7.30 (s, 1H), 6.77 (d, *J* = 12.0 Hz, 1H), 6.24-6.20 (m, 1H), 2.65-2.62 (m, 2H), 2.59-2.56 (m, 2H); ^{13}C NMR (100 MHz, $CDCl_3$) δ 199.6, 148.5, 137.3, 135.8, 133.7, 133.1, 129.6, 128.7, 124.3, 124.2, 123.1, 38.2, 24.8. IR (KBr): υ 1589, 1535, 1354, 1259, 732 cm^{-1}. ESI HRMS: calcd. for $C_{13}H_{11}NO_3$+Na $[M+Na]^+$ 252.0637, found 252.0638.

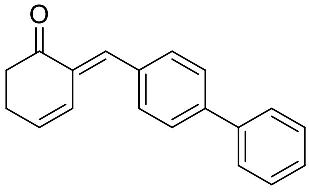

2-([1,1'-Biphenyl]-4-ylmethylene)cyclohex-3-en-1-one (2j)

Yellow solid; 47% yield; m. p. 107-109 ºC; ^{1}H NMR (400 MHz, $CDCl_3$) δ 7.63-7.60 (m, 4H), 7.51 (d, *J* = 8.0 Hz, 2H), 7.46-7.43 (m, 3H), 7.38-7.34 (m, 1H), 6.99 (d, *J* = 8.0 Hz, 1H), 6.20-6.15 (m, 1H), 2.70-2.67 (m, 2H), 2.62-2.59 (m, 2H); ^{13}C NMR (100 MHz, $CDCl_3$) δ 200.3, 141.5, 140.4, 134.6, 131.7, 131.2, 131.2, 130.7, 128.9, 127.8, 127.2, 127.1, 125.4, 38.4, 24.7. IR (KBr): υ 1599, 1484, 1353, 1270, 1004 cm^{-1}. ESI HRMS: calcd. for $C_{19}H_{16}O$+Na $[M+Na]^+$ 283.1099, found 283.1096.

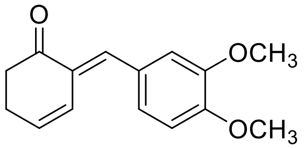

2-(3,4-Dimethoxybenzylidene)cyclohex-3-en-1-one (2k)

Yellow solid; 62% yield; m. p. 70-72 ºC; ^{1}H NMR (400 MHz, $CDCl_3$) δ 7.39 (s, 1H), 7.08 (d, *J* = 8.0 Hz, 1H), 6.98-6.95 (m, 2H), 6.89 (d, *J* = 8.0 Hz, 1H), 6.17-6.12 (m, 1H), 3.92 (s, 3H), 3.90 (s, 3H), 2.66-2.65 (m, 2H), 2.61-2.58 (m, 2H); ^{13}C NMR (100 MHz, $CDCl_3$) δ 200.4, 149.8, 148.9, 132.3, 130.5, 129.9, 128.4, 125.5, 123.8, 113.3, 111.2, 56.1, 38.3, 24.6. IR (KBr): υ 1680, 1570, 1515, 1255, 1133, 1025 cm^{-1}. ESI HRMS: calcd. for $C_{15}H_{16}O_3$+H $[M+H]^+$ 245.1178, found 245.1180.

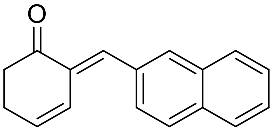

2-(Naphthalen-2-ylmethylene)cyclohex-3-en-1-one (2l)

Yellow solid; 57% yield; m. p. 81-83 ºC; ^{1}H NMR (400 MHz, $CDCl_3$) δ 7.91 (s, 1H), 7.85-7.83 (m, 3H), 7.59 (s, 1H), 7.55-7.53 (m, 1H), 7.52-7.49 (m, 2H), 7.04 (d, *J* = 8.0 Hz, 1H), 6.23-6.18 (m, 1H), 2.73-2.70 (m, 2H), 2.65-2.62 (m, 2H); ^{13}C NMR (100 MHz, $CDCl_3$) δ 200.4, 133.3, 133.3, 133.1, 132.3, 131.5, 131.4, 130.0, 128.5, 128.2, 127.8, 127.4, 126.6, 125.5, 38.4, 24.8. IR (KBr): υ 1684, 1560, 1442, 1229, 1123 cm^{-1}. ESI HRMS: calcd. for $C_{17}H_{14}O$+H $[M+H]^+$ 235.1123, found 235.1125.

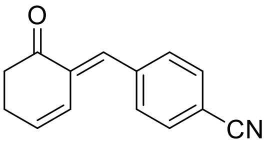

4-((6-Oxocyclohex-2-en-1-ylidene)methyl)benzonitrile (2o)

Pale yellow solid; 42% yield; m. p. 98-100 ºC; ^{1}H NMR (400 MHz, $CDCl_3$) δ 7.57 (d, *J* = 8.0 Hz, 2H), 7.41 (d, *J* = 8.0 Hz, 2H), 7.23 (s, 1H), 6.73 (d, *J* = 8.0 Hz, 1H), 6.19-6.16 (m, 1H), 2.59-2.58 (m, 2H), 2.55-2.52 (m, 2H); ^{13}C NMR (100 MHz, $CDCl_3$) δ 199.3, 140.0, 133.5, 132.9, 132.0, 128.9, 124.2, 118.4, 111.5, 37.9, 24.6. IR (KBr): υ 2231, 1687, 1583, 1266, 1137, 875 cm^{-1}. ESI HRMS: calcd. for $C_{14}H_{11}NO$+H $[M+H]^+$ 210.0919, found 210.0916.

III. Synthesis and characterization data of products 4 and 5f/5o

In a vial containing a magnetic stirrer was placed (*E*)-2-methylene-3-cyclohexenone **2** (0.2 mmol), primary aliphatic amine **3** (2.0 mmol) and DME (2 mL). The reaction mixture was stirred at 60 ºC and the reaction process was monitored by TLC analysis. After completion, the solvent was concentrated under reduced

pressure and the residue was purified by column chromatography on silica gel to give product **4**. In the case of reaction with a *meta*-nitro bearing (*E*)-2-methylene-3-cyclohexenone **2f**, 2-benzylphenol **5f** was partially obtained together with normal product **4fa**. A *para*-cyano substituted 3-cyclohexenone **2o** generated 2-benzylphenol **5o** exclusively.

Product **4ab** is a known compound, and the spectroscopic data were consistent with that reported in cited reference[3].

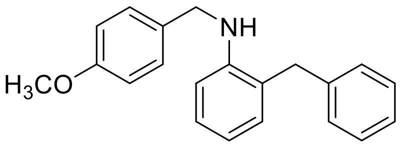

2-Benzyl-*N*-(4-methoxybenzyl)aniline (4aa)

White solid; 82% yield; m. p. 102-104 ºC; ^{1}H NMR (400 MHz, $CDCl_3$) δ 7.29-7.25 (m, 2H), 7.22 (d, *J* = 8.0 Hz, 1H), 7.16-7.13 (m, 3H), 7.07 (d, *J* = 4.0 Hz, 1H), 7.00 (d, *J* = 8.0 Hz, 2H), 6.78 (d, *J* = 8.0 Hz, 2H), 6.73-6.70 (m, 1H), 6.63 (d, *J* = 8.0 Hz, 1H), 4.16 (s, 2H), 3.89 (s, 2H), 3.81 (br.s, 1H), 3.77 (s, 3H); ^{13}C NMR (100 MHz, $CDCl_3$) δ 158.8, 146.2, 139.5, 131.4, 130.7, 128.8, 128.7, 128.6, 128.0, 126.5, 124.8, 117.3, 114.0, 111.0, 55.4, 47.7, 38.4. IR (KBr): υ 1599, 1364, 1239, 1068 cm^{-1}. ESI HRMS: calcd. for $C_{21}H_{21}NO$+Na [M+Na]$^+$ 326.1521, found 326.1523.

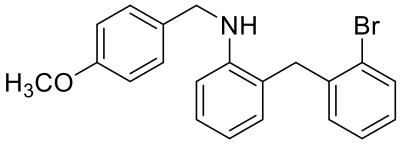

2-(2-Bromobenzyl)-*N*-(4-methoxybenzyl)aniline (4ba)

Pale yellow solid; 28% yield; m. p. 99-103 ºC; ^{1}H NMR (400 MHz, $CDCl_3$) δ 7.50 (dd, *J* = 8.0 Hz, 4.0 Hz, 1H), 7.15-7.09 (m, 2H), 7.07-7.01 (m, 3H), 6.94 (d, *J* = 8.0 Hz, 1H), 6.90 (d, *J* = 8.0 Hz, 1H), 6.74 (d, *J* = 8.0 Hz, 2H), 6.65-6.61 (m, 1H), 6.59 (d, *J* = 8.0 Hz, 1H), 4.17 (s, 2H), 3.88 (s, 2H), 3.71 (s, 3H); ^{13}C NMR (100 MHz, $CDCl_3$) δ 158.9, 146.0, 138.6, 132.9, 131.4, 130.6, 130.4, 128.6, 128.2, 128.1, 127.7, 125.2, 123.4, 117.4, 111.0, 55.4, 47.6, 38.0. IR (KBr): υ 1604, 1513, 1360, 1247, 1174 cm^{-1}. ESI HRMS: calcd. for $C_{21}H_{20}BrNO$+H [M+H]$^+$ 382.0807, found 382.0805.

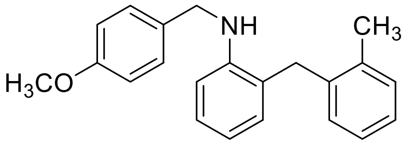

***N*-(4-Methoxybenzyl)-2-(2-methylbenzyl)aniline (4ca)**

Pale yellow solid; 38% yield; m. p. 118-122 ºC; ^{1}H NMR (400 MHz, $CDCl_3$) δ 7.17-7.09 (m, 6H), 6.98 (d, *J* = 4.0 Hz, 1H), 6.86 (d, *J* = 8.0 Hz, 1H), 6.82 (d, *J* = 12.0 Hz, 2H), 6.70-6.66 (m, 2H), 4.23 (s, 2H), 3.79 (s, 2H), 3.78 (s, 3H), 2.26 (s, 3H); ^{13}C NMR (100 MHz, $CDCl_3$) δ 158.9, 146.1, 137.2, 136.9, 131.5, 130.4, 130.0, 129.0, 128.7, 127.7, 126.7, 126.3, 124.2, 117.5, 114.1, 110.8, 55.4, 47.8, 35.3, 19.7. IR (KBr): υ 1601, 1510, 1354, 1242, 1173 cm^{-1}. ESI HRMS: calcd. for $C_{22}H_{23}NO$+Na [M+Na]$^+$ 340.1677, found 340.1674.

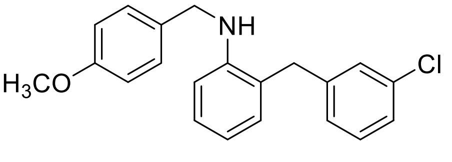

2-(3-Chlorobenzyl)-*N*-(4-methoxybenzyl)aniline (4da)

Yellow oil; 65% yield; ^{1}H NMR (400 MHz, $CDCl_3$) δ 7.12-7.06 (m, 4H),

6.96-6.94 (m, 4H), 6.73 (d, *J* = 8.0 Hz, 2H), 6.66-6.63 (m, 1H), 6.56 (d, *J* = 8.0 Hz, 1H), 4.10 (s, 2H), 3.77 (s, 2H), 3.70 (s, 3H), 3.64 (br.s, 1H); ^{13}C NMR (100 MHz, $CDCl_3$) δ 158.9, 146.0, 141.7, 134.7, 131.3, 130.8, 130.0, 128.8, 128.6, 128.3, 126.9, 126.8, 123.8, 117.4, 114.1, 111.2, 55.4, 47.7, 38.0. IR (neat): υ 1596, 1513, 1364, 1249, 1175 cm^{-1}. ESI HRMS: calcd. for $C_{21}H_{20}ClNO$+Na [M+Na]$^+$ 360.1131, found 360.1134.

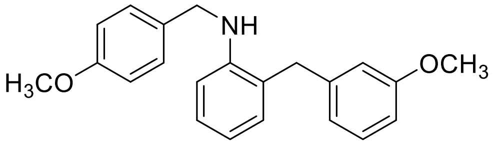

2-(3-Methoxybenzyl)-*N*-(4-methoxybenzyl)aniline (4ea)

Pale yellow oil; 52% yield; ^{1}H NMR (400 MHz, $CDCl_3$) δ 7.13-7.04 (m, 2H), 6.99 (d, *J* = 8.0 Hz, 1H), 6.94 (d, *J* = 8.0 Hz, 2H), 6.72-6.62 (m, 6H), 6.54 (d, *J* = 8.0 Hz, 1H), 4.09 (s, 2H), 3.78 (s, 3H), 3.69 (s, 3H), 3.63 (s, 3H); ^{13}C NMR (100 MHz, $CDCl_3$) δ 160.0, 158.8, 146.2, 141.2, 131.4, 130.7, 129.8, 128.6, 128.0, 124.6, 121.1, 117.3, 114.3, 112.0, 111.0, 55.4, 55.2, 47.6, 38.5. IR (neat): υ 1599, 1355, 1261, 1090, 799 cm^{-1}. ESI HRMS: calcd. for $C_{22}H_{23}NO_2$+H [M+H]$^+$ 334.1807, found 334.1806.

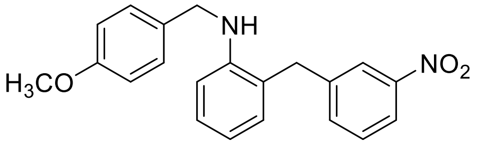

***N*-(4-Methoxybenzyl)-2-(3-nitrobenzyl)aniline (4fa)**

Yellow solid; 23% yield; m. p. 96-98 °C; ^{1}H NMR (400 MHz, $CDCl_3$) δ 8.00 (d, *J* = 8.0 Hz, 1H), 7.95 (s, 1H), 7.41-7.34 (m, 2H), 7.14-7.11 (m, 1H), 7.00-6.96 (m, 3H), 6.73 (d, *J* = 8.0 Hz, 2H), 6.69-6.66 (m, 1H), 6.62 (d, *J* = 8.0 Hz, 1H), 4.12 (s, 2H), 3.89 (s, 2H), 3.71 (s, 3H); ^{13}C NMR (100 MHz, $CDCl_3$) δ 159.0, 148.7, 145.9, 141.8, 134.8, 131.1, 130.7, 129.6, 128.7, 128.7, 123.6, 123.1, 121.7, 117.7, 114.2, 111.4, 55.4, 47.8, 37.8. IR (KBr): υ 1602, 1527, 1353, 1246 cm^{-1}. ESI HRMS: calcd. for $C_{21}H_{20}N_2O_3$ +Na [M+Na]$^+$ 371.1372, found 371.1376.

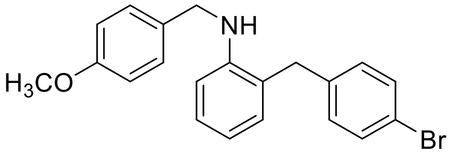

2-(4-Bromobenzyl)-*N*-(4-methoxybenzyl)aniline (4ga)

Yellow oil; 76% yield; ^{1}H NMR (400 MHz, $CDCl_3$) δ 7.28 (d, *J* = 8.0 Hz, 2H), 7.09-7.05 (m, 1H), 6.93-6.90 (m, 5H), 6.72 (d, *J* = 12.0 Hz, 2H), 6.64-6.61 (m, 1H), 6.55 (d, *J* = 8.0 Hz, 1H), 4.06 (s, 2H), 3.71 (s, 2H), 3.68 (s, 3H), 3.60 (br.s, 1H); ^{13}C NMR (100 MHz, $CDCl_3$) δ 158.9, 146.0, 138.6, 131.8, 131.2, 130.7, 130.4, 128.7, 128.2, 124.0, 120.3, 117.4, 114.1, 111.1, 55.4, 47.7, 37.7. IR (neat): υ 1603, 1513, 1362, 1248, 748 cm^{-1}. ESI HRMS: calcd. for $C_{21}H_{20}BrNO$ +H [M+H]$^+$ 382.0807, found 382.0805.

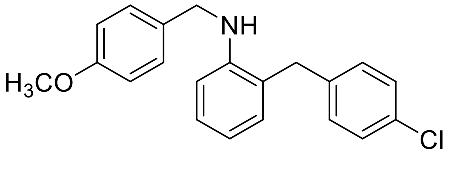

2-(4-Chlorobenzyl)-*N*-(4-methoxybenzyl)aniline (4ha)

Yellow solid; 78% yield, m. p. 46-49 °C; ^{1}H NMR (400 MHz, $CDCl_3$) δ 7.14 (d, *J* = 8.0 Hz, 2H), 7.09-7.05 (m, 1H), 6.99-6.92 (m, 5H), 6.72 (d,

J = 8.0 Hz, 2H), 6.65-6.61 (m, 1H), 6.56 (d, J = 8.0 Hz, 1H), 4.07 (s, 2H), 3.74 (s, 2H), 3.69 (s, 3H), 3.61 (br.s, 1H); ^{13}C NMR (100 MHz, $CDCl_3$) δ 158.9, 146.0, 138.0, 132.3, 131.3, 130.7, 130.0, 128.9, 128.7, 128.2, 124.1, 117.4, 114.1, 111.1, 55.4, 47.7, 37.6. IR (neat): υ 1602, 1513, 1364, 1248, 747 cm^{-1}. ESI HRMS: calcd. for $C_{21}H_{20}ClNO$ +Na $[M+Na]^+$ 360.1131, found 360.1133.

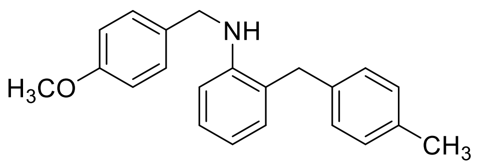

_N_-(4-Methoxybenzyl)-2-(4-methylbenzyl)aniline (4ia)

Pale yellow oil; 77% yield; ^{1}H NMR (400 MHz, $CDCl_3$) δ 7.15-7.11 (m, 1H), 7.08-7.02 (m, 5H), 7.00 (d, J = 8.0 Hz, 2H), 6.78 (d, J = 8.0 Hz, 2H), 6.72-6.68 (m, 1H), 6.61 (d, J = 8.0 Hz, 1H), 4.16 (s, 2H), 3.83 (s, 3H), 3.76 (s, 3H), 2.32 (s, 3H); ^{13}C NMR (100 MHz, $CDCl_3$) δ 158.8, 146.2, 136.3 136.0, 131.5, 130.6, 129.5, 128.6, 127.9, 125.0, 117.3, 114.0, 111.0, 55.4, 47.6, 38.0, 21.2. IR (neat): υ 1603, 1514, 1360, 1248, 1175, 748 cm^{-1}. ESI HRMS: calcd. for $C_{22}H_{23}NO$ +H $[M+H]^+$ 318.1858, found 318.1856.

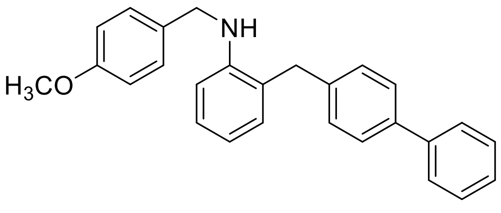

2-([1,1'-Biphenyl]-4-ylmethyl)-_N_-(4-methoxybenzyl)aniline (4ja)

Yellow solid; 75% yield; m. p. 75-77 °C; ^{1}H NMR (400 MHz, $CDCl_3$) δ 7.48 (d, J = 8.0 Hz, 2H), 7.41 (d, J = 8.0 Hz, 2H), 7.35-7.31 (m, 2H), 7.25-7.21 (m, 1H), 7.13 (d, J = 8.0 Hz, 2H), 7.10-7.06 (m, 1H), 7.02 (d, J = 8.0 Hz, 1H), 6.91 (d, J = 8.0 Hz, 2H), 6.67-6.63 (m, 3H), 6.56 (d, J = 8.0 Hz, 1H), 4.08 (s, 2H), 3.83 (s, 2H), 3.74 (br.s, 1H), 3.63 (s, 3H); ^{13}C NMR (100 MHz, $CDCl_3$) δ 158.8, 146.2, 141.0, 139.4, 138.6, 131.3, 130.7, 129.1, 128.9, 128.6, 128.0, 127.5, 127.3, 127.1, 124.6, 117.3, 114.0, 111.0, 55.3, 47.7, 38.0. IR (KBr): υ 1599, 1511, 1365, 1250, 747 cm^{-1}. ESI HRMS: calcd. for $C_{27}H_{25}NO$ +Na $[M+Na]^+$ 402.1834, found 402.1838.

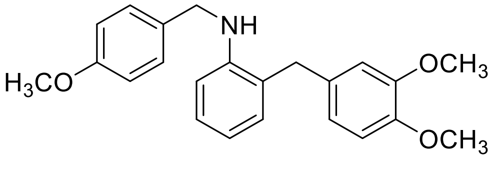

2-(3,4-Dimethoxybenzyl)-_N_-(4-methoxybenzyl)aniline (4ka)

Pale yellow solid; 63% yield; m. p. 102-105 °C; ^{1}H NMR (400 MHz, $CDCl_3$) δ 7.20-7.16 (m, 1H), 7.10-7.06 (m, 3H), 6.83 (d, J = 8.0 Hz, 2H), 6.79 (d, J = 8.0 Hz, 1H), 6.76 (d, J = 8.0 Hz, 1H), 6.73-6.70 (m, 2H), 6.67 (d, J = 8.0 Hz, 1H), 4.19 (s, 2H), 3.88 (s, 3H), 3.85 (s, 2H), 3.80 (s, 3H), 3.76 (s, 3H); ^{13}C NMR (100 MHz, $CDCl_3$) δ 158.8, 149.2, 147.7, 146.2, 131.9, 131.4, 130.4, 128.7, 127.9, 125.0, 120.6, 117.3, 114.0, 111.9, 111.5, 110.9, 56.0, 55.8, 55.3, 47.7, 38.0. IR (KBr): υ 1598, 1365, 1136, 777 cm^{-1}. ESI HRMS: calcd. for $C_{23}H_{25}NO_3$ +Na $[M+Na]^+$ 386.1732, found 386.1736.

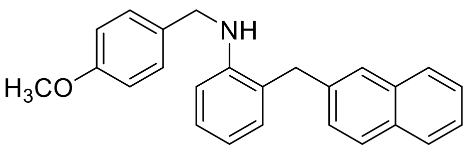

N-(4-Methoxybenzyl)-2-(naphthalen-2-ylmethyl)aniline (4la)

Pale yellow solid; 76% yield; m. p. 89-91 °C; ^{1}H NMR (400 MHz, $CDCl_3$) δ 7.73-7.70 (m, 1H), 7.66 (d, J = 8.0 Hz, 1H), 7.61-7.59 (m, 1H), 7.47 (s, 1H), 7.36-7.34 (m, 2H), 7.21 (d, J = 8.0 Hz, 1H), 7.11-7.07 (m, 1H), 7.04 (d, J = 8.0 Hz, 1H), 6.79 (d, J = 8.0 Hz, 2H), 6.68-6.64 (m, 1H), 6.56-6.52 (m, 3H), 4.02 (s, 2H), 3.95 (s, 2H), 3.79 (br.s, 1H), 3.63 (s, 3H); ^{13}C NMR (100 MHz, $CDCl_3$) δ 158.7, 146.3, 137.0, 133.8, 132.4, 131.3, 128.5, 128.1, 127.8, 127.7, 127.2, 127.0, 126.2, 125.6, 124.5, 117.3, 113.9, 111.0, 55.3, 47.6, 38.7. IR (KBr): υ 1599, 1511, 1356, 1247 cm^{-1}. ESI HRMS: calcd. for $C_{25}H_{23}NO$ +H $[M+H]^+$ 354.1858, found 354.1857.

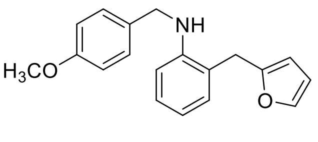

2-(Furan-2-ylmethyl)-N-(4-methoxybenzyl)aniline (4ma)

Yellow solid; 42% yield; m. p. 98-100 °C; ^{1}H NMR (400 MHz, $CDCl_3$) δ 7.22 (s, 1H), 7.11 (d, J = 8.0 Hz, 2H), 7.08-7.04 (m, 1H), 7.00 (d, J = 8.0 Hz, 1H), 6.77 (d, J = 8.0 Hz, 2H), 6.65-6.61 (m, 1H), 6.57 (d, J = 8.0 Hz, 1H), 6.20 (d, J = 4.0 Hz, 1H), 5.91 (d, J = 4.0 Hz, 1H), 4.16 (s, 2H), 3.79 (s, 2H), 3.71 (s, 3H); ^{13}C NMR (100 MHz, $CDCl_3$) δ 158.9, 153.4, 146.2, 141.7, 131.4, 130.3, 128.8, 128.3, 122.5, 117.5, 114.1, 111.2, 110.5, 106.5, 55.4, 47.7, 31.2. IR (KBr): υ 1600, 1513, 1354, 1244 cm^{-1}. ESI HRMS: calcd. for $C_{19}H_{19}NO_2$ +H $[M+H]^+$ 294.1494, found 294.1496.

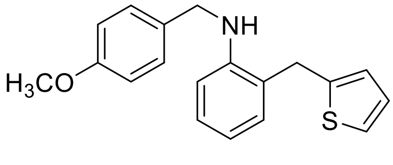

N-(4-Methoxybenzyl)-2-(thiophen-2-ylmethyl)aniline (4na)

Pale yellow solid; 73% yield; m. p. 113-115 °C; ^{1}H NMR (400 MHz, $CDCl_3$) δ 7.09-7.02 (m, 3H), 6.98 (d, J = 8.0 Hz, 2H), 6.83-6.81 (m, 1H), 6.72 (d, J = 8.0 Hz, 2H), 6.69 (d, J = 4.0 Hz, 1H), 6.65-6.61 (m, 1H), 6.56 (d, J = 8.0 Hz, 1H), 4.11 (s, 2H), 3.96 (s, 2H), 3.86 (br.s, 1H), 3.68 (s, 3H); ^{13}C NMR (100 MHz, $CDCl_3$) δ 158.8, 146.1, 143.0, 131.3, 130.1, 128.6, 128.3, 127.0, 125.3, 124.5, 124.2, 117.4, 114.1, 111.3, 55.4, 47.7, 32.8. IR (KBr): υ 1599, 1510, 1353, 1241, 1172, cm^{-1}. ESI HRMS: calcd. for $C_{19}H_{19}NOS$ +H $[M+H]^+$ 310.1266, found 310.1268.

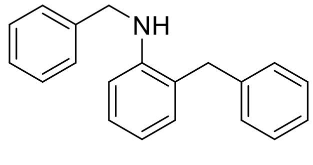

N,2-Dibenzylaniline (4ab)

Yellow solid; 73% yield; m. p. 50-52 °C; ^{1}H NMR (400 MHz, $CDCl_3$) δ 7.21-7.12 (m, 6H), 7.09 (d, J = 4.0 Hz, 2H), 7.05 (d, J = 4.0 Hz, 1H), 7.01-6.98 (m, 3H), 6.66-6.62 (m, 1H), 6.53 (d, J = 8.0 Hz, 1H), 4.16 (s, 2H), 3.82 (s, 3H); ^{13}C NMR (100 MHz, $CDCl_3$) δ 146.1, 139.5, 139.4, 130.8, 128.8, 128.7, 128.6, 128.0, 127.3, 127.2, 126.5, 124.8, 117.4, 111.0, 48.1, 38.4. IR (KBr): υ 1602, 1509, 1450, 1360, 1122 cm^{-1}. ESI HRMS: calcd. for $C_{20}H_{19}N$ +Na $[M+Na]^+$ 296.1415, found 296.1418.

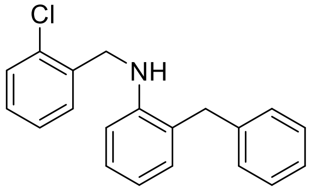

2-Benzyl-*N*-(2-chlorobenzyl)aniline (4ac)

Yellow oil; 65% yield; ^{1}H NMR (400 MHz, $CDCl_3$) δ 7.24-7.18 (m, 3H), 7.15-7.10 (m, 3H), 7.08-6.98 (m, 4H), 6.92 (d, J = 8.0 Hz, 1H), 6.66-6.63 (m, 1H), 6.46 (d, J = 12.0 Hz, 1H), 4.28 (s, 2H), 3.95 (br.s, 1H), 3.86 (s, 2H); ^{13}C NMR (100 MHz, $CDCl_3$) δ 145.6, 139.4, 136.7, 133.3, 130.9, 129.5, 128.8, 128.7, 128.6, 128.3, 128.0, 126.9, 126.6, 124.8, 117.6, 111.1, 45.5, 38.4. IR (neat): υ 1604, 1514, 1357, 1266 cm^{-1}. ESI HRMS: calcd. for $C_{20}H_{18}ClN$ +H $[M+H]^+$ 308.1206, found 308.1208.

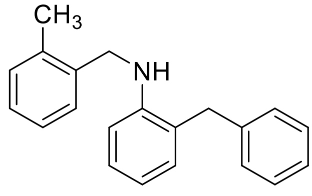

2-Benzyl-*N*-(2-methylbenzyl)aniline (4ad)

Pale yellow solid; 80% yield; m. p. 72-74 ºC; ^{1}H NMR (400 MHz, $CDCl_3$) δ 7.19-7.14 (m, 2H), 7.13-7.10 (m, 2H), 7.07-7.05 (m, 4H), 7.03-7.00 (m, 2H), 6.95 (d, J = 8.0 Hz, 1H), 6.67-6.64 (m, 1H), 6.55 (d, J = 8.0 Hz, 1H), 4.10 (s, 2H), 3.80 (s, 2H), 3.64 (br.s, 1H), 2.08 (s, 3H); ^{13}C NMR (100 MHz, $CDCl_3$) δ 146.2, 139.5, 136.9, 136.31, 130.8, 130.4, 128.8, 128.7, 128.0, 127.4, 126.5, 126.2, 124.7, 117.3, 110.7, 46.2, 38.4, 18.8. IR (KBr): υ 1599, 1509, 1355, 1264 cm^{-1}. ESI HRMS: calcd. for $C_{21}H_{21}N$ +Na $[M+Na]^+$ 310.1572, found 310.1576.

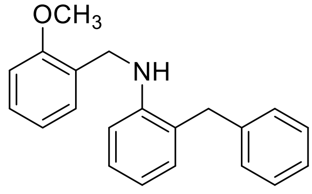

2-Benzyl-*N*-(2-methoxybenzyl)aniline (4ae)

Yellow oil; 75% yield; ^{1}H NMR (400 MHz, $CDCl_3$) δ 7.18-7.15 (m, 2H), 7.11-7.06 (m, 5H), 6.95 (d, J = 8.0 Hz, 1H), 6.91 (d, J = 8.0 Hz, 1H), 6.74-6.69 (m, 2H), 6.62-6.57 (m, 2H), 4.18 (s, 2H), 3.93 (br.s, 1H), 3.80 (s, 2H), 3.59 (s, 3H); ^{13}C NMR (100 MHz, $CDCl_3$) δ 157.4, 146.4, 139.6, 130.7, 128.7, 128.7, 128.2, 127.9, 127.3, 126.4, 124.8, 120.5, 117.2, 111.2, 110.2, 55.2, 43.3, 38.2. IR (neat): υ 1512, 1461, 1354, 1242 cm^{-1}. ESI HRMS: calcd. for $C_{21}H_{21}NO$ +H $[M+H]^+$ 304.1701, found 304.1705.

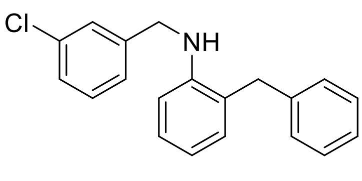

2-Benzyl-*N*-(3-chlorobenzyl)aniline (4af)

Yellow oil; 52% yield; ^{1}H NMR (400 MHz, $CDCl_3$) δ 7.22-7.18 (m, 2H), 7.14 (d, J = 8.0 Hz, 1H), 7.09-7.05 (m, 4H), 7.03-6.99 (m, 2H), 6.96 (s, 1H), 6.86 (d, J = 4.0 Hz, 1H), 6.66-6.62 (m, 1H), 6.44 (d, J = 8.0 Hz, 1H), 4.11 (s, 2H), 3.83 (s, 3H); ^{13}C NMR (100 MHz, $CDCl_3$) δ 145.6, 141.7, 139.3, 134.5, 130.9, 129.9, 128.9, 128.6, 128.0, 127.3, 127.2, 126.7, 125.3, 124.8, 117.6, 111.0, 47.5, 38.5. IR (neat): υ 1599, 1514, 1352, 1268, 1076 cm^{-1}. ESI HRMS: calcd. for $C_{20}H_{18}ClN$ +Na $[M+Na]^+$ 330.1025, found 330.1028.

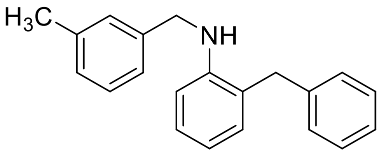

2-Benzyl-*N*-(3-methylbenzyl)aniline (4ag)

Yellow oil; 50% yield; ^{1}H NMR (400 MHz, $CDCl_3$) δ 7.22-7.18 (m, 2H), 7.16-7.14 (m, 1H), 7.11-7.05 (m, 4H), 7.00 (d, *J* = 8.0 Hz, 1H), 6.95 (d, *J* = 8.0 Hz, 1H), 6.83-6.79 (m, 2H), 6.66-6.63 (m, 1H), 6.55 (d, *J* = 8.0 Hz, 1H), 4.12 (s, 2H), 3.83 (s, 2H), 2.20 (s, 3H); ^{13}C NMR (100 MHz, $CDCl_3$) δ 146.2, 139.5, 139.3, 138.3, 130.8, 128.8, 128.7, 128.5, 128.0, 128.0, 127.9, 126.6, 124.7, 124.5, 117.3, 111.0, 48.3, 38.4, 21.5. IR (neat): υ 1603, 1513, 1453, 1353, 1266 cm^{-1}. ESI HRMS: calcd. for $C_{21}H_{21}N$ +H $[M+H]^+$ 288.1752, found 288.1750.

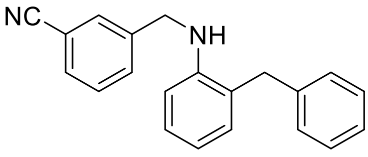

3-(((2-Benzylphenyl)amino)methyl)benzonitrile (4ah)

Yellow solid; 57% yield; m. p. 92-94 °C; ^{1}H NMR (400 MHz, $CDCl_3$) δ 7.38 (d, *J* = 8.0 Hz, 1H), 7.26-7.16 (m, 6H), 7.10 (d, *J* = 8.0 Hz, 2H), 7.05-7.01 (m, 2H), 6.68-6.65 (m, 1H), 6.38 (d, *J* = 8.0 Hz, 1H), 4.18 (s, 2H), 3.86 (s, 2H); ^{13}C NMR (100 MHz, $CDCl_3$) δ 145.2, 141.1, 139.3, 131.5, 131.1, 130.9, 130.4, 129.3, 128.9, 128.5, 128.0, 126.9, 124.9, 118.9, 117.9, 112.7, 110.9, 47.0, 38.5. IR (KBr): υ 2230, 1599, 1364, 1268, 1070 cm^{-1}. ESI HRMS: calcd. for $C_{21}H_{18}N_2$ +Na $[M+Na]^+$ 321.1368, found 321.1366.

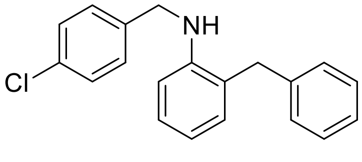

2-Benzyl-*N*-(4-chlorobenzyl)aniline (4ai)

Pale yellow solid; 66% yield; m. p. 74-75 °C; ^{1}H NMR (400 MHz, $CDCl_3$) δ 7.23-7.19 (m, 2H), 7.15 (d, *J* = 4.0 Hz, 1H), 7.12 (d, *J* = 8.0 Hz, 2H), 7.10-7.08 (m, 2H), 7.05-7.01 (m, 2H), 6.90 (d, *J* = 8.0 Hz, 2H), 6.68-6.64 (m, 1H), 6.47 (d, *J* = 8.0 Hz, 1H), 4.13 (s, 2H), 3.84 (s, 2H); ^{13}C NMR (100 MHz, $CDCl_3$) δ 145.7, 139.4, 138.0, 132.8, 130.9, 128.9, 128.8, 128.6, 128.5, 128.0, 126.6, 124.8, 117.6, 111.1, 47.4, 38.5. IR (KBr): υ 1604, 1513, 1453, 1269, 1091, 811 cm^{-1}. ESI HRMS: calcd. for $C_{20}H_{18}ClN$ +H $[M+H]^+$ 308.1206, found 308.1209.

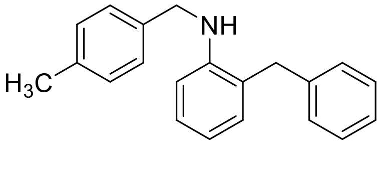

2-Benzyl-*N*-(4-methylbenzyl)aniline (4aj)

Yellow oil; 64% yield; ^{1}H NMR (400 MHz, $CDCl_3$) δ 7.22-7.18 (m, 2H), 7.15 (d, *J* = 4.0 Hz, 1H), 7.09 (d, *J* = 4.0 Hz, 2H), 7.05 (d, *J* = 8.0 Hz, 1H), 6.99-6.97 (m, 3H), 6.89 (d, *J* = 8.0 Hz, 2H), 6.65-6.62 (m, 1H), 6.54 (d, *J* = 8.0 Hz, 1H), 4.12 (s, 2H), 3.82 (s, 2H), 3.78 (br.s, 1H), 2.23 (s, 3H); ^{13}C NMR (100 MHz, $CDCl_3$) δ 146.1, 139.5, 136.8, 136.3, 130.7, 129.3, 128.8, 128.7, 128.0, 127.3, 126.5, 124.7, 117.3, 111.0, 47.9, 38.4, 21.2. IR (neat): υ 1604, 1513, 1453, 1354, 802 cm^{-1}. ESI HRMS: calcd. for $C_{21}H_{21}N$ +H $[M+H]^+$ 288.1752, found 288.1750.

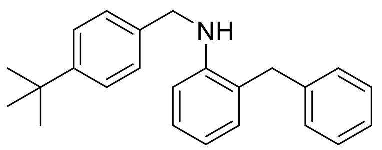

2-Benzyl-*N*-(4-(*tert*-butyl)benzyl)aniline (4ak)

Yellow oil; 66% yield; ^{1}H NMR (400 MHz, CDCl$_3$) δ 7.22-7.20 (m, 4H), 7.16 (d, *J* = 8.0 Hz, 1H), 7.11-7.06 (m, 3H), 7.00-6.96 (m, 3H), 6.67-6.63 (m, 1H), 6.58 (d, *J* = 8.0 Hz, 1H), 4.15 (s, 2H), 3.83 (s, 2H), 1.23 (s, 9H); ^{13}C NMR (100 MHz, CDCl$_3$) δ 150.1, 146.2, 139.5, 136.3, 130.7, 128.8, 128.8, 128.0, 127.1, 126.5, 125.6, 124.8, 117.3, 111.0, 47.9, 38.3, 34.6, 31.5. IR (neat): υ 1603, 1513, 1364, 1266, 746 cm^{-1}. ESI HRMS: calcd. for $C_{24}H_{27}N$ +Na [M+Na]$^+$ 352.2041, found 352.2045.

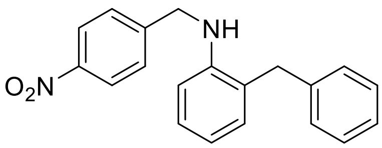

2-Benzyl-*N*-(4-nitrobenzyl)aniline (4al)

Yellow solid; 76% yield; m. p. 97-99 ºC; ^{1}H NMR (400 MHz, CDCl$_3$) δ 8.05 (d, *J* = 12.0 Hz, 2H), 7.32-7.28 (m, 2H), 7.25 (d, *J* = 8.0 Hz, 1H), 7.20-7.16 (m, 4H), 7.13 (d, *J* = 4.0 Hz, 1H), 7.09 (d, *J* = 8.0 Hz, 1H), 6.77-6.73 (m, 1H), 6.44 (d, *J* = 8.0 Hz, 1H), 4.35 (s, 2H), 3.96 (s, 2H); ^{13}C NMR (100 MHz, CDCl$_3$) δ 147.4, 147.1, 145.1, 139.3, 131.2, 128.9, 128.6, 128.0, 127.6, 126.7, 124.9, 123.8, 118.0, 111.0, 47.2, 38.5. IR (KBr): υ 1602, 1519, 1453, 1343, 1264, 759 cm^{-1}. ESI HRMS: calcd. for $C_{20}H_{18}N_2O_2$ +H [M+H]$^+$ 319.1447, found 319.1445.

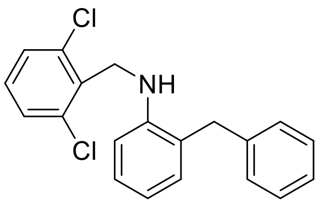

2-Benzyl-*N*-(2,6-dichlorobenzyl)aniline (4am)

Pale yellow oil; 57% yield; ^{1}H NMR (400 MHz, CDCl$_3$) δ 7.17-7.12 (m, 5H), 7.10-7.07 (m, 1H), 7.05-7.01 (m, 3H), 6.95 (d, *J* = 8.0 Hz, 1H), 6.83 (d, *J* = 8.0 Hz, 1H), 6.68-6.65 (m, 1H), 4.41 (s, 2H), 3.80 (br.s, 1H), 3.76 (s, 2H); ^{13}C NMR (100 MHz, CDCl$_3$) δ 145.7, 139.4, 138.0, 132.8, 130.9, 128.9, 128.8, 128.6, 128.5, 128.0, 126.6, 124.8, 117.6, 111.1, 47.4, 38.5. IR (neat): υ 1602, 1436, 1353, 1255, 1189 cm^{-1}. ESI HRMS: calcd. for $C_{20}H_{17}Cl_2N$ +Na [M+Na]$^+$ 364.0636, found 364.0638.

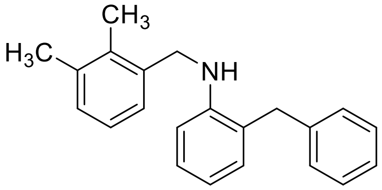

2-Benzyl-*N*-(2,3-dimethylbenzyl)aniline (4an)

Pale yellow oil; 77% yield; ^{1}H NMR (400 MHz, CDCl$_3$) δ 7.17-7.08 (m, 4H), 7.04 (d, *J* = 4.0 Hz, 2H), 6.98 (d, *J* = 8.0 Hz, 2H), 6.93-6.89 (m, 1H), 6.85 (d, *J* = 8.0 Hz, 1H), 6.66-6.63 (m, 1H), 6.58 (d, *J* = 8.0 Hz, 1H), 4.09 (s, 2H), 3.76 (s, 2H), 3.57 (br.s, 1H), 2.17 (s, 3H), 1.90 (s, 3H); ^{13}C NMR (100 MHz, CDCl$_3$) δ 146.2, 139.5, 137.1, 136.8, 135.1, 130.7, 129.2, 128.7, 128.7, 128.0, 126.5, 126.4, 125.6, 124.7, 117.2, 110.7, 47.0, 38.3, 20.5, 14.5. IR (neat): υ 1603, 1513, 1453, 1354, 1264 cm^{-1}. ESI HRMS: calcd. for $C_{22}H_{23}N$ +Na [M+Na]$^+$ 324.1728,

found 324.1726.

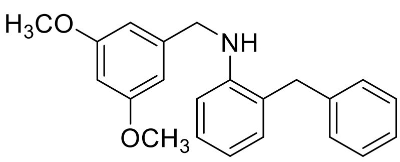

2-Benzyl-*N*-(3,5-dimethoxybenzyl)aniline (4ao)

Yellow oil; 55% yield; ^{1}H NMR (400 MHz, $CDCl_3$) δ 7.21-7.18 (m, 2H), 7.14-7.10 (m, 3H), 7.08-7.04 (m, 1H), 7.00 (d, *J* = 8.0 Hz, 1H), 6.66-6.63 (m, 1H), 6.52 (d, *J* = 8.0 Hz, 1H), 6.25 (s, 3H), 4.12 (s, 2H), 3.84 (s, 2H), 3.63 (s, 6H); ^{13}C NMR (100 MHz, $CDCl_3$) δ 161.1, 146.0, 142.1, 139.5, 130.8, 128.8, 128.6, 128.0, 126.5, 124.7, 117.4, 111.1, 105.1, 99.3, 55.4, 48.3, 38.3. IR (neat): υ 1597, 1357, 1261, 1203, 1066 cm^{-1}. ESI HRMS: calcd. for $C_{22}H_{23}NO_2$ +Na $[M+Na]^+$ 356.1626, found 356.1624.

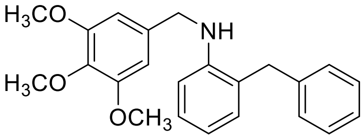

2-Benzyl-*N*-(3,4,5-trimethoxybenzyl)aniline (4ap)

Pale yellow solid; 78% yield; m. p. 95-96 ºC; ^{1}H NMR (400 MHz, $CDCl_3$) δ 7.21-7.18 (m, 2H), 7.14-7.07 (m, 4H), 7.04 (d, *J* = 8.0 Hz, 1H), 6.70-6.66 (m, 1H), 6.55 (d, *J* = 8.0 Hz, 1H), 6.30 (s, 2H), 4.12 (s, 2H), 3.86 (s, 2H), 3.74 (s, 3H), 3.67 (s, 6H); ^{13}C NMR (100 MHz, $CDCl_3$) δ 153.4, 145.9, 139.5, 137.0, 135.1, 130.8, 128.7, 128.5, 128.0, 126.4, 124.5, 117.5, 111.0, 104.0, 60.9, 56.1, 48.4, 38.3. IR (KBr): υ 1598, 1508, 1352, 1234, 1124 cm^{-1}. ESI HRMS: calcd. for $C_{23}H_{25}NO_3$ +H $[M+H]^+$ 364.1913, found 364.1916.

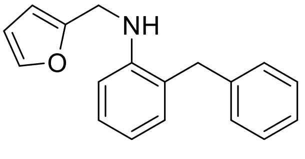

2-Benzyl-*N*-(furan-2-ylmethyl)aniline (4aq)

Yellow oil; 62% yield; ^{1}H NMR (400 MHz, $CDCl_3$) δ 7.20-7.17 (m, 3H), 7.14-7.07 (m, 4H), 6.97 (d, *J* = 8.0 Hz, 1H), 6.68-6.64 (m, 1H), 6.61 (d, *J* = 8.0 Hz, 1H), 6.18-6.16 (m, 1H), 5.92 (d, *J* = 4.0 Hz, 1H), 4.15 (s, 2H), 3.81 (s, 3H); ^{13}C NMR (100 MHz, $CDCl_3$) δ 152.9, 145.7, 141.8, 139.3, 130.7, 128.8, 128.7, 127.9, 126.5, 125.2, 117.9, 111.2, 110.4, 106.6, 41.4, 38.1. IR (neat): υ 1603, 1513, 1364, 1259, 1146 cm^{-1}. ESI HRMS: calcd. for $C_{18}H_{17}NO$ +Na $[M+Na]^+$ 286.1208, found 286.1206.

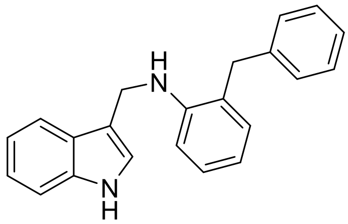

***N*-((1*H*-Indol-3-yl)methyl)-2-benzylaniline (4ar)**

Brownish red oil; 60% yield; ^{1}H NMR (400 MHz, $CDCl_3$) δ 7.88 (s, 1H), 7.31 (d, *J* = 8.0 Hz, 1H), 7.26 (d, *J* = 8.0 Hz, 1H), 7.16-7.10 (m, 6H), 7.04 (d, *J* = 8.0 Hz, 2H), 6.98 (d, *J* = 8.0 Hz, 2H), 6.85 (s, 1H), 6.74 (d, *J* = 8.0 Hz, 1H), 6.69-6.65 (m, 1H), 4.34 (s, 2H), 3.77 (s, 2H); ^{13}C NMR (100 MHz, $CDCl_3$) δ 146.4, 139.5, 130.6, 128.8, 128.7, 128.0, 126.7, 126.4, 124.8, 122.6, 122.3, 119.8, 119.0, 117.2, 113.9, 111.2, 110.9, 40.1, 38.1. IR (neat): υ 1598,

1454, 1364, 1264, 1072 cm^{-1}. ESI HRMS: calcd. for $C_{22}H_{20}N_2$ +Na $[M+Na]^+$ 335.1524, found 335.1526.

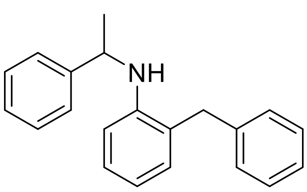

2-Benzyl-*N*-(1-phenylethyl)aniline (4as)

Yellow solid; 74% yield; m. p. 80-81 ºC; ^1H NMR (400 MHz, $CDCl_3$) δ 7.25-7.21 (m, 2H), 7.16-7.10 (m, 5H), 7.08-7.04 (m, 1H), 7.00-6.95 (m, 3H), 6.93-6.89 (m, 1H), 6.58-6.54 (m, 1H), 6.28 (d, *J* = 8.0 Hz, 1H), 4.31-4.30 (m, 1H), 3.87 (s, 2H), 3.81(s, 1H), 1.20 (d, *J* = 8.0 Hz, 3H); ^{13}C NMR (100 MHz, $CDCl_3$) δ 145.3, 139.7, 130.8, 128.8, 128.7, 128.6, 127.9, 126.8, 126.7, 125.8, 124.5, 117.0, 112.0, 53.2, 38.8, 25.1. IR (KBr): υ 1601, 1513, 1450, 1367, 1068 cm^{-1}. ESI HRMS: calcd. for $C_{21}H_{21}N$ +H $[M+H]^+$ 288.1752, found 288.1754.

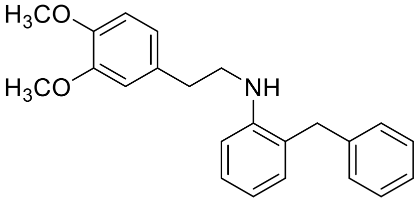

2-Benzyl-*N*-(3,4-dimethoxyphenethyl)aniline (4at)

Yellow oil; 53% yield; ^1H NMR (400 MHz, $CDCl_3$) δ 7.15-7.08 (m, 4H), 6.97-6.94 (m, 3H), 6.67-6.61 (m, 3H), 6.57-6.55 (m, 2H), 3.78 (s, 3H), 3.73 (s, 3H), 3.67 (s, 2H), 3.44 (br.s, 1H), 3.23 (t, *J* = 12.0 Hz, 2H), 2.67 (t, *J* = 12.0 Hz, 2H); ^{13}C NMR (100 MHz, $CDCl_3$) δ 149.1, 147.7, 146.2, 139.3, 131.7, 130.7, 128.6, 128.5, 127.9, 126.3, 124.9, 120.7, 117.3, 111.9, 111.4, 110.9, 56.0, 55.9, 45.0, 38.0, 35.1. IR (neat): υ 1603, 1514, 1453, 1365, 1262 cm^{-1}. ESI HRMS: calcd. for $C_{23}H_{25}NO_2$ +Na $[M+Na]^+$ 370.1783, found 370.1780.

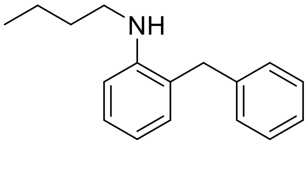

2-Benzyl-*N*-butylaniline (4au)

Yellow oil; 64% yield; ^1H NMR (400 MHz, $CDCl_3$) δ 7.21-7.17 (m, 2H), 7.13-7.09 (m, 4H), 6.97 (d, *J* = 8.0 Hz, 1H), 6.63-6.60 (m, 1H), 6.55 (d, *J* = 8.0 Hz, 1H), 3.79 (s, 2H), 3.35 (br.s, 1H), 2.93 (t, *J* = 12.0 Hz, 2H), 1.40-1.32 (m, 2H), 1.16-1.07 (m, 2H), 0.76 (t, *J* = 16.0 Hz, 3H); ^{13}C NMR (100 MHz, $CDCl_3$) δ 146.6, 139.6, 130.7, 128.8, 128.6, 128.0, 126.5, 124.6, 116.8, 43.5, 38.4, 31.4, 20.2, 13.9. IR (neat): υ 1604, 1513, 1453, 1314, 1267 cm^{-1}. ESI HRMS: calcd. for $C_{17}H_{21}N$ +Na $[M+Na]^+$ 262.1572, found 262.1573.

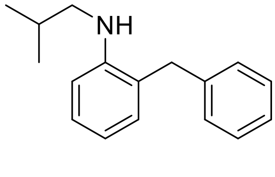

2-Benzyl-*N*-isobutylaniline (4av)

Yellow oil; 72% yield; ^1H NMR (400 MHz, $CDCl_3$) δ 7.21-7.18 (m, 2H), 7.13-7.08 (m, 4H), 6.99 (d, *J* = 8.0 Hz, 1H), 6.64-6.60 (m, 1H), 6.53 (d, *J* = 8.0 Hz, 1H), 3.81 (s, 2H), 3.44 (br.s, 1H), 2.76 (d, *J* = 4.0 Hz, 2H), 1.69-1.62 (m, 1H), 0.71(s, 3H), 0.69 (s, 3H); ^{13}C NMR (100 MHz, $CDCl_3$) δ 146.6, 139.6, 130.8, 128.8, 128.6, 128.0, 126.6, 124.5, 116.7, 110.5, 51.7, 38.6, 27.8, 20.4. IR

(neat): υ 1602, 1364, 1259, 1070 cm^{-1}. ESI HRMS: calcd. for $C_{17}H_{21}N$ +H $[M+H]^+$ 240.1752, found 240.1750.

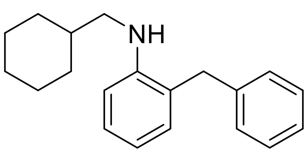

2-Benzyl-*N*-(cyclohexylmethyl)aniline (4aw)

Yellow oil; 54% yield; ^1H NMR (400 MHz, $CDCl_3$) δ 7.21-7.17 (m, 2H), 7.13-7.08 (m, 4H), 6.98 (d, *J* = 8.0 Hz, 1H), 6.63-6.59 (m, 1H), 6.53 (d, *J* = 8.0 Hz, 1H), 3.80 (s, 2H), 3.45 (br.s, 1H), 2.78 (d, *J* = 4.0 Hz, 2H), 1.55-1.54 (m, 3H), 1.43-1.40 (m, 2H), 1.34-1.31 (m, 1H), 1.09-1.03 (m, 3H), 0.74-0.68 (m, 2H); ^{13}C NMR (100 MHz, $CDCl_3$) δ 146.6, 139.7, 130.8, 128.8, 128.6, 127.9, 126.5, 124.5, 116.6, 110.5, 50.4, 38.6, 37.4, 31.1, 26.6, 26.1. IR (neat): υ 1604, 1513, 1450, 1314, 1263 cm^{-1}. ESI HRMS: calcd. for $C_{20}H_{25}N$ +Na $[M+Na]^+$ 302.1885, found 302.1883.

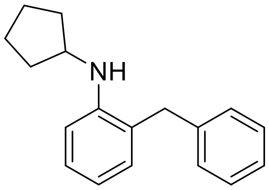

2-Benzyl-*N*-cyclopentylaniline (4ax)

Yellow solid; 61% yield; m. p. 67-69 ºC; ^1H NMR (400 MHz, $CDCl_3$) δ 7.21-7.17 (m, 2H), 7.13-7.07 (m, 4H), 6.97 (d, *J* = 8.0 Hz, 1H), 6.62-6.57 (m, 2H), 3.77 (s, 2H), 3.67-3.64 (m, 1H), 3.38 (br.s, 1H), 1.79-1.74 (m, 2H), 1.41-1.36 (m, 4H), 1.22-1.18 (m, 2H); ^{13}C NMR (100 MHz, $CDCl_3$) δ 146.1, 139.7, 130.8, 128.8, 128.6, 127.9, 126.5, 124.7, 116.6, 111.6, 54.4, 38.6, 33.5, 23.9. IR (KBr): υ 1604, 1511, 1453, 1354, 1073 cm^{-1}. ESI HRMS: calcd. for $C_{18}H_{21}N$ +Na $[M+Na]^+$ 274.1572, found 274.1575.

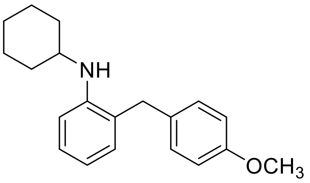

***N*-Cyclohexyl-2-(4-methoxybenzyl)aniline (4py)**

Yellow oil; 44% yield; ^1H NMR (400 MHz, $CDCl_3$) δ 7.09-7.05 (m, 1H), 7.02 (d, *J* = 8.0 Hz, 2H), 6.96 (d, *J* = 4.0 Hz, 1H), 6.75 (d, *J* = 12.0 Hz, 2H), 6.60-6.56 (m, 2H), 3.72 (s, 2H), 3.70 (s, 3H), 3.37 (br.s, 1H), 3.17-3.13 (m, 1H), 1.79-1.77 (m, 2H), 1.52-1.49 (m, 3H), 1.23-1.18 (m, 2H), 1.11-1.06 (m, 1H), 0.97-0.88 (m, 2H); ^{13}C NMR (100 MHz, $CDCl_3$) δ 158.3, 145.5, 131.6, 130.8, 129.6, 127.8, 124.9, 116.4, 114.2, 111.2, 55.4, 51.2, 37.7, 33.2, 26.0, 24.7. IR (neat): υ 1602, 1511, 1355, 1246 cm^{-1}. ESI HRMS: calcd. for $C_{20}H_{25}NO$ +H $[M+H]^+$ 296.2014, found 296.2016.

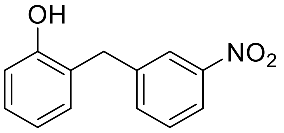

2-(3-Nitrobenzyl)phenol (5f)

Pale yellow oil; 43% yield; ^1H NMR (400 MHz, $CDCl_3$) δ 8.03 (s, 1H), 7.96 (d, *J* = 8.0 Hz, 1H), 7.49 (d, *J* = 8.0 Hz, 1H), 7.36-7.32 (m, 1H), 7.08-7.04 (m, 2H), 6.84-6.81 (m, 1H), 6.69 (d, *J* = 4.0 Hz, 1H), 5.06 (s, 1H), 4.00 (s, 2H); ^{13}C NMR (100 MHz, $CDCl_3$) δ 153.5, 148.4, 143.0, 135.2, 131.0, 129.3. 128.4, 126.1, 123.8, 121.2, 115.7, 35.9. IR (neat): υ 3450, 1595, 1528, 1355, 1096

cm^{-1}. ESI HRMS: calcd. for $C_{13}H_{11}NO_3$+H $[M+H]^+$ 230.0817, found 230.0815.

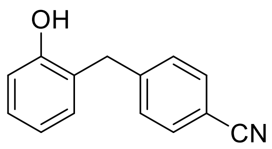

4-(2-Hydroxybenzyl)benzonitrile (5o)

White solid; 93% yield; m. p. 124-126 °C; ^{1}H NMR (400 MHz, $CDCl_3$) δ 7.53 (d, *J* = 12.0 Hz, 2H), 7.32 (d, *J* = 8.0 Hz, 2H), 7.14-7.10 (m, 1H), 7.08 (d, *J* = 8.0 Hz, 1H), 6.90-6.86 (m, 1H), 6.78 (d, *J* = 8.0 Hz, 1H), 5.37 (s, 1H), 4.02 (s, 2H); ^{13}C NMR (100 MHz, $CDCl_3$) δ 153.8, 147.0, 132.2, 131.0, 128.2, 125.9, 120.9, 119.2, 115.6, 109.3, 36.3. IR (KBr): υ 3364, 2239, 1591, 1454, 1358, 1225, 756 cm^{-1}. ESI HRMS: calcd. for $C_{14}H_{11}NO$+H $[M+H]^+$ 210.0919, found 210.0917.

IV. Gram-scale synthesis of 4aa.

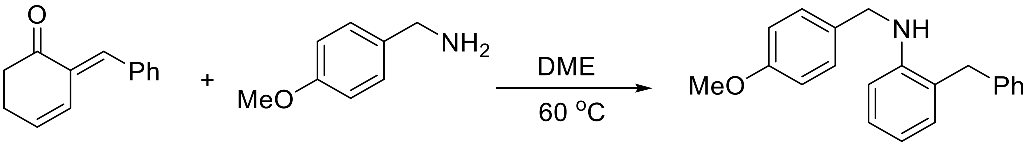

2a, on 6.0 mmol scale **3a** **4aa**, 13 h, 74% (1.35 g)

In a vial containing a magnetic stirrer was placed (*E*)-2-benzylidenecyclohex-3-en-1-one (**2a**, 1.11 g, 6.0 mmol), (4-methoxyphenyl)methanamine (**3a**, 8.23 g, 60.0 mmol) and DME (60 mL). The reaction mixture was stirred at 60 °C and the reaction process was monitored by TLC analysis. After completion, the solvent was concentrated under reduced pressure and the residue was purified by column chromatography on silica gel to give product **4aa** in 74% yield.

V. Successive one-pot synthesis of 4aa.

(4-Methoxyphenyl)methanamine (**3a**, 0.27 g, 2.0 mmol) was added to a solution of (*E*)-2-benzylidenecyclohex-3-en-1-one (**2a**, 36.8 mg, 0.2 mmol) in DME (2 mL). The reaction mixture was stirred at 60 °C and monitored by TLC analysis. Upon full conversion of **2a**, **3a** (27.4 mg, 0.2 mmol, 1 equiv) and **2a** (36.8 mg, 0.2 mmol, 1 equiv) were added synchronously. After running five times of the experiments, the solvent was concentrated under reduced pressure and the residue was purified by column chromatography on silica gel to give product **4aa** in 65% yield.

VI. Derivation of the products

1. Procedure for preparation of 2-benzylaniline (6)

In a vial containing a magnetic stirrer was added 2-benzyl-*N*-(4-methoxybenzyl)aniline (**4aa**, 60.6 mg, 0.2

mmol), $Pd(OH)_2$ (12.2 mg, 20 wt %) and MeOH (10 mL) under H_2 atmosphere (1 atm). The reaction mixture was stirred at 40 °C and monitored by TLC. After 3 h, the reaction mixture was quenched with water and then extracted three times with ethyl acetate. The combined organic layer was washed successively with water and dried over $NaSO_4$. The solvent was concentrated under reduced pressure and the residue was purified by column chromatography on silica gel (eluent: petroleum ether/ethyl acetate = 20:1) to give product **6**.

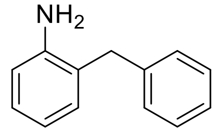

2-Benzylaniline (6)

Pale yellow solid; 82% yield, m. p. 53-55 °C; ^{1}H NMR (400 MHz, $CDCl_3$) δ 7.23-7.19 (m, 2H), 7.16-7.10 (m, 3H), 7.04-6.98 (m, 2H), 6.71-6.68 (m, 1H), 6.60 (d, *J* = 8.0 Hz, 1H), 3.83 (s, 2H), 3.13 (br.s, 2H); ^{13}C NMR (100 MHz, $CDCl_3$) δ 144.8, 139.5, 131.0, 128.8, 128.6, 127.8, 126.5, 125.2, 118.9, 116.1, 38.2. IR (KBr): υ 1593, 1365, 1265, 1067 cm^{-1}. ESI HRMS: calcd. for $C_{13}H_{13}N$+Na $[M+Na]^+$ 206.0946, found 206.0942.

2. Procedure for the preparation of 2-benzyl-*N*-cyclopentyl-*N*-methylaniline (7)

2-Benzyl-*N*-cyclopentylaniline (**4ax**, 50.2 mg, 0.2 mmol), NaOH (16.0 mg, 0.4 mmol) and CH_3CN (10 mL) were added to a round-bottom flask within a magnetic stirrer. After stirring for 30 min at room temperature, MeI (249.0 μL, 4.0 mmol) was added dropwise and the reaction mixture was continued to stir at room temperature for 72 h. After completion, the solvent was removed under vacuum and the residue was purified by silica gel column chromatography (eluent: petroleum ether/ethyl acetate = 60:1) to give product **7**.

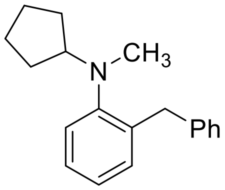

2-Benzyl-*N*-cyclopentyl-*N*-methylaniline (7)

Pale yellow oil; 98% yield; ^{1}H NMR (400 MHz, $CDCl_3$) δ 7.24-7.12 (m, 7H), 7.08 (d, *J* = 4.0 Hz, 1H), 7.00-6.97 (m, 1H), 4.09 (s, 2H), 3.38-3.34 (m, 1H), 2.48 (s, 3H), 1.71-1.65 (m, 2H), 1.61-1.58 (m, 2H), 1.49-1.45 (m, 2H), 1.41-1.34 (m, 2H); ^{13}C NMR (100 MHz, $CDCl_3$) δ 152.8, 142.1, 138.4, 130.6, 129.3, 128.2, 126.9, 125.7, 124.2, 123.1, 65.1, 42.6, 36.9, 31.5, 24.4. IR (neat): υ 1597, 1492, 1451, 1355, 1268 cm^{-1}. ESI HRMS: calcd. for $C_{19}H_{23}N$+Na $[M+Na]^+$ 288.1728, found 288.1726.

VII. References.

[1] Ren, H.-X.; Song, X.-J.; Wu, L.; Huang, Z.-C.; Zou, Y.; Li, X.; Chen, X.-W.; Tian, F.; Wang, L.-X. *Eur. J. Org. Chem.* **2019**, 715-719.

[2] Jiang, L.; Li, L.; Li, M.; Yuan, M.-W.; Yuan, M.-L. *Synthetic Commun.* **2023**, *53*, 1520-1528.

[3] Rosamilia, A. E.; Scott, J. L.; Strauss, C. R. *Org. Lett.* **2005**, *7*, 1525-1528.

VIII. Copies of ¹H and ¹³C NMR spectra of new compounds.

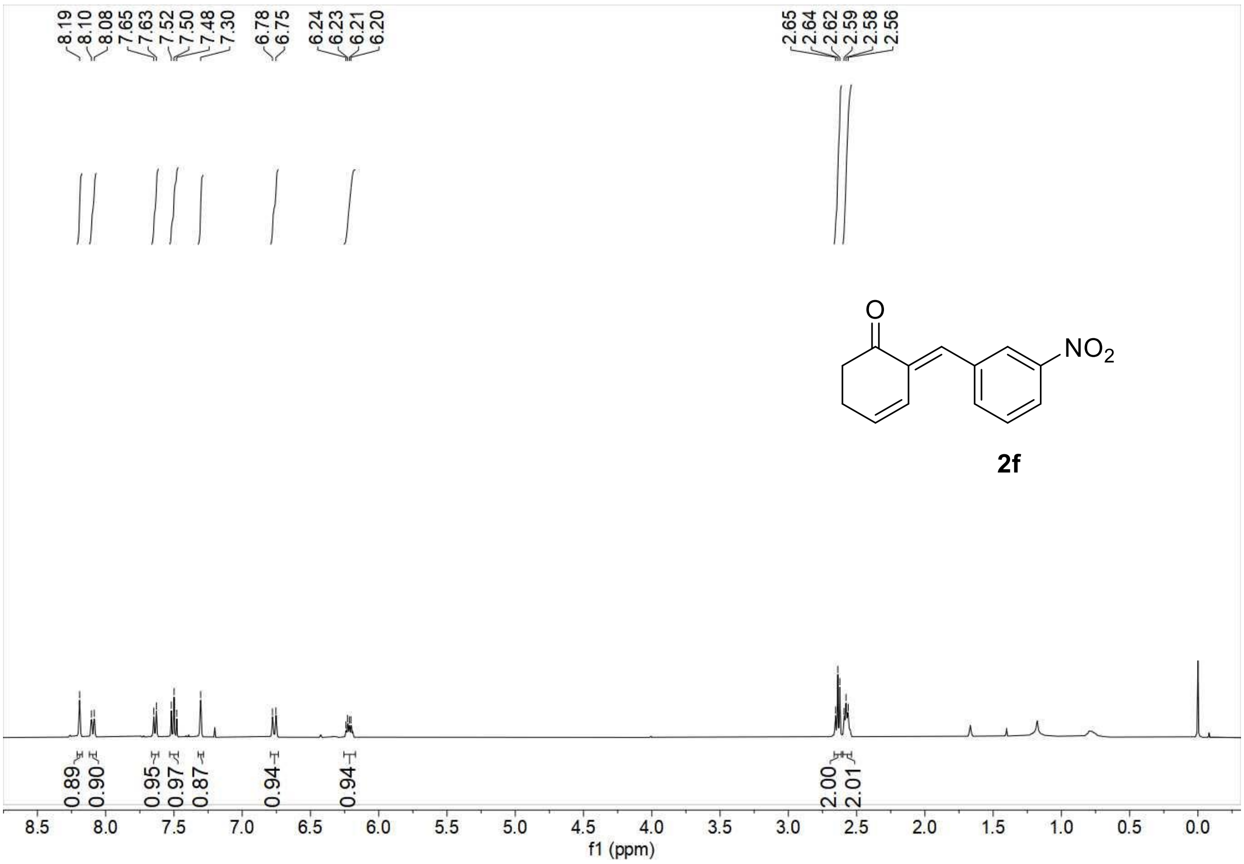

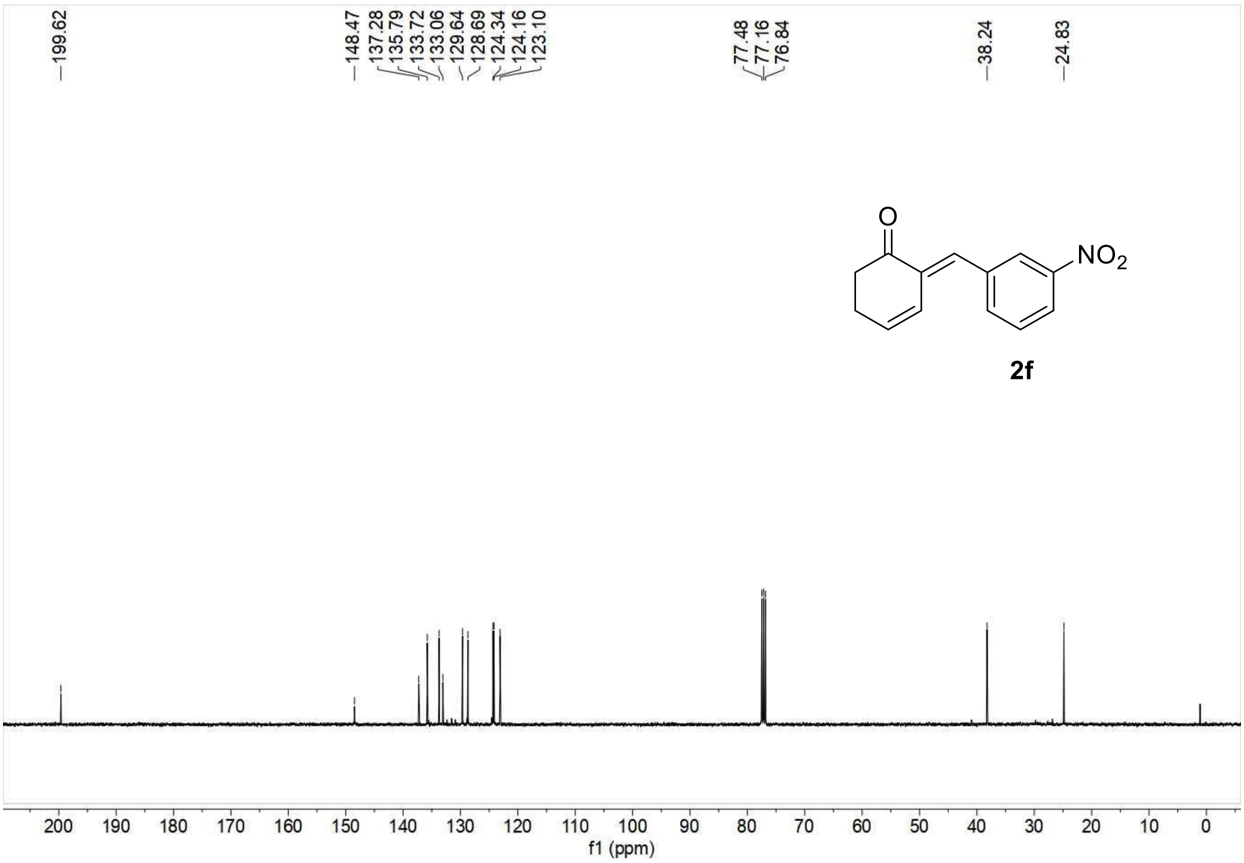

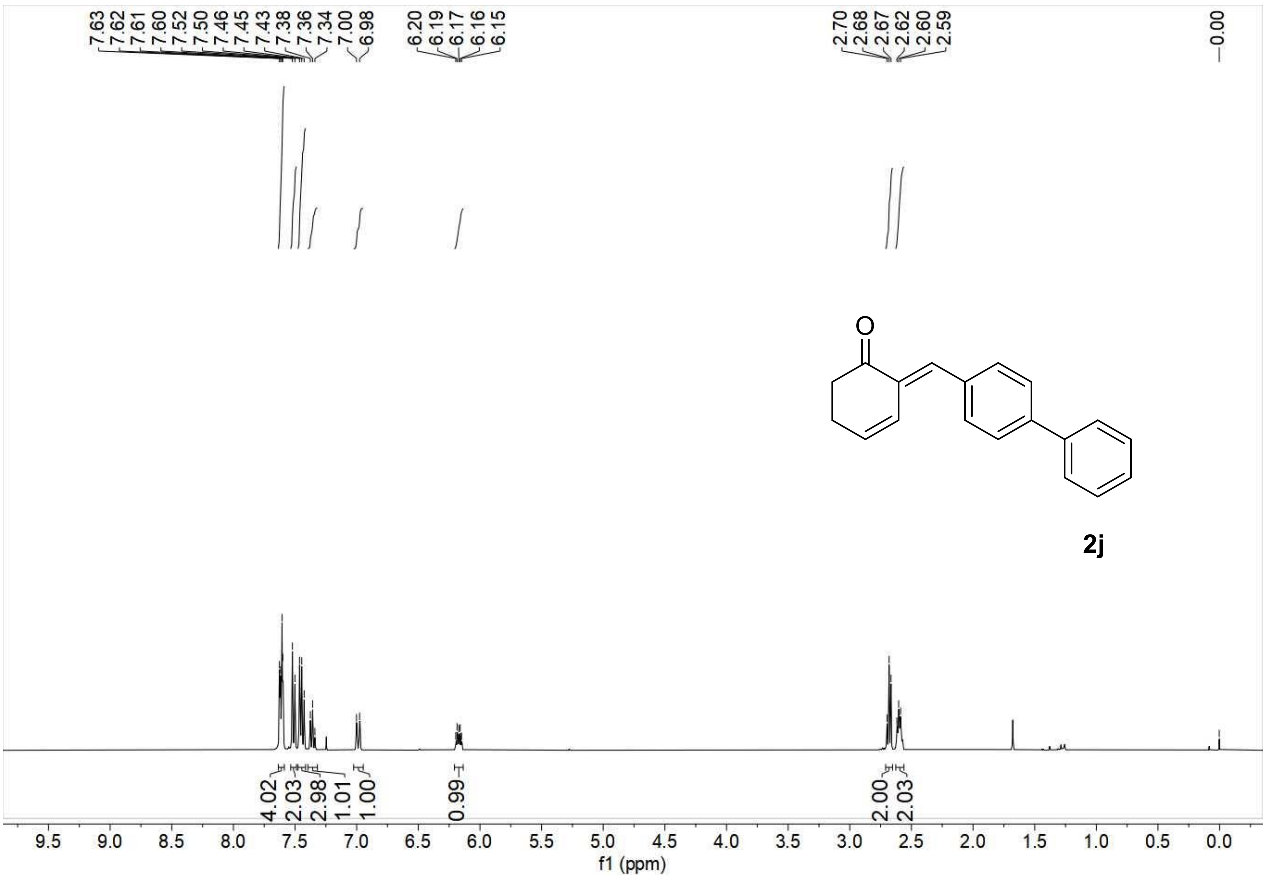
7.63 7.62 7.61 7.60 7.52 7.50 7.46 7.45 7.43 7.38 7.36 7.34 7.00 6.98
6.20 6.19 6.17 6.16 6.15
2.70 2.68 2.67 2.62 2.60 2.59
0.00
O
2j
4.02 2.03 2.98 1.01 1.00
0.99
2.00 2.03
f1 (ppm)

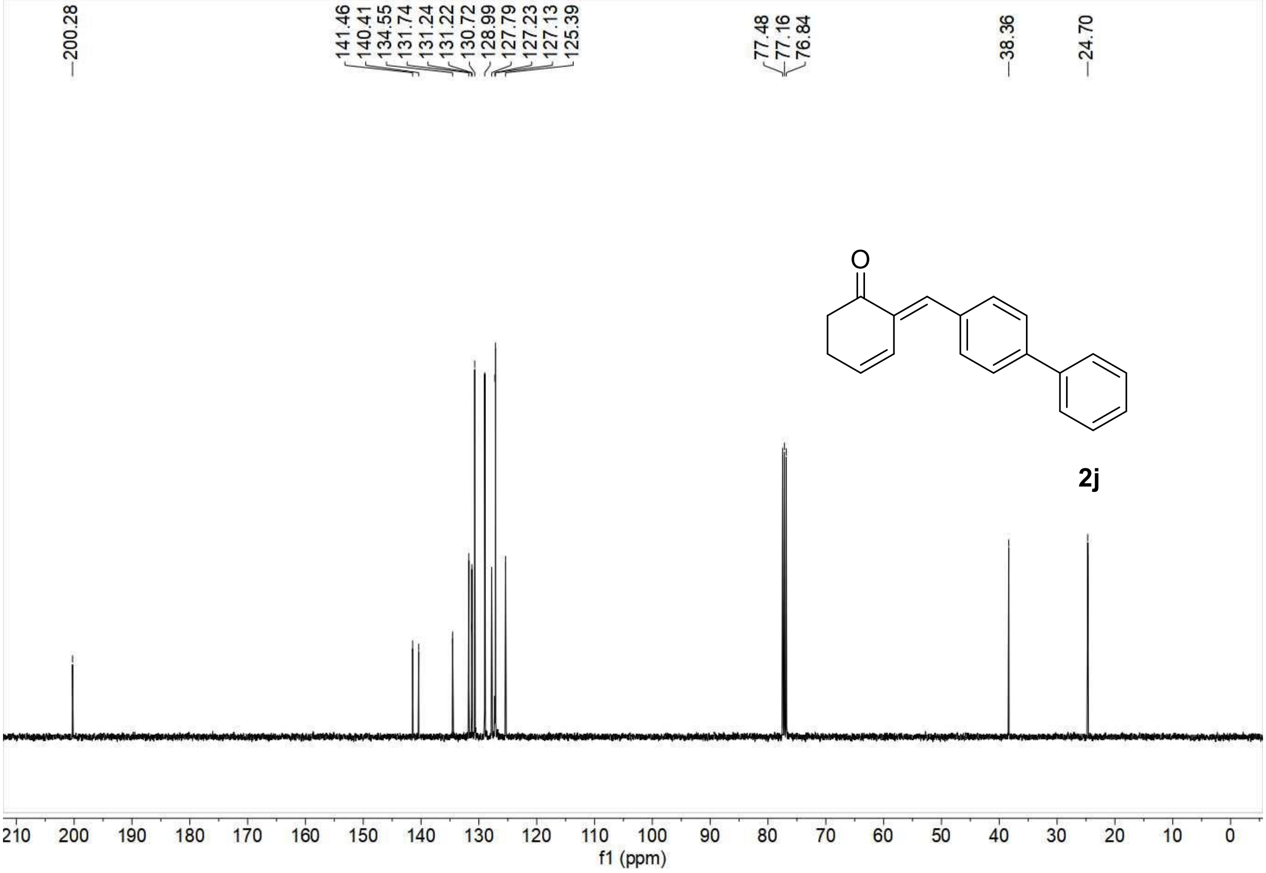
200.28
141.46 140.41 134.55 131.74 131.24 131.22 130.72 128.99 127.79 127.23 127.13 125.39
77.48 77.16 76.84
38.36
24.70
O
2j
f1 (ppm)

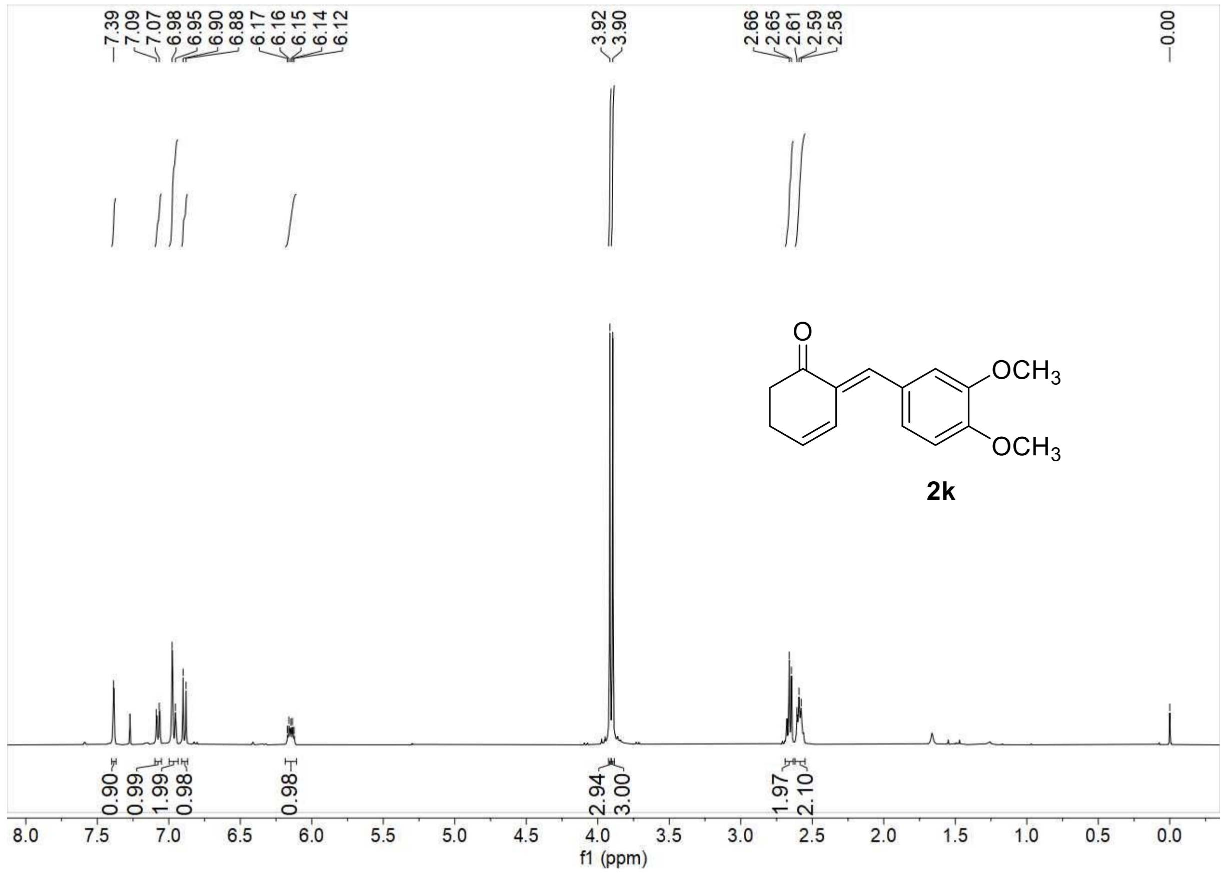
7.39
7.09
7.07
6.98
6.95
6.90
6.88
6.17
6.16
6.15
6.14
6.12
3.92
3.90
2.66
2.65
2.61
2.59
2.58
0.00
O
OCH3
OCH3
2k
0.90
0.99
1.99
0.98
0.98
2.94
3.00
1.97
2.10
f1 (ppm)

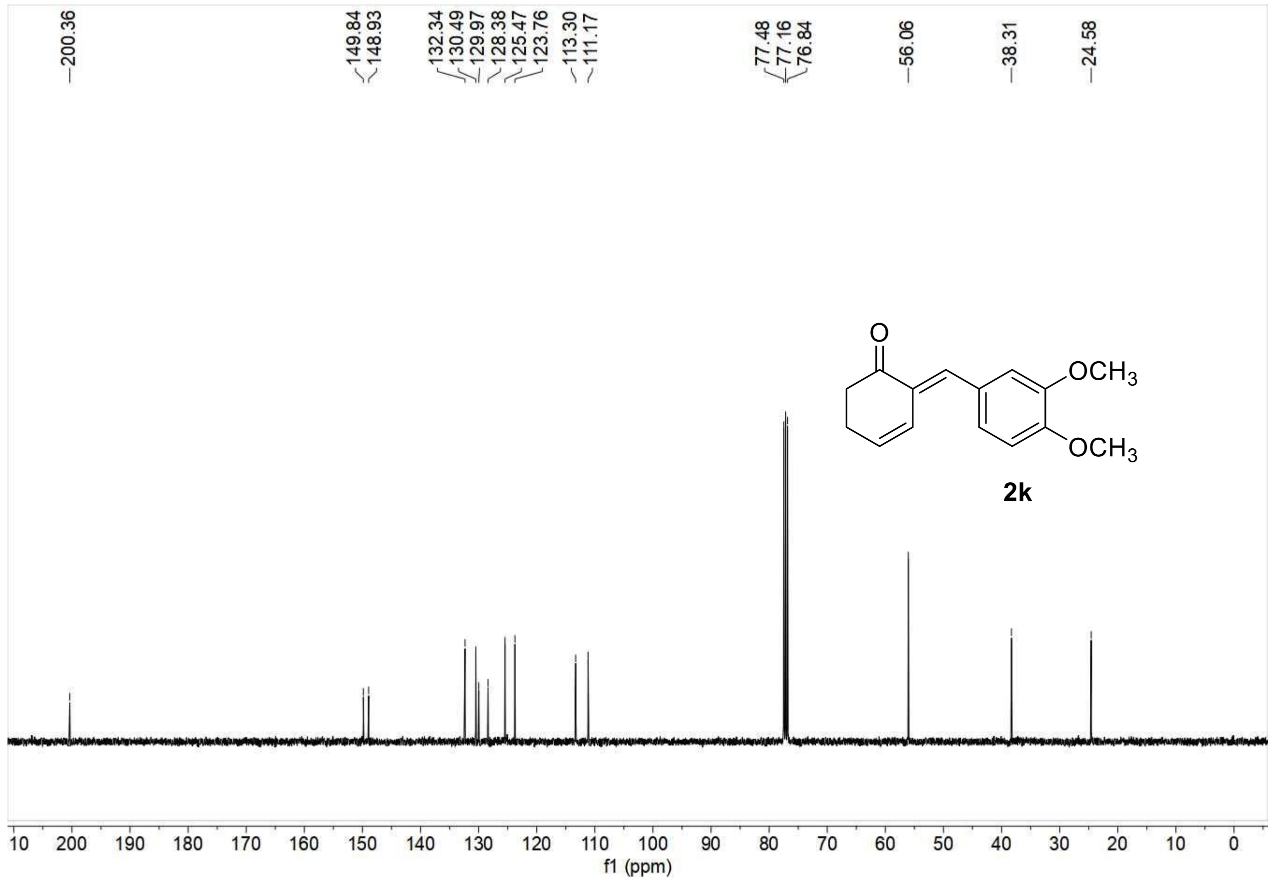
200.36
149.84
148.93
132.34
130.49
129.97
128.38
125.47
123.76
113.30
111.17
77.48
77.16
76.84
56.06
38.31
24.58
O
OCH3
OCH3
2k
f1 (ppm)

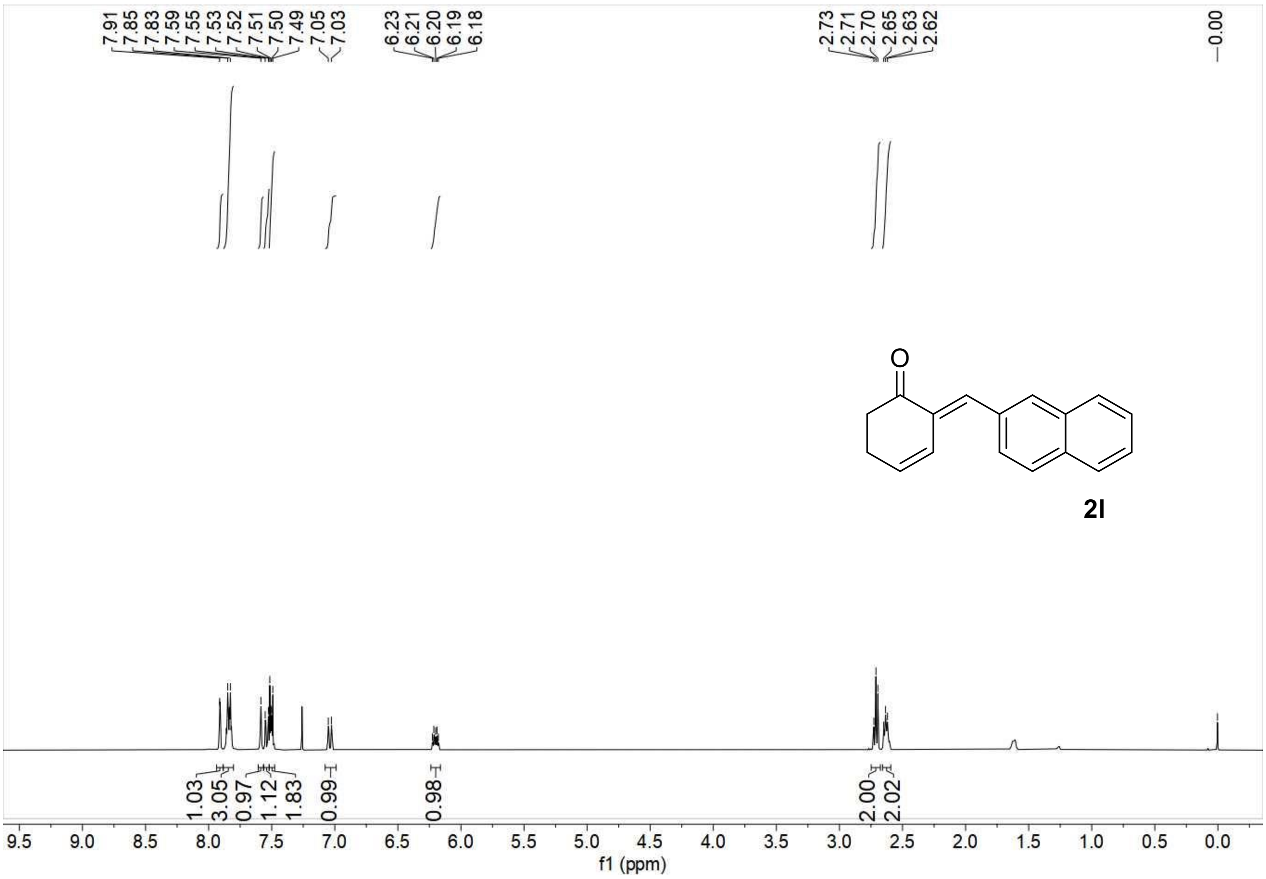
7.91
7.85
7.83
7.59
7.55
7.53
7.52
7.51
7.50
7.49
7.05
7.03
6.23
6.21
6.20
6.19
6.18
2.73
2.71
2.70
2.65
2.63
2.62
0.00
O
2l
1.03
3.05
0.97
1.12
1.83
0.99
0.98
2.00
2.02
f1 (ppm)

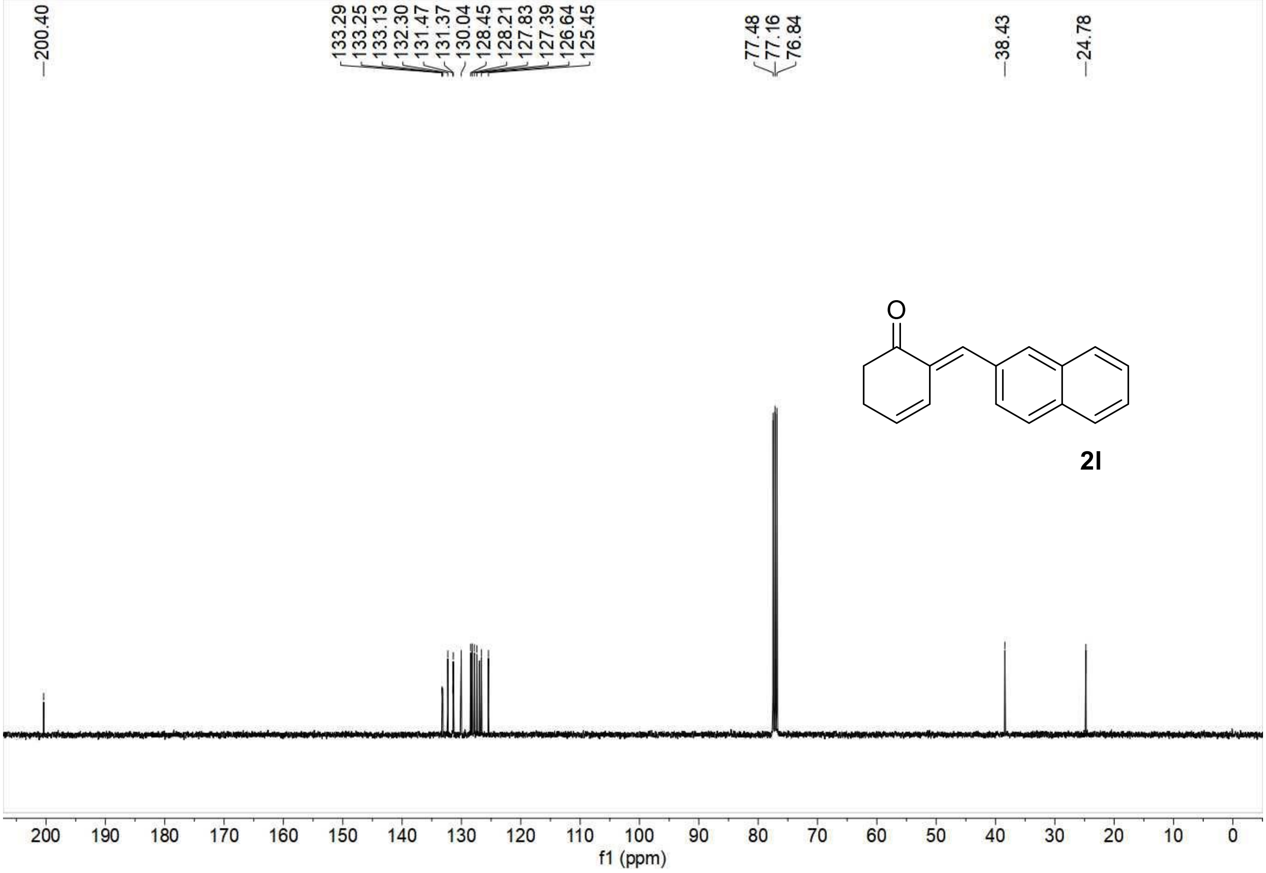
200.40
133.29
133.25
133.13
132.30
131.47
131.37
130.04
128.45
128.21
127.83
127.39
126.64
125.45
77.48
77.16
76.84
38.43
24.78
O
2l
f1 (ppm)

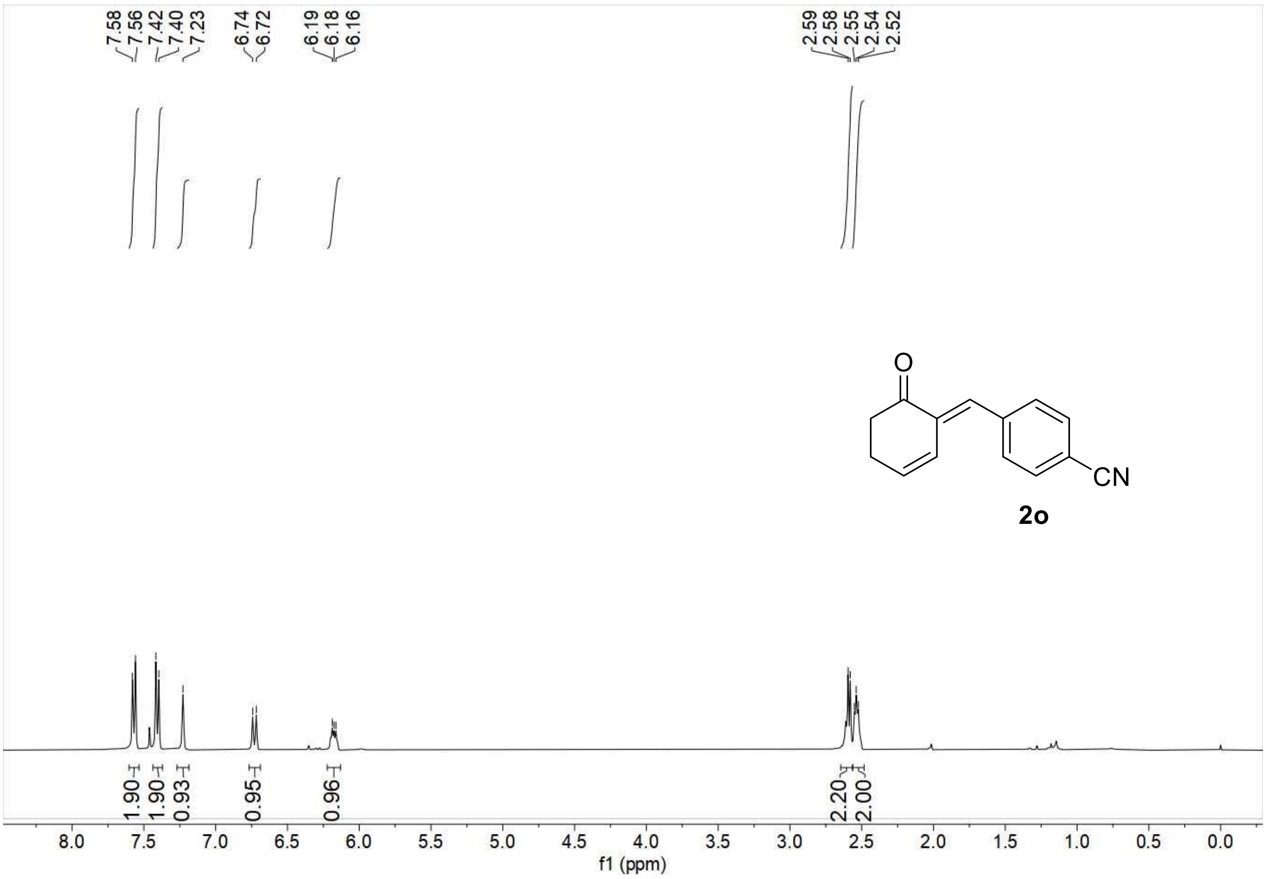
7.58
7.56
7.42
7.40
7.23
6.74
6.72
6.19
6.18
6.16
2.59
2.58
2.55
2.54
2.52
O
CN
2o
1.90
1.90
0.93
0.95
0.96
2.20
2.00
8.0 7.5 7.0 6.5 6.0 5.5 5.0 4.5 4.0 3.5 3.0 2.5 2.0 1.5 1.0 0.5 0.0
f1 (ppm)

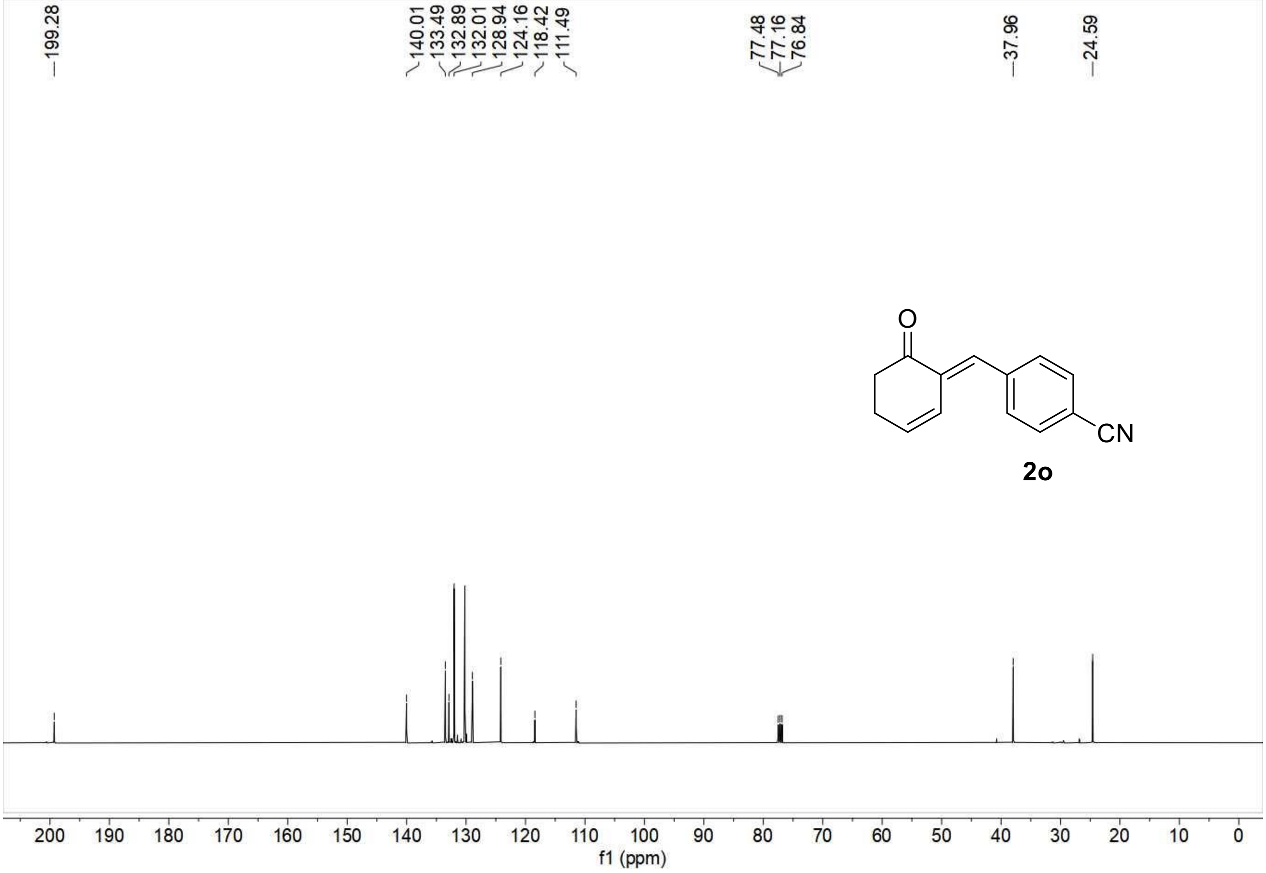
199.28
140.01
133.49
132.89
132.01
128.94
124.16
118.42
111.49
77.48
77.16
76.84
37.96
24.59
O
CN
2o
200 190 180 170 160 150 140 130 120 110 100 90 80 70 60 50 40 30 20 10 0
f1 (ppm)

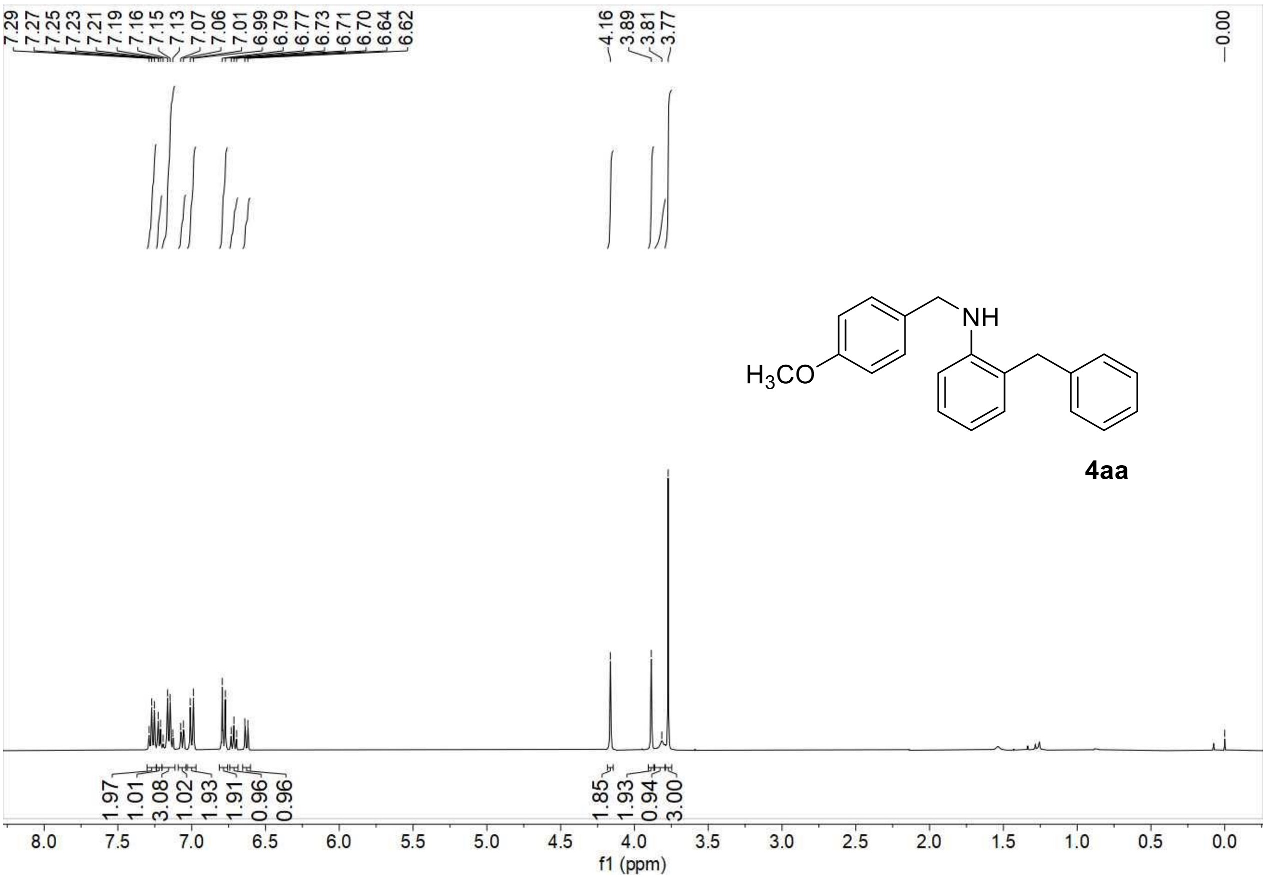
7.29
7.27
7.25
7.23
7.21
7.19
7.16
7.15
7.13
7.07
7.06
7.01
6.99
6.79
6.77
6.73
6.71
6.70
6.64
6.62
4.16
3.89
3.81
3.77
0.00
NH
H3CO
4aa
1.97
1.01
3.08
1.02
1.93
1.91
0.96
0.96
1.85
1.93
0.94
3.00
f1 (ppm)

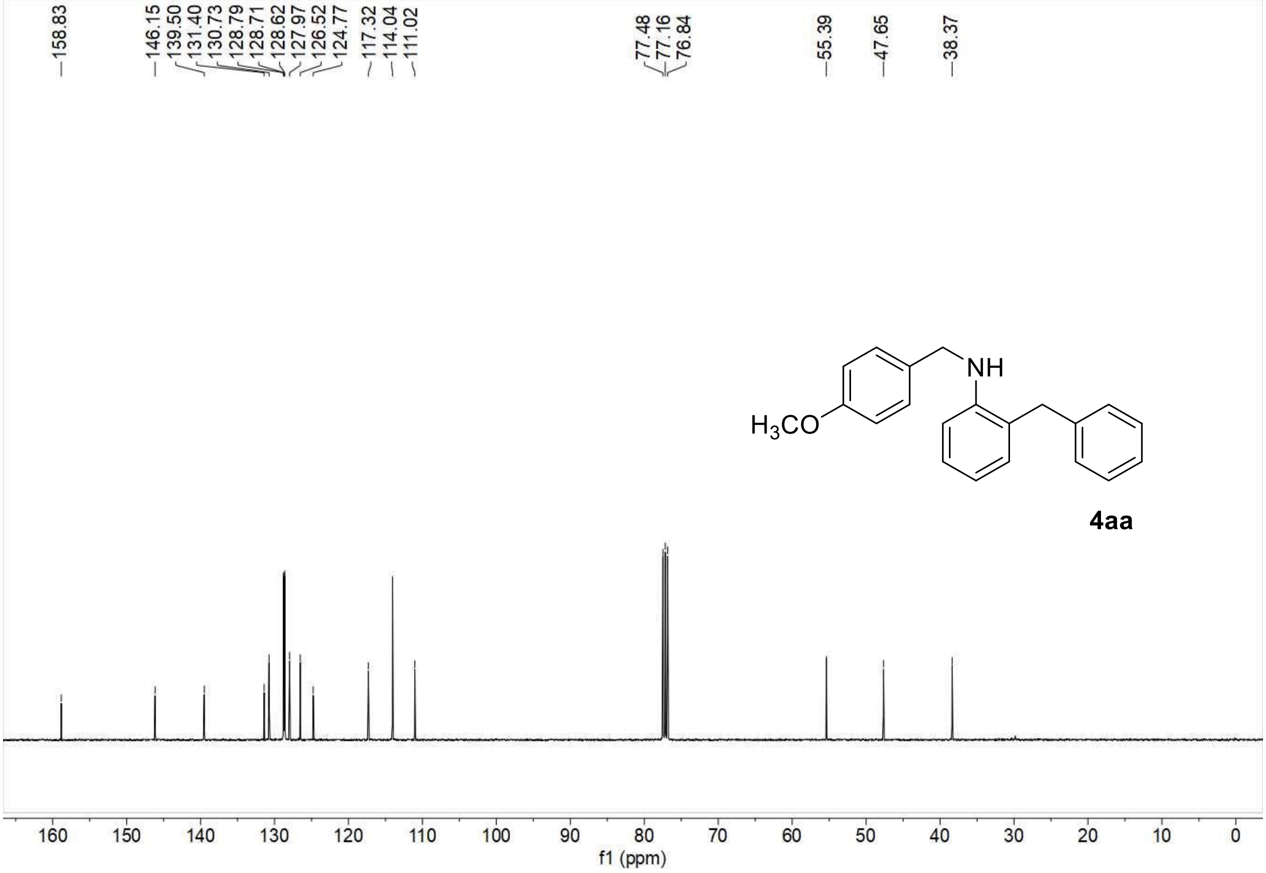
158.83
146.15
139.50
131.40
130.73
128.79
128.71
128.62
127.97
126.52
124.77
117.32
114.04
111.02
77.48
77.16
76.84
55.39
47.65
38.37
NH
H3CO
4aa
f1 (ppm)

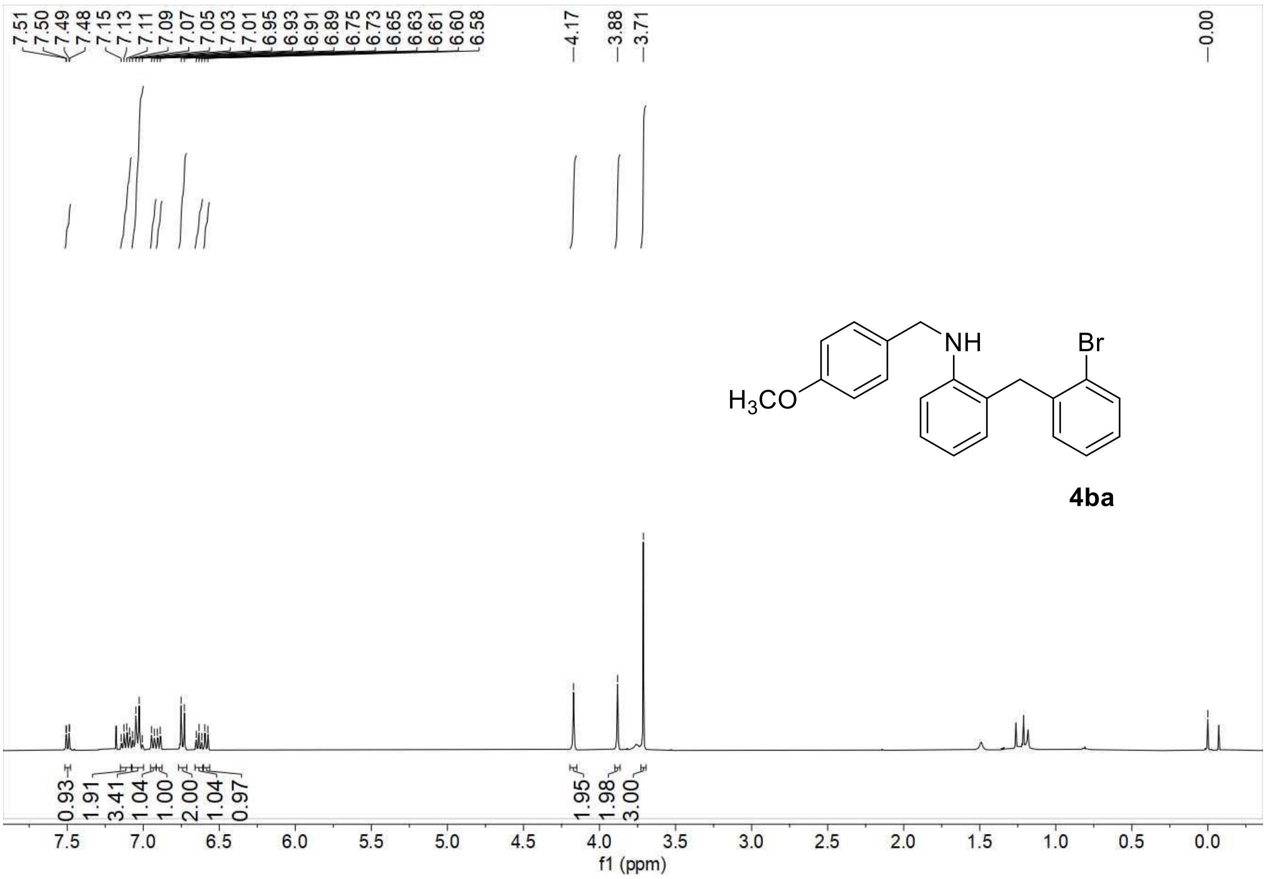
7.51
7.50
7.49
7.48
7.15
7.13
7.11
7.09
7.07
7.05
7.03
7.01
6.95
6.93
6.91
6.89
6.75
6.73
6.65
6.63
6.61
6.60
6.58
4.17
3.88
3.71
0.00
NH
Br
H3CO
4ba
0.93
1.91
3.41
1.04
1.00
2.00
1.04
0.97
1.95
1.98
3.00
f1 (ppm)

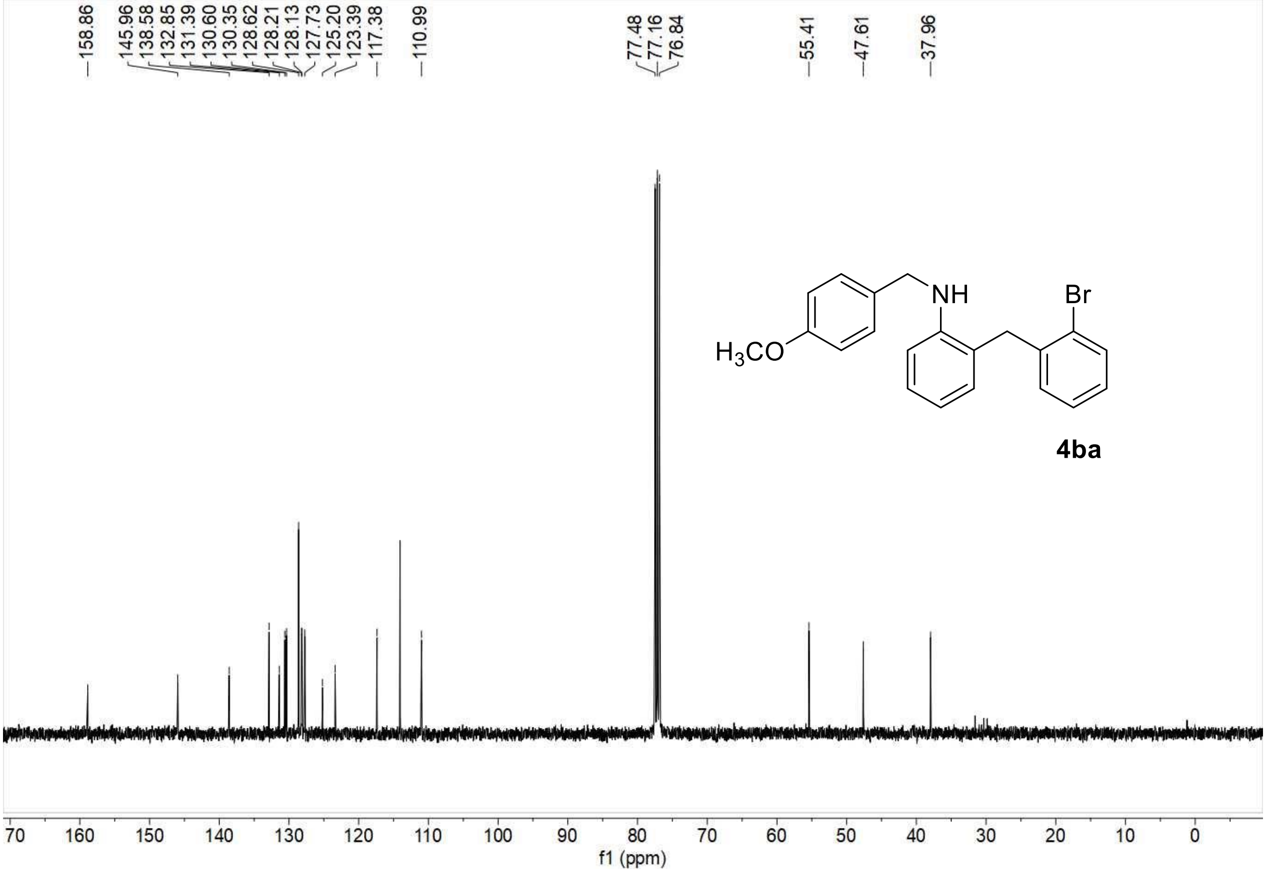
158.86
145.96
138.58
132.85
131.39
130.60
130.35
128.62
128.21
128.13
127.73
125.20
123.39
117.38
110.99
77.48
77.16
76.84
55.41
47.61
37.96
NH
Br
H3CO
4ba
f1 (ppm)

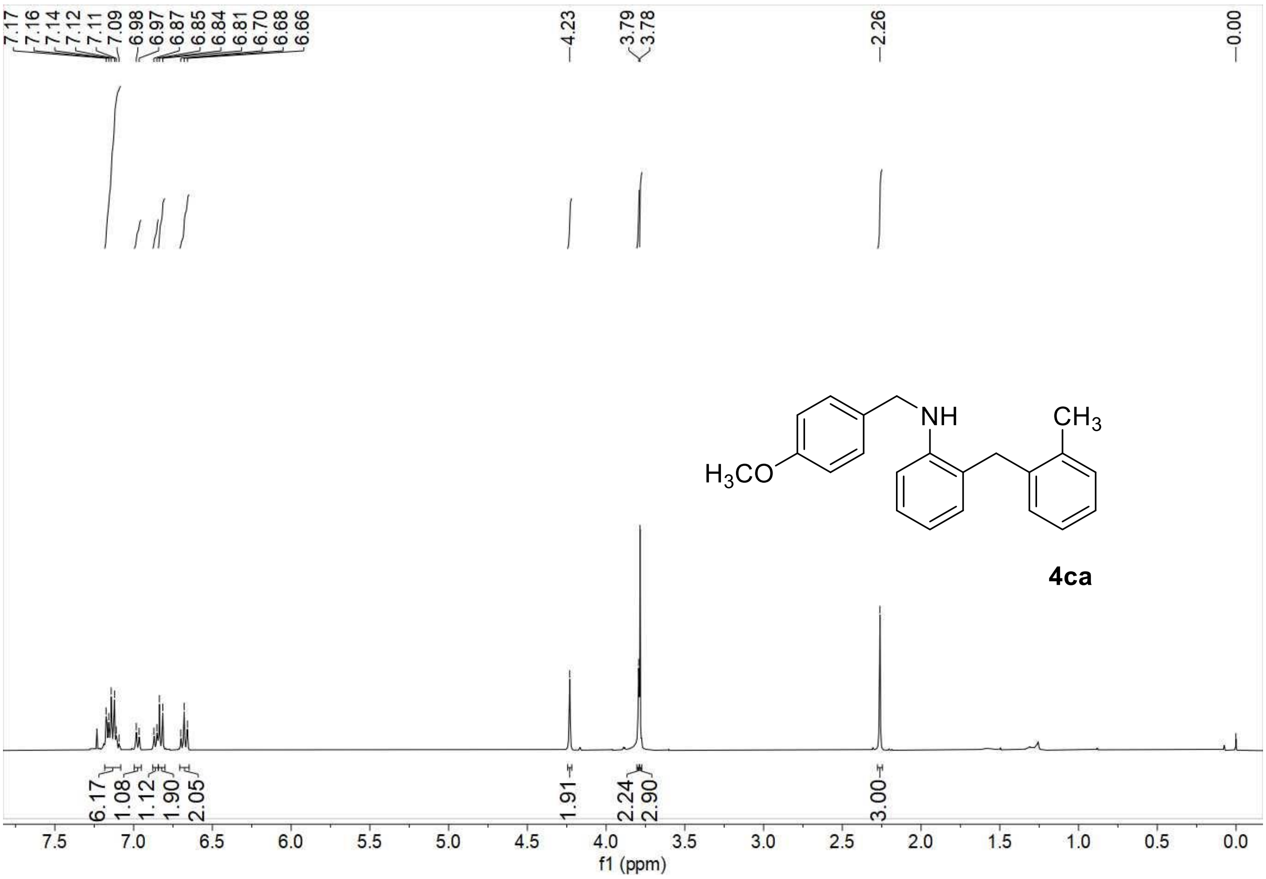
7.17
7.16
7.14
7.12
7.11
7.09
6.98
6.97
6.87
6.85
6.84
6.81
6.70
6.68
6.66
4.23
3.79
3.78
2.26
0.00
6.17
1.08
1.12
1.90
2.05
1.91
2.24
2.90
3.00
NH
CH3
H3CO
4ca
f1 (ppm)

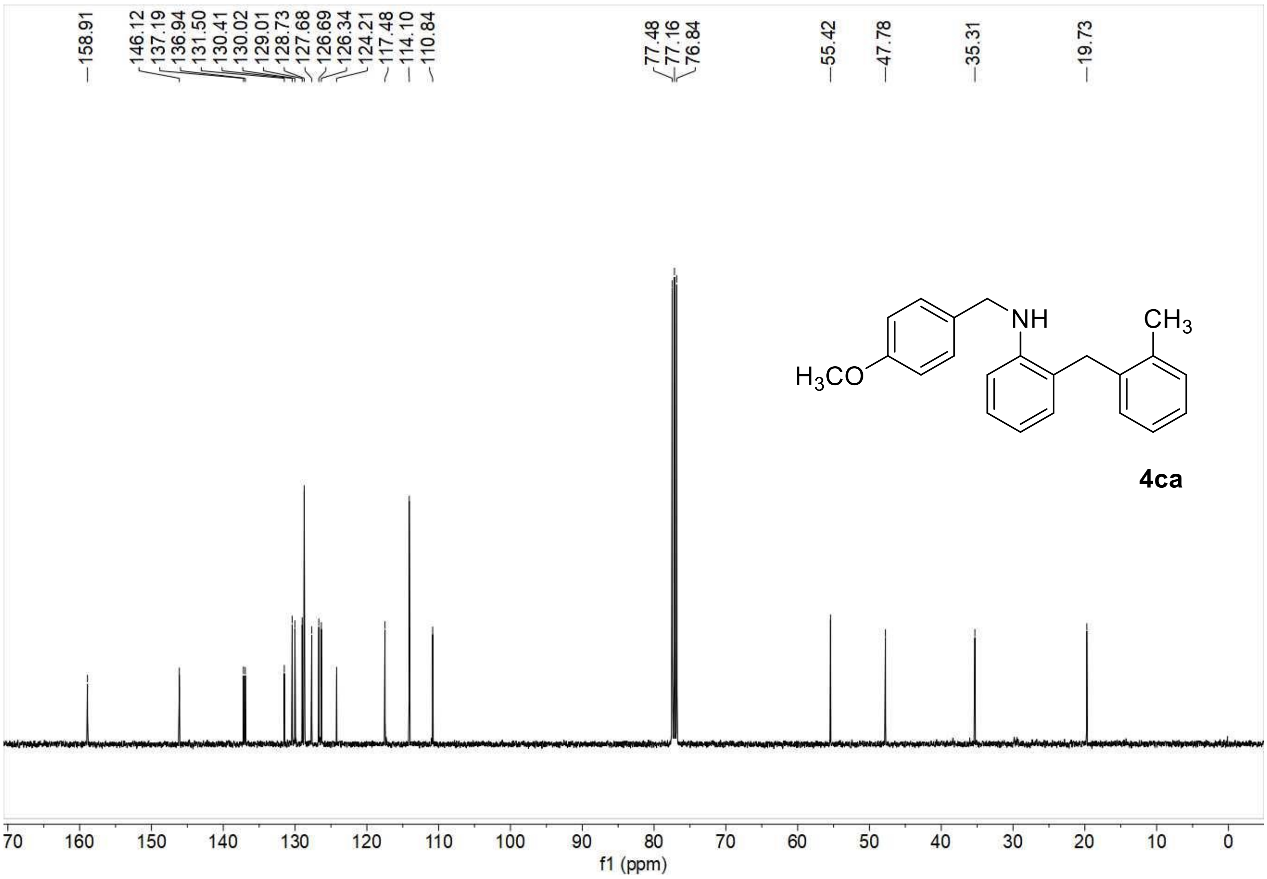
158.91
146.12
137.19
136.94
131.50
130.41
130.02
129.01
128.73
127.68
126.69
126.34
124.21
117.48
114.10
110.84
77.48
77.16
76.84
55.42
47.78
35.31
19.73
NH
CH3
H3CO
4ca
f1 (ppm)

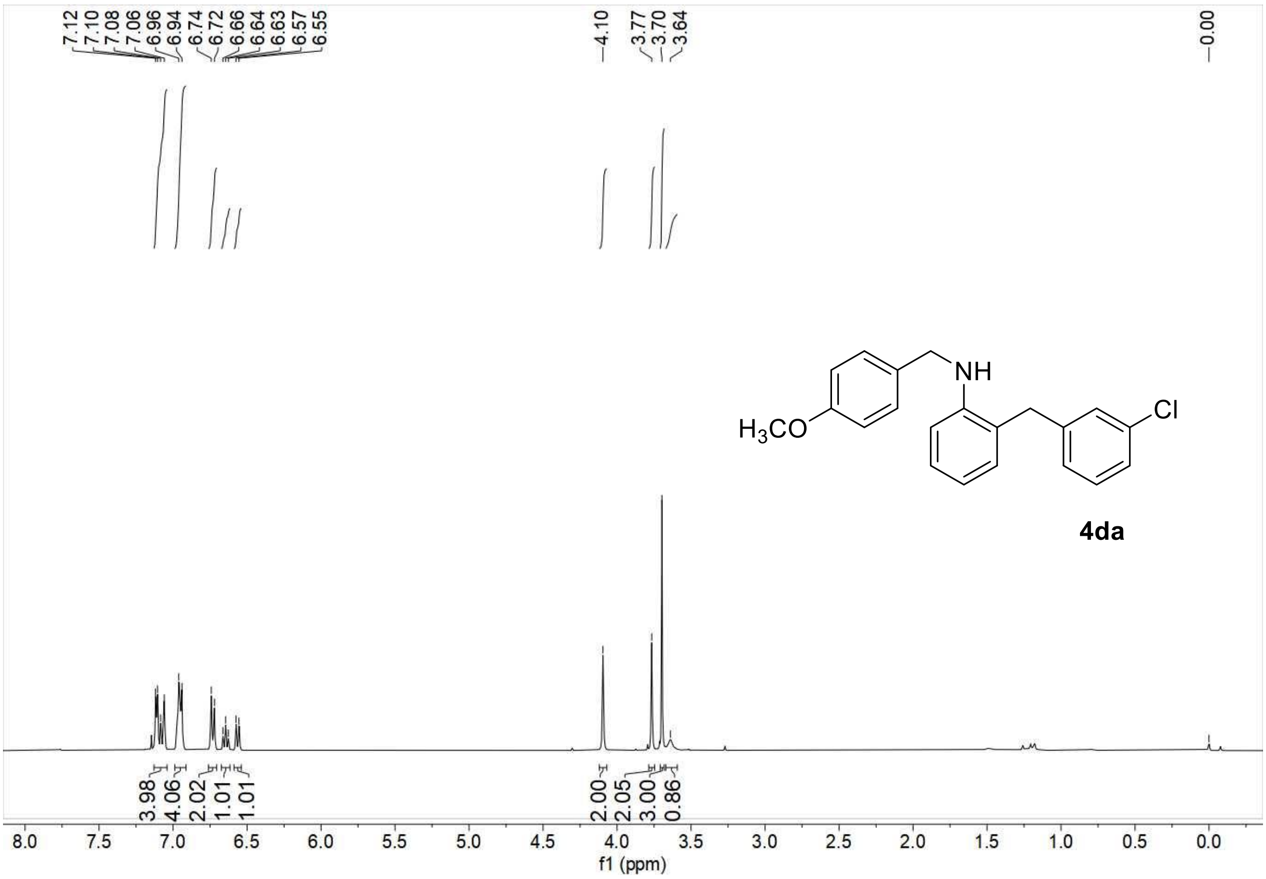

7.12
7.10
7.08
7.06
6.96
6.94
6.74
6.72
6.66
6.64
6.63
6.57
6.55
4.10
3.77
3.70
3.64
0.00
NH
H3CO
Cl
4da
3.98
4.06
2.02
1.01
1.01
2.00
2.05
3.00
0.86
f1 (ppm)

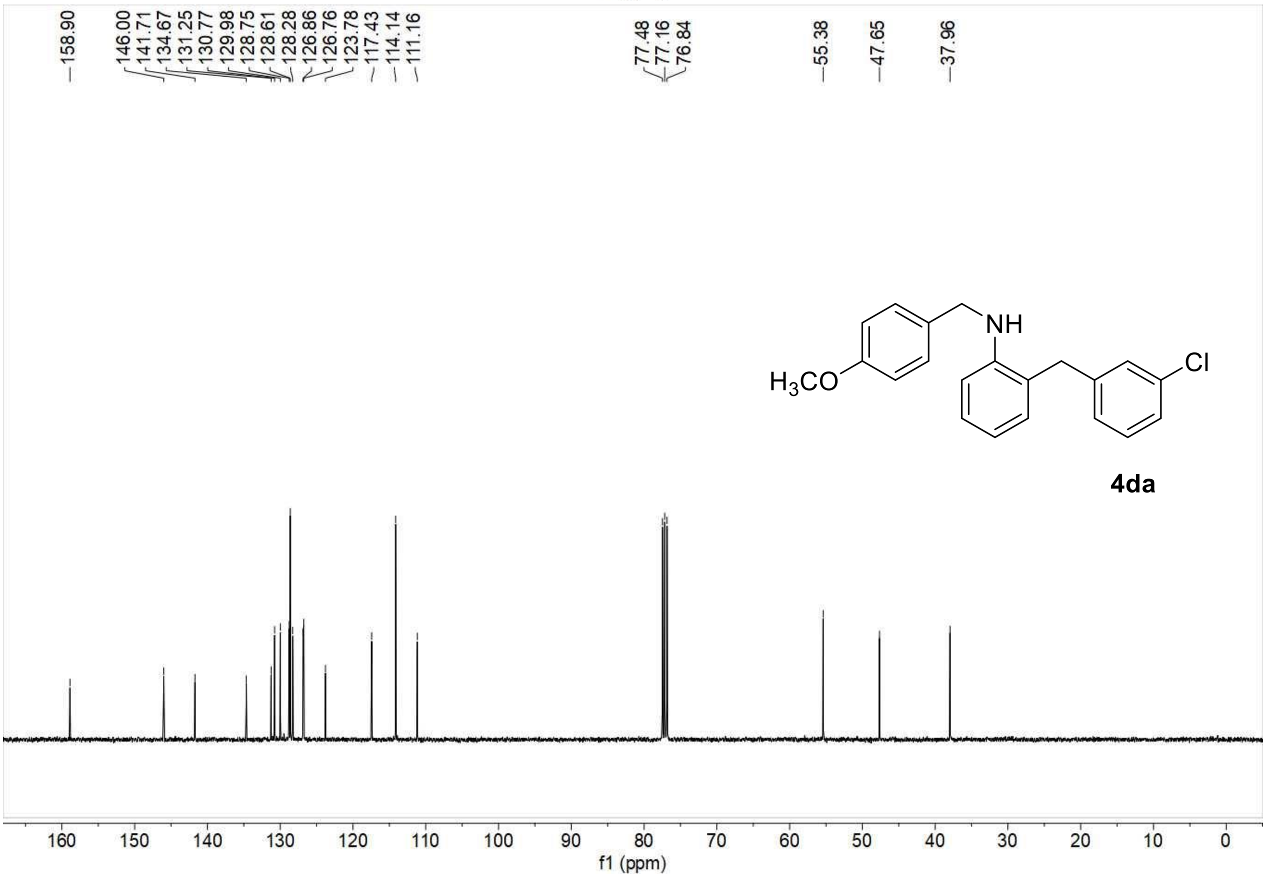

158.90
146.00
141.71
134.67
131.25
130.77
129.98
128.75
128.61
128.28
126.86
126.76
123.78
117.43
114.14
111.16
77.48
77.16
76.84
55.38
47.65
37.96
NH
H3CO
Cl
4da
f1 (ppm)

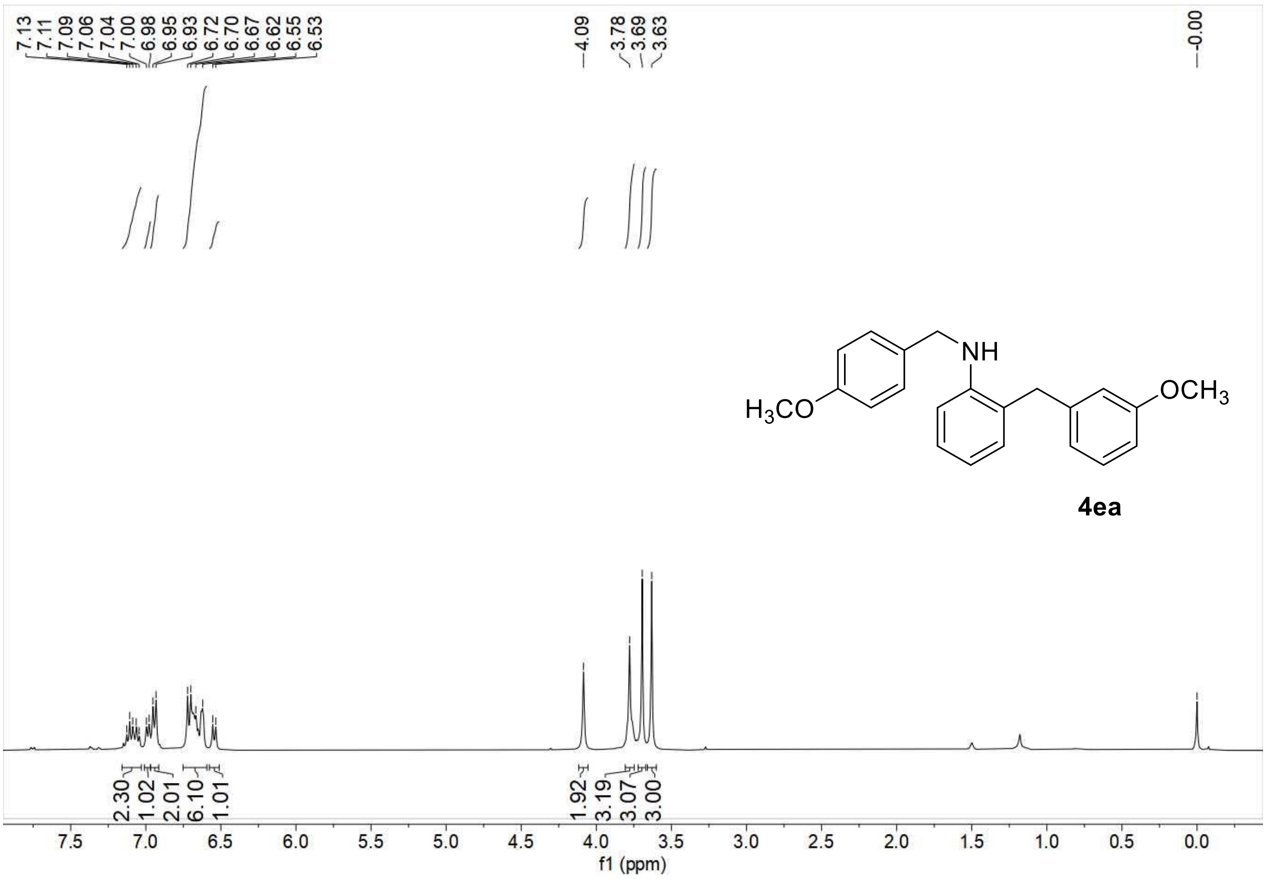
7.13
7.11
7.09
7.06
7.04
7.00
6.98
6.95
6.93
6.72
6.70
6.67
6.62
6.55
6.53
4.09
3.78
3.69
3.63
-0.00
NH
H_3CO
OCH_3
4ea
2.30
1.02
2.01
6.10
1.01
1.92
3.19
3.07
3.00
7.5 7.0 6.5 6.0 5.5 5.0 4.5 4.0 3.5 3.0 2.5 2.0 1.5 1.0 0.5 0.0
f1 (ppm)

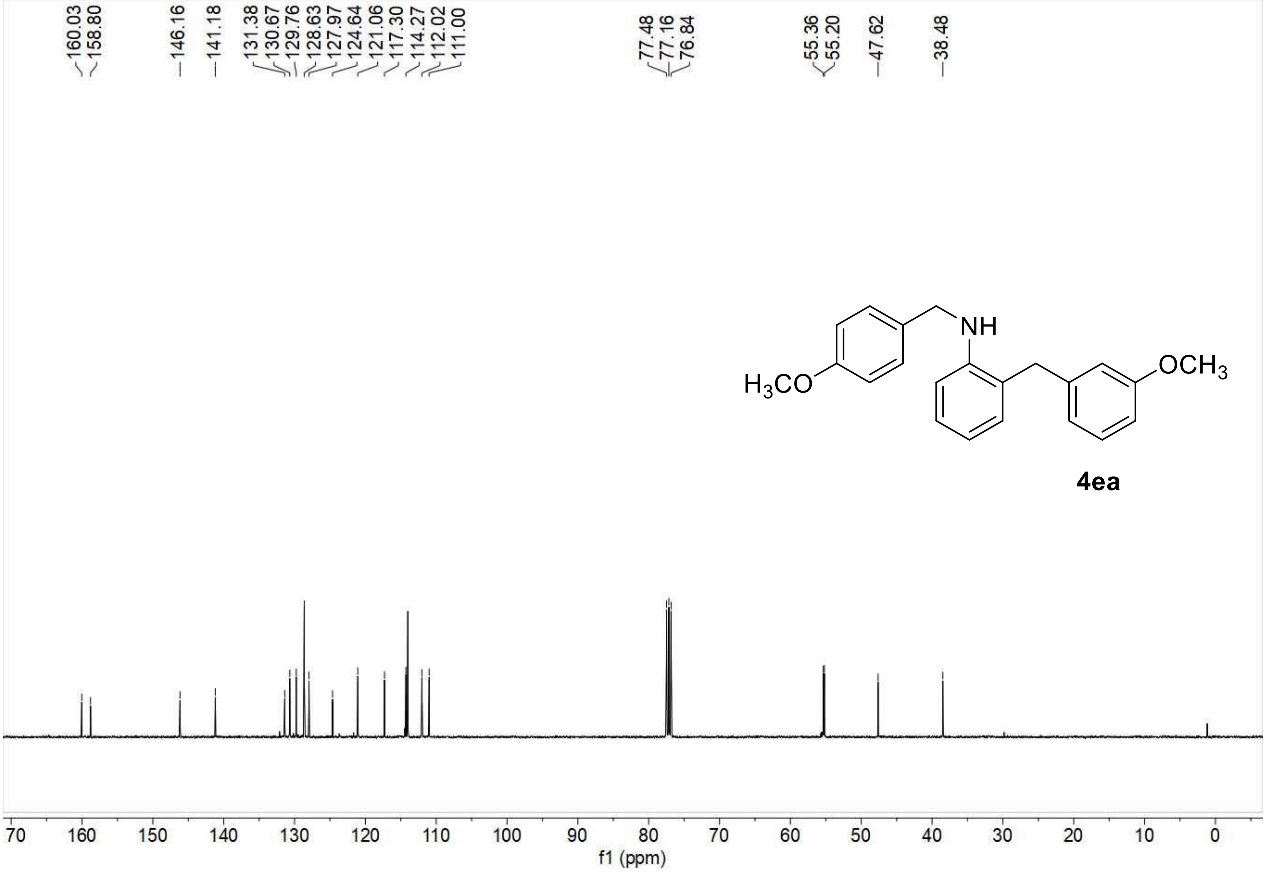
160.03
158.80
146.16
141.18
131.38
130.67
129.76
128.63
127.97
124.64
121.06
117.30
114.27
112.02
111.00
77.48
77.16
76.84
55.36
55.20
47.62
38.48
NH
H_3CO
OCH_3
4ea
70 160 150 140 130 120 110 100 90 80 70 60 50 40 30 20 10 0
f1 (ppm)

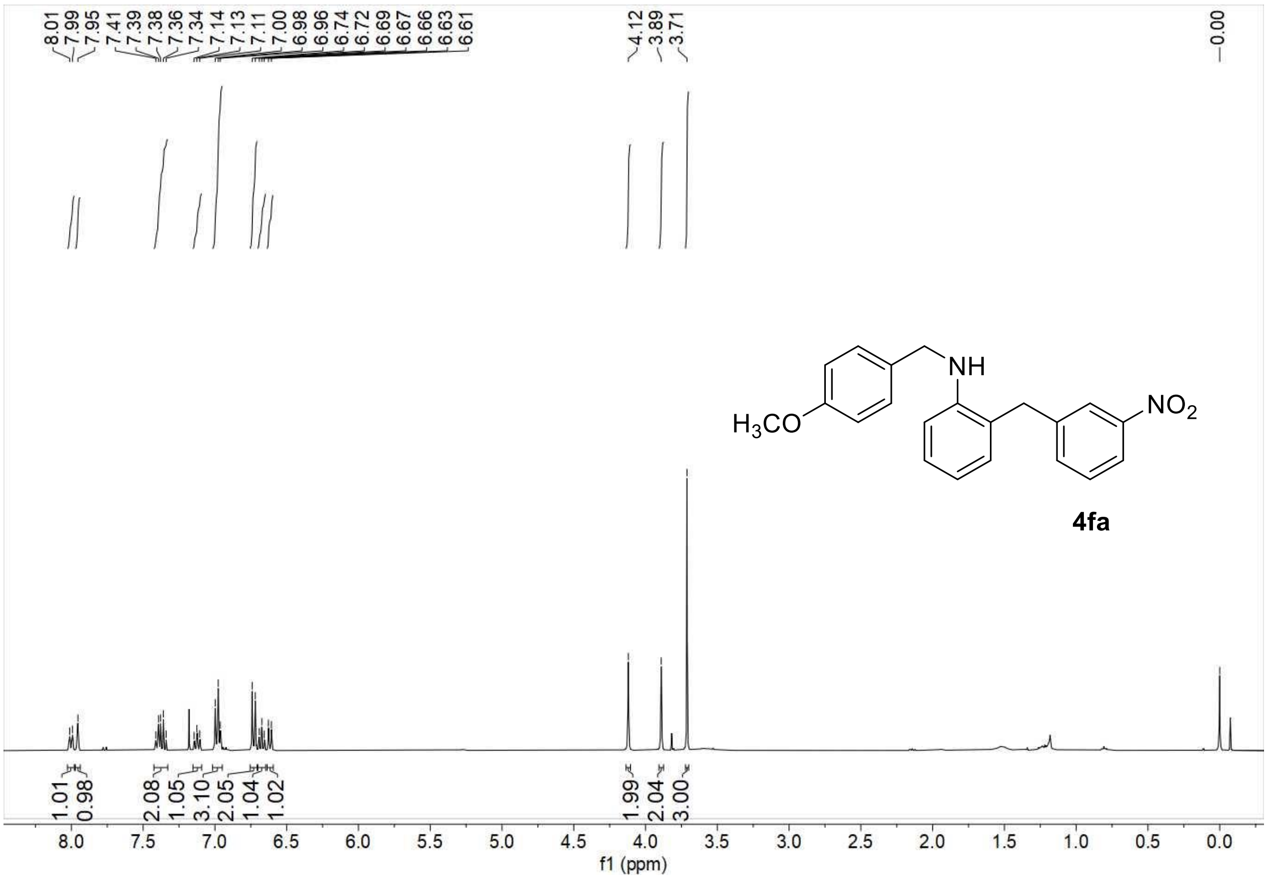
8.01
7.99
7.95
7.41
7.39
7.38
7.36
7.34
7.14
7.13
7.11
7.00
6.98
6.96
6.74
6.72
6.69
6.67
6.66
6.63
6.61
4.12
3.89
3.71
0.00
NH
NO2
H3CO
4fa
1.01
0.98
2.08
1.05
3.10
2.05
1.04
1.02
1.99
2.04
3.00
f1 (ppm)

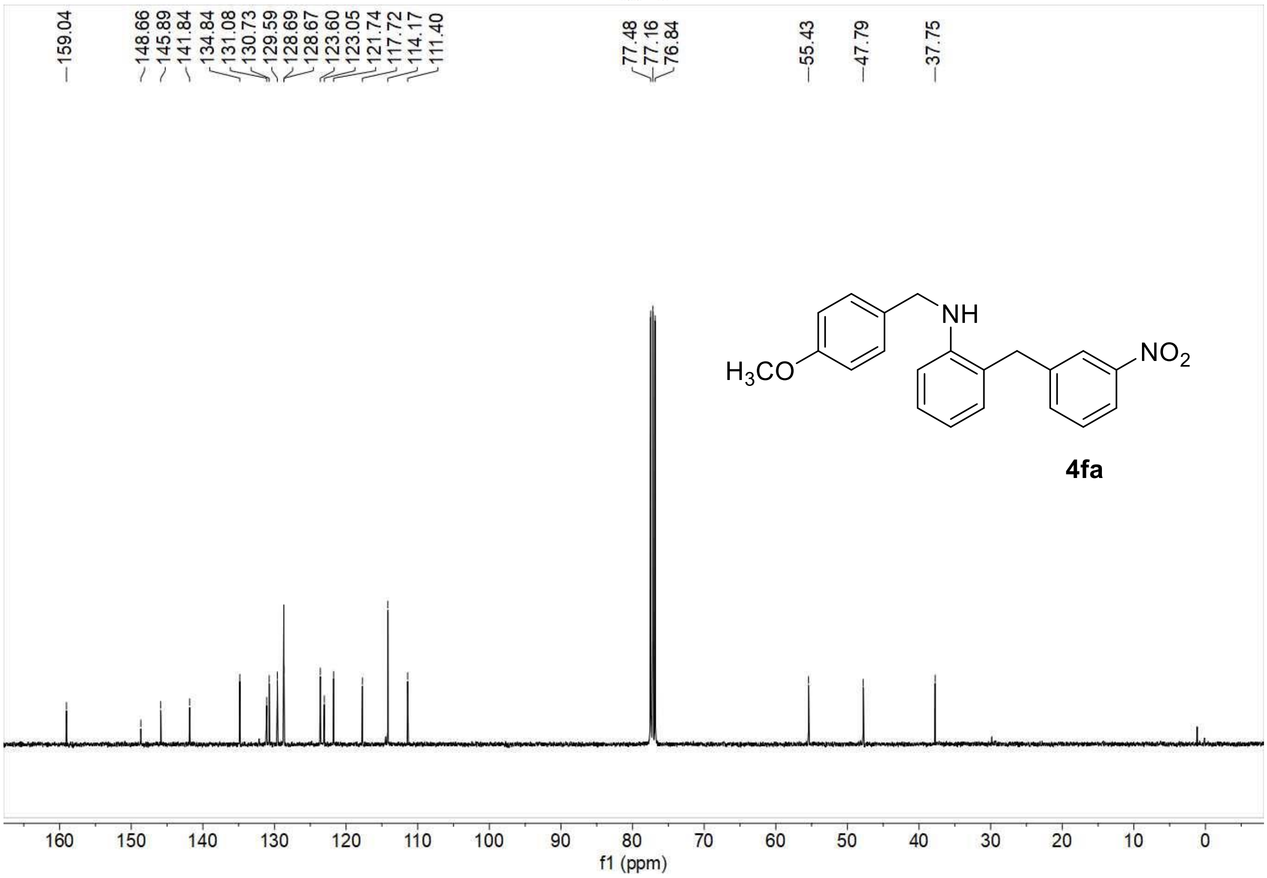
159.04
148.66
145.89
141.84
134.84
131.08
130.73
129.59
128.69
128.67
123.60
123.05
121.74
117.72
114.17
111.40
77.48
77.16
76.84
55.43
47.79
37.75
NH
NO2
H3CO
4fa
f1 (ppm)

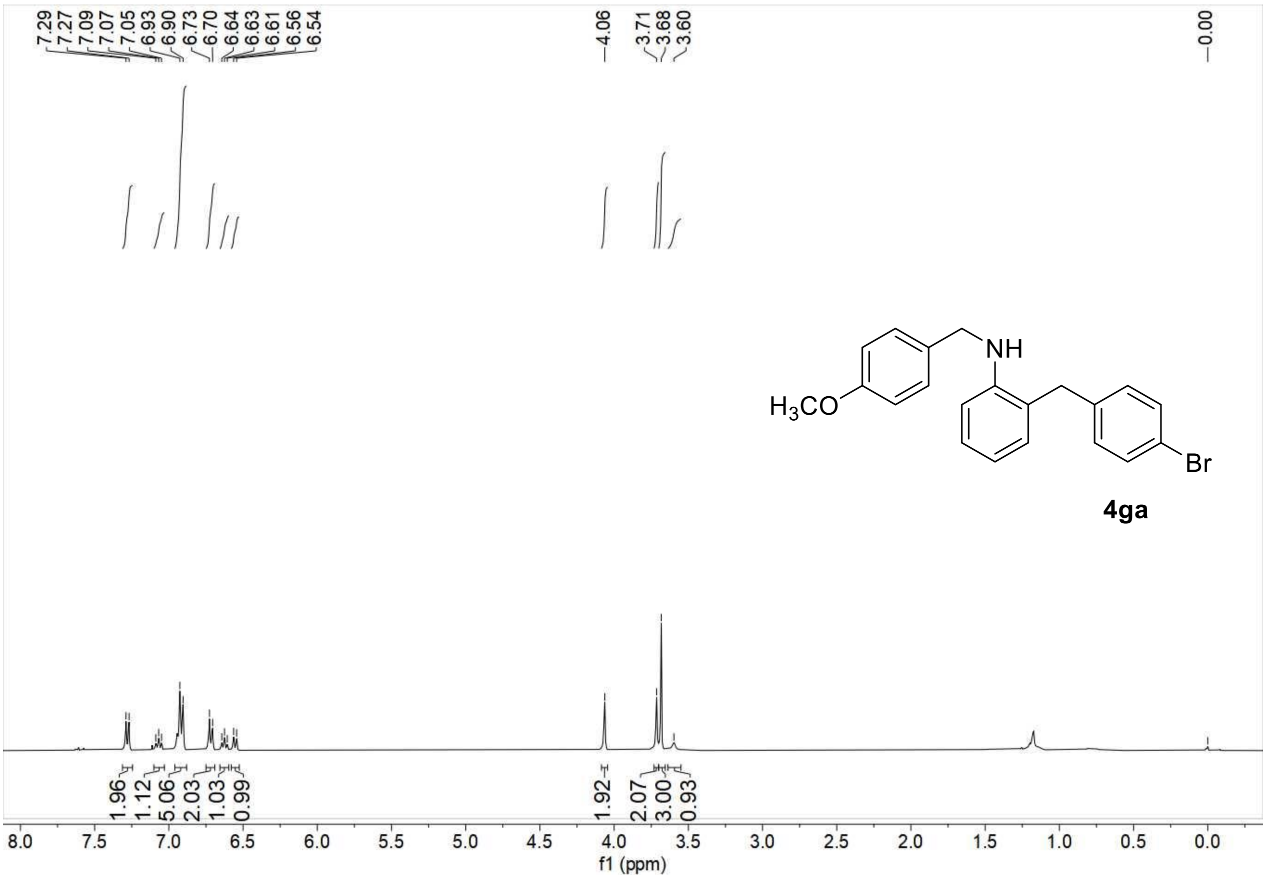
7.29 7.27 7.09 7.07 7.05 6.93 6.90 6.73 6.70 6.64 6.63 6.61 6.56 6.54
4.06
3.71 3.68 3.60
0.00
NH
H3CO
Br
4ga
1.96 1.12 5.06 2.03 1.03 0.99
1.92
2.07 3.00 0.93
8.0 7.5 7.0 6.5 6.0 5.5 5.0 4.5 4.0 3.5 3.0 2.5 2.0 1.5 1.0 0.5 0.0
f1 (ppm)

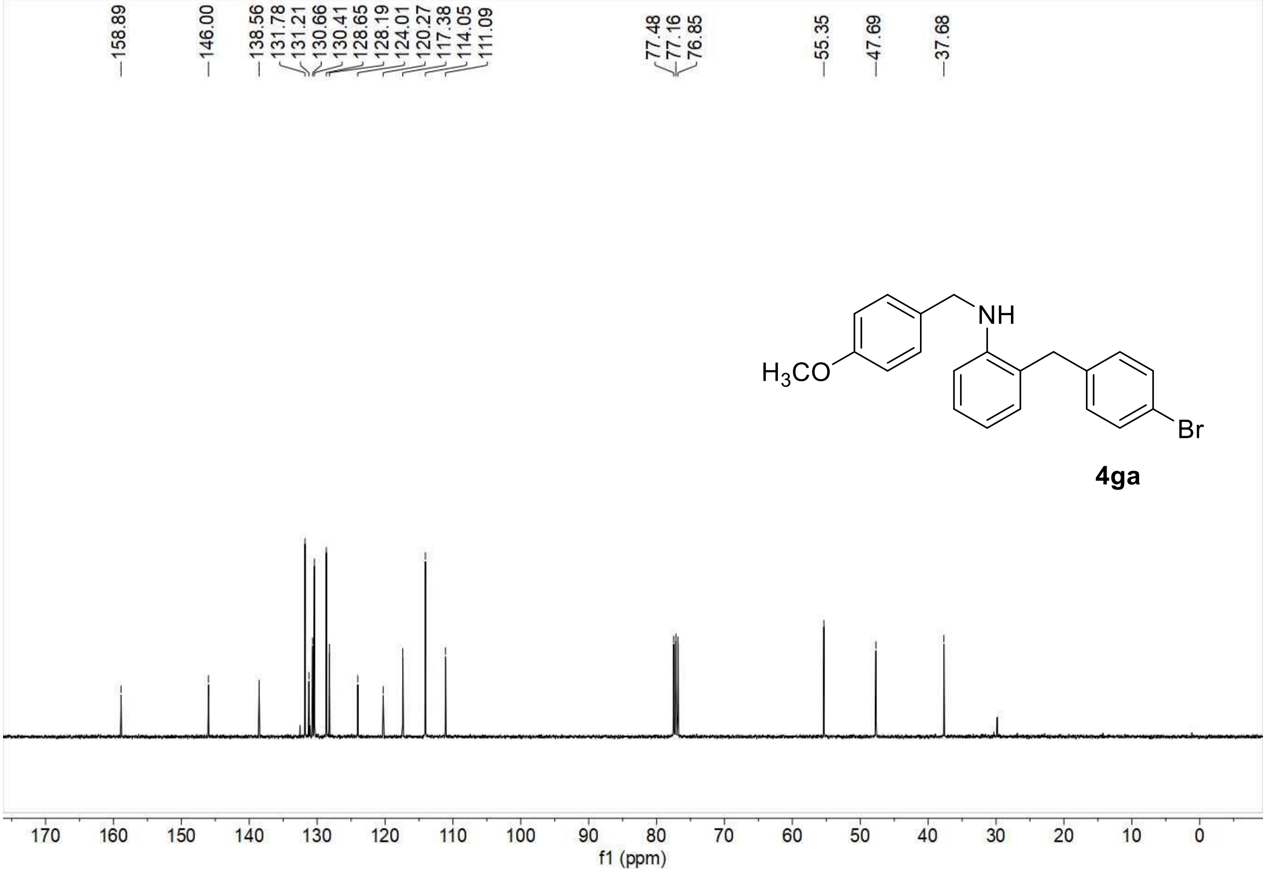
158.89
146.00
138.56 131.78 131.21 130.66 130.41 128.65 128.19 124.01 120.27 117.38 114.05 111.09
77.48 77.16 76.85
55.35
47.69
37.68
NH
H3CO
Br
4ga
170 160 150 140 130 120 110 100 90 80 70 60 50 40 30 20 10 0
f1 (ppm)

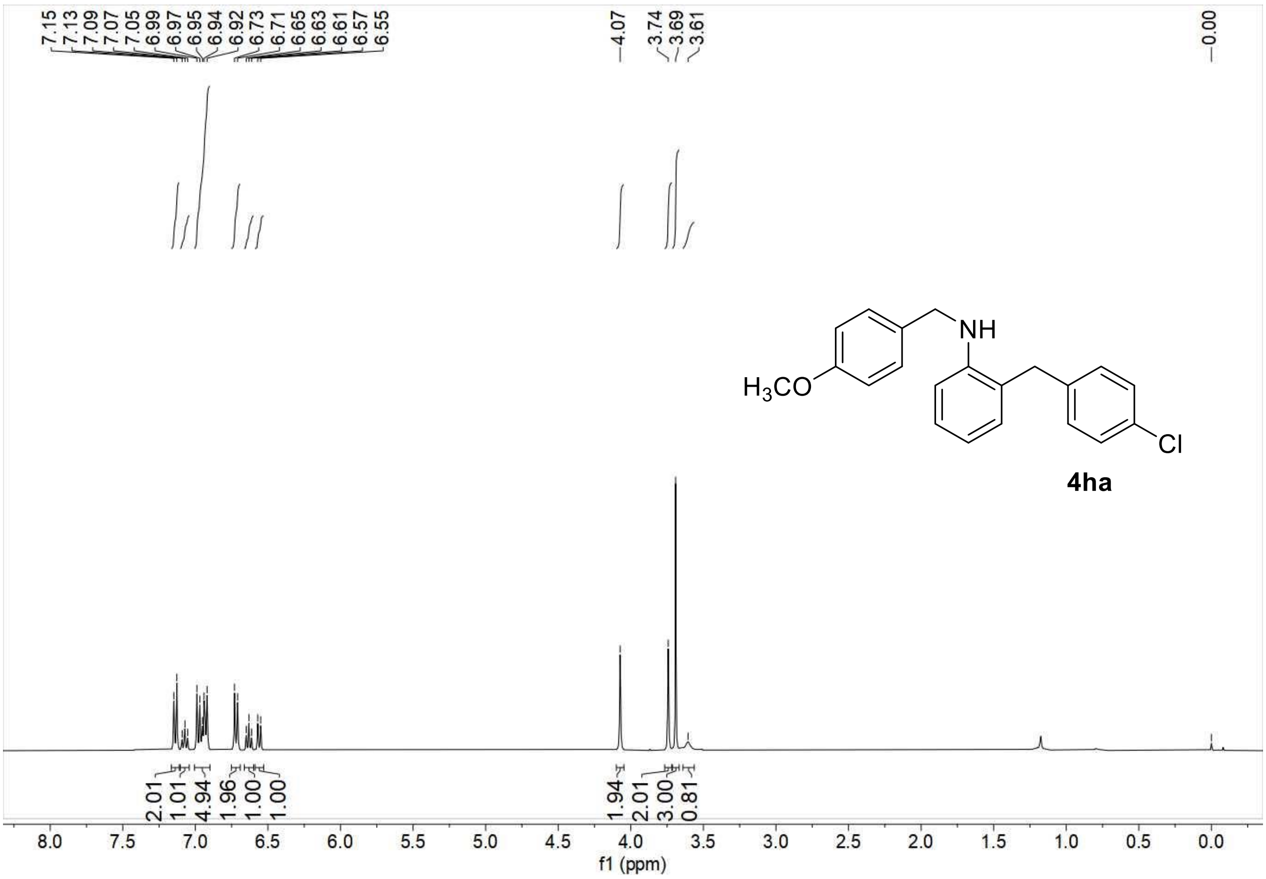
7.15
7.13
7.09
7.07
7.05
6.99
6.97
6.95
6.94
6.92
6.73
6.71
6.65
6.63
6.61
6.57
6.55
4.07
3.74
3.69
3.61
0.00
NH
H_3CO
Cl
4ha
2.01
1.01
4.94
1.96
1.00
1.00
1.94
2.01
3.00
0.81
f1 (ppm)

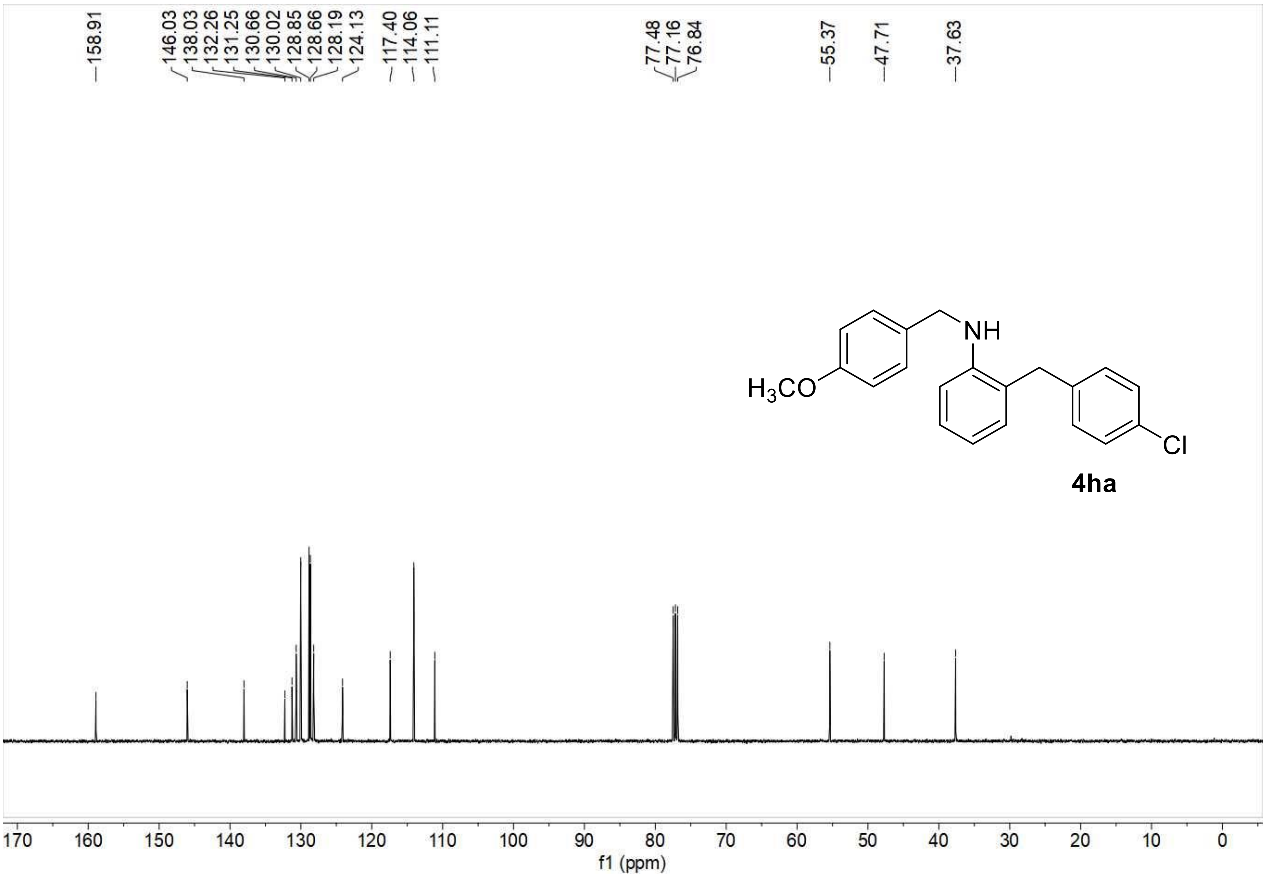
158.91
146.03
138.03
132.26
131.25
130.66
130.02
128.85
128.66
128.19
124.13
117.40
114.06
111.11
77.48
77.16
76.84
55.37
47.71
37.63
NH
H_3CO
Cl
4ha
f1 (ppm)

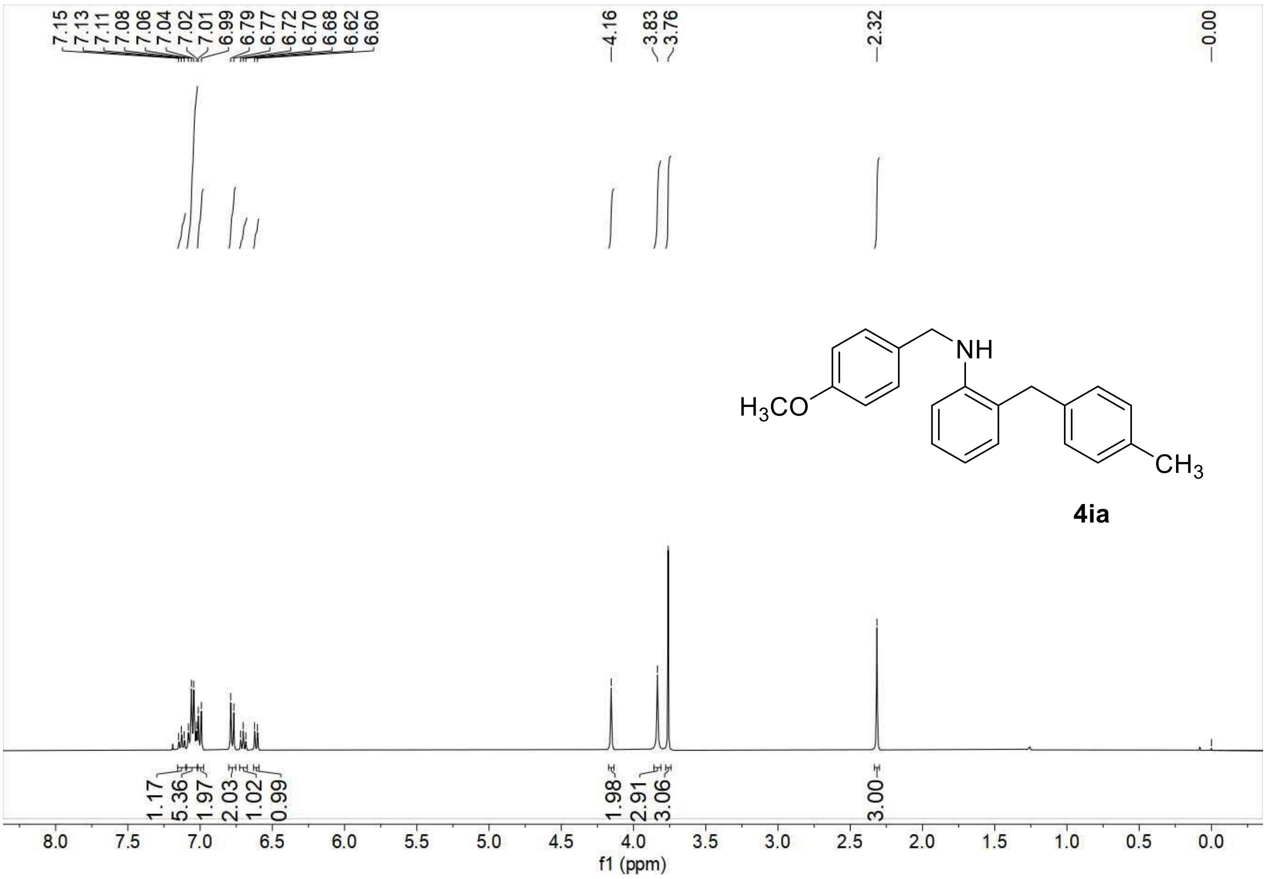

7.15 7.13 7.11 7.08 7.06 7.04 7.02 7.01 6.99 6.79 6.77 6.72 6.70 6.68 6.62 6.60
4.16
3.83 3.76
2.32
0.00
NH
H_3CO
CH_3
1.17 5.36 1.97 2.03 1.02 0.99
1.98 2.91 3.06
3.00
8.0 7.5 7.0 6.5 6.0 5.5 5.0 4.5 4.0 3.5 3.0 2.5 2.0 1.5 1.0 0.5 0.0
f1 (ppm)

4ia

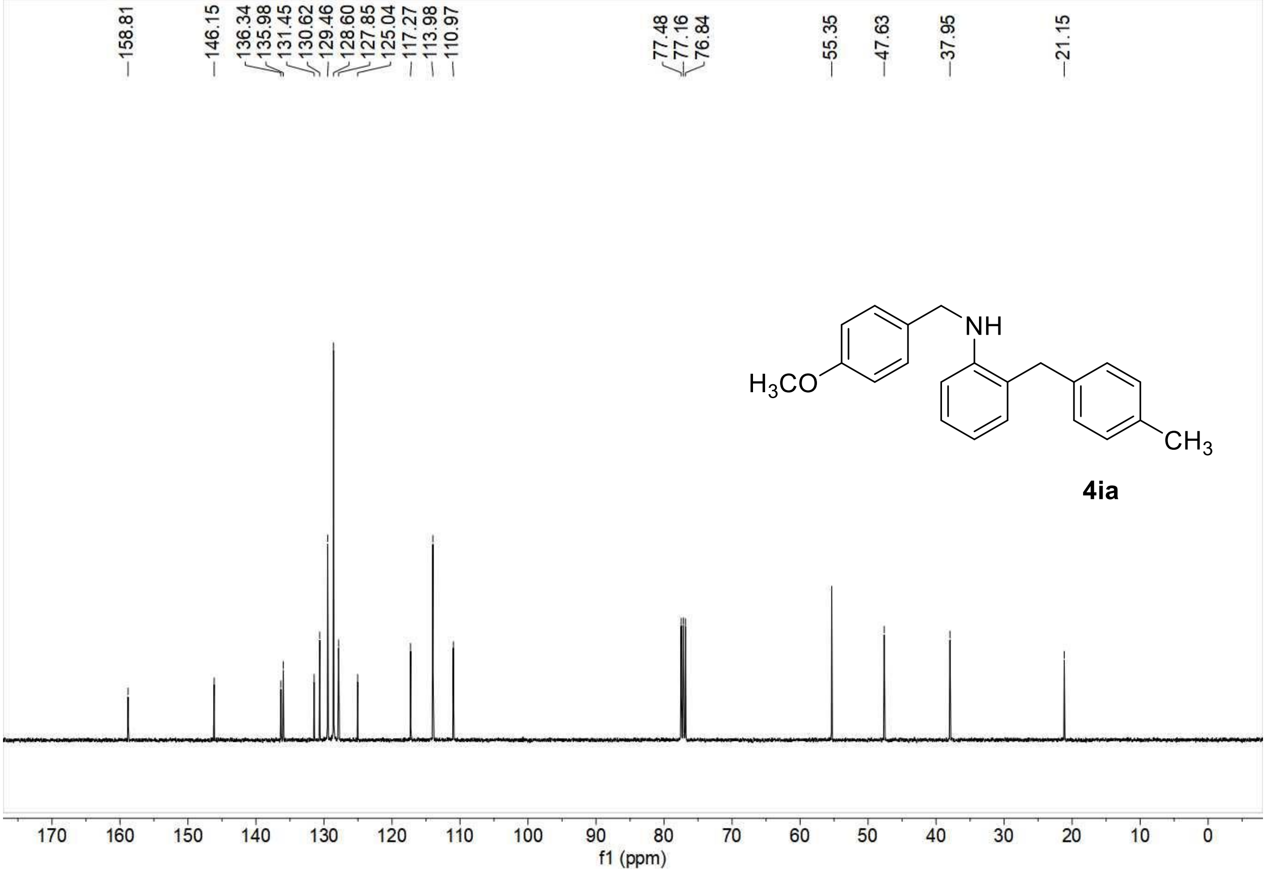

158.81
146.15
136.34 135.98 131.45 130.62 129.46 128.60 127.85 125.04 117.27 113.98 110.97
77.48 77.16 76.84
55.35
47.63
37.95
21.15
NH
H_3CO
CH_3
170 160 150 140 130 120 110 100 90 80 70 60 50 40 30 20 10 0
f1 (ppm)

4ia

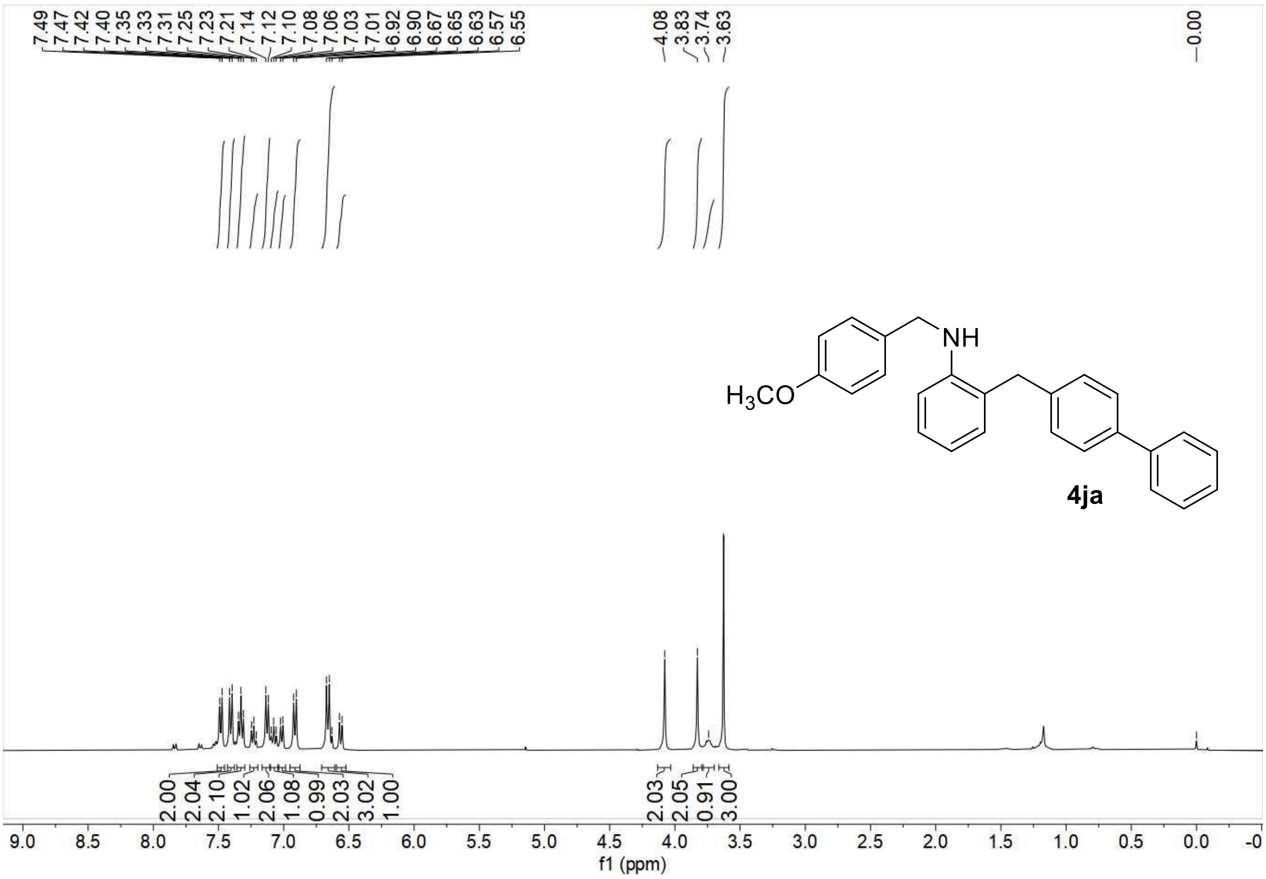

7.49 7.47 7.42 7.40 7.35 7.33 7.31 7.25 7.23 7.21 7.14 7.12 7.10 7.08 7.06 7.03 7.01 6.92 6.90 6.67 6.65 6.63 6.57 6.55
4.08 3.83 3.74 3.63
0.00
NH
H_3CO
4ja
2.00 2.04 2.10 1.02 2.06 1.08 0.99 2.03 3.02 1.00
2.03 2.05 0.91 3.00
f1 (ppm)

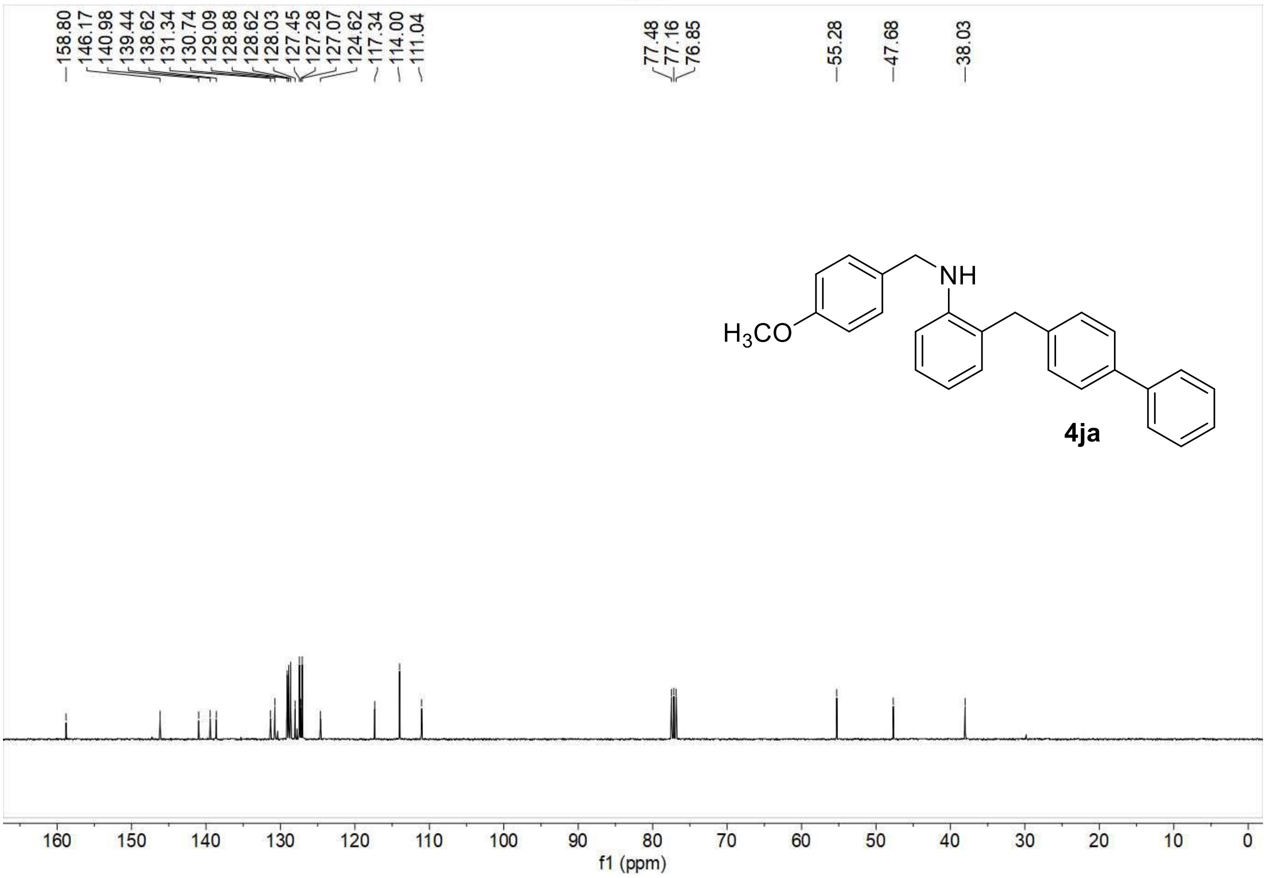

158.80 146.17 140.98 139.44 138.62 131.34 130.74 129.09 128.88 128.62 128.03 127.45 127.28 127.07 124.62 117.34 114.00 111.04
77.48 77.16 76.85
55.28
47.68
38.03
NH
H_3CO
4ja
f1 (ppm)

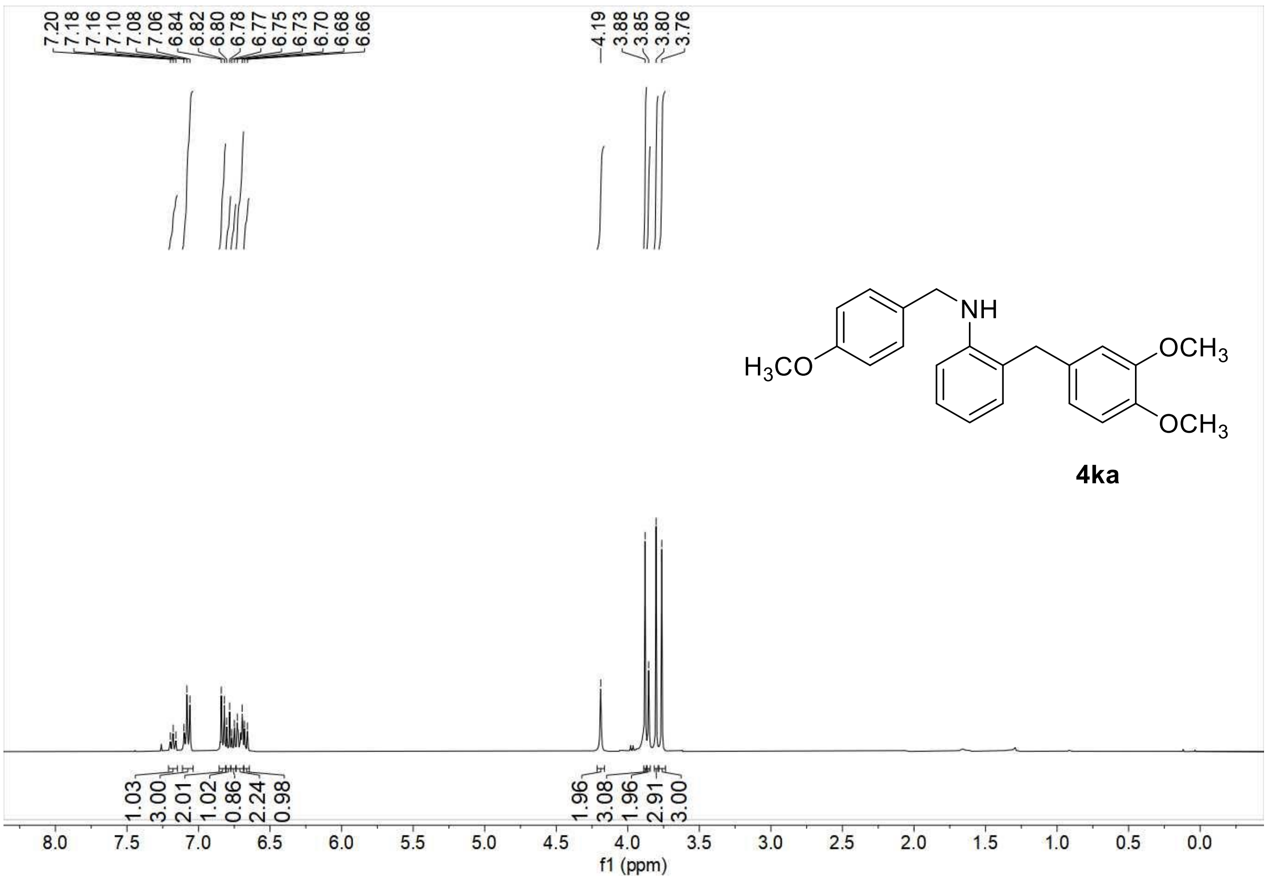

NH
H_3CO
OCH_3
OCH_3
4ka
f1 (ppm)

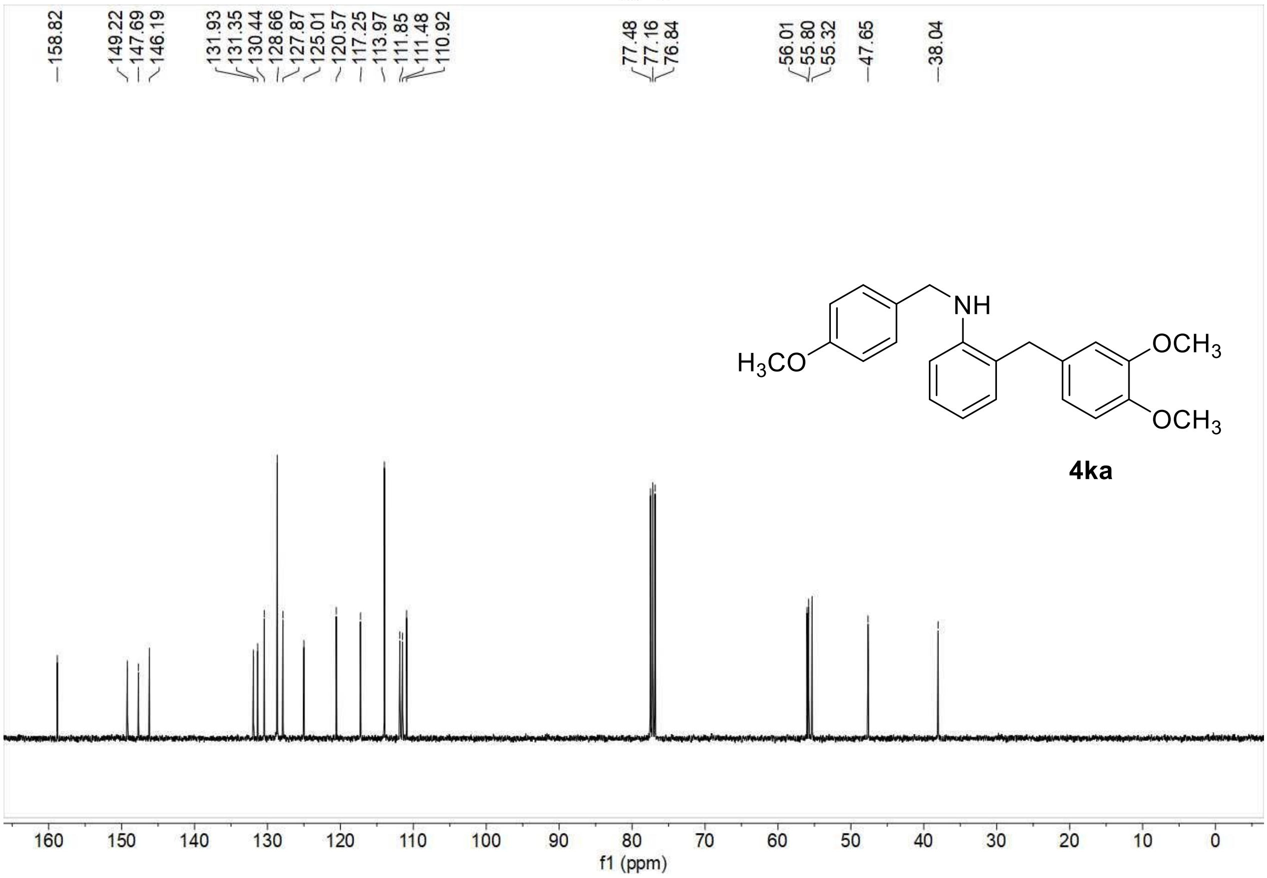

NH
H_3CO
OCH_3
OCH_3
4ka
f1 (ppm)

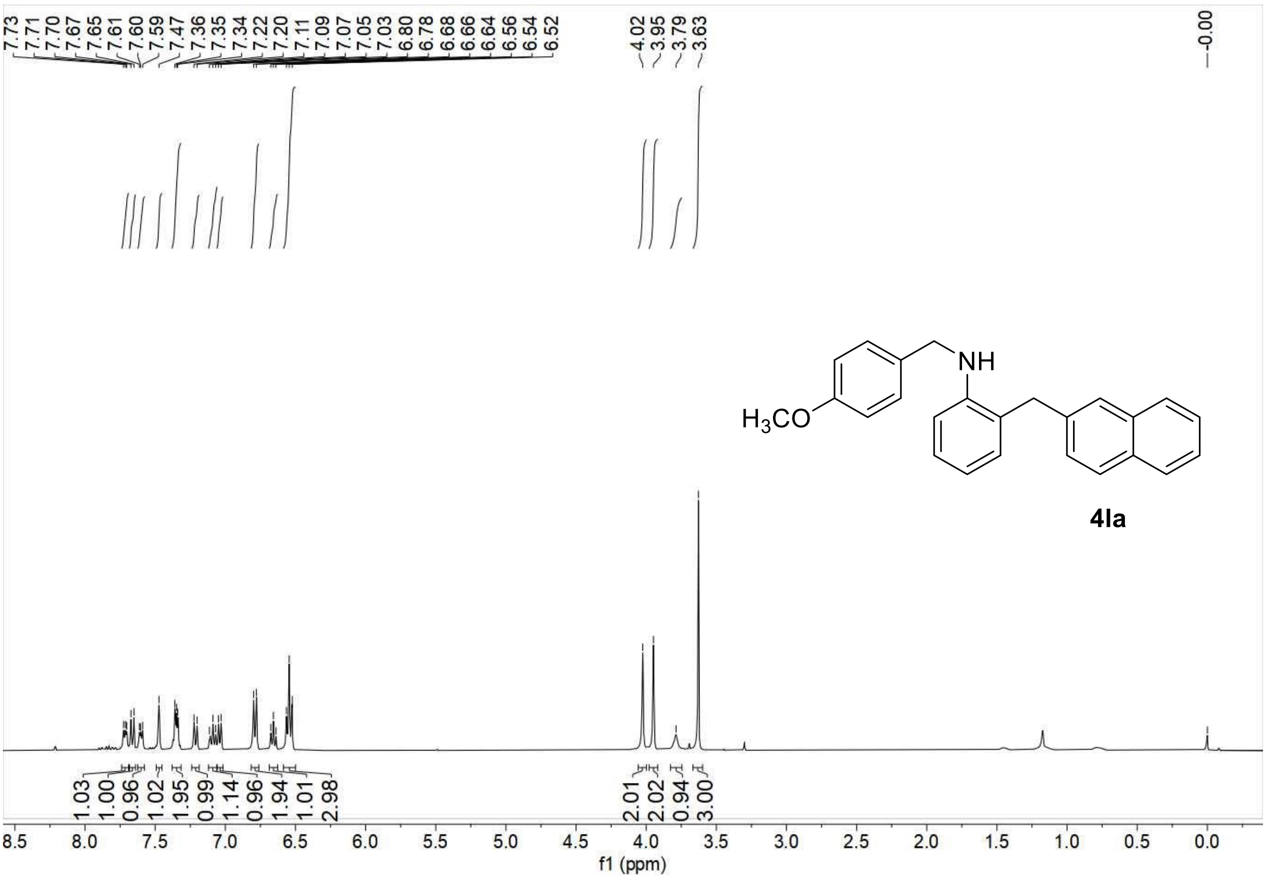
7.73
7.71
7.70
7.67
7.65
7.61
7.60
7.59
7.47
7.36
7.35
7.34
7.22
7.20
7.11
7.09
7.07
7.05
7.03
6.80
6.78
6.68
6.66
6.64
6.56
6.54
6.52
4.02
3.95
3.79
3.63
-0.00
NH
H_3CO
4la
1.03
1.00
0.96
1.02
1.95
0.99
1.14
0.96
1.94
1.01
2.98
2.01
2.02
0.94
3.00
8.5 8.0 7.5 7.0 6.5 6.0 5.5 5.0 4.5 4.0 3.5 3.0 2.5 2.0 1.5 1.0 0.5 0.0
f1 (ppm)

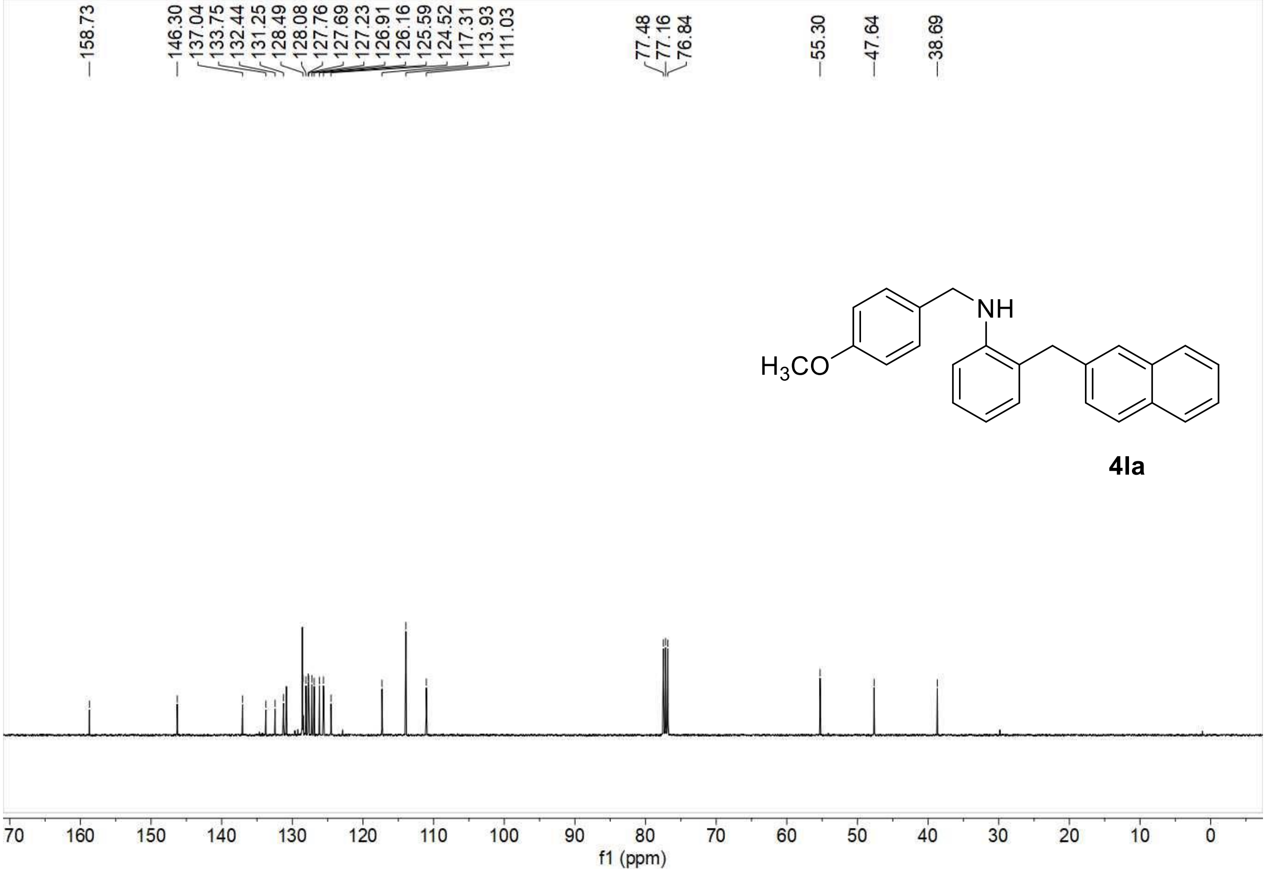
158.73
146.30
137.04
133.75
132.44
131.25
128.49
128.08
127.76
127.69
127.23
126.91
126.16
125.59
124.52
117.31
113.93
111.03
77.48
77.16
76.84
55.30
47.64
38.69
NH
H_3CO
4la
170 160 150 140 130 120 110 100 90 80 70 60 50 40 30 20 10 0
f1 (ppm)

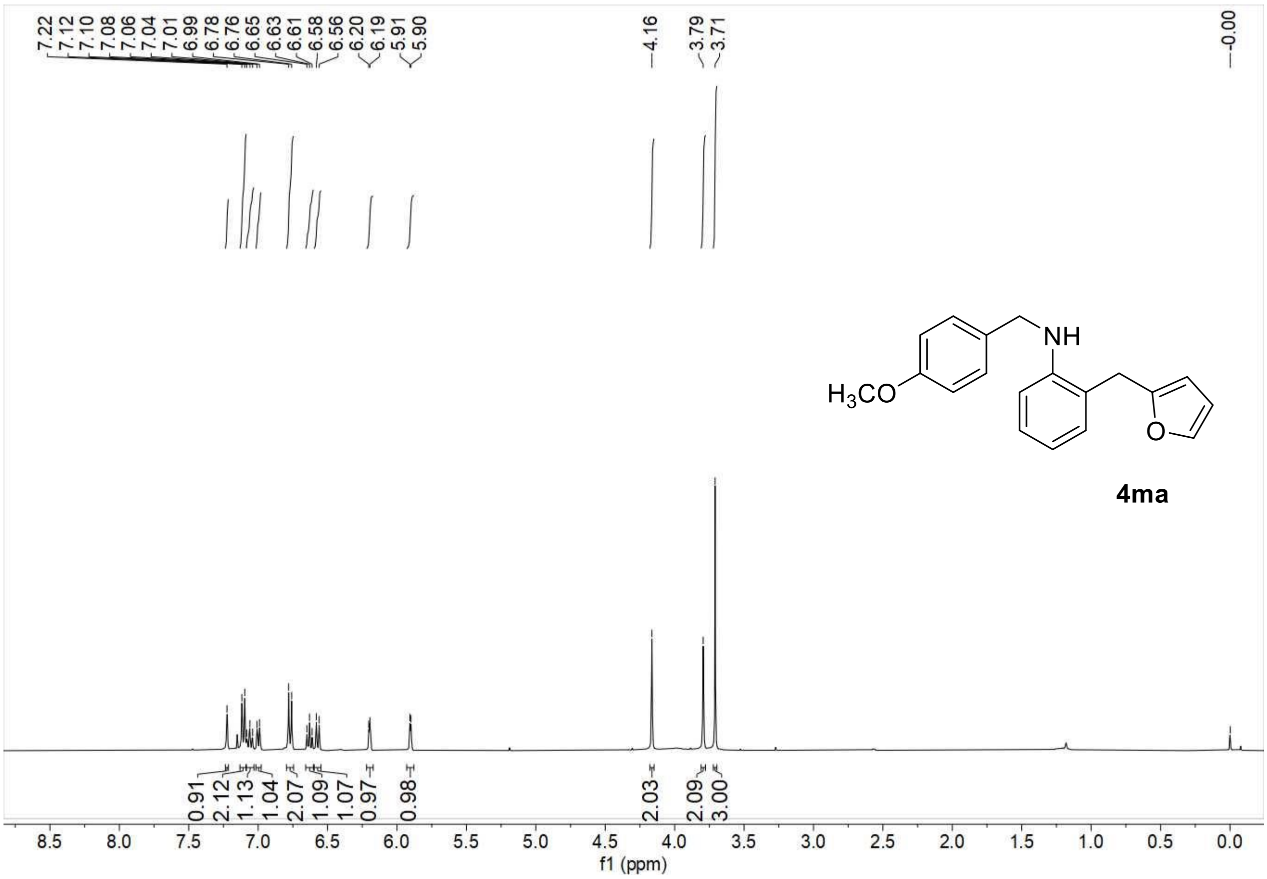
7.22
7.12
7.10
7.08
7.06
7.04
7.01
6.99
6.78
6.76
6.65
6.63
6.61
6.58
6.56
6.20
6.19
5.91
5.90
4.16
3.79
3.71
0.00
NH
H_3CO
O
4ma
0.91
2.12
1.13
1.04
2.07
1.09
1.07
0.97
0.98
2.03
2.09
3.00
f1 (ppm)

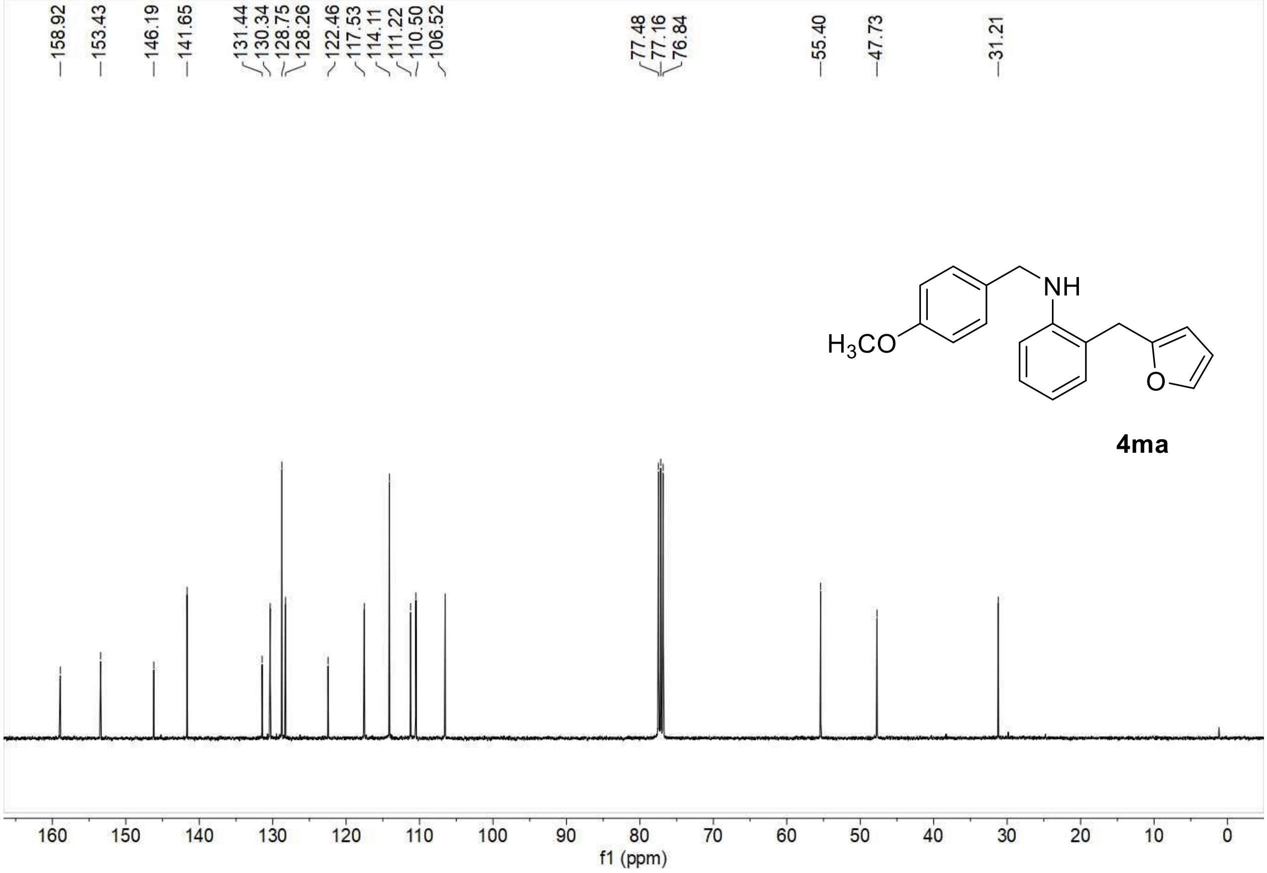
158.92
153.43
146.19
141.65
131.44
130.34
128.75
128.26
122.46
117.53
114.11
111.22
110.50
106.52
77.48
77.16
76.84
55.40
47.73
31.21
NH
H_3CO
O
4ma
f1 (ppm)

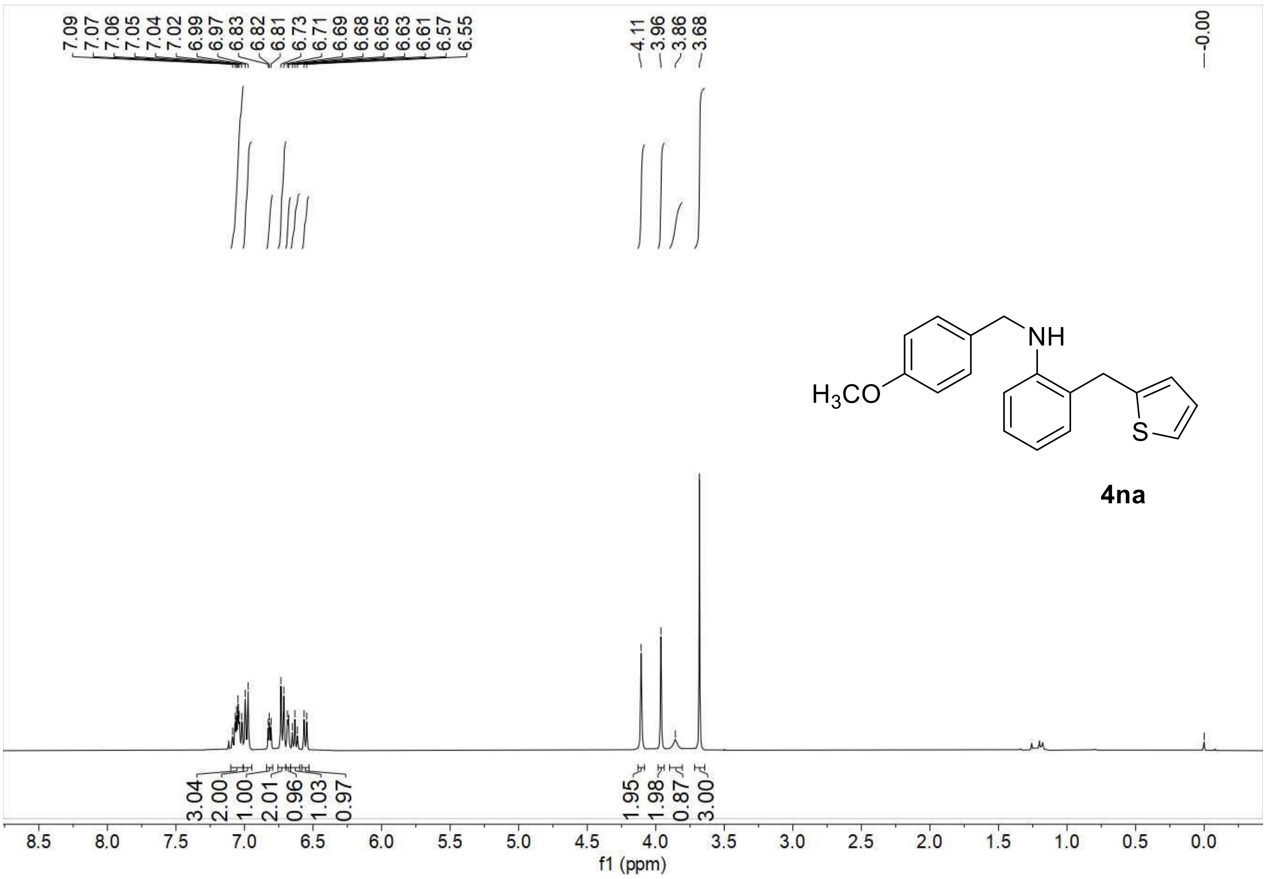

7.09
7.07
7.06
7.05
7.04
7.02
6.99
6.97
6.83
6.82
6.81
6.73
6.71
6.69
6.68
6.65
6.63
6.61
6.57
6.55
4.11
3.96
3.86
3.68
0.00
NH
H3CO
S
4na
3.04
2.00
1.00
2.01
0.96
1.03
0.97
1.95
1.98
0.87
3.00
f1 (ppm)

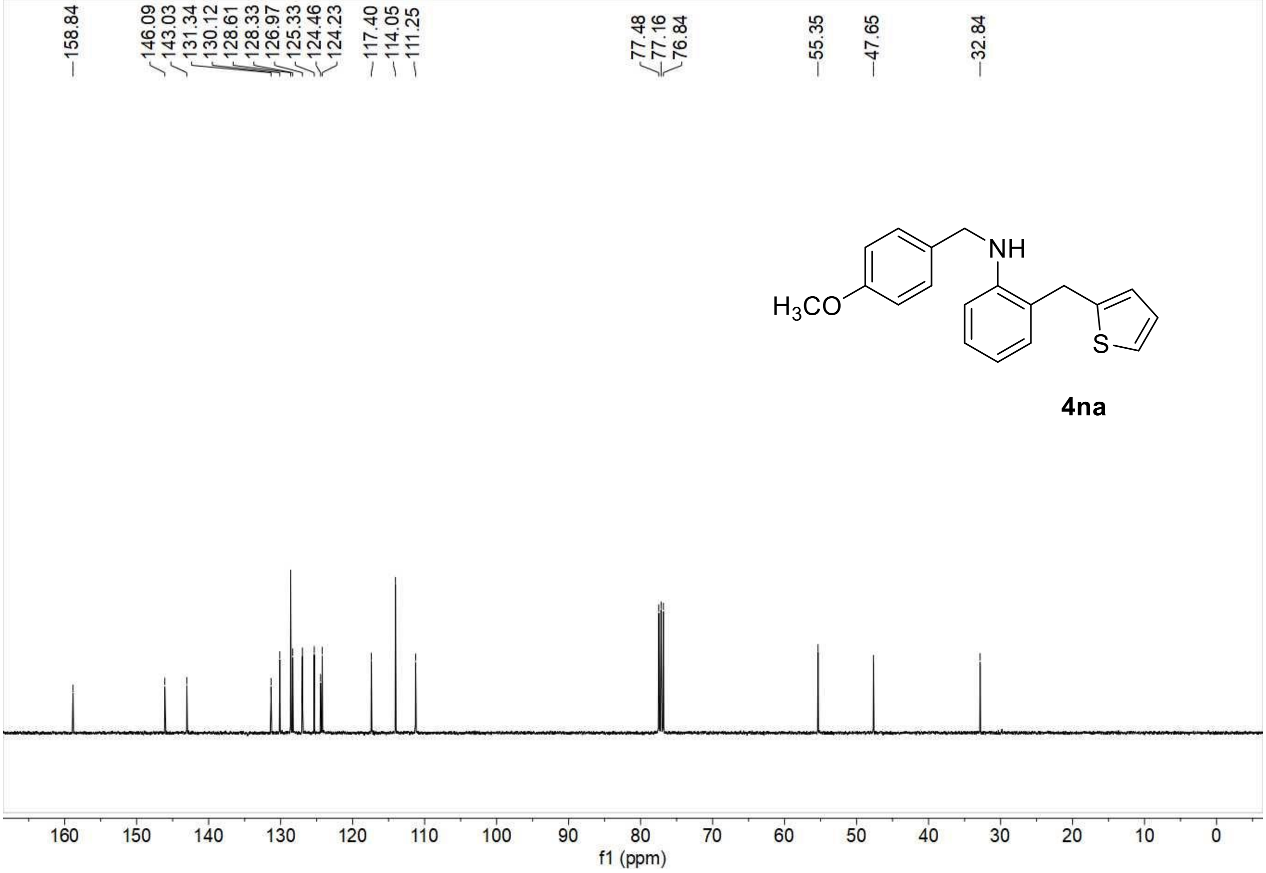

158.84
146.09
143.03
131.34
130.12
128.61
128.33
126.97
125.33
124.46
124.23
117.40
114.05
111.25
77.48
77.16
76.84
55.35
47.65
32.84
NH
H3CO
S
4na
f1 (ppm)

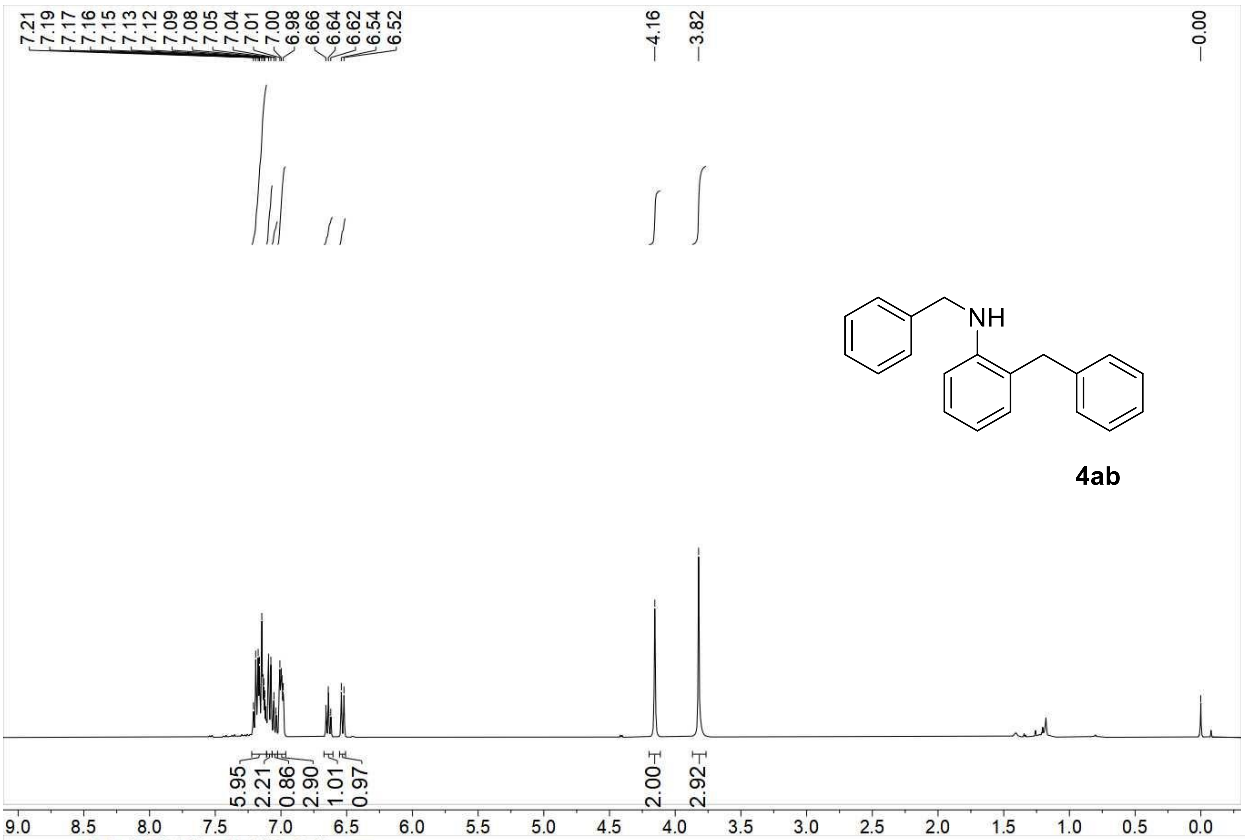
7.21 7.19 7.17 7.16 7.15 7.13 7.12 7.09 7.08 7.05 7.04 7.01 7.00 6.98 6.66 6.64 6.62 6.54 6.52
4.16
3.82
0.00
NH
4ab
5.95 2.21 0.86 2.90 1.01 0.97
2.00
2.92
9.0 8.5 8.0 7.5 7.0 6.5 6.0 5.5 5.0 4.5 4.0 3.5 3.0 2.5 2.0 1.5 1.0 0.5 0.0

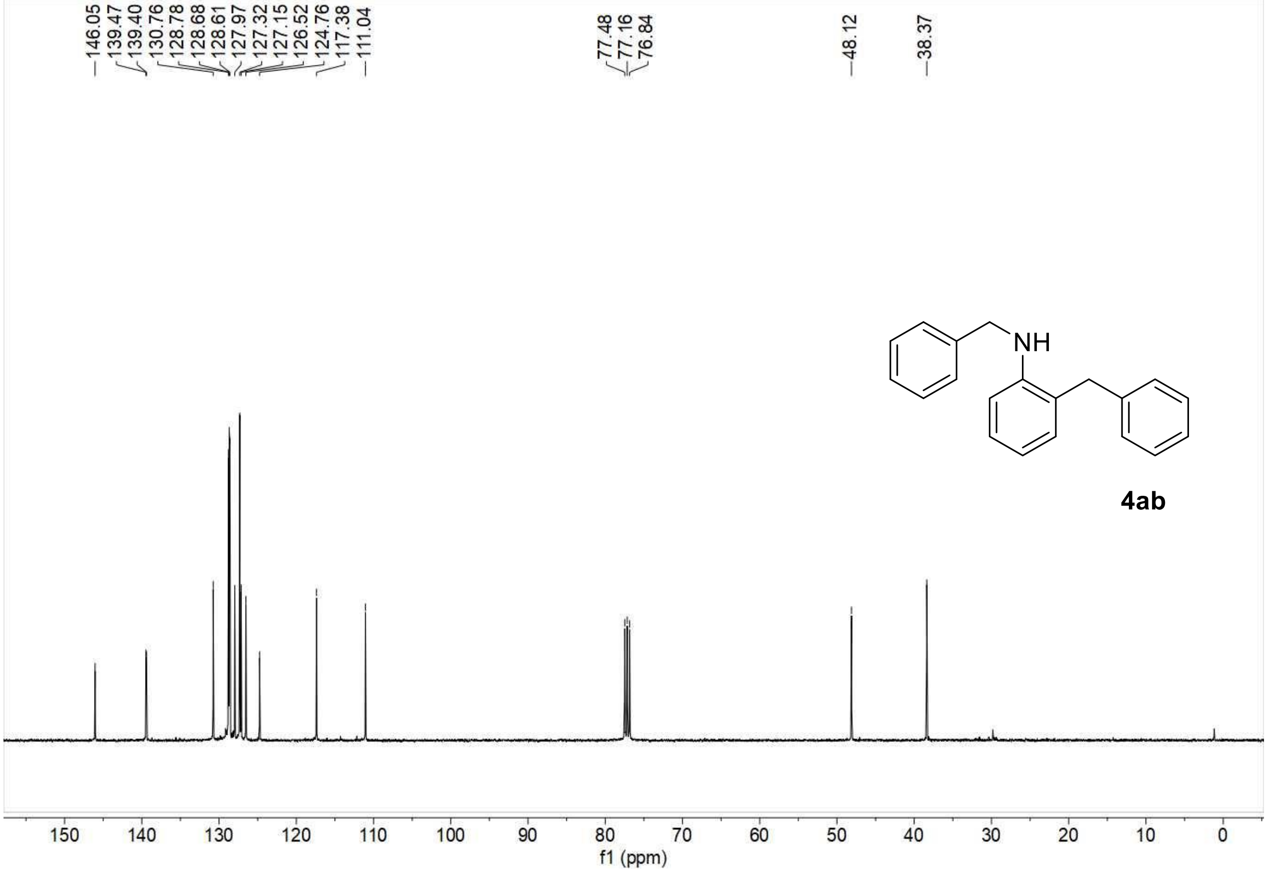
146.05 139.47 139.40 130.76 128.78 128.68 128.61 127.97 127.32 127.15 126.52 124.76 117.38 111.04
77.48 77.16 76.84
48.12
38.37
NH
4ab
150 140 130 120 110 100 90 80 70 60 50 40 30 20 10 0
f1 (ppm)

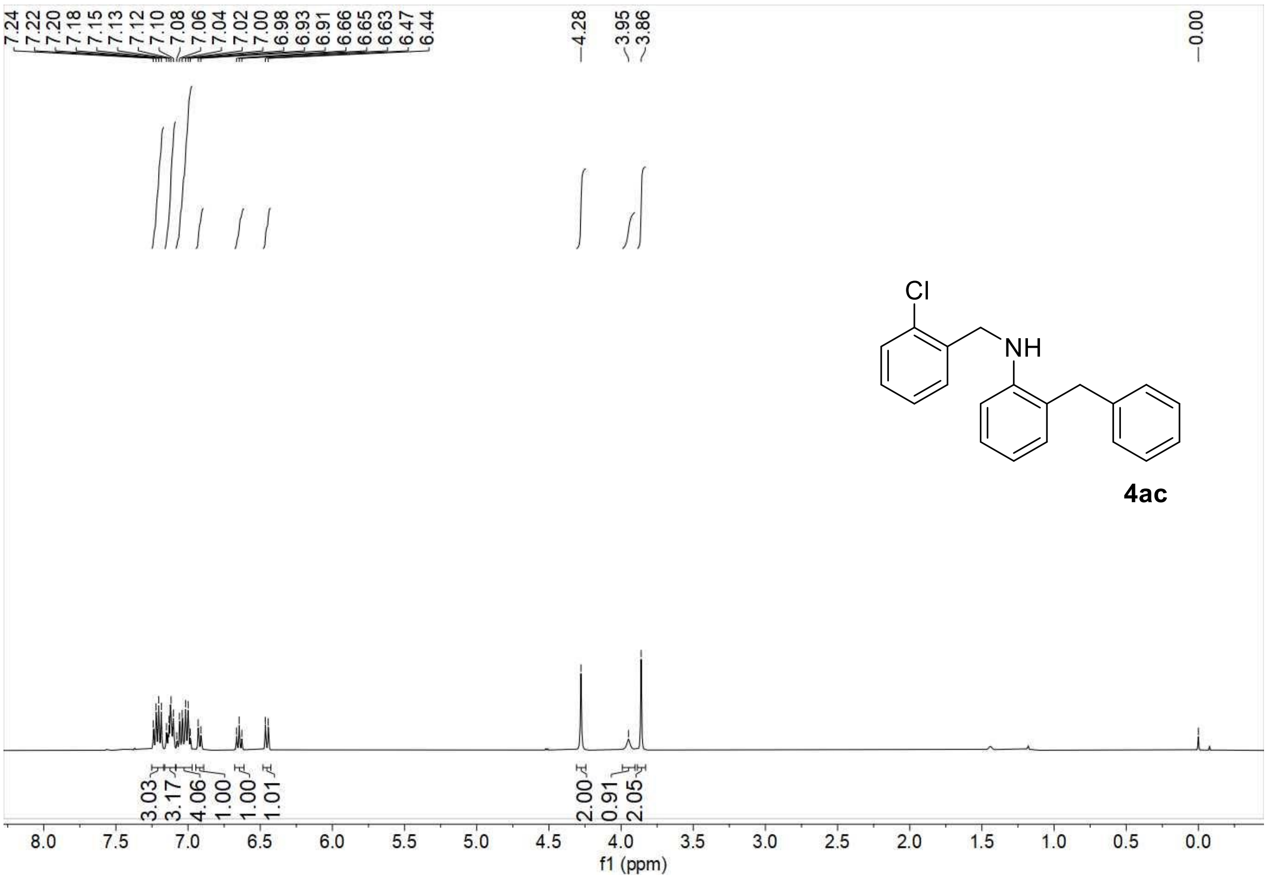
7.24 7.22 7.20 7.18 7.15 7.13 7.12 7.10 7.08 7.06 7.04 7.02 7.00 6.98 6.93 6.91 6.66 6.65 6.63 6.47 6.44
4.28
3.95
3.86
0.00
Cl
NH
4ac
3.03 3.17 4.06 1.00 1.00 1.01
2.00 0.91 2.05
8.0 7.5 7.0 6.5 6.0 5.5 5.0 4.5 4.0 3.5 3.0 2.5 2.0 1.5 1.0 0.5 0.0
f1 (ppm)

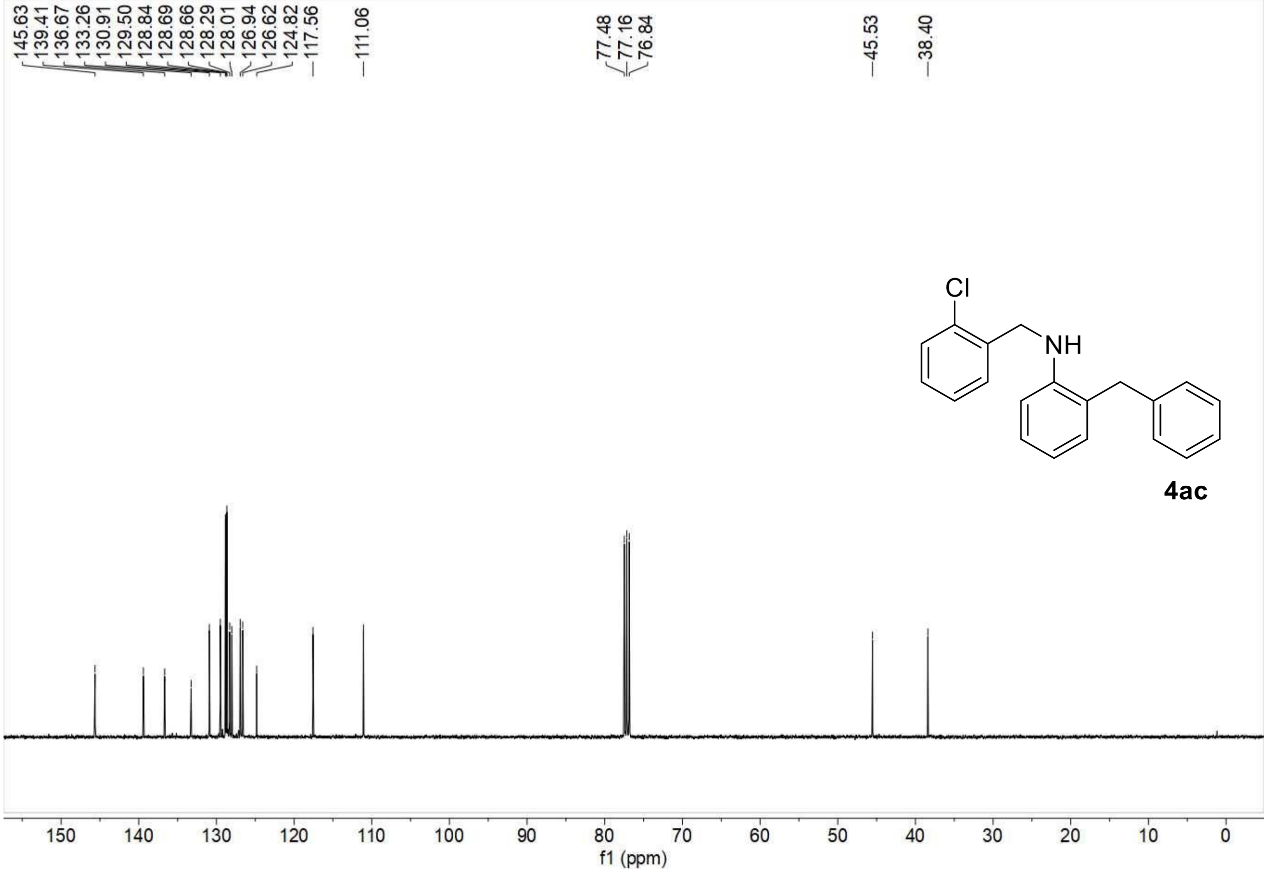
145.63 139.41 136.67 133.26 130.91 129.50 128.84 128.69 128.66 128.29 128.01 126.94 126.62 124.82 117.56
111.06
77.48 77.16 76.84
45.53
38.40
Cl
NH
4ac
150 140 130 120 110 100 90 80 70 60 50 40 30 20 10 0
f1 (ppm)

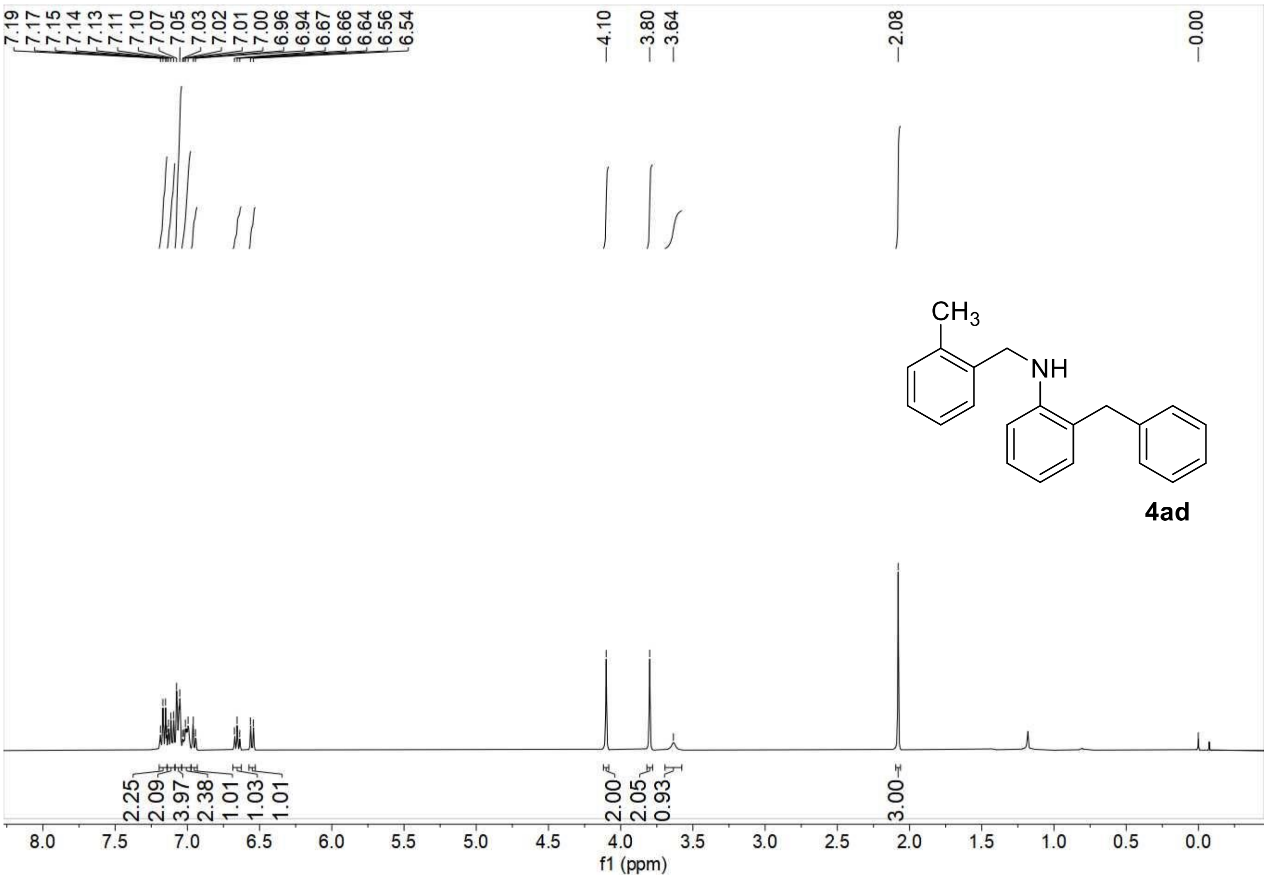
7.19
7.17
7.15
7.14
7.13
7.11
7.10
7.07
7.05
7.03
7.02
7.01
7.00
6.96
6.94
6.67
6.66
6.64
6.56
6.54
4.10
3.80
3.64
2.08
0.00
CH3
NH
4ad
2.25
2.09
3.97
2.38
1.01
1.03
1.01
2.00
2.05
0.93
3.00
f1 (ppm)

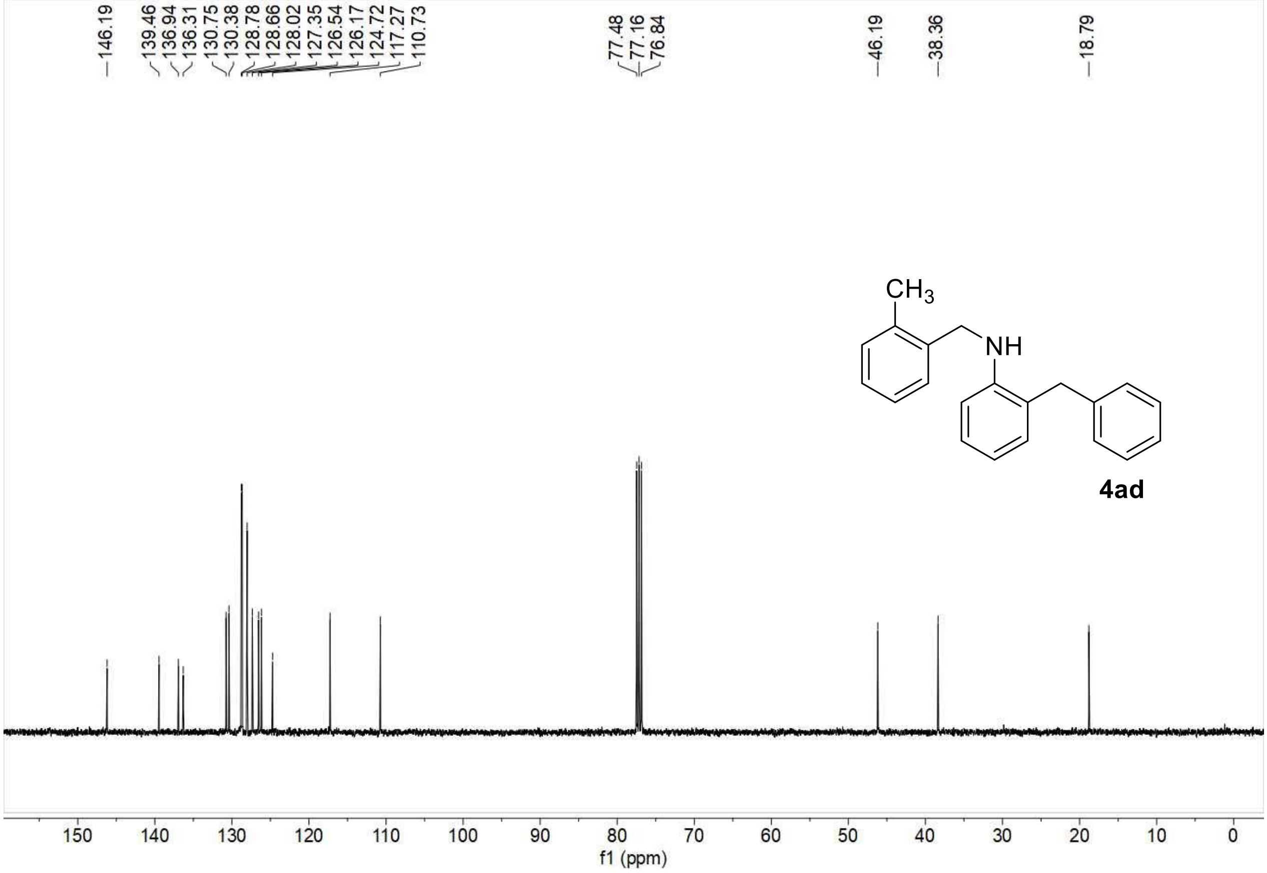
146.19
139.46
136.94
136.31
130.75
130.38
128.78
128.66
128.02
127.35
126.54
126.17
124.72
117.27
110.73
77.48
77.16
76.84
46.19
38.36
18.79
CH3
NH
4ad
f1 (ppm)

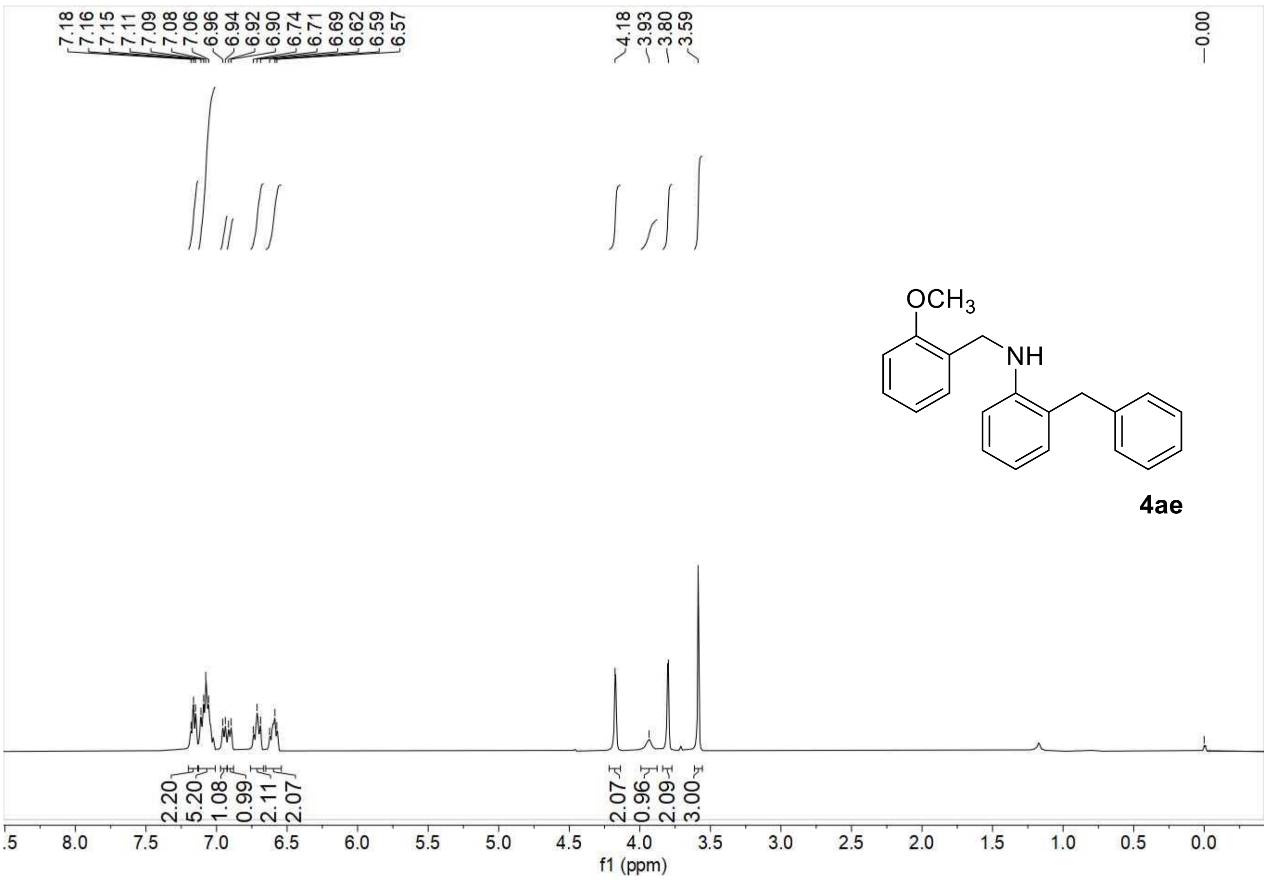
7.18
7.16
7.15
7.11
7.09
7.08
7.06
6.96
6.94
6.92
6.90
6.74
6.71
6.69
6.62
6.59
6.57
4.18
3.93
3.80
3.59
0.00
OCH3
NH
4ae
2.20
5.20
1.08
0.99
2.11
2.07
2.07
0.96
2.09
3.00
f1 (ppm)

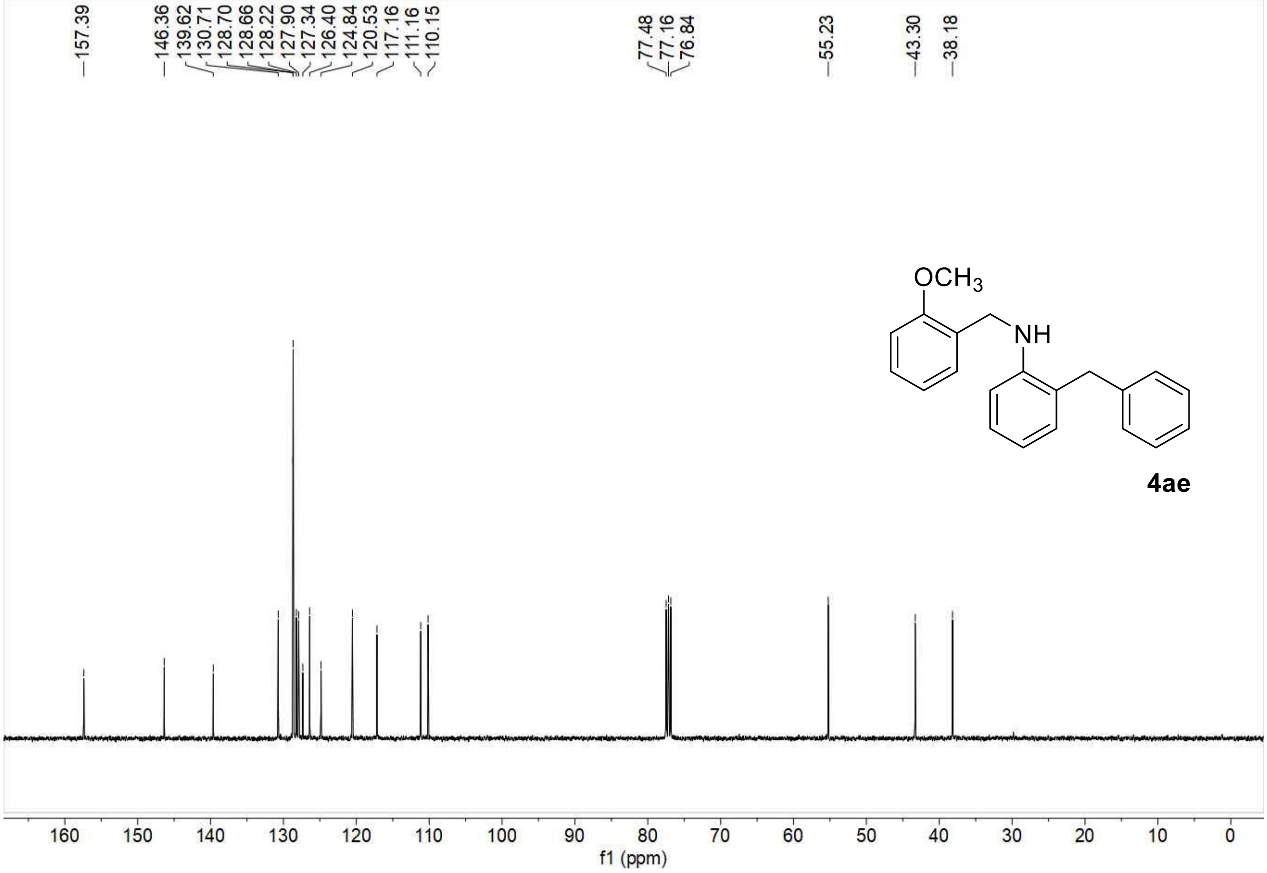
157.39
146.36
139.62
130.71
128.70
128.66
128.22
127.90
127.34
126.40
124.84
120.53
117.16
111.16
110.15
77.48
77.16
76.84
55.23
43.30
38.18
OCH3
NH
4ae
f1 (ppm)

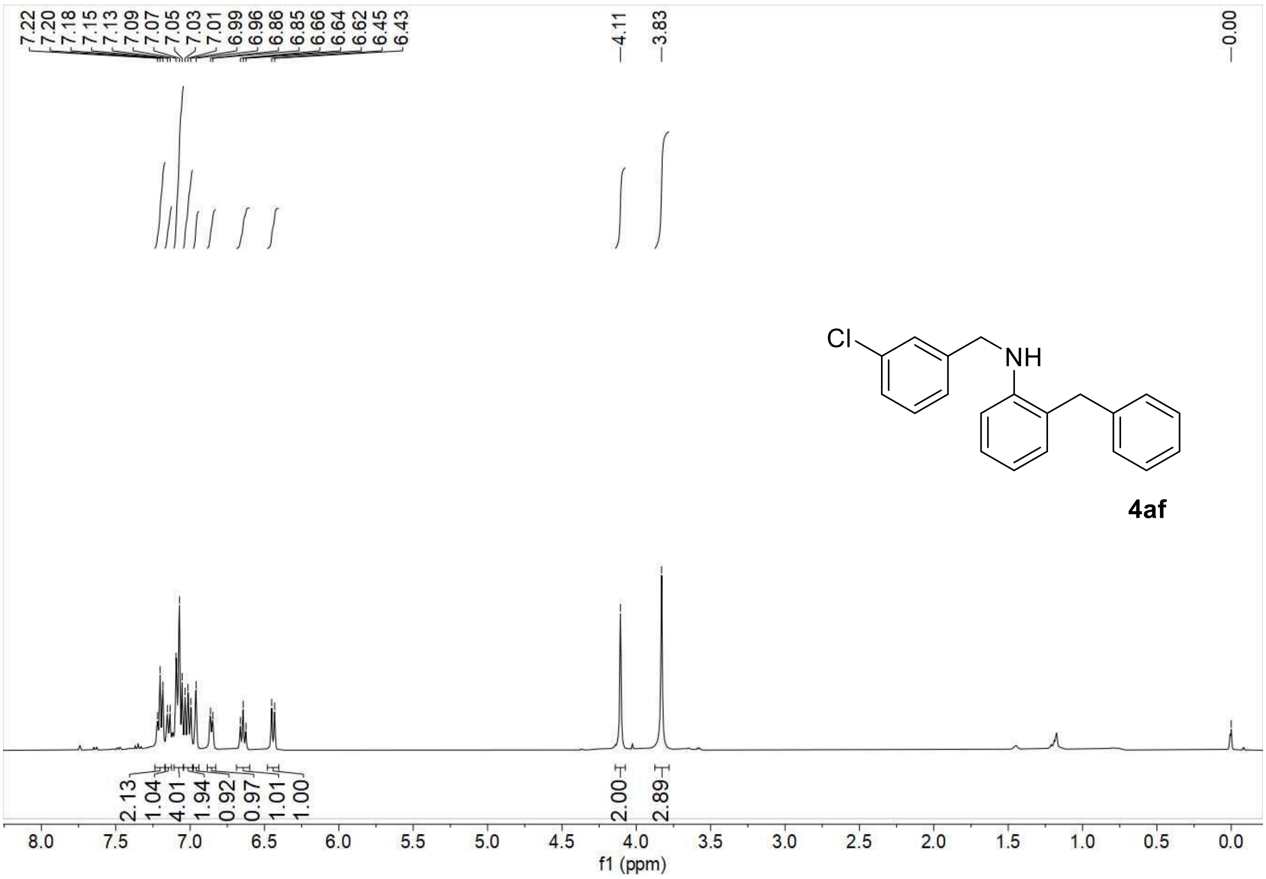
7.22
7.20
7.18
7.15
7.13
7.09
7.07
7.05
7.03
7.01
6.99
6.96
6.86
6.85
6.66
6.64
6.62
6.45
6.43
4.11
3.83
0.00
Cl
NH
4af
2.13
1.04
4.01
1.94
0.92
0.97
1.01
1.00
2.00
2.89
f1 (ppm)

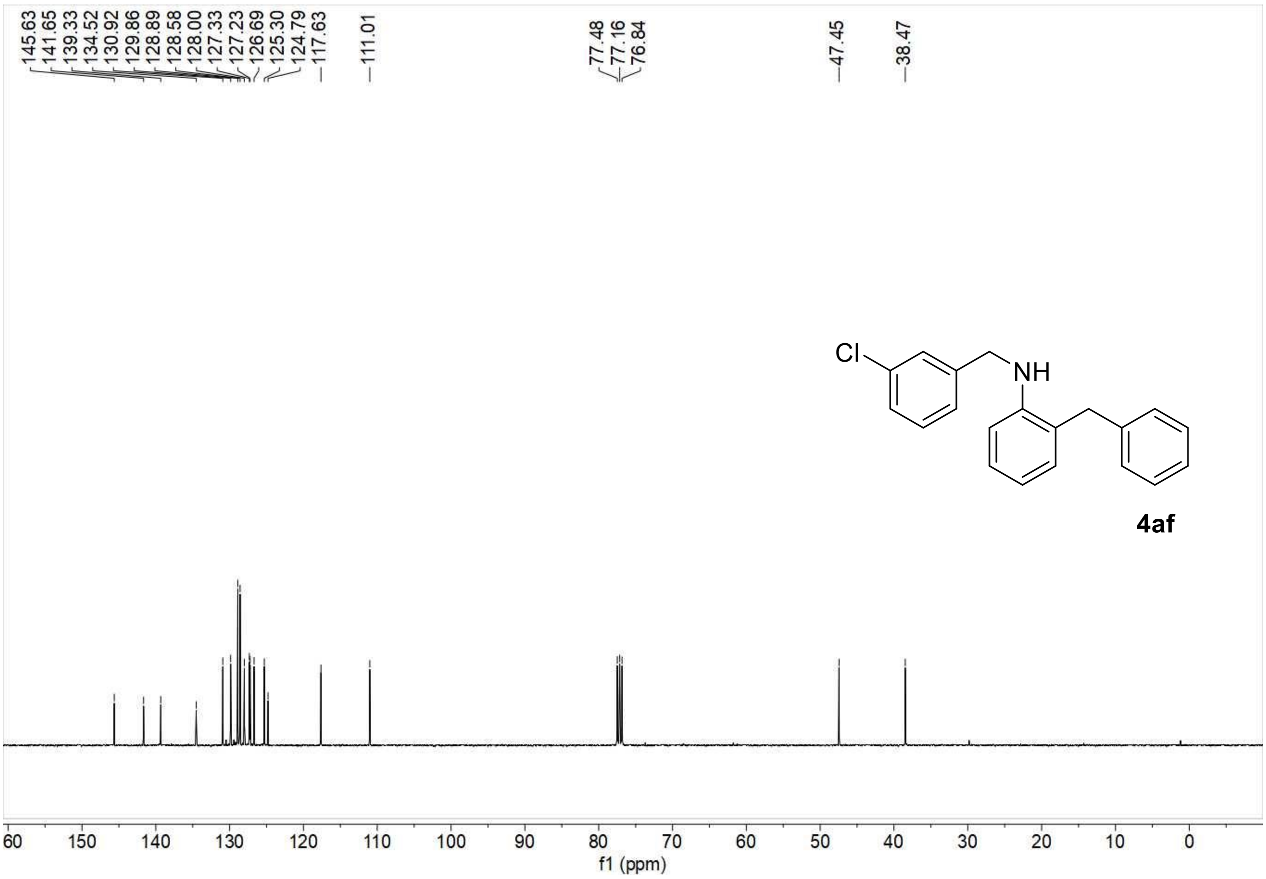
145.63
141.65
139.33
134.52
130.92
129.86
128.89
128.58
128.00
127.33
127.23
126.69
125.30
124.79
117.63
111.01
77.48
77.16
76.84
47.45
38.47
Cl
NH
4af
f1 (ppm)

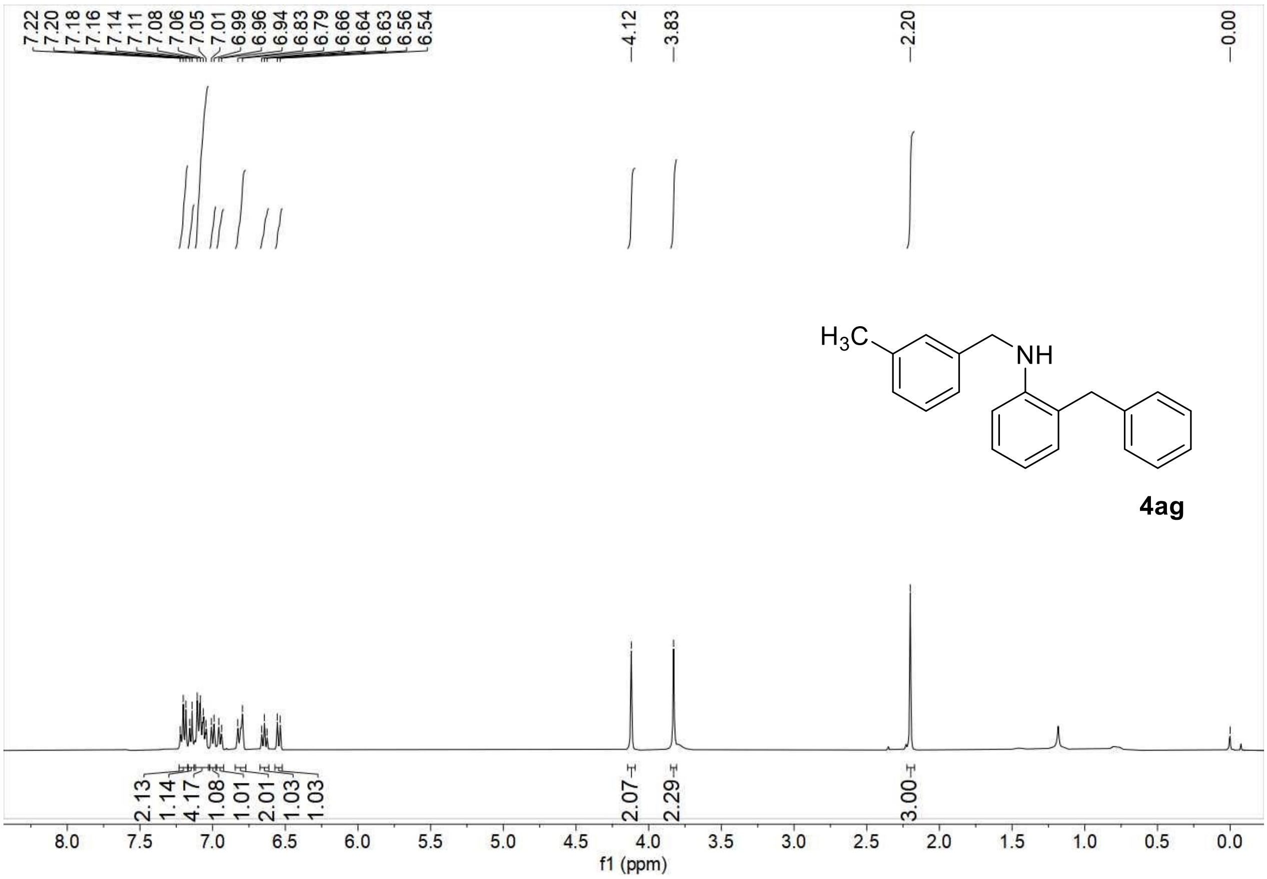
7.22
7.20
7.18
7.16
7.14
7.11
7.08
7.06
7.05
7.01
6.99
6.96
6.94
6.83
6.79
6.66
6.64
6.63
6.56
6.54
4.12
3.83
2.20
0.00
H3C
NH
4ag
2.13
1.14
4.17
1.08
1.01
2.01
1.03
1.03
2.07
2.29
3.00
f1 (ppm)

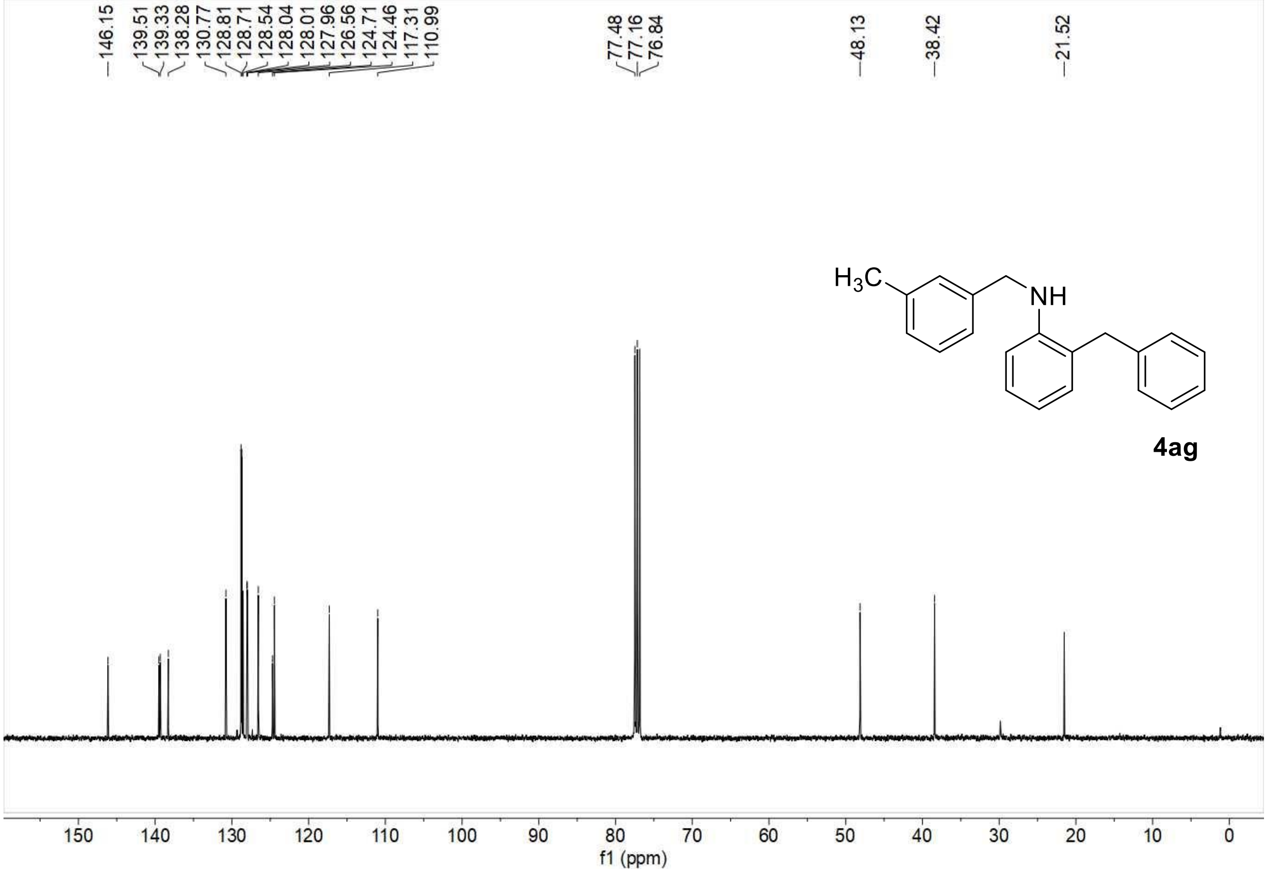
146.15
139.51
139.33
138.28
130.77
128.81
128.71
128.54
128.04
128.01
127.96
126.56
124.71
124.46
117.31
110.99
77.48
77.16
76.84
48.13
38.42
21.52
H3C
NH
4ag
f1 (ppm)

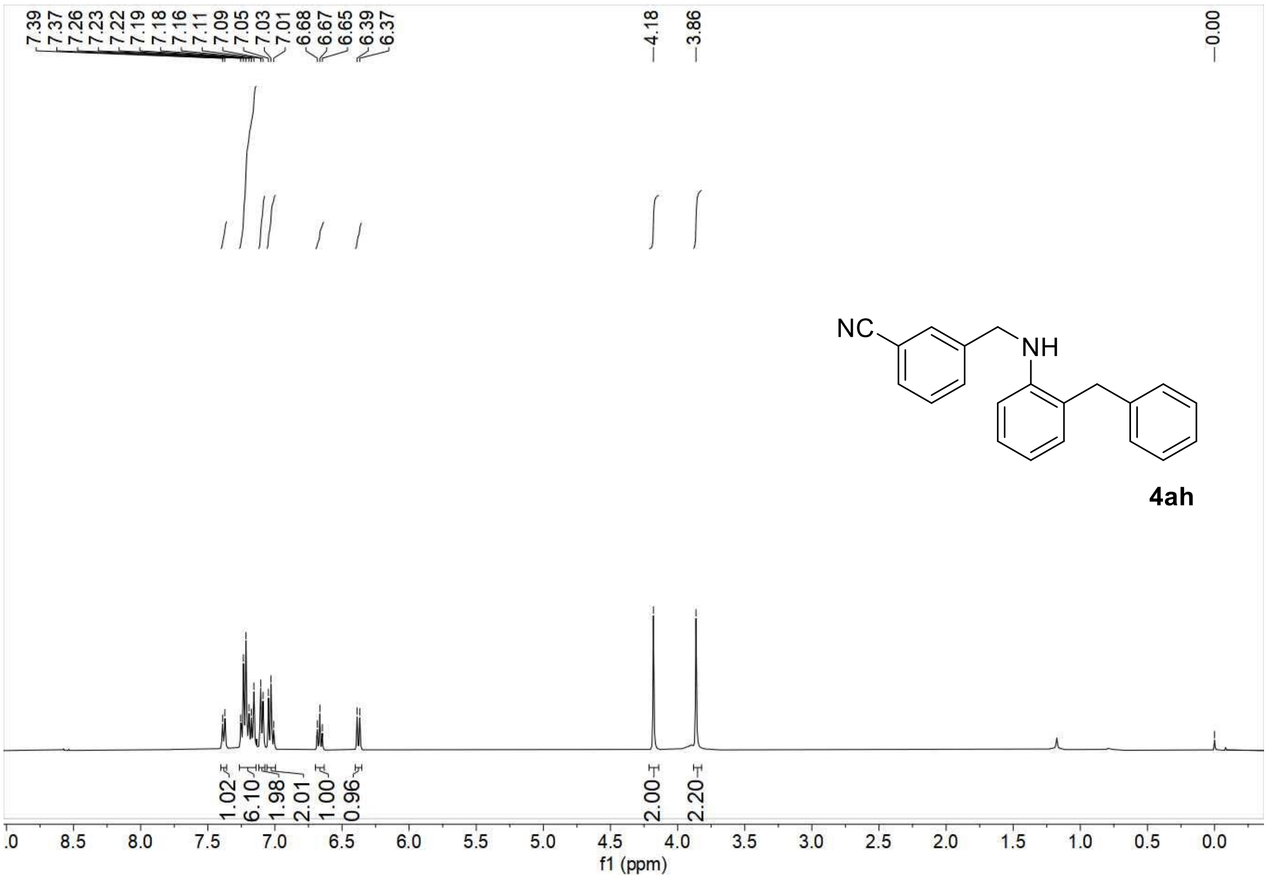
7.39
7.37
7.26
7.23
7.22
7.19
7.18
7.16
7.11
7.09
7.05
7.03
7.01
6.68
6.67
6.65
6.39
6.37
4.18
3.86
0.00
NC
NH
4ah
1.02
6.10
1.98
2.01
1.00
0.96
2.00
2.20
f1 (ppm)

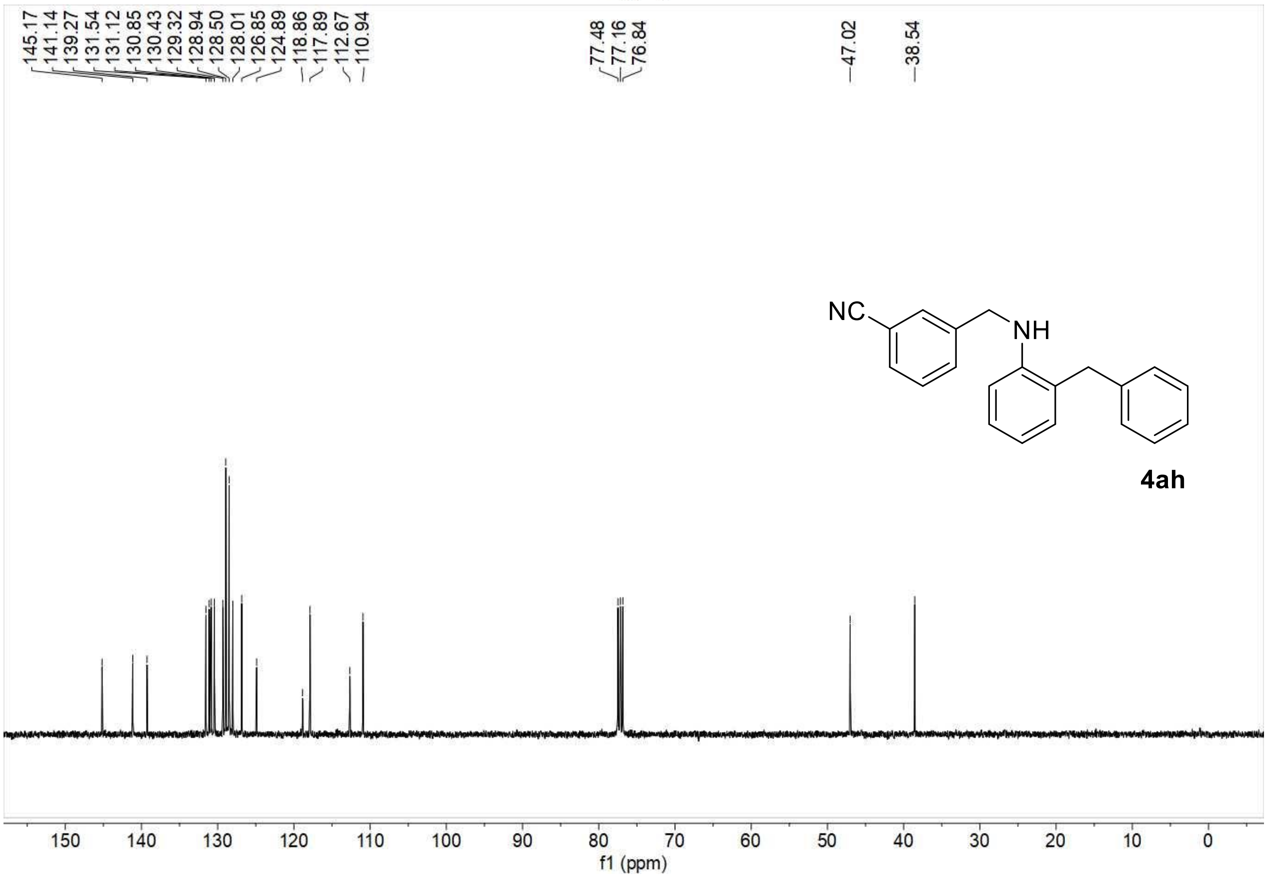
145.17
141.14
139.27
131.54
131.12
130.85
130.43
129.32
128.94
128.50
128.01
126.85
124.89
118.86
117.89
112.67
110.94
77.48
77.16
76.84
47.02
38.54
NC
NH
4ah
f1 (ppm)

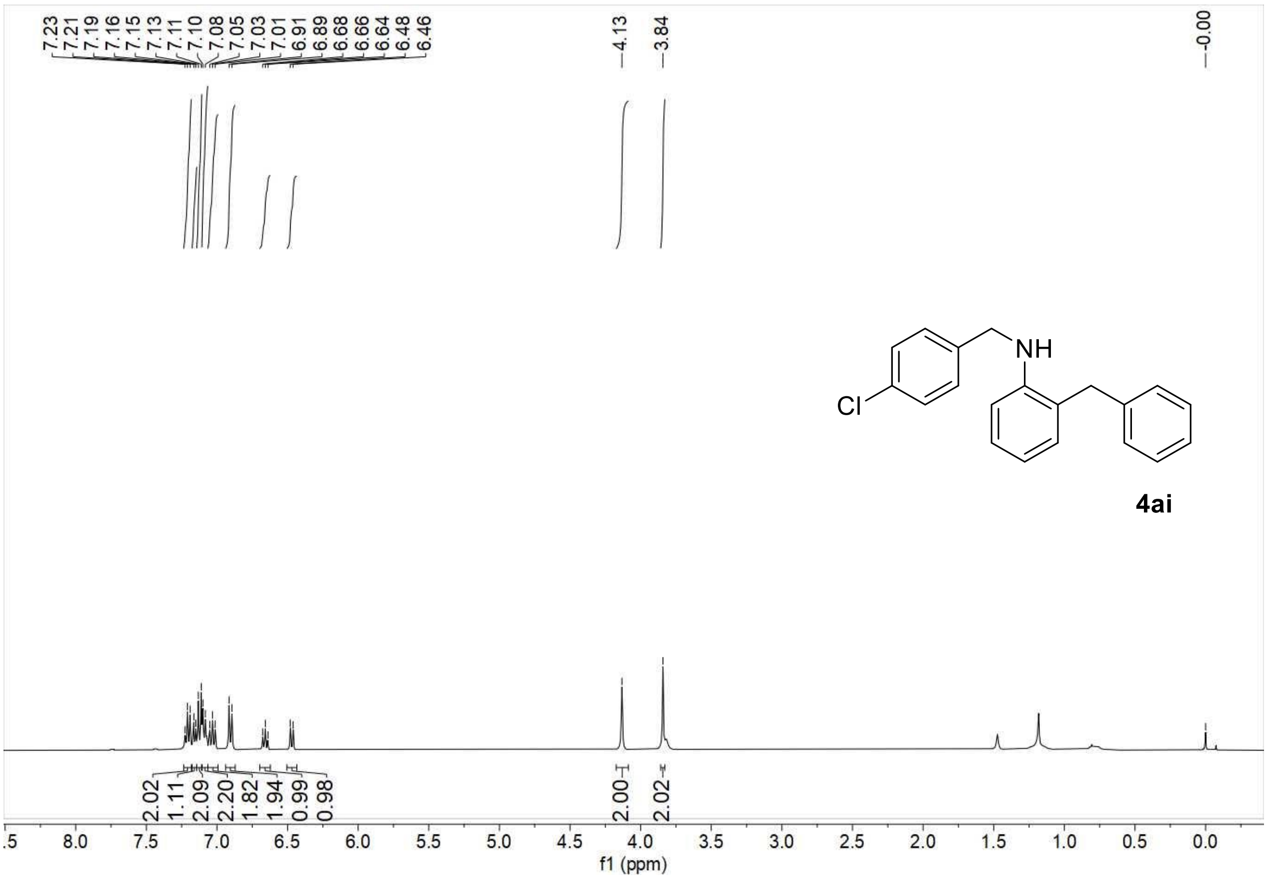
7.23 7.21 7.19 7.16 7.15 7.13 7.11 7.10 7.08 7.05 7.03 7.01 6.91 6.89 6.68 6.66 6.64 6.48 6.46
4.13
3.84
0.00
NH
Cl
4ai
2.02 1.11 2.09 2.20 1.82 1.94 0.99 0.98
2.00
2.02
f1 (ppm)

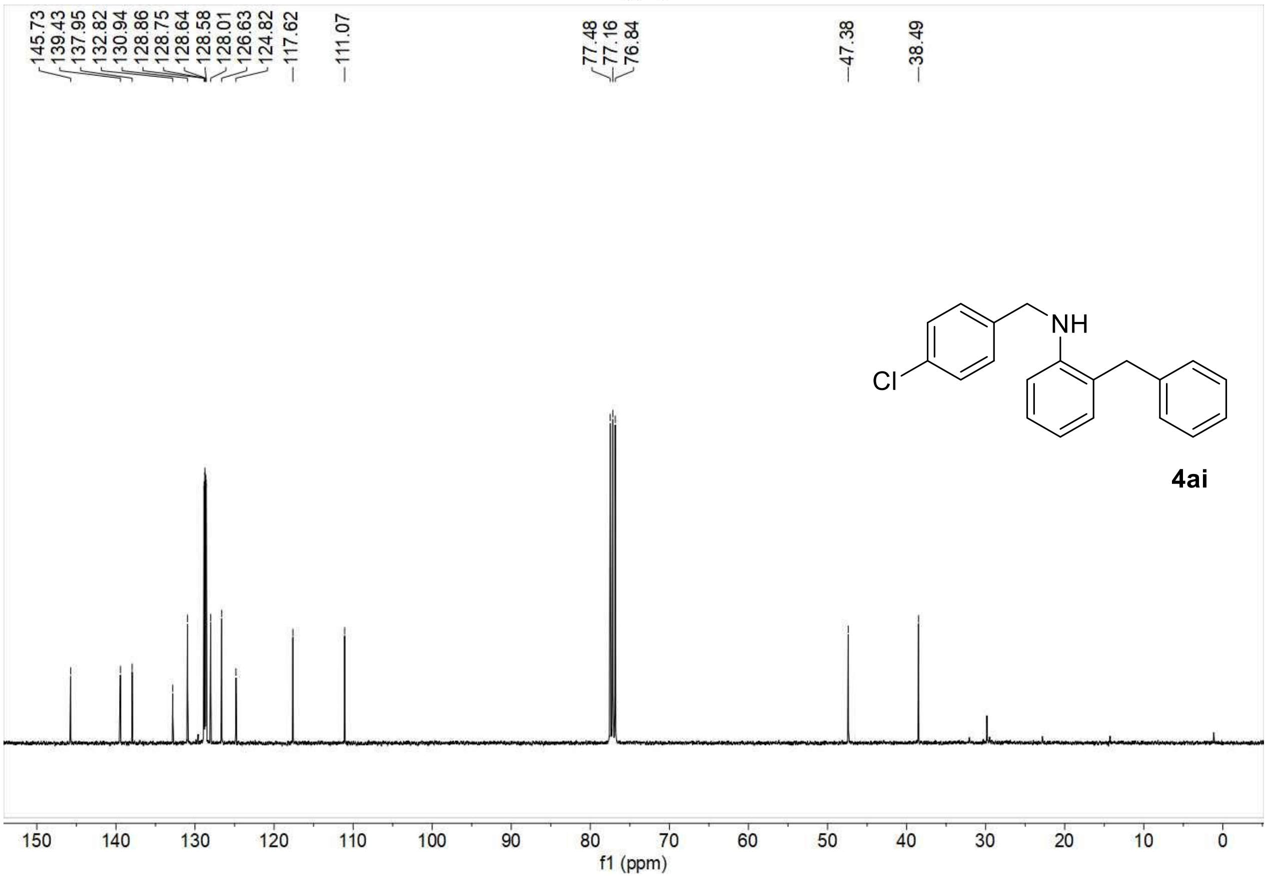
145.73 139.43 137.95 132.82 130.94 128.86 128.75 128.64 128.58 128.01 126.63 124.82 117.62
111.07
77.48 77.16 76.84
47.38
38.49
NH
Cl
4ai
f1 (ppm)

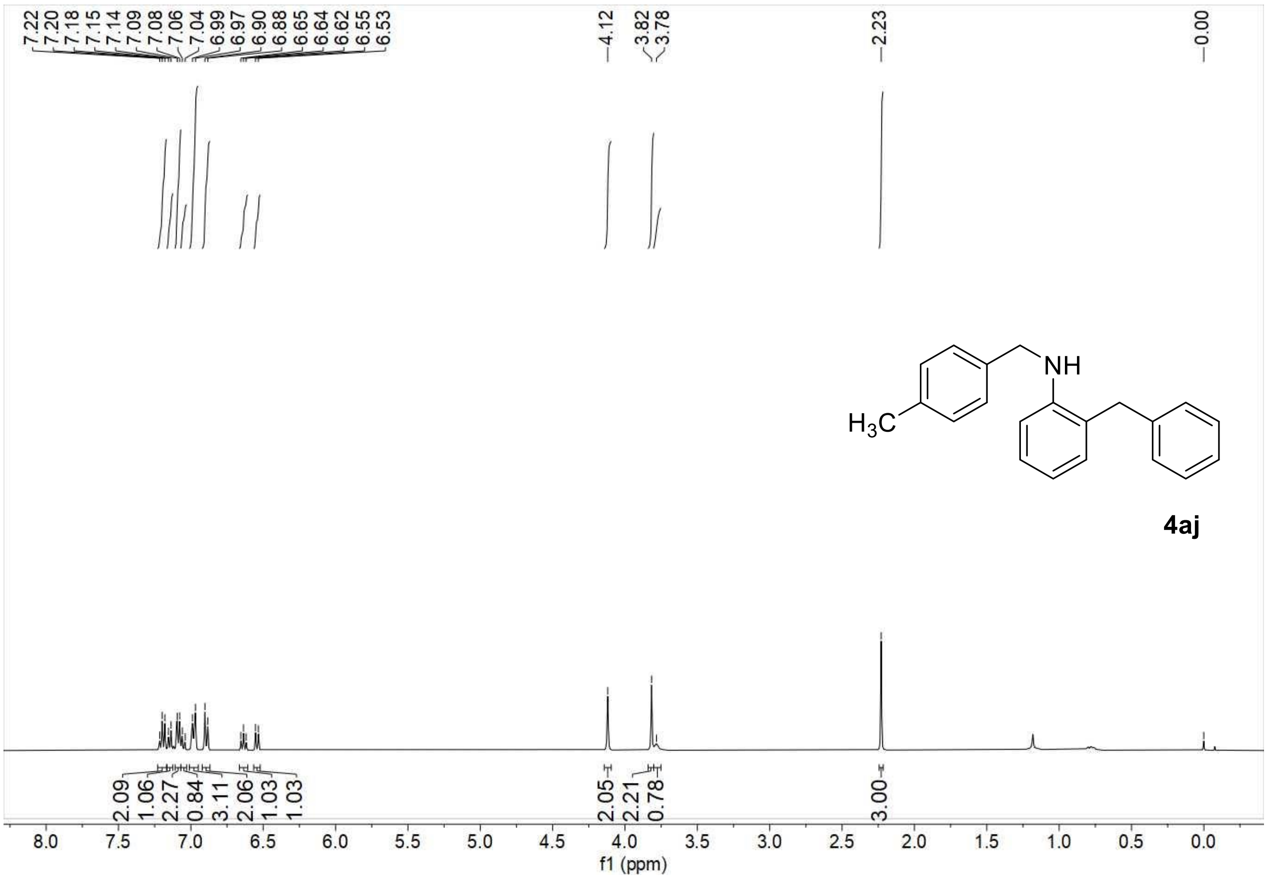
7.22
7.20
7.18
7.15
7.14
7.09
7.08
7.06
7.04
6.99
6.97
6.90
6.88
6.65
6.64
6.62
6.55
6.53
4.12
3.82
3.78
2.23
0.00
NH
H_3C
2.09
1.06
2.27
0.84
3.11
2.06
1.03
1.03
2.05
2.21
0.78
3.00
f1 (ppm)

4aj

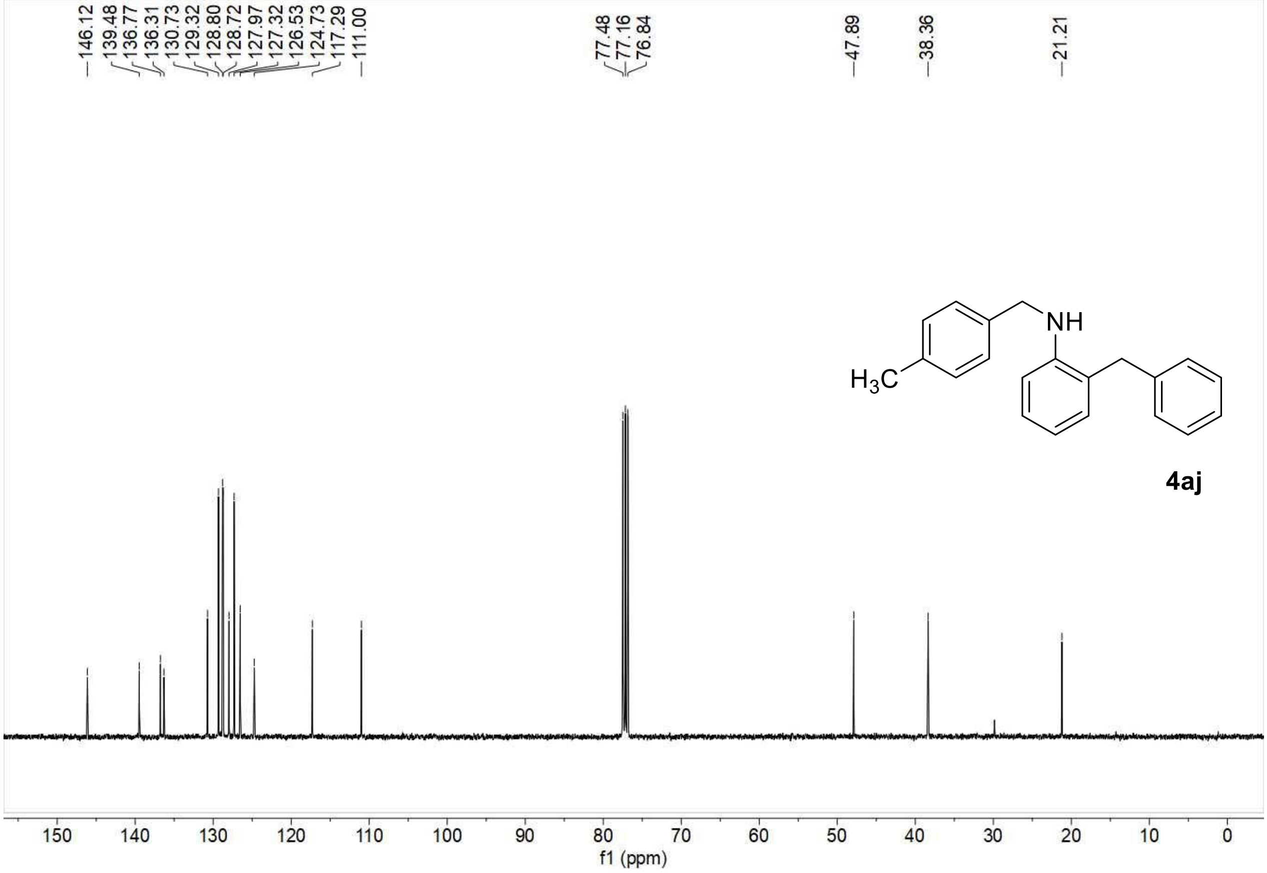
146.12
139.48
136.77
136.31
130.73
129.32
128.80
128.72
127.97
127.32
126.53
124.73
117.29
111.00
77.48
77.16
76.84
47.89
38.36
21.21
NH
H_3C
f1 (ppm)

4aj

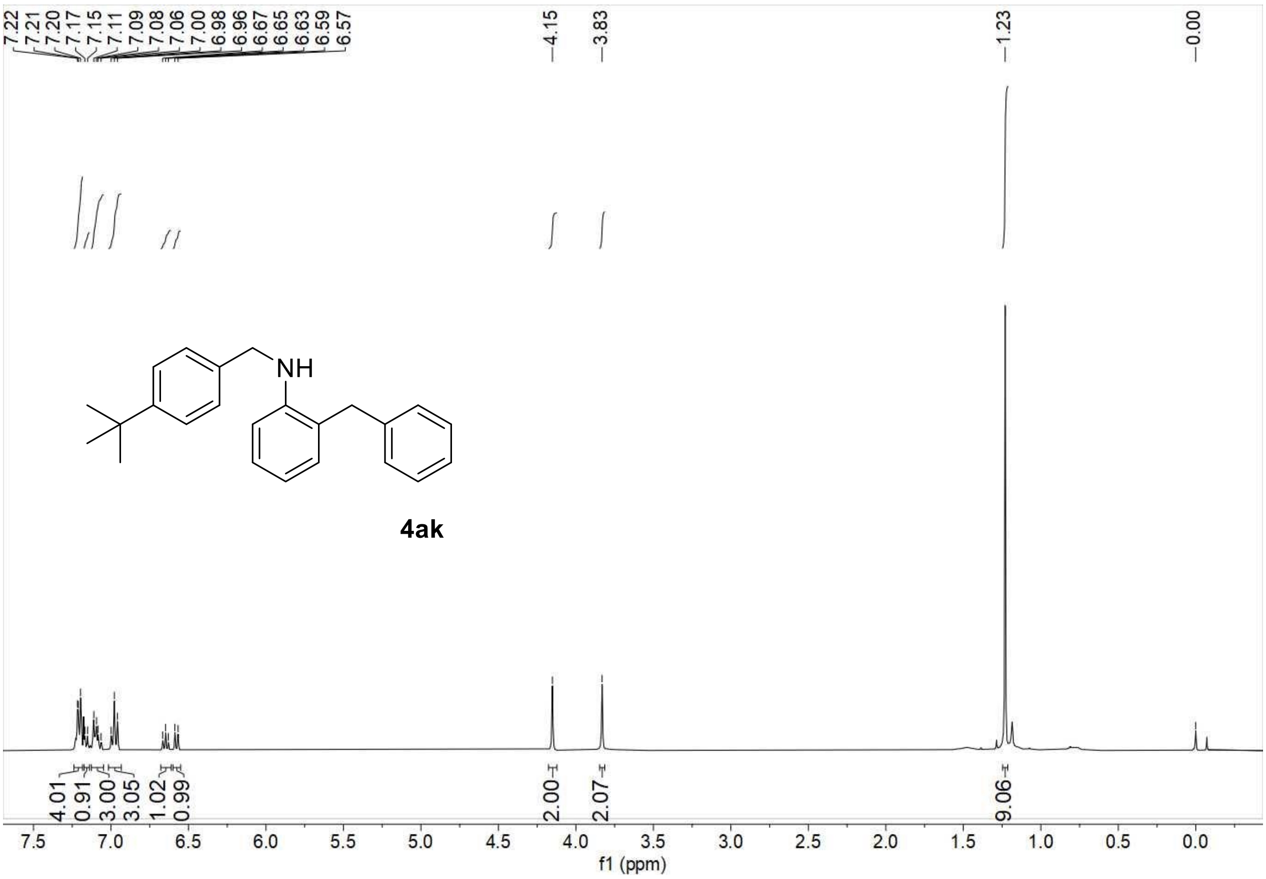
7.22
7.21
7.20
7.17
7.15
7.11
7.09
7.08
7.06
7.00
6.98
6.96
6.67
6.65
6.63
6.59
6.57
4.15
3.83
1.23
0.00
NH
4ak
4.01
0.91
3.00
3.05
1.02
0.99
2.00
2.07
9.06
7.5 7.0 6.5 6.0 5.5 5.0 4.5 4.0 3.5 3.0 2.5 2.0 1.5 1.0 0.5 0.0
f1 (ppm)

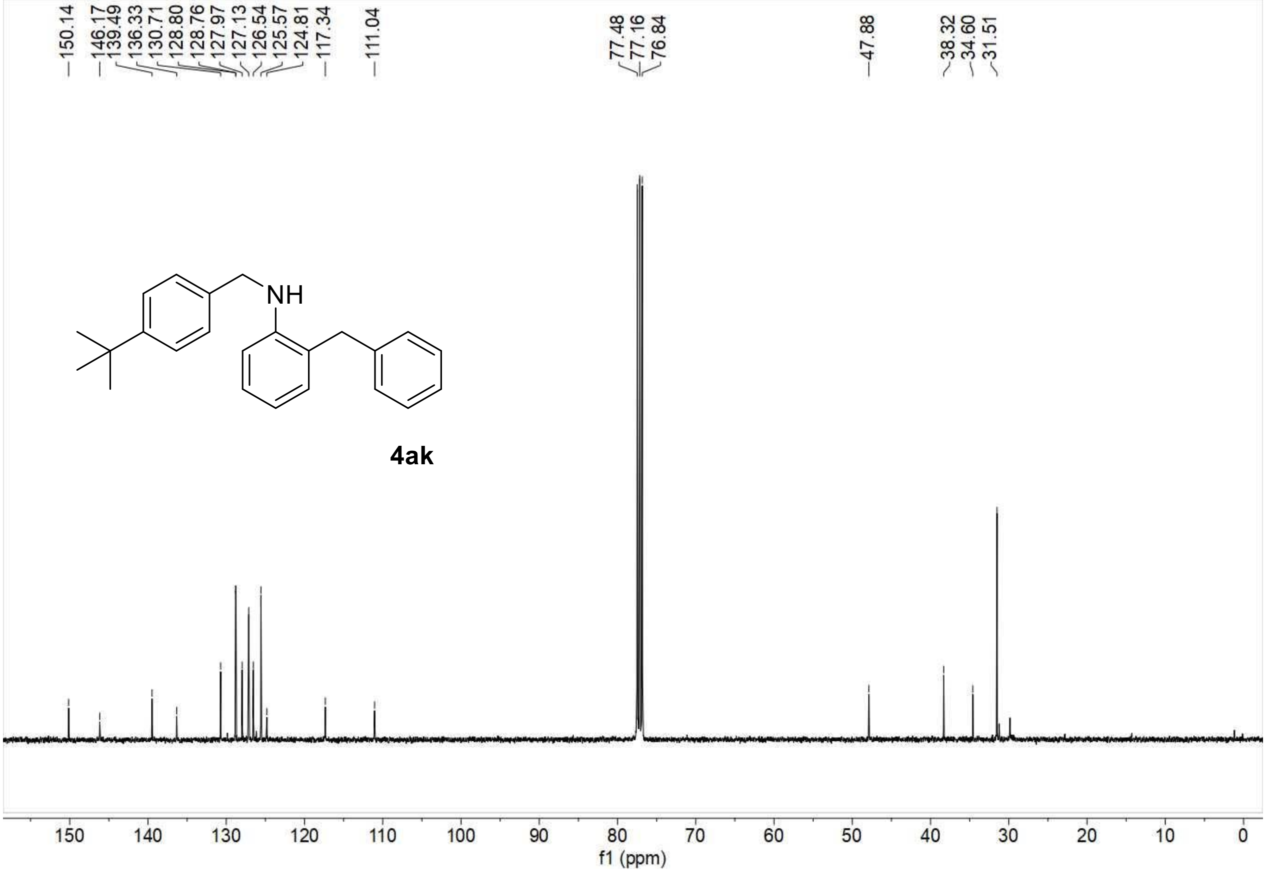
150.14
146.17
139.49
136.33
130.71
128.80
128.76
127.97
127.13
126.54
125.57
124.81
117.34
111.04
77.48
77.16
76.84
47.88
38.32
34.60
31.51
NH
4ak
150 140 130 120 110 100 90 80 70 60 50 40 30 20 10 0
f1 (ppm)

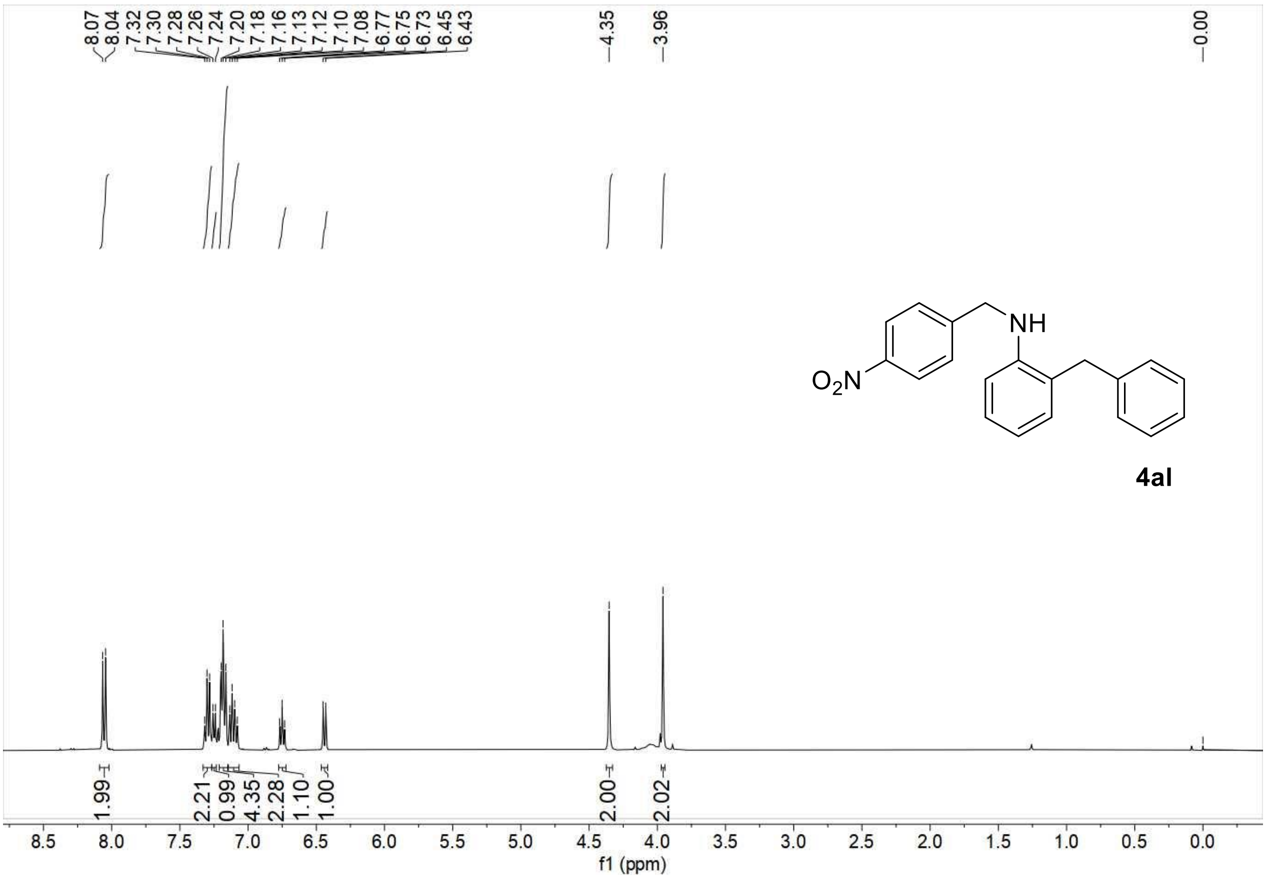

8.07 8.04 7.32 7.30 7.28 7.26 7.24 7.20 7.18 7.16 7.13 7.12 7.10 7.08 6.77 6.75 6.73 6.45 6.43
4.35
3.96
0.00
NH
O2N
4al
1.99
2.21 0.99 4.35 2.28 1.10 1.00
2.00
2.02
8.5 8.0 7.5 7.0 6.5 6.0 5.5 5.0 4.5 4.0 3.5 3.0 2.5 2.0 1.5 1.0 0.5 0.0
f1 (ppm)

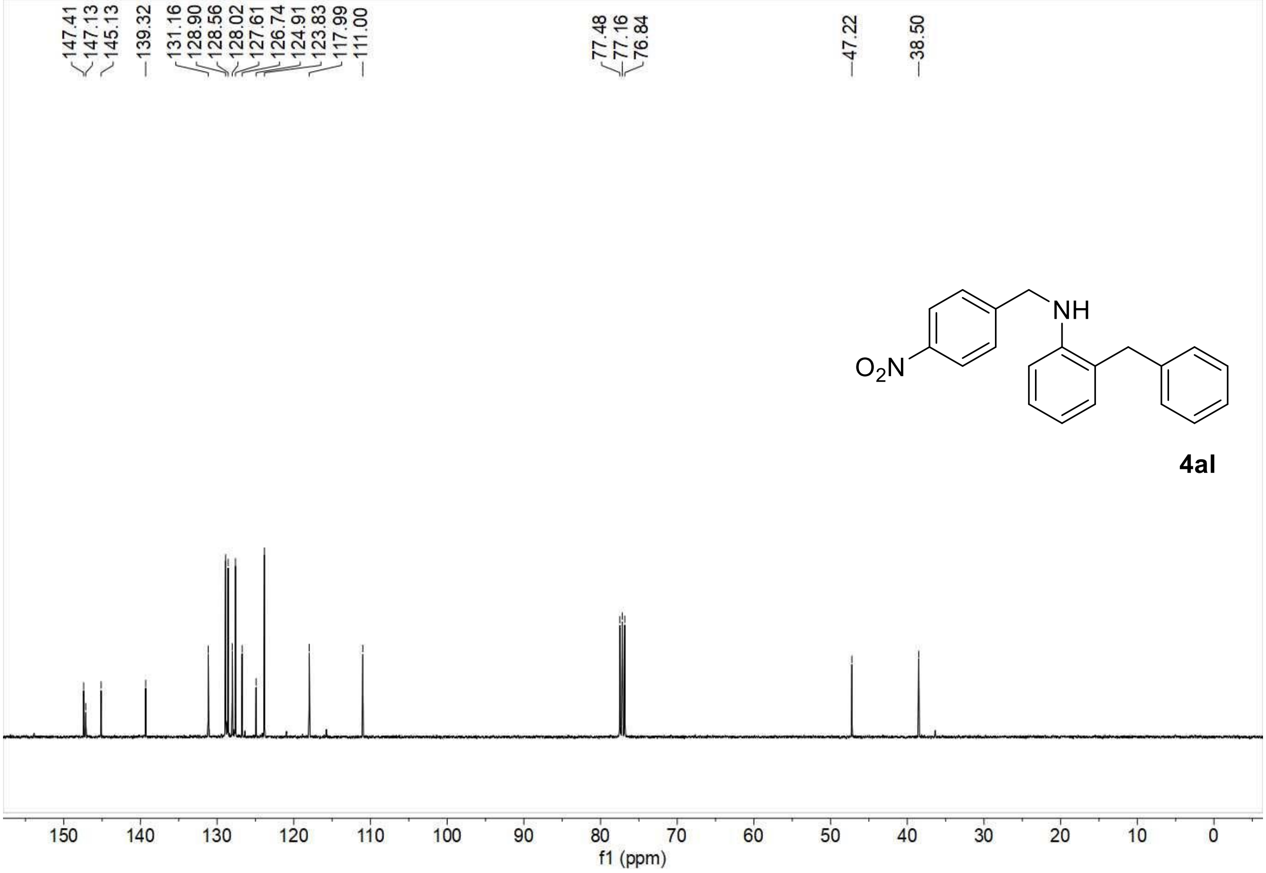

147.41 147.13 145.13
139.32
131.16 128.90 128.56 128.02 127.61 126.74 124.91 123.83 117.99
111.00
77.48 77.16 76.84
47.22
38.50
NH
O2N
4al
150 140 130 120 110 100 90 80 70 60 50 40 30 20 10 0
f1 (ppm)

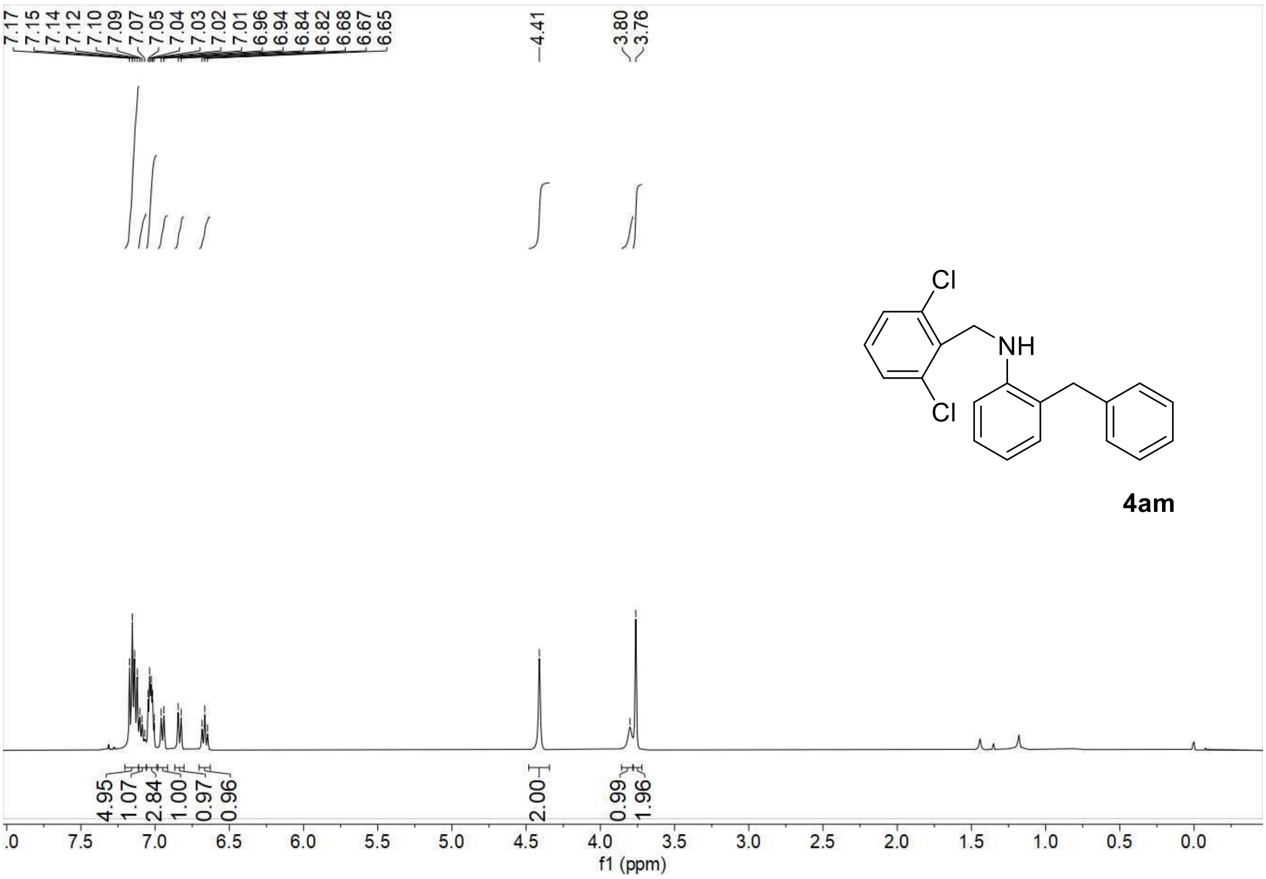
7.17
7.15
7.14
7.12
7.10
7.09
7.07
7.05
7.04
7.03
7.02
7.01
6.96
6.94
6.84
6.82
6.68
6.67
6.65
4.41
3.80
3.76
Cl
NH
Cl
4.95
1.07
2.84
1.00
0.97
0.96
2.00
0.99
1.96
f1 (ppm)

4am

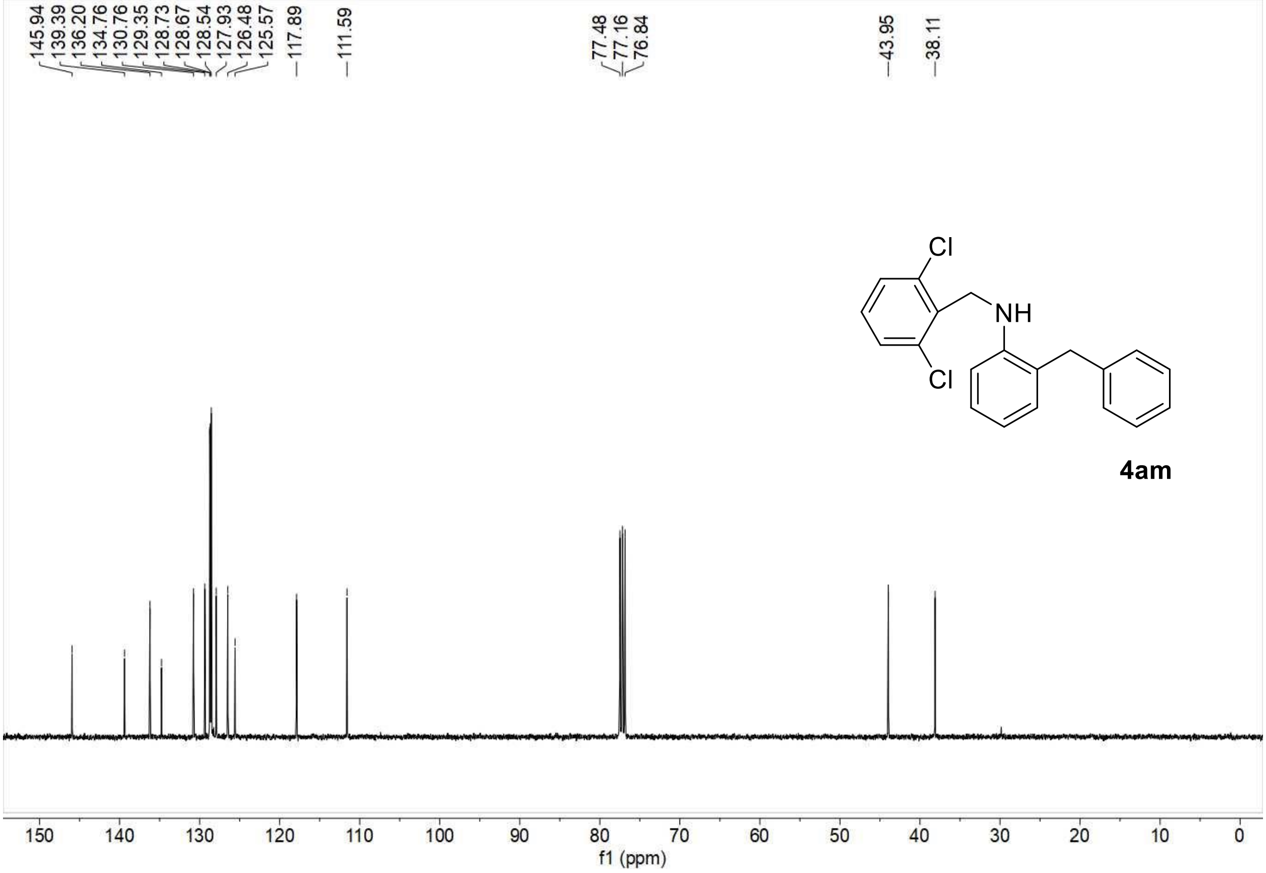
145.94
139.39
136.20
134.76
130.76
129.35
128.73
128.67
128.54
127.93
126.48
125.57
117.89
111.59
77.48
77.16
76.84
43.95
38.11
Cl
NH
Cl
f1 (ppm)

4am

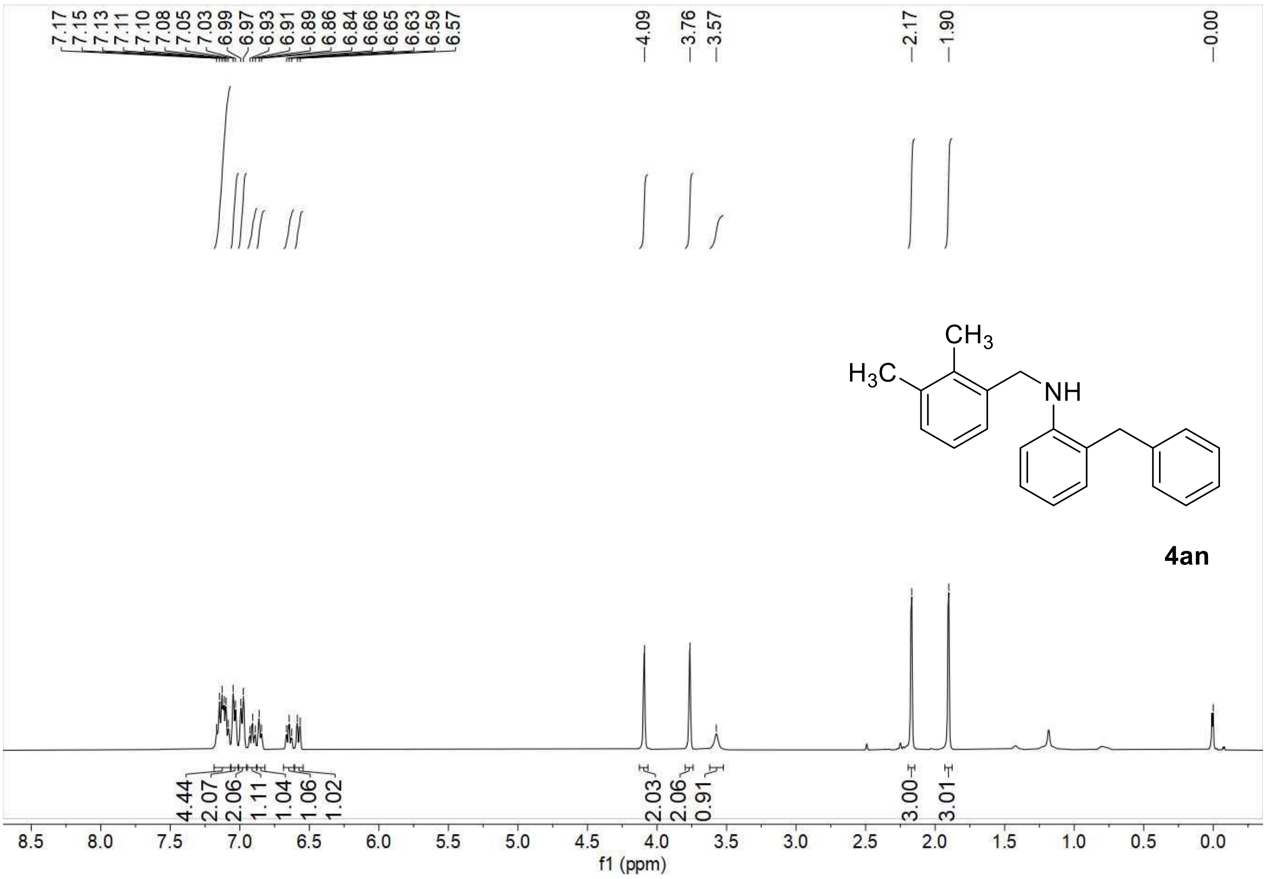
7.17 7.15 7.13 7.11 7.10 7.08 7.05 7.03 6.99 6.97 6.93 6.91 6.89 6.86 6.84 6.66 6.65 6.63 6.59 6.57
4.09 3.76 3.57
2.17 1.90
0.00
CH3
H3C
NH
4an
4.44 2.07 2.06 1.11 1.04 1.06 1.02
2.03 2.06 0.91
3.00 3.01
8.5 8.0 7.5 7.0 6.5 6.0 5.5 5.0 4.5 4.0 3.5 3.0 2.5 2.0 1.5 1.0 0.5 0.0
f1 (ppm)

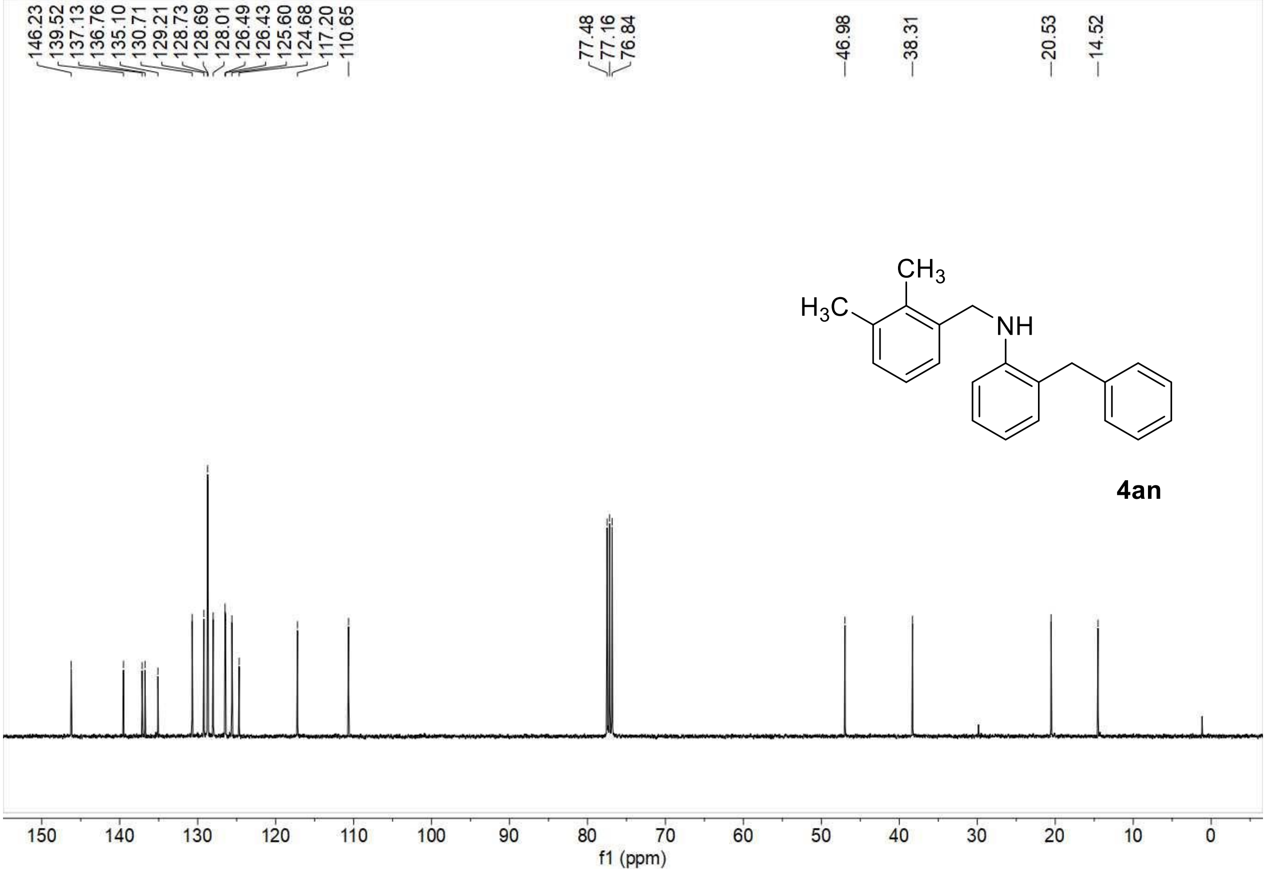
146.23 139.52 137.13 136.76 135.10 130.71 129.21 128.73 128.69 128.01 126.49 126.43 125.60 124.68 117.20 110.65
77.48 77.16 76.84
46.98 38.31
20.53 14.52
CH3
H3C
NH
4an
150 140 130 120 110 100 90 80 70 60 50 40 30 20 10 0
f1 (ppm)

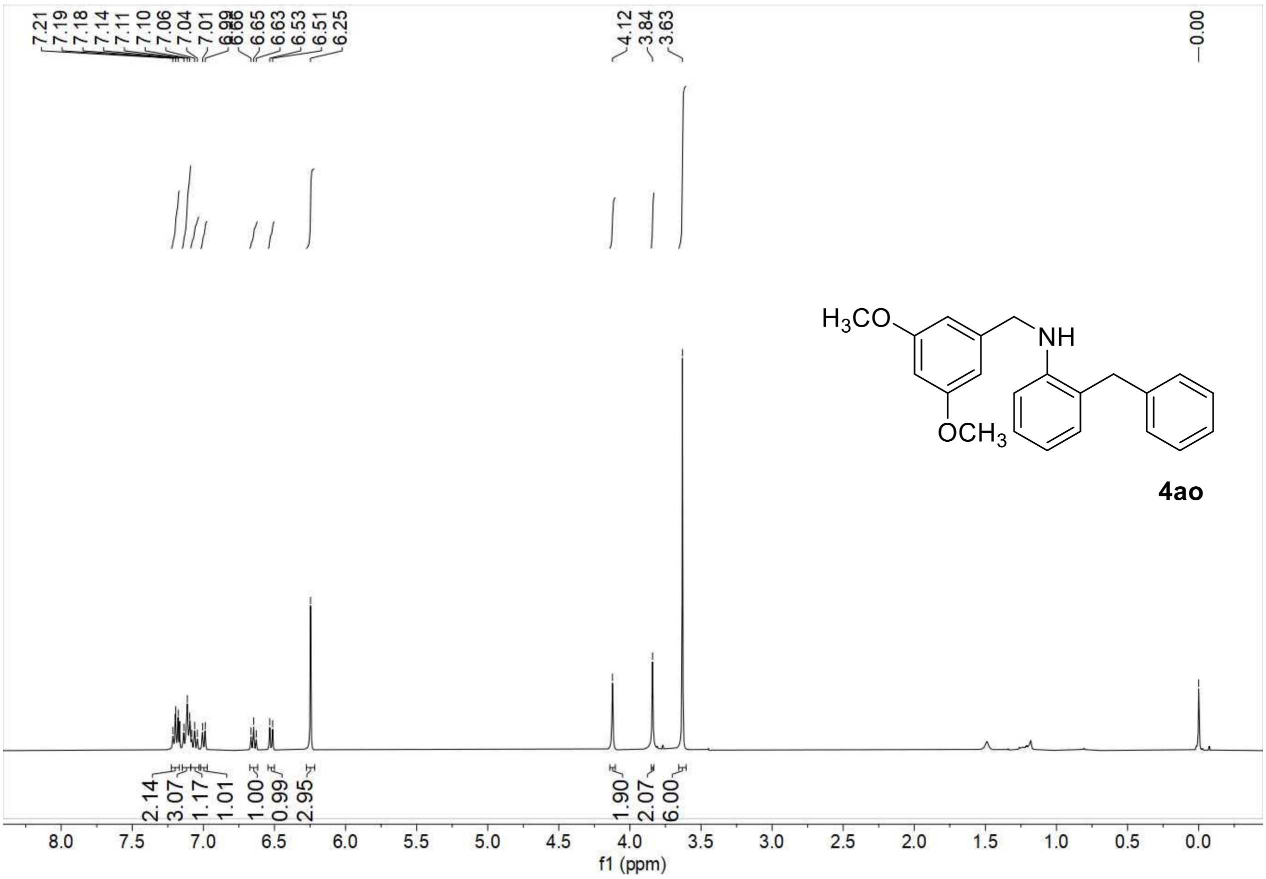
7.21
7.19
7.18
7.14
7.11
7.10
7.06
7.04
7.01
6.65
6.63
6.53
6.51
6.25
4.12
3.84
3.63
0.00
H_3CO
NH
OCH_3
4ao
2.14
3.07
1.17
1.01
1.00
0.99
2.95
1.90
2.07
6.00
f1 (ppm)

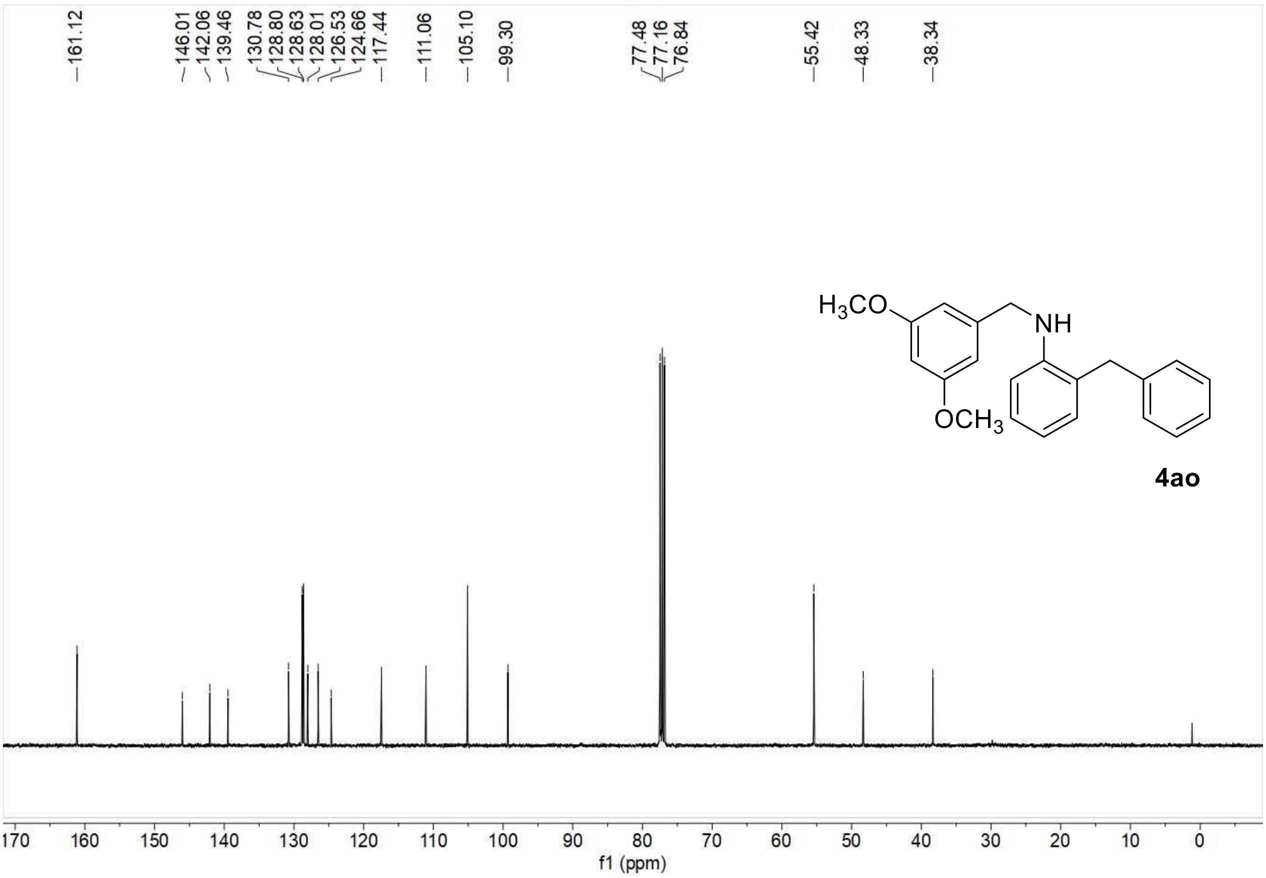
161.12
146.01
142.06
139.46
130.78
128.80
128.63
128.01
126.53
124.66
117.44
111.06
105.10
99.30
77.48
77.16
76.84
55.42
48.33
38.34
H_3CO
NH
OCH_3
4ao
f1 (ppm)

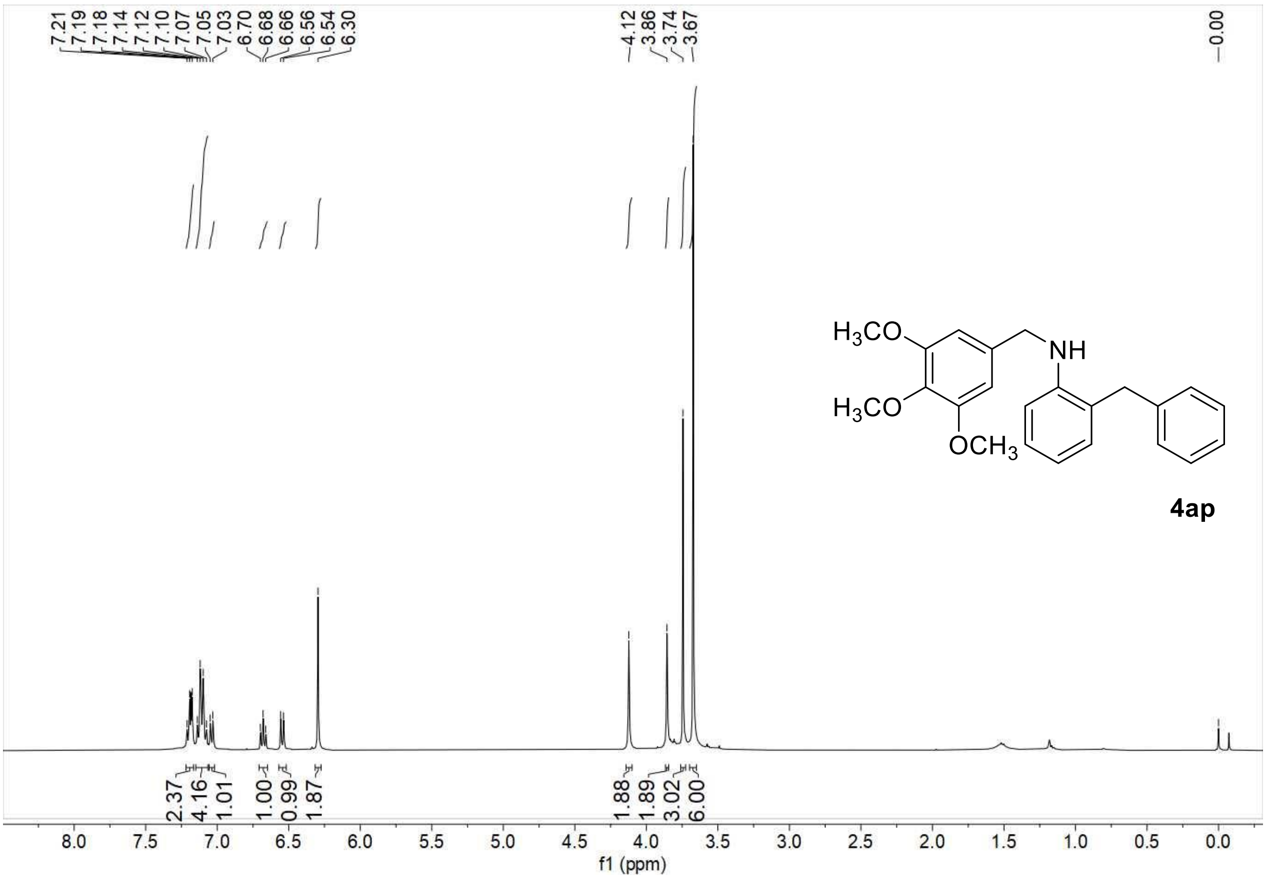
7.21
7.19
7.18
7.14
7.12
7.10
7.07
7.05
7.03
6.70
6.68
6.66
6.56
6.54
6.30
4.12
3.86
3.74
3.67
0.00
H3CO
NH
H3CO
OCH3
4ap
2.37
4.16
1.01
1.00
0.99
1.87
1.88
1.89
3.02
6.00
f1 (ppm)

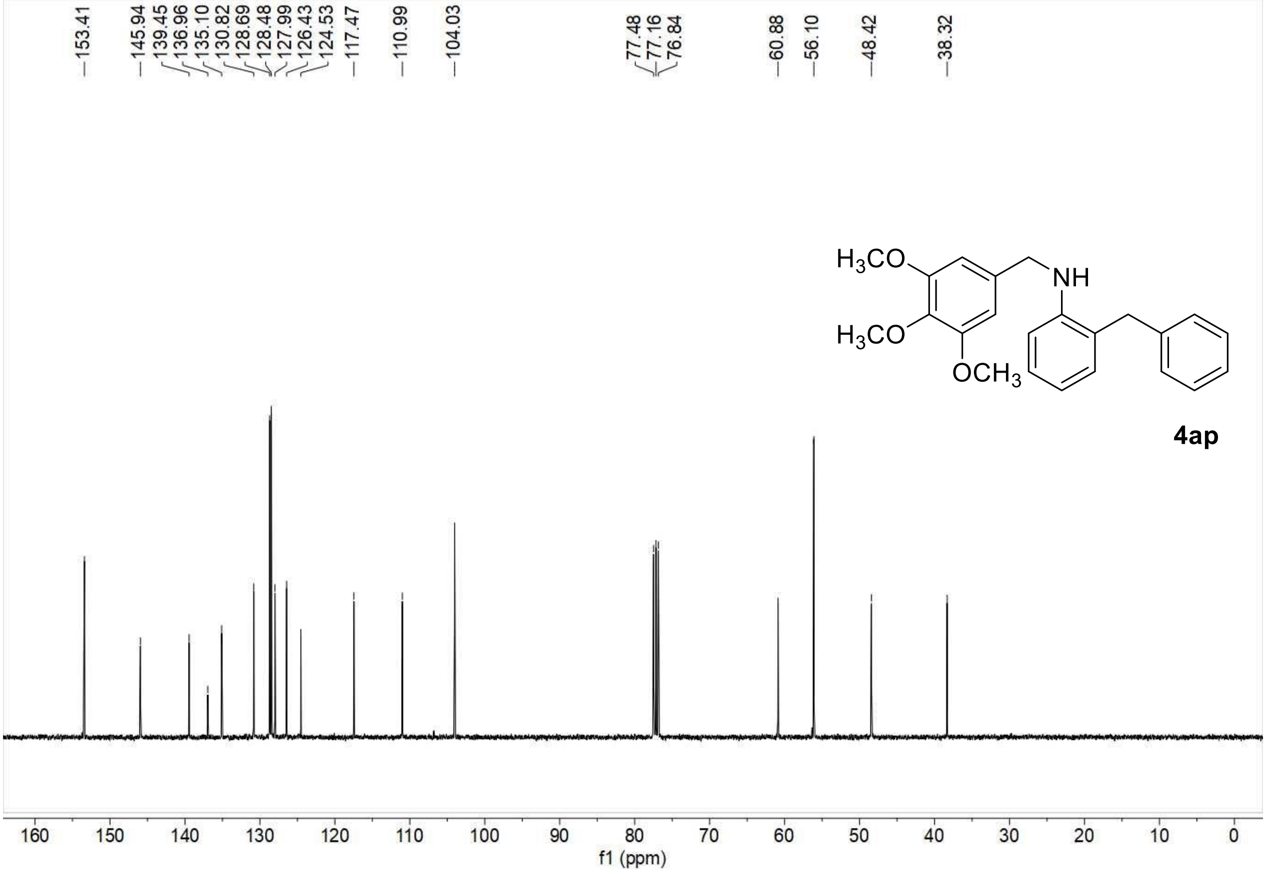
153.41
145.94
139.45
136.96
135.10
130.82
128.69
128.48
127.99
126.43
124.53
117.47
110.99
104.03
77.48
77.16
76.84
60.88
56.10
48.42
38.32
H3CO
NH
H3CO
OCH3
4ap
f1 (ppm)

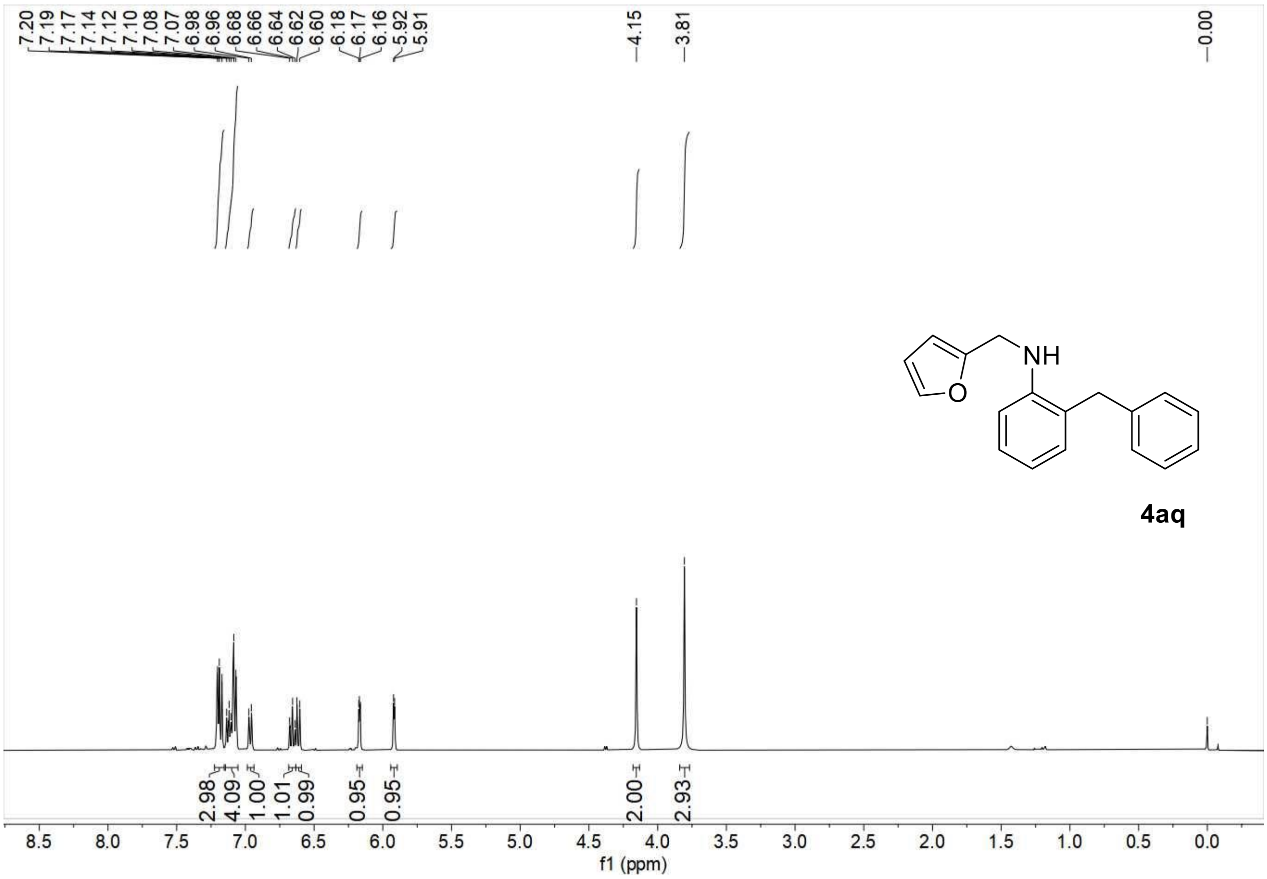
7.20 7.19 7.17 7.14 7.12 7.10 7.08 7.07 6.98 6.96 6.68 6.66 6.64 6.62 6.60 6.18 6.17 6.16 5.92 5.91
4.15
3.81
0.00
NH
O
4aq
2.98 4.09 1.00 1.01 0.99 0.95 0.95 2.00 2.93
8.5 8.0 7.5 7.0 6.5 6.0 5.5 5.0 4.5 4.0 3.5 3.0 2.5 2.0 1.5 1.0 0.5 0.0
f1 (ppm)

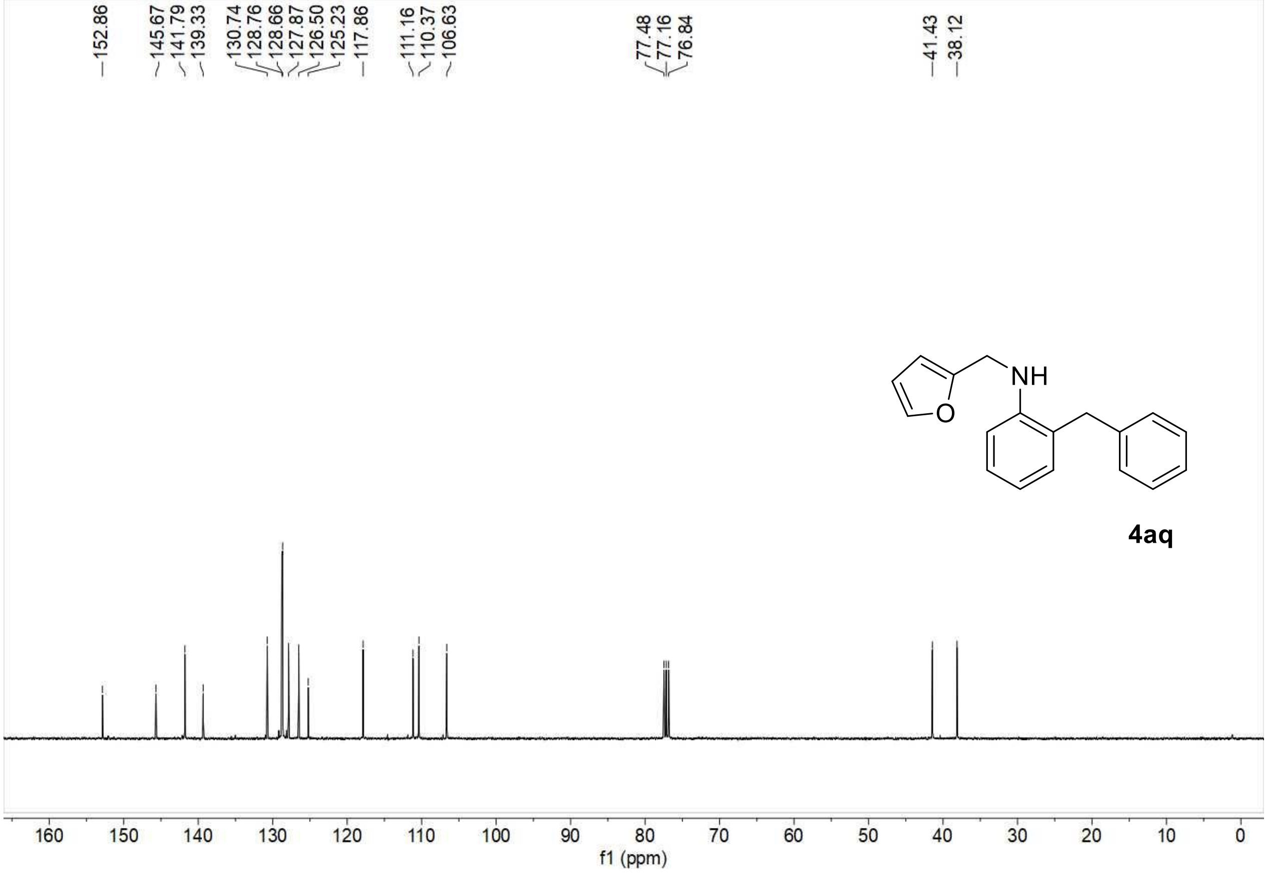
152.86
145.67 141.79 139.33
130.74 128.76 128.66 127.87 126.50 125.23 117.86
111.16 110.37 106.63
77.48 77.16 76.84
41.43 38.12
NH
O
4aq
160 150 140 130 120 110 100 90 80 70 60 50 40 30 20 10 0
f1 (ppm)

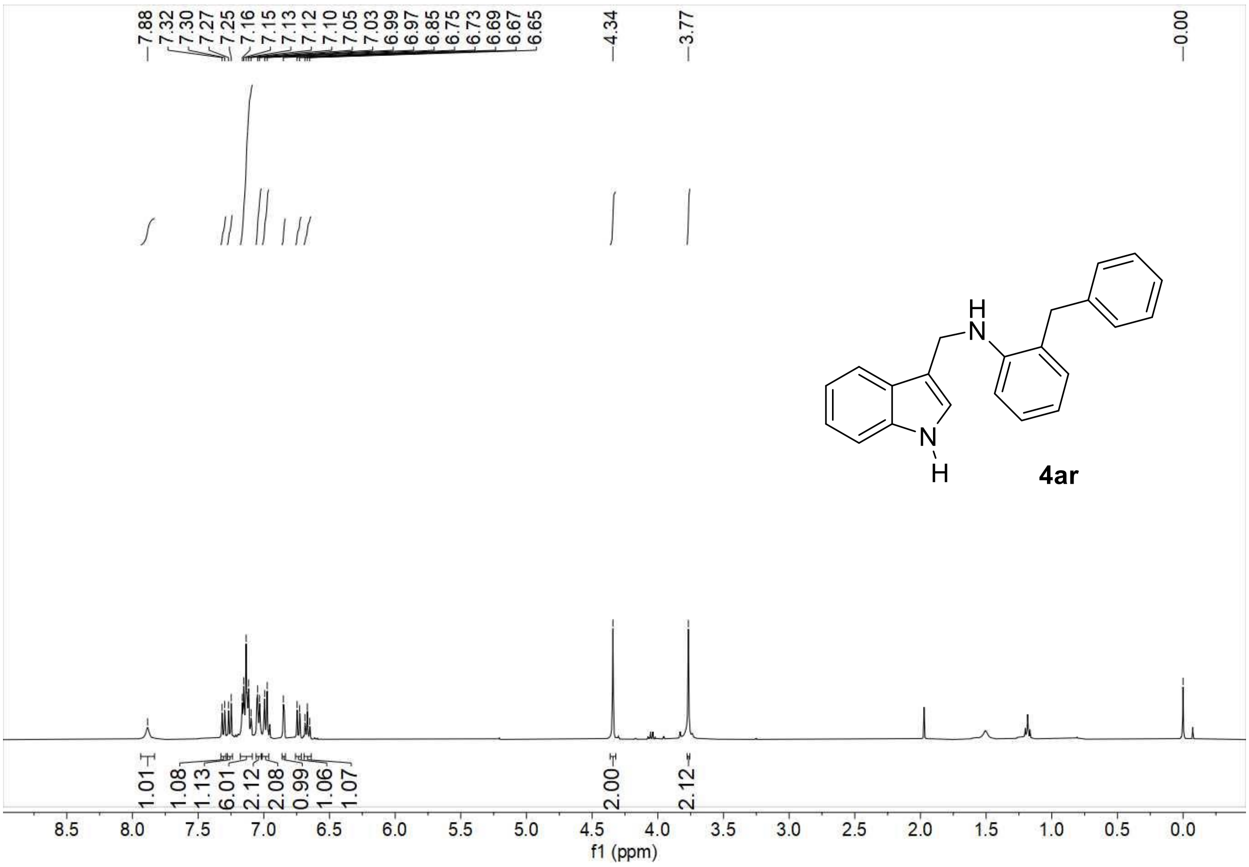

7.88
7.32
7.30
7.27
7.25
7.16
7.15
7.13
7.12
7.10
7.05
7.03
6.99
6.97
6.85
6.75
6.73
6.69
6.67
6.65
4.34
3.77
0.00
4ar
1.01
1.08
1.13
6.01
2.12
2.08
0.99
1.06
1.07
2.00
2.12
f1 (ppm)

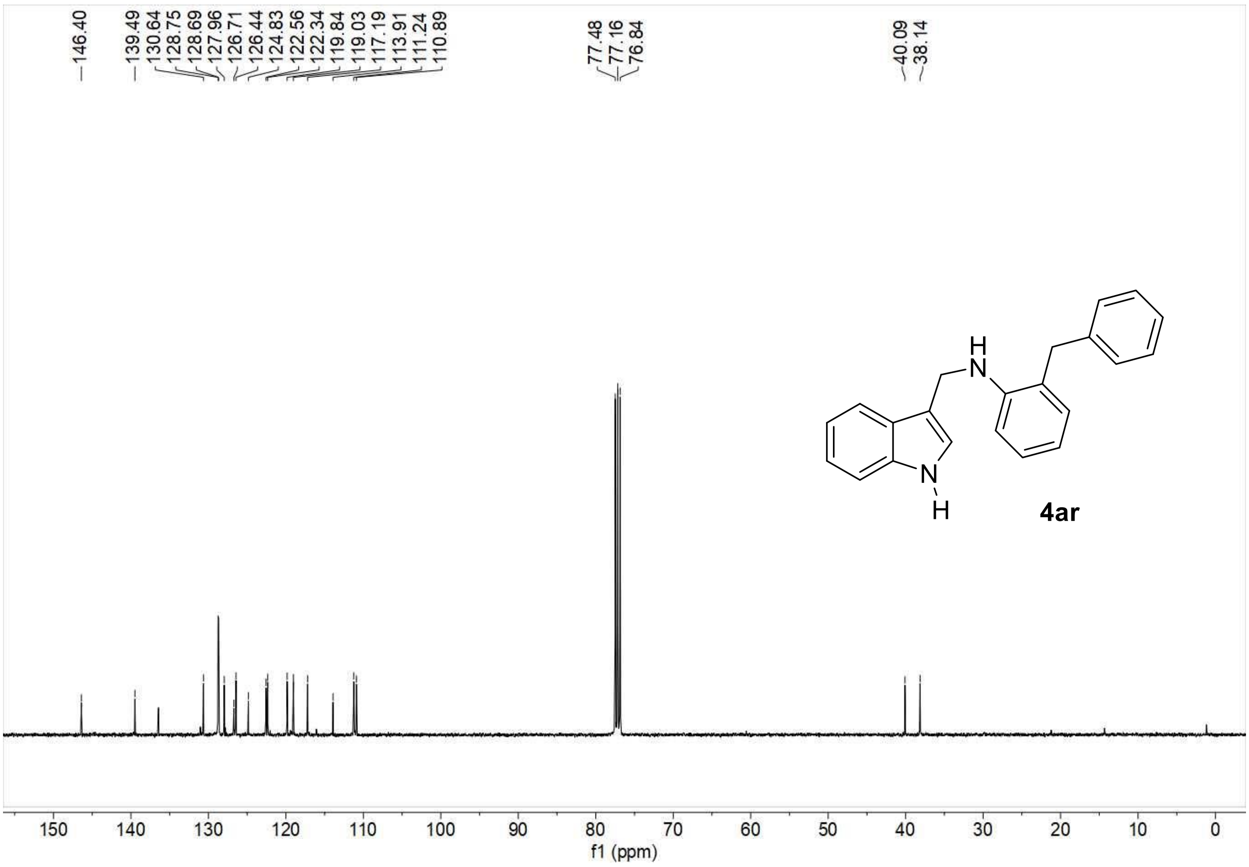

146.40
139.49
130.64
128.75
128.69
127.96
126.71
126.44
124.83
122.56
122.34
119.84
119.03
117.19
113.91
111.24
110.89
77.48
77.16
76.84
40.09
38.14
4ar
f1 (ppm)

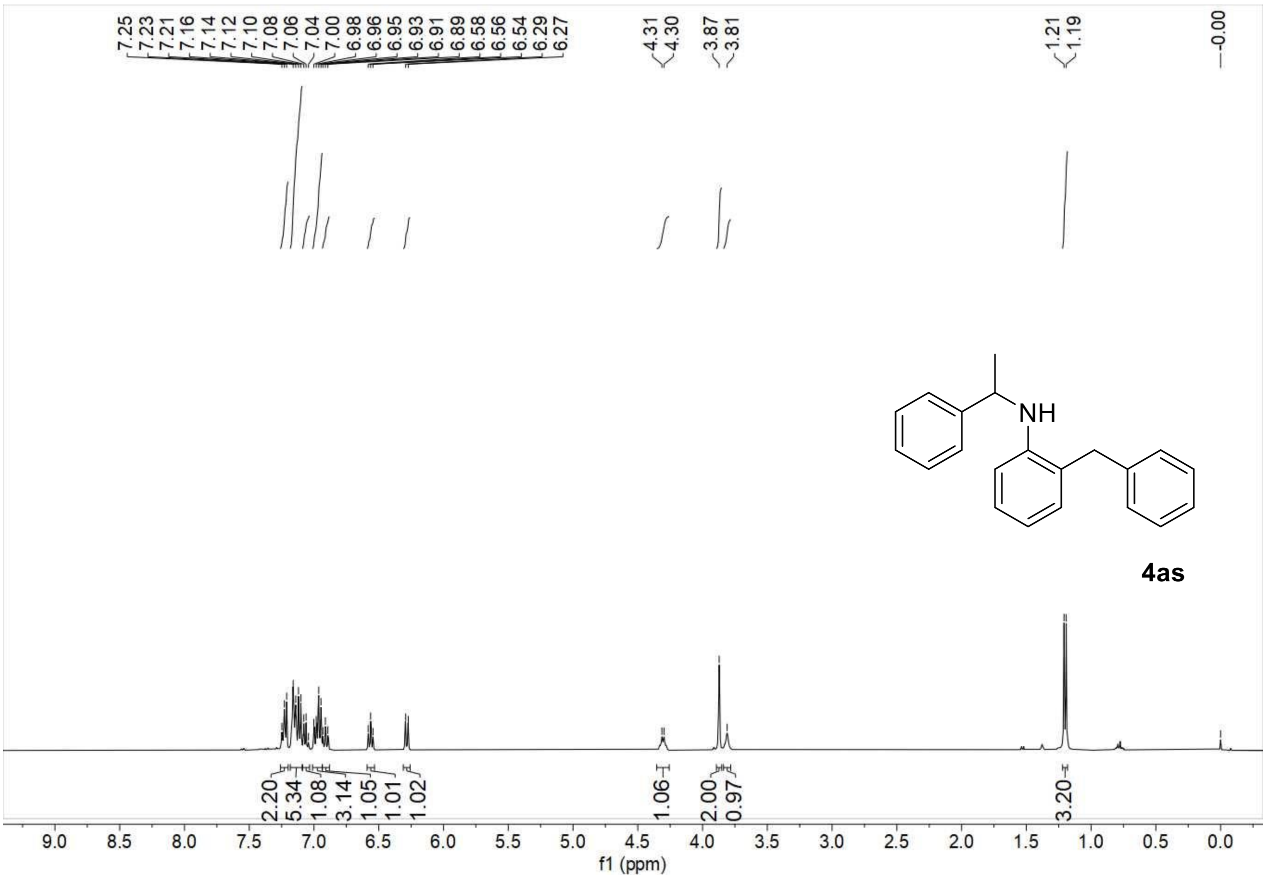
7.25
7.23
7.21
7.16
7.14
7.12
7.10
7.08
7.06
7.04
7.00
6.98
6.96
6.95
6.93
6.91
6.89
6.58
6.56
6.54
6.29
6.27
4.31
4.30
3.87
3.81
1.21
1.19
-0.00
NH
4as
2.20
5.34
1.08
3.14
1.05
1.01
1.02
1.06
2.00
0.97
3.20
f1 (ppm)

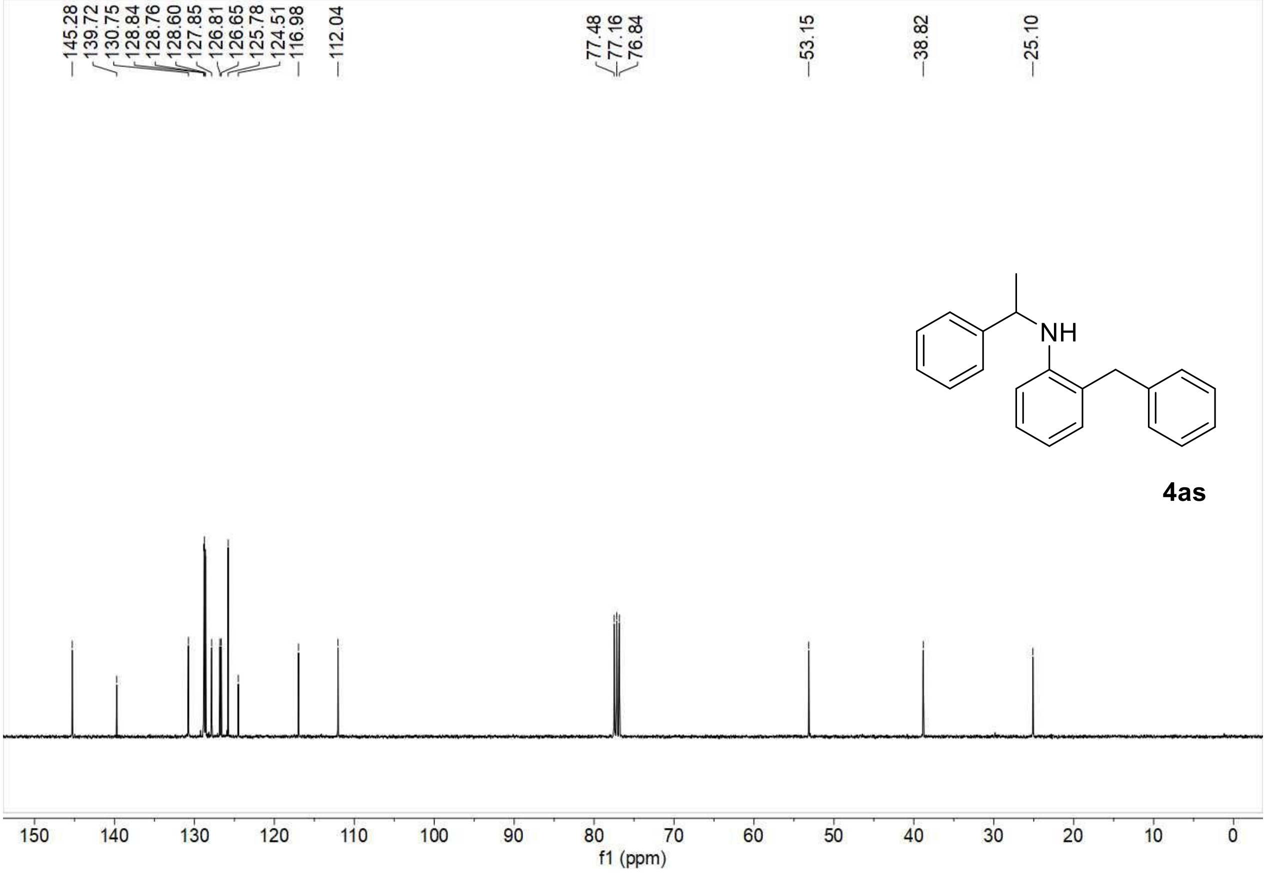
145.28
139.72
130.75
128.84
128.76
128.60
127.85
126.81
126.65
125.78
124.51
116.98
112.04
77.48
77.16
76.84
53.15
38.82
25.10
NH
4as
f1 (ppm)

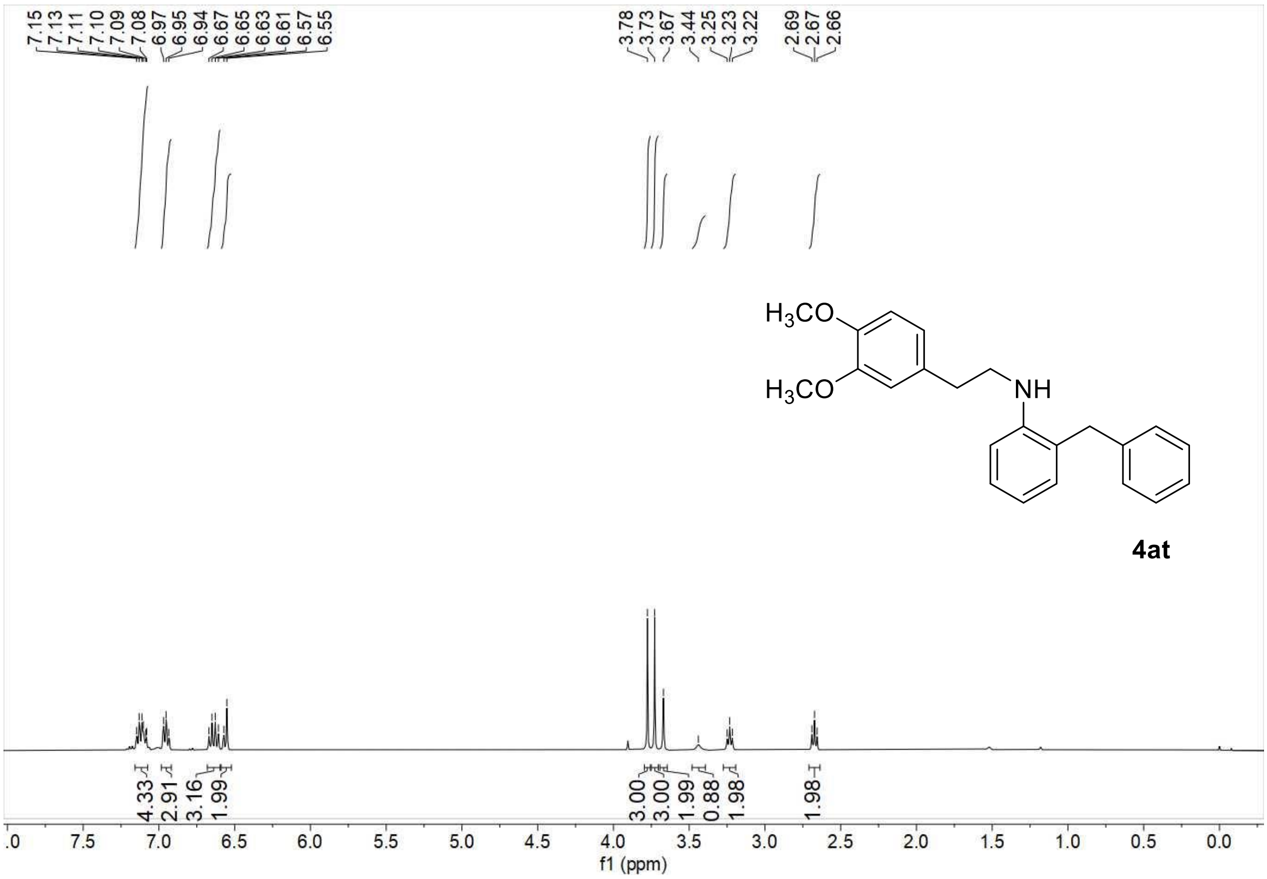
7.15 7.13 7.11 7.10 7.09 7.08 6.97 6.95 6.94 6.67 6.65 6.63 6.61 6.57 6.55
3.78 3.73 3.67 3.44 3.25 3.23 3.22
2.69 2.67 2.66
H_3CO
H_3CO
NH
4at
4.33 2.91 3.16 1.99
3.00 3.00 1.99 0.88 1.98
1.98
.0 7.5 7.0 6.5 6.0 5.5 5.0 4.5 4.0 3.5 3.0 2.5 2.0 1.5 1.0 0.5 0.0
f1 (ppm)

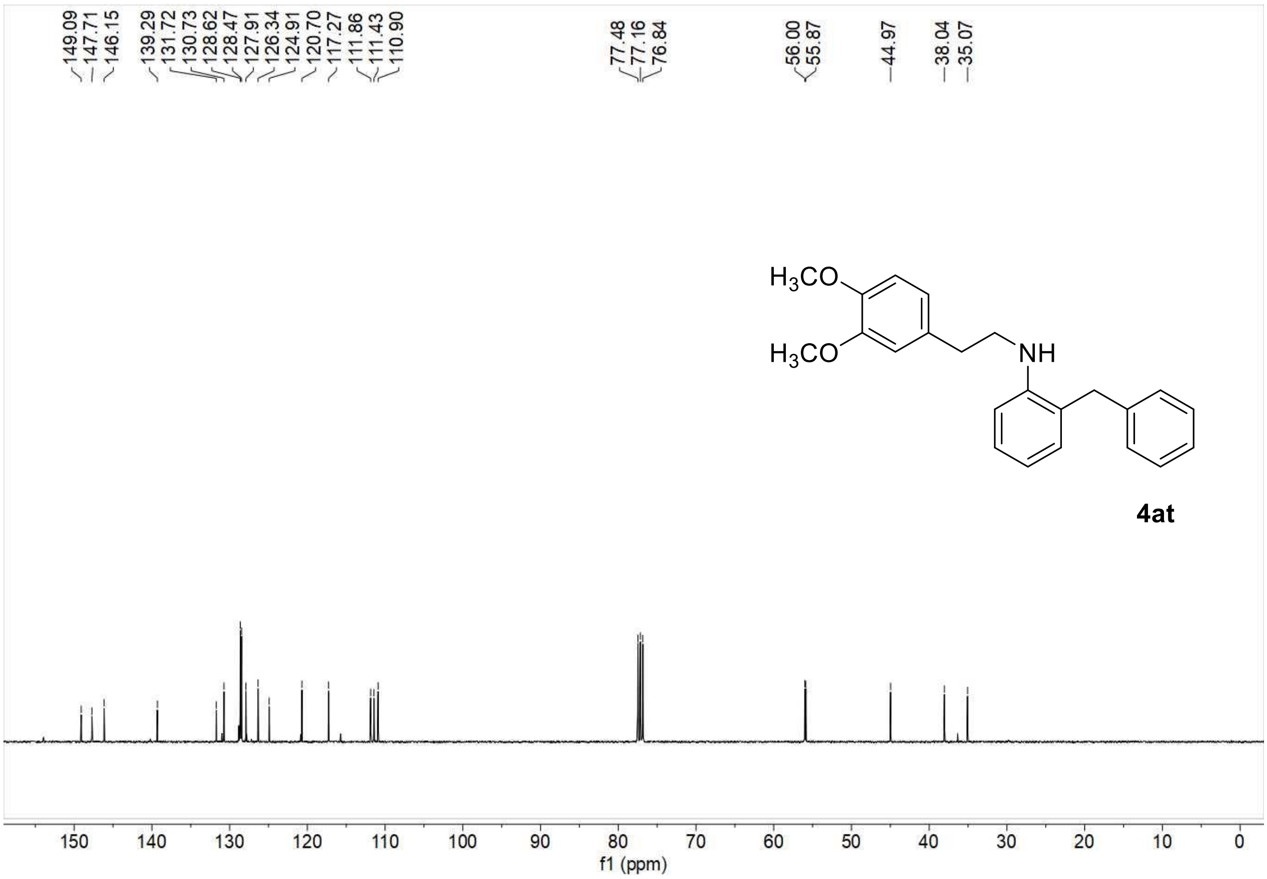
149.09 147.71 146.15 139.29 131.72 130.73 128.62 128.47 127.91 126.34 124.91 120.70 117.27 111.86 111.43 110.90
77.48 77.16 76.84
56.00 55.87
44.97
38.04 35.07
H_3CO
H_3CO
NH
4at
150 140 130 120 110 100 90 80 70 60 50 40 30 20 10 0
f1 (ppm)

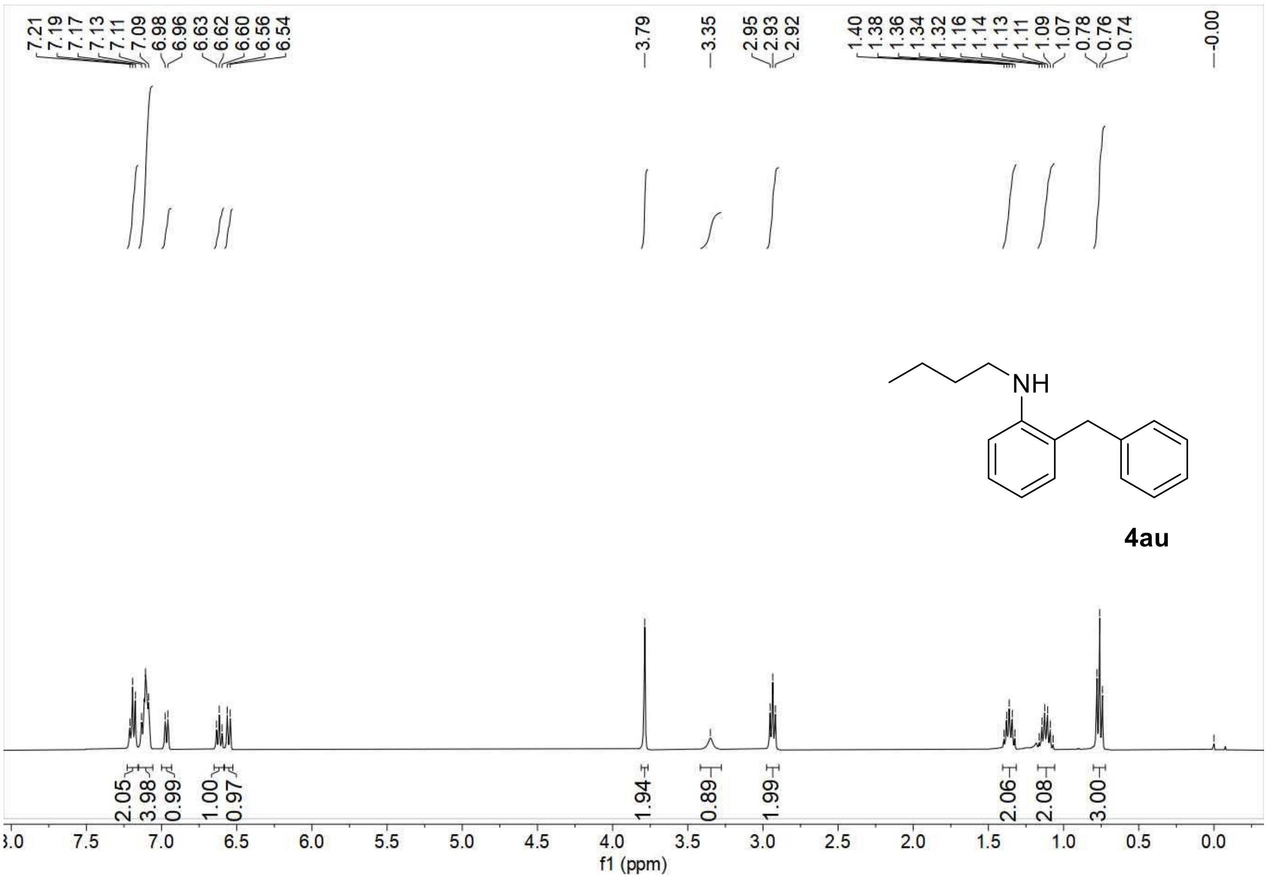
7.21 7.19 7.17 7.13 7.11 7.09 6.98 6.96 6.63 6.62 6.60 6.56 6.54
3.79
3.35
2.95 2.93 2.92
1.40 1.38 1.36 1.34 1.32 1.16 1.14 1.13 1.11 1.09 1.07 0.78 0.76 0.74
-0.00
NH
4au
2.05 3.98 0.99 1.00 0.97 1.94 0.89 1.99 2.06 2.08 3.00
f1 (ppm)

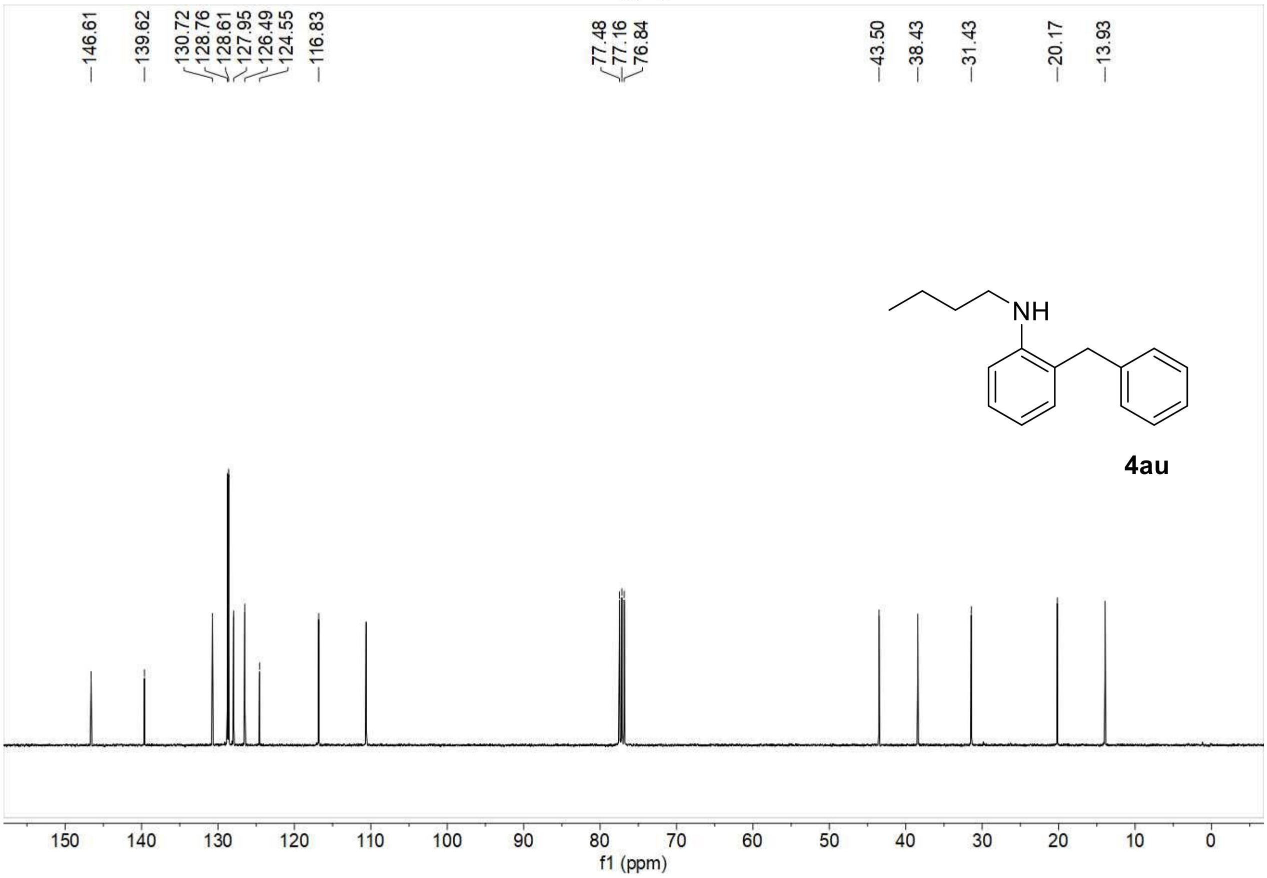
146.61
139.62
130.72 128.76 128.61 127.95 126.49 124.55
116.83
77.48 77.16 76.84
43.50
38.43
31.43
20.17
13.93
NH
4au
f1 (ppm)

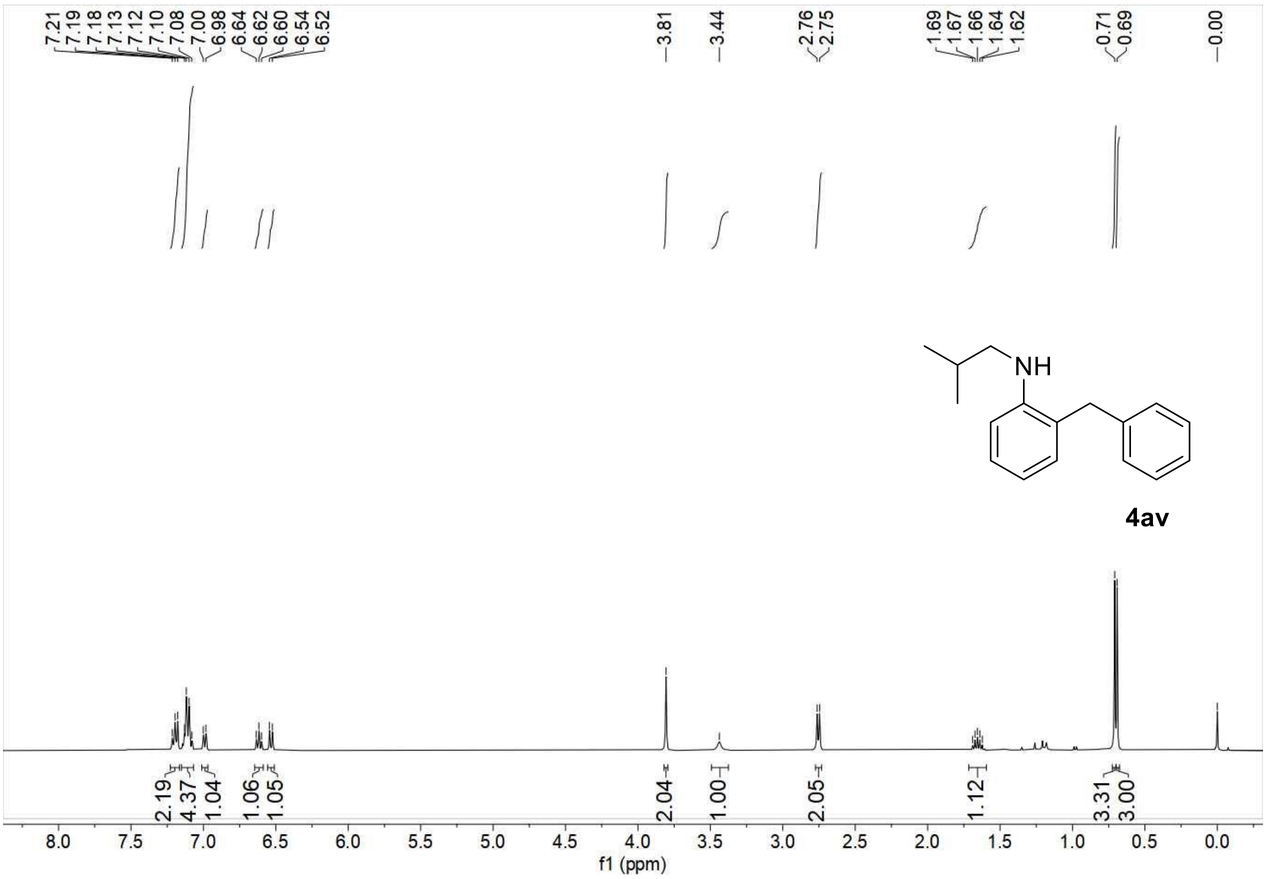
7.21 7.19 7.18 7.13 7.12 7.10 7.08 7.00 6.98 6.64 6.62 6.60 6.54 6.52
3.81
3.44
2.76 2.75
1.69 1.67 1.66 1.64 1.62
0.71 0.69
0.00
NH
4av
2.19 4.37 1.04 1.06 1.05
2.04
1.00
2.05
1.12
3.31 3.00
8.0 7.5 7.0 6.5 6.0 5.5 5.0 4.5 4.0 3.5 3.0 2.5 2.0 1.5 1.0 0.5 0.0
f1 (ppm)

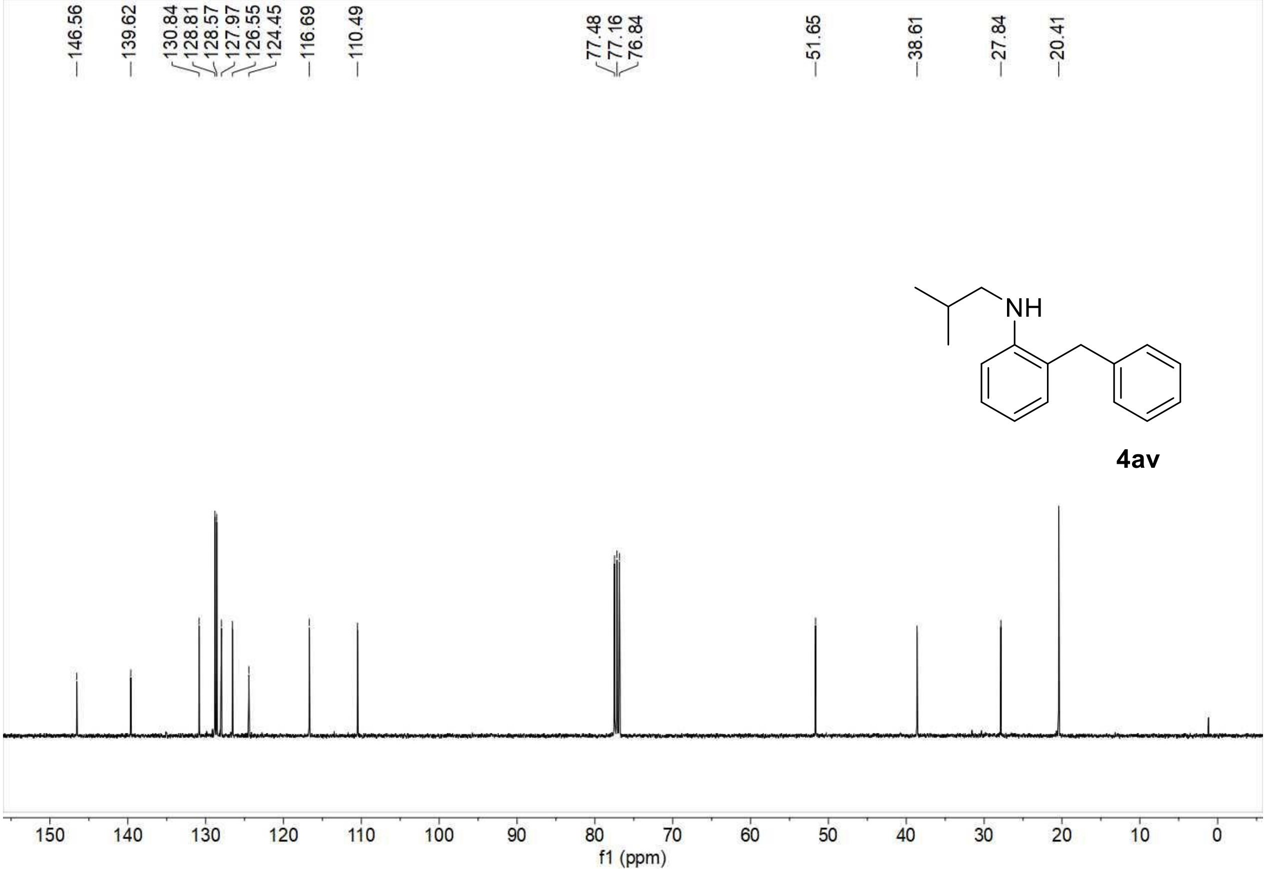
146.56
139.62
130.84 128.81 128.57 127.97 126.55 124.45
116.69
110.49
77.48 77.16 76.84
51.65
38.61
27.84
20.41
NH
4av
150 140 130 120 110 100 90 80 70 60 50 40 30 20 10 0
f1 (ppm)

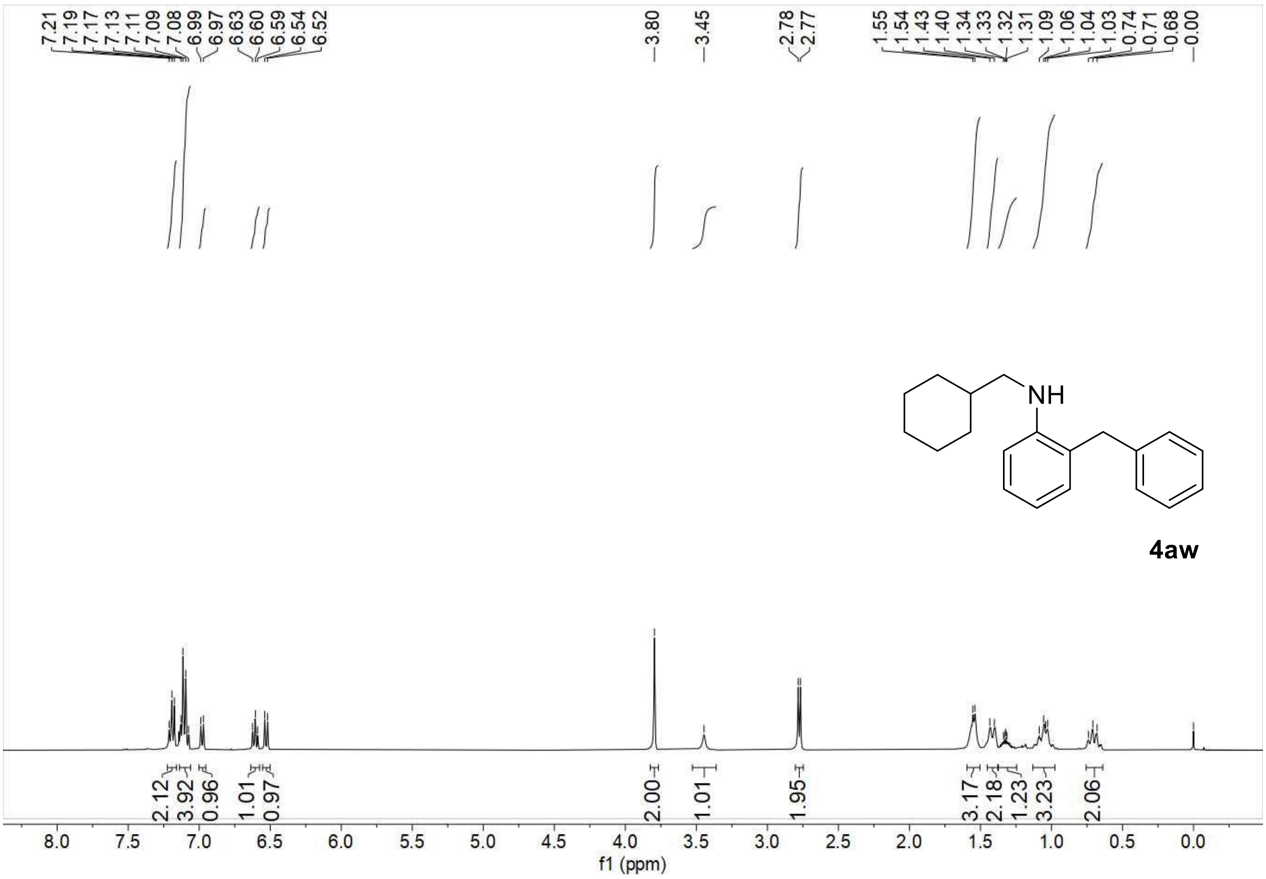

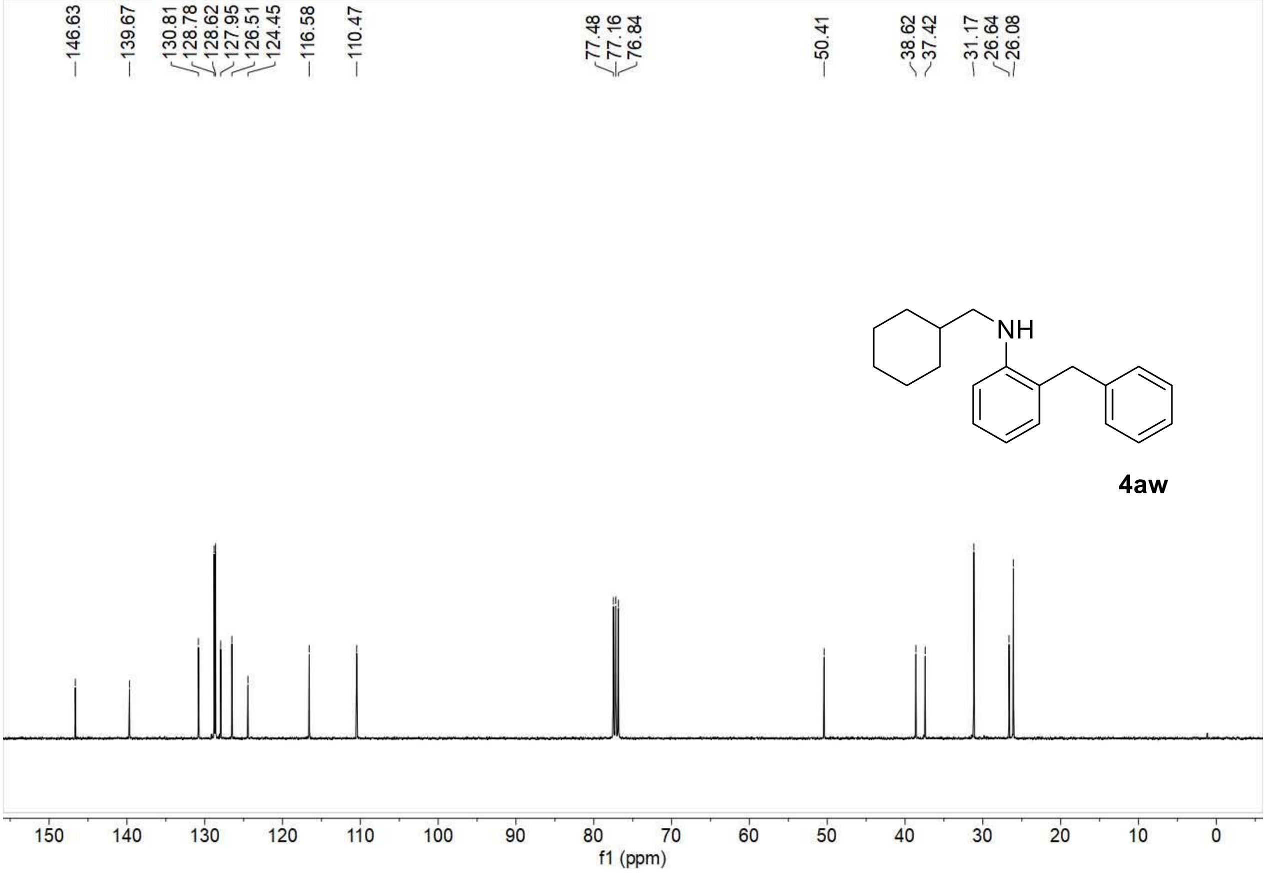

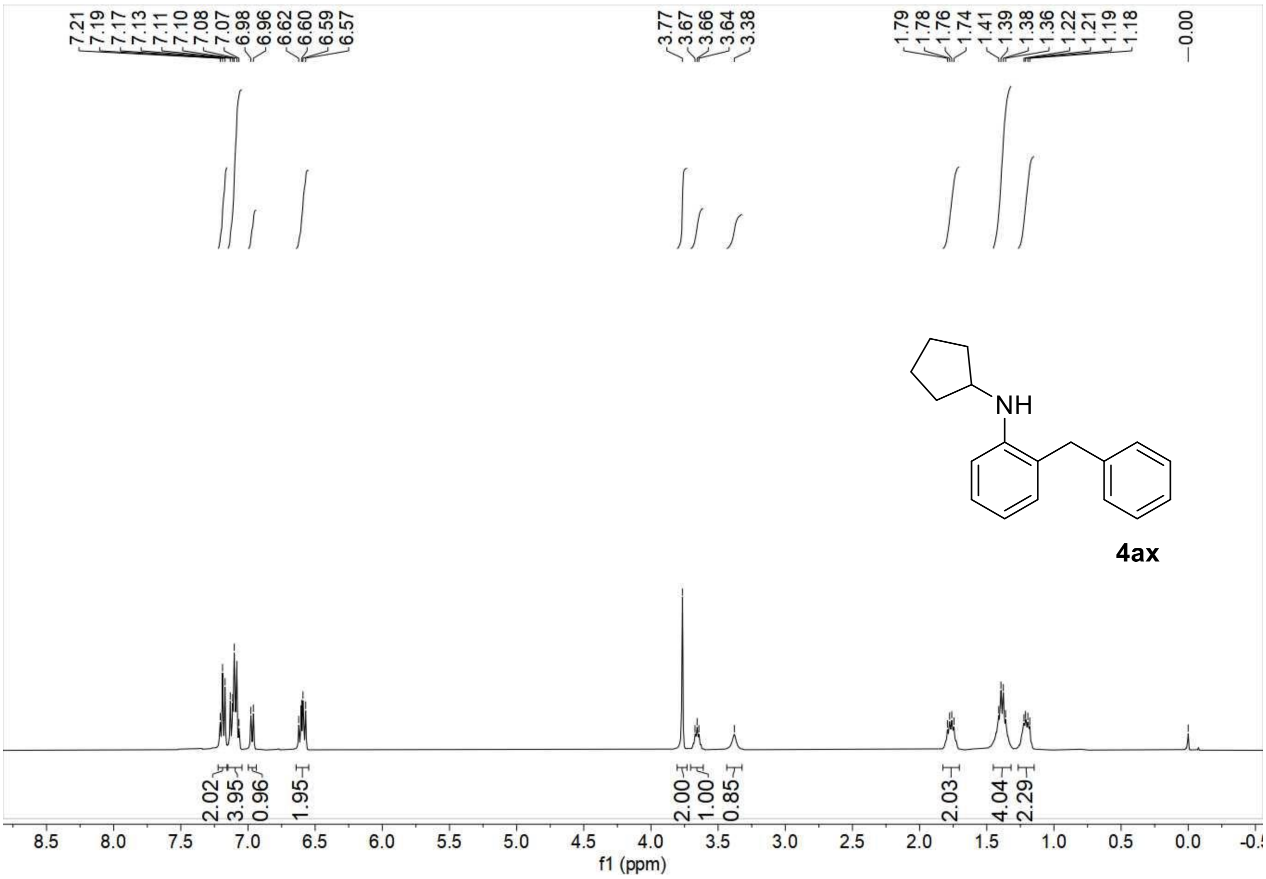
7.21
7.19
7.17
7.13
7.11
7.10
7.08
7.07
6.98
6.96
6.62
6.60
6.59
6.57
3.77
3.67
3.66
3.64
3.38
1.79
1.78
1.76
1.74
1.41
1.39
1.38
1.36
1.22
1.21
1.19
1.18
0.00
NH
4ax
2.02
3.95
0.96
1.95
2.00
1.00
0.85
2.03
4.04
2.29
f1 (ppm)

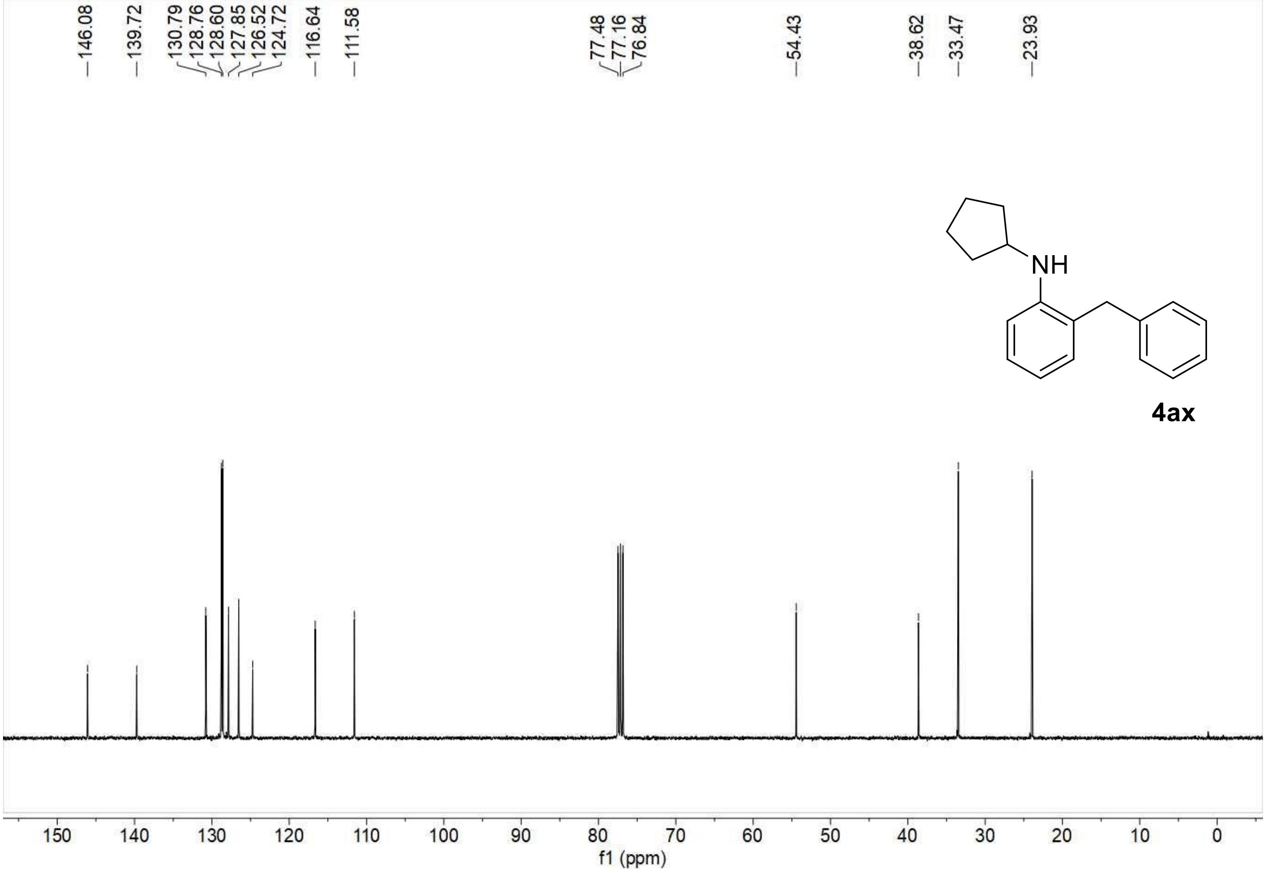
146.08
139.72
130.79
128.76
128.60
127.85
126.52
124.72
116.64
111.58
77.48
77.16
76.84
54.43
38.62
33.47
23.93
NH
4ax
f1 (ppm)

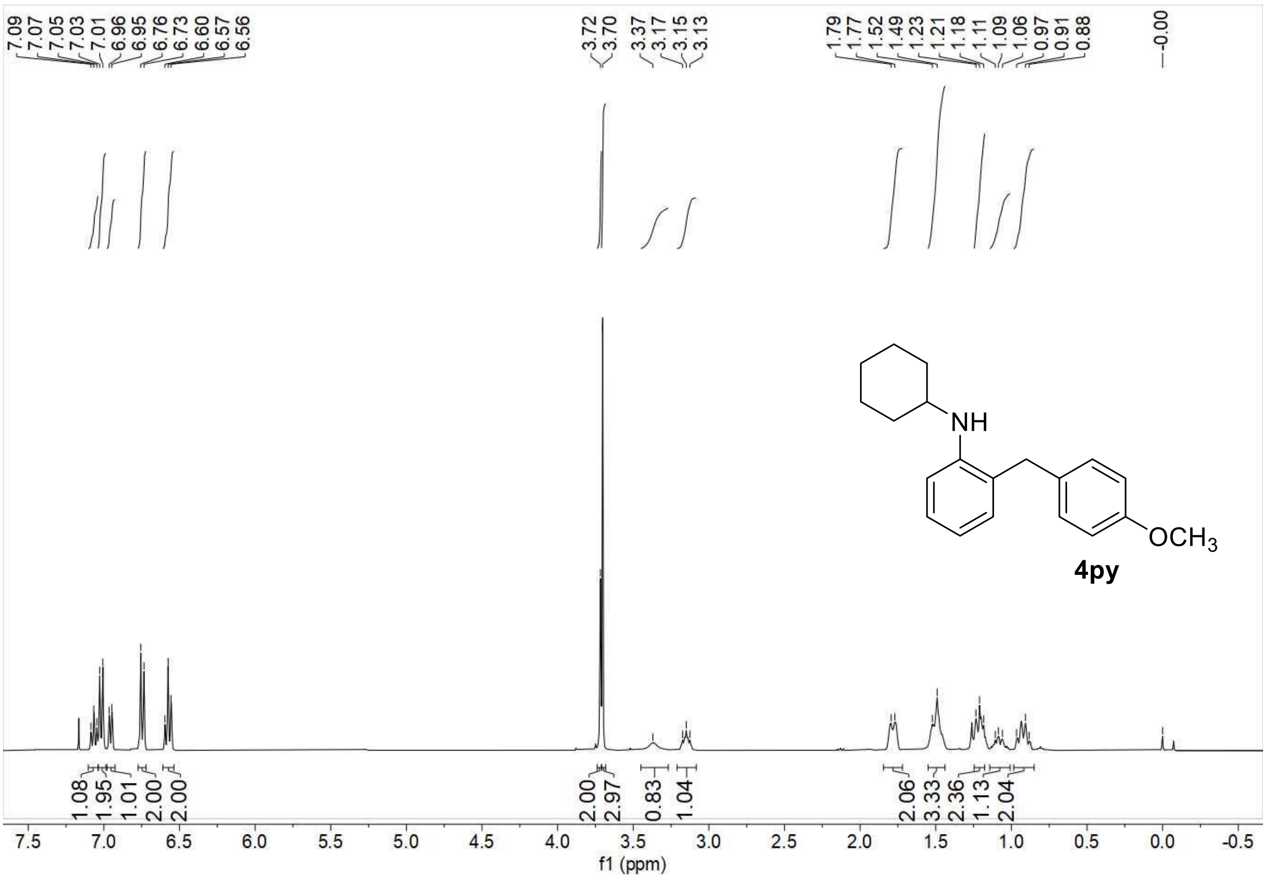
7.09 7.07 7.05 7.03 7.01 6.96 6.95 6.76 6.73 6.60 6.57 6.56
3.72 3.70 3.37 3.17 3.15 3.13
1.79 1.77 1.52 1.49 1.23 1.21 1.18 1.11 1.09 1.06 0.97 0.91 0.88
-0.00
NH
OCH3
4py
1.08 1.95 1.01 2.00 2.00
2.00 2.97 0.83 1.04
2.06 3.33 2.36 1.13 2.04
f1 (ppm)

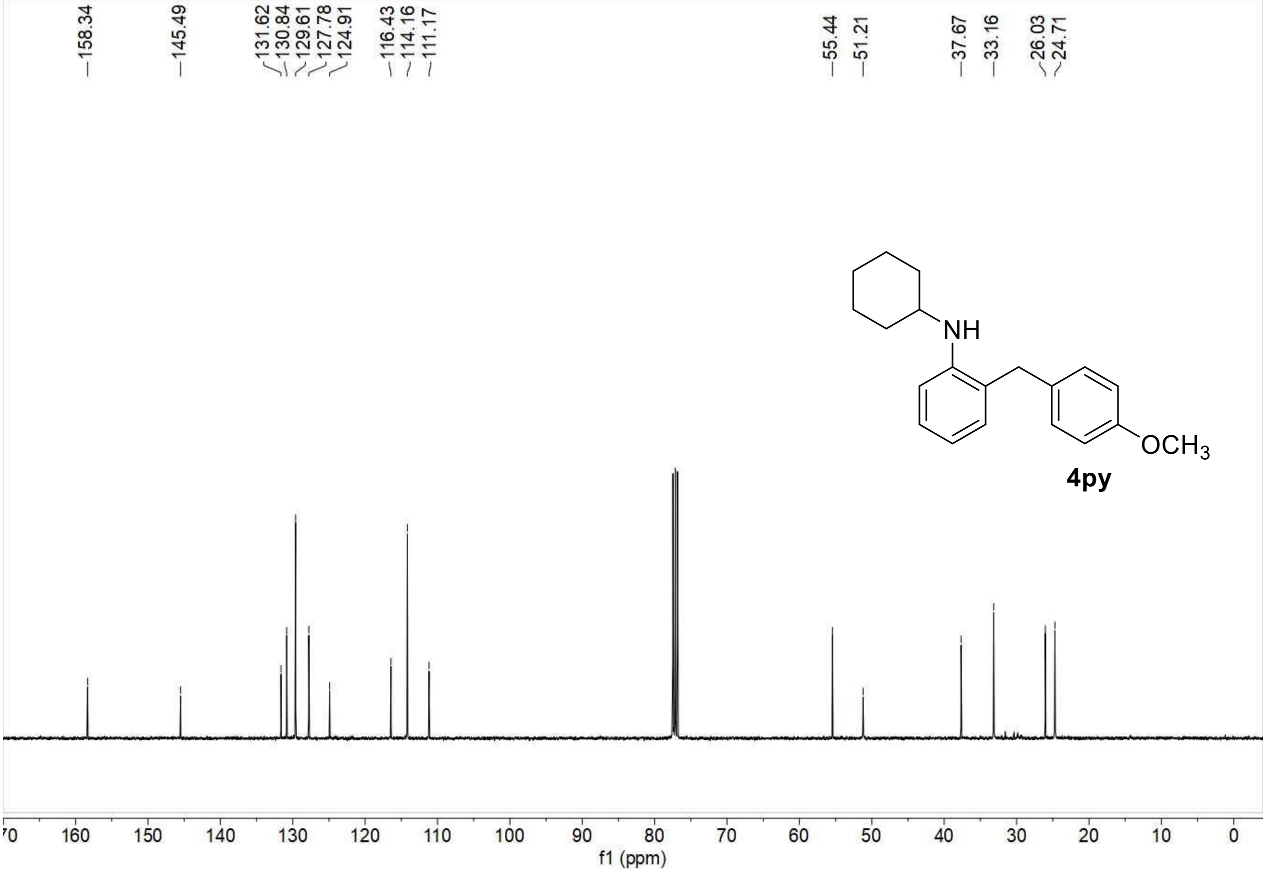
158.34
145.49
131.62 130.84 129.61 127.78 124.91
116.43 114.16 111.17
55.44
51.21
37.67
33.16
26.03 24.71
NH
OCH3
4py
f1 (ppm)

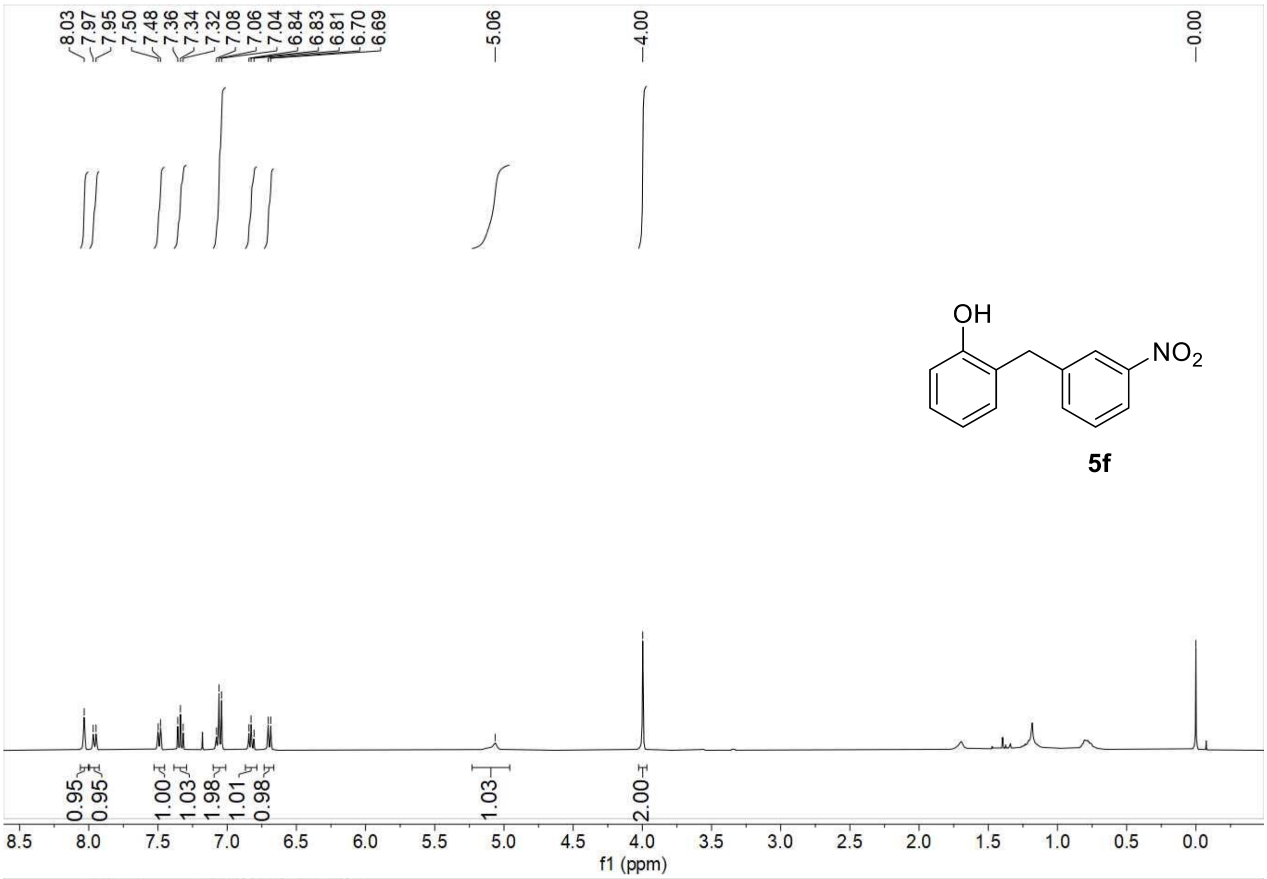
8.03
7.97
7.95
7.50
7.48
7.36
7.34
7.32
7.08
7.06
7.04
6.84
6.83
6.81
6.70
6.69
5.06
4.00
0.00
OH
NO2
5f
0.95
0.95
1.00
1.03
1.98
1.01
0.98
1.03
2.00
8.5 8.0 7.5 7.0 6.5 6.0 5.5 5.0 4.5 4.0 3.5 3.0 2.5 2.0 1.5 1.0 0.5 0.0
f1 (ppm)

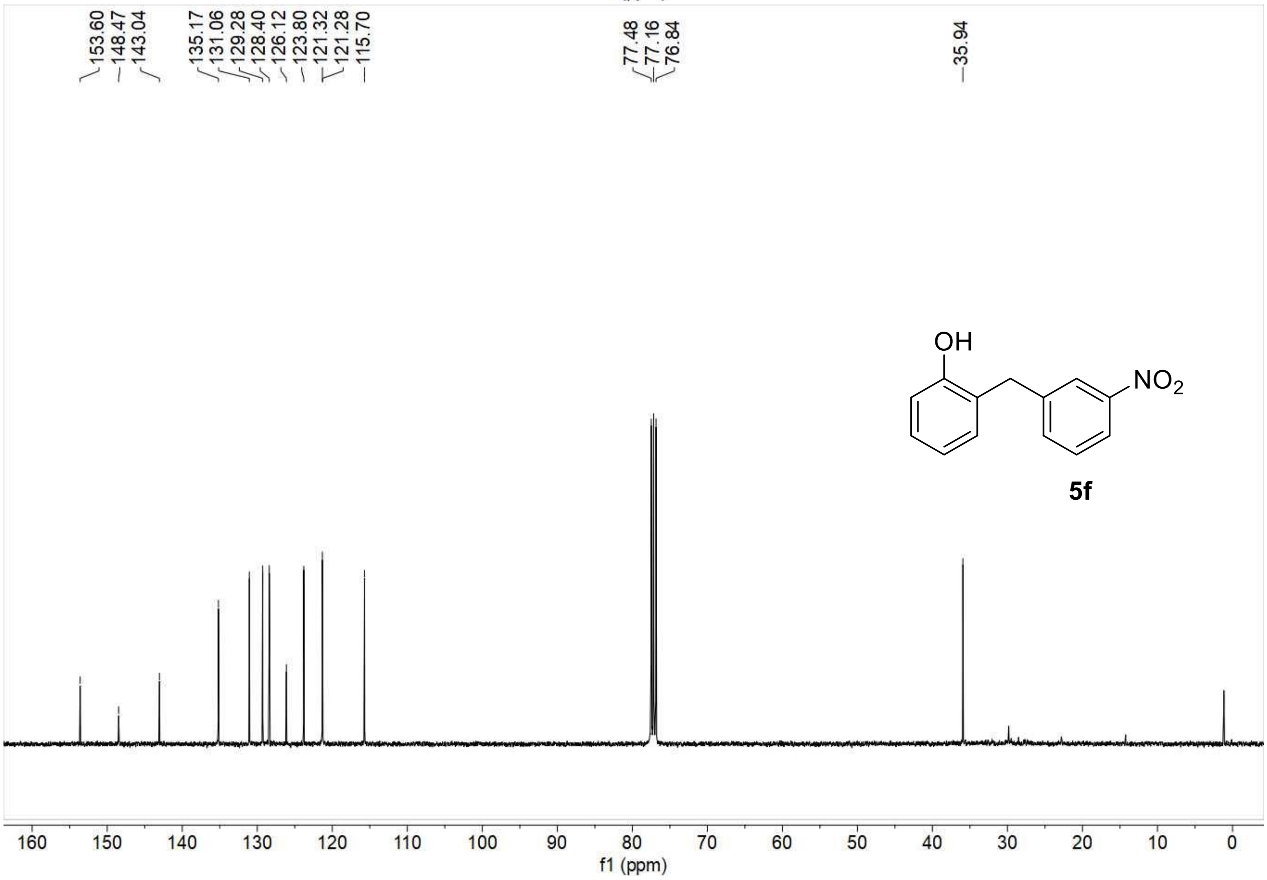
153.60
148.47
143.04
135.17
131.06
129.28
128.40
126.12
123.80
121.32
121.28
115.70
77.48
77.16
76.84
35.94
OH
NO2
5f
160 150 140 130 120 110 100 90 80 70 60 50 40 30 20 10 0
f1 (ppm)

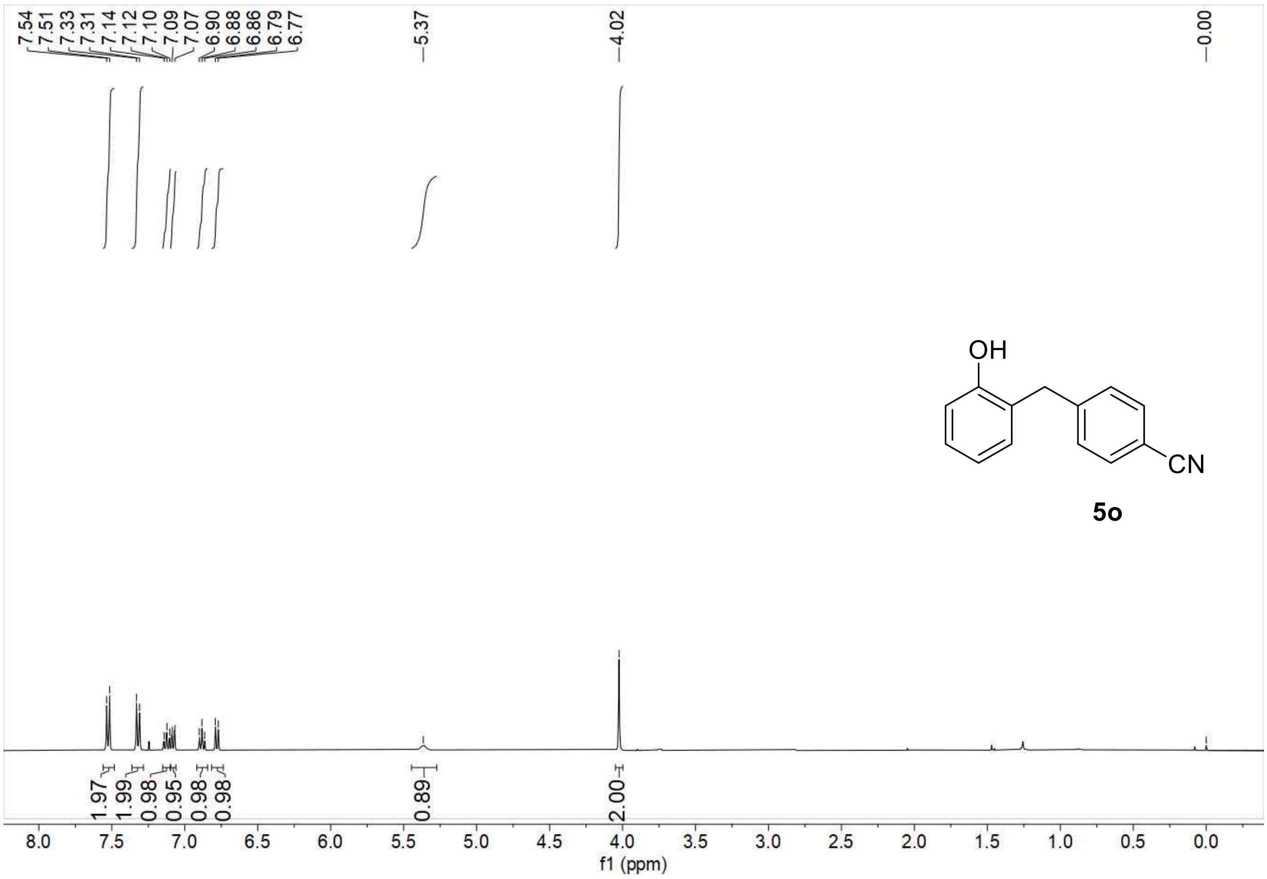
7.54
7.51
7.33
7.31
7.14
7.12
7.10
7.09
7.07
6.90
6.88
6.86
6.79
6.77
5.37
4.02
0.00
OH
CN
5o
1.97
1.99
0.98
0.95
0.98
0.98
0.89
2.00
8.0 7.5 7.0 6.5 6.0 5.5 5.0 4.5 4.0 3.5 3.0 2.5 2.0 1.5 1.0 0.5 0.0
f1 (ppm)

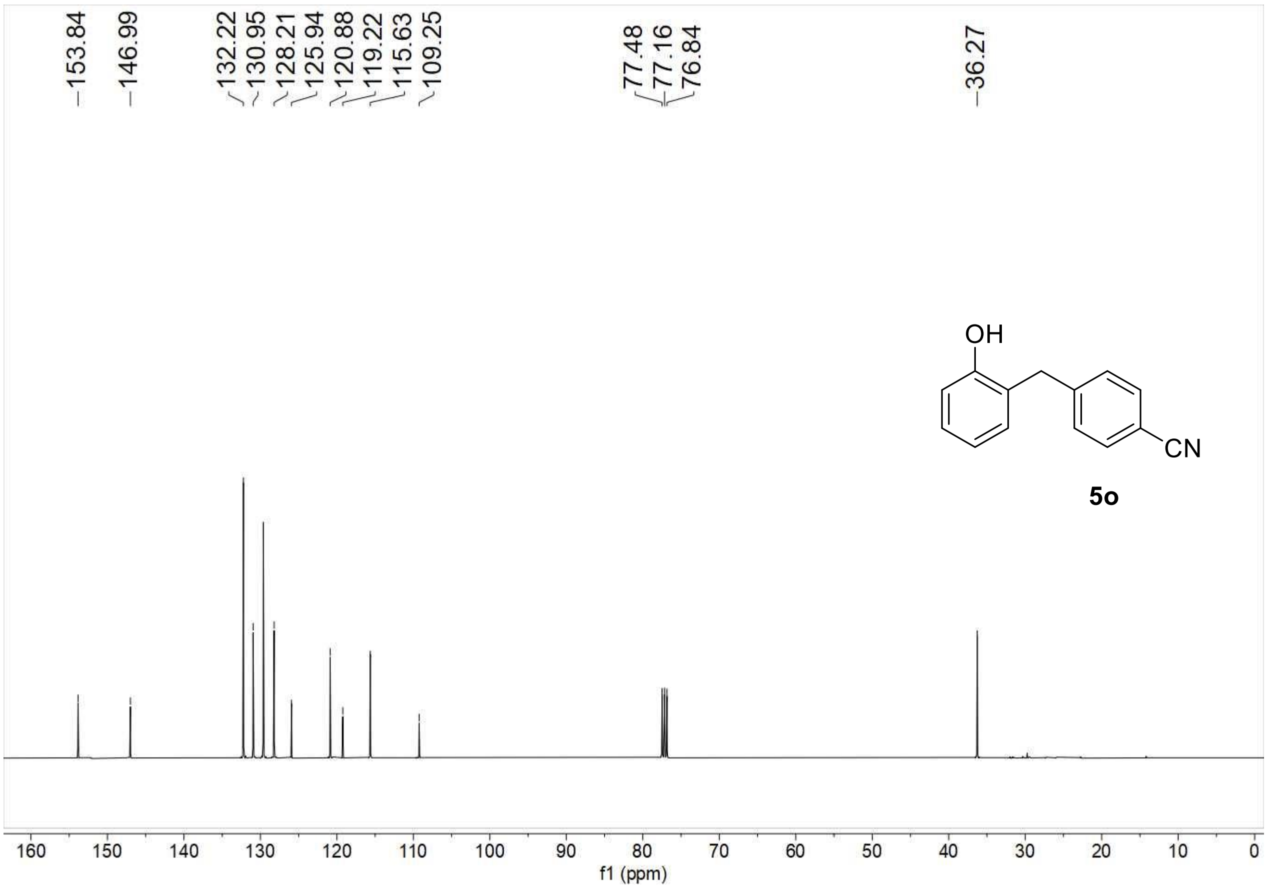
153.84
146.99
132.22
130.95
128.21
125.94
120.88
119.22
115.63
109.25
77.48
77.16
76.84
36.27
OH
CN
5o
160 150 140 130 120 110 100 90 80 70 60 50 40 30 20 10 0
f1 (ppm)

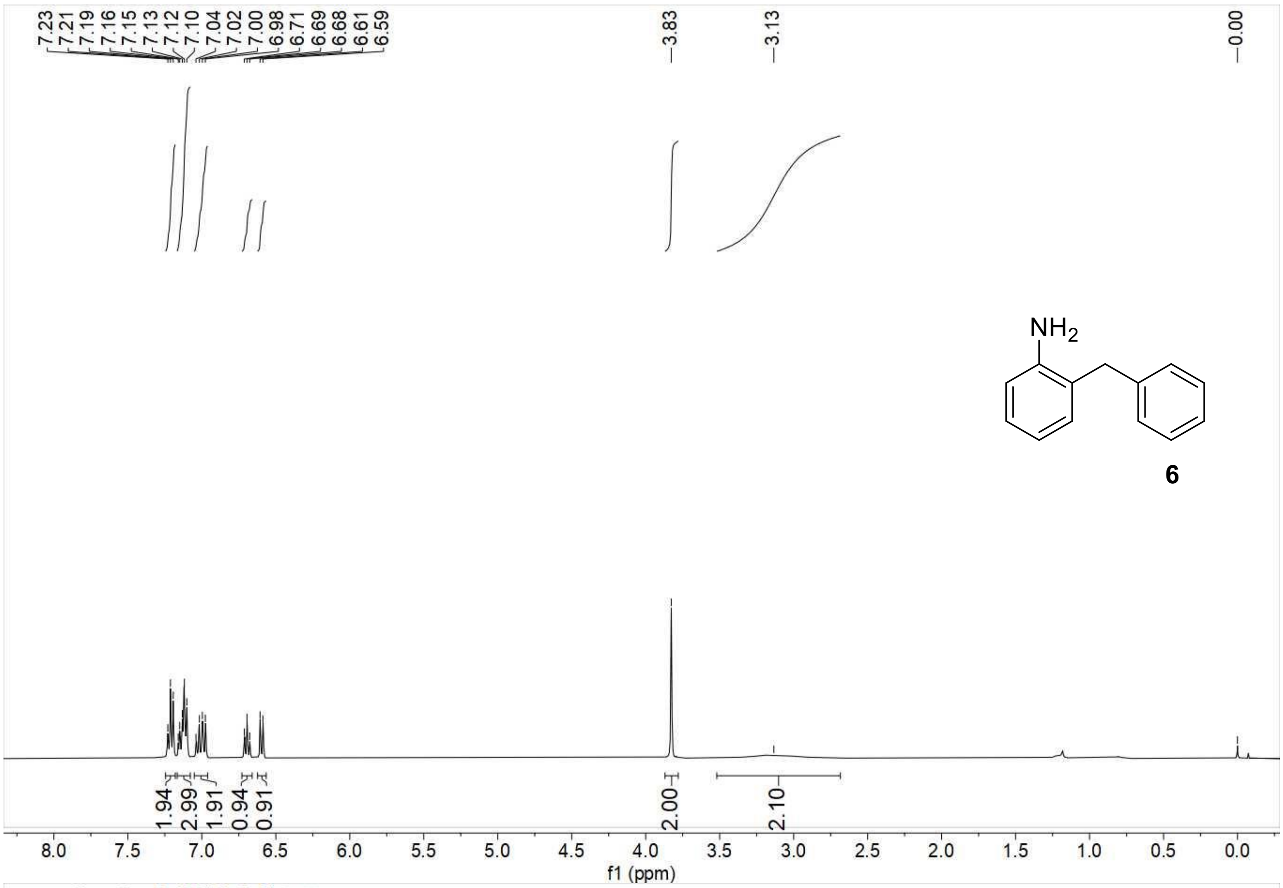
7.23
7.21
7.19
7.16
7.15
7.13
7.12
7.10
7.04
7.02
7.00
6.98
6.71
6.69
6.68
6.61
6.59
3.83
3.13
0.00
NH2
6
1.94
2.99
1.91
0.94
0.91
2.00
2.10
f1 (ppm)

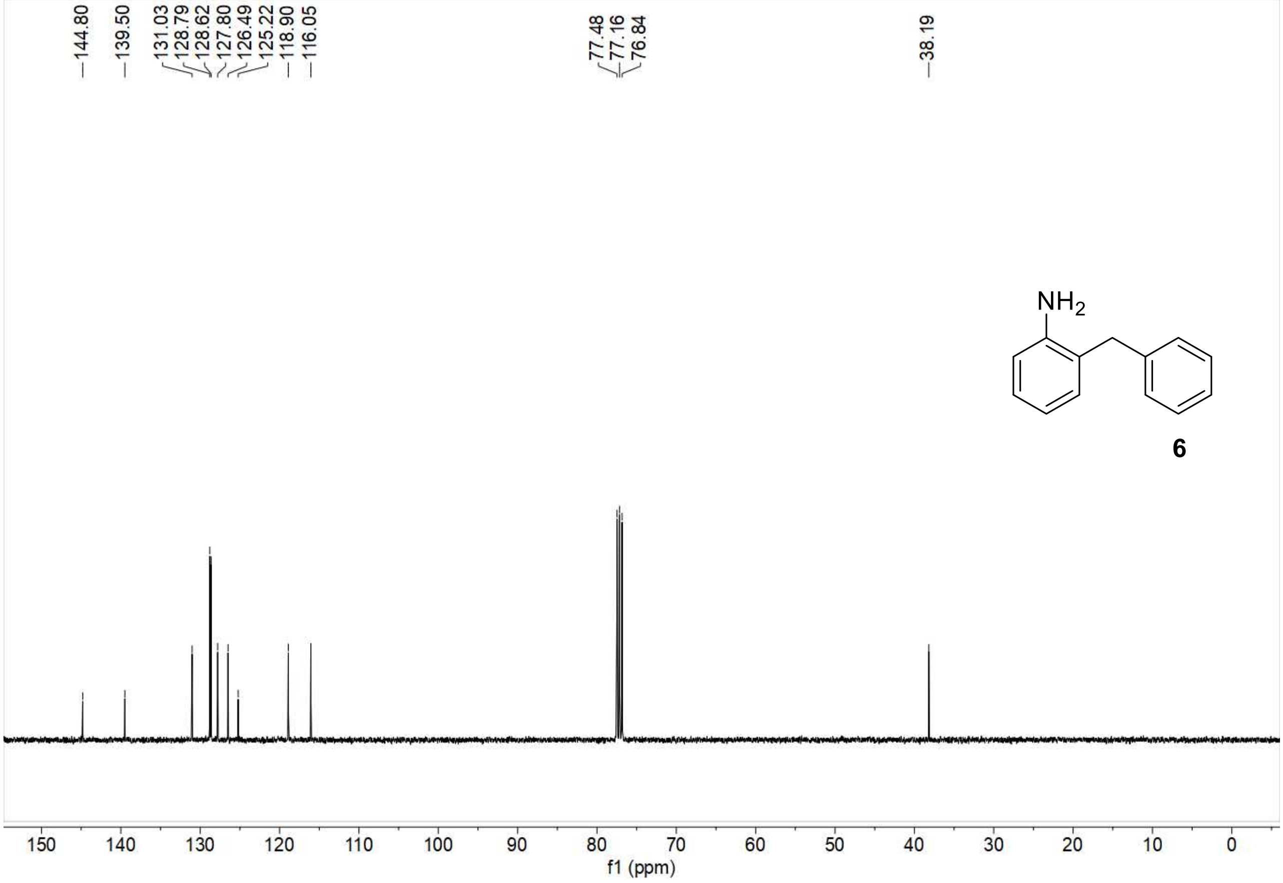
144.80
139.50
131.03
128.79
128.62
127.80
126.49
125.22
118.90
116.05
77.48
77.16
76.84
38.19
NH2
6
f1 (ppm)

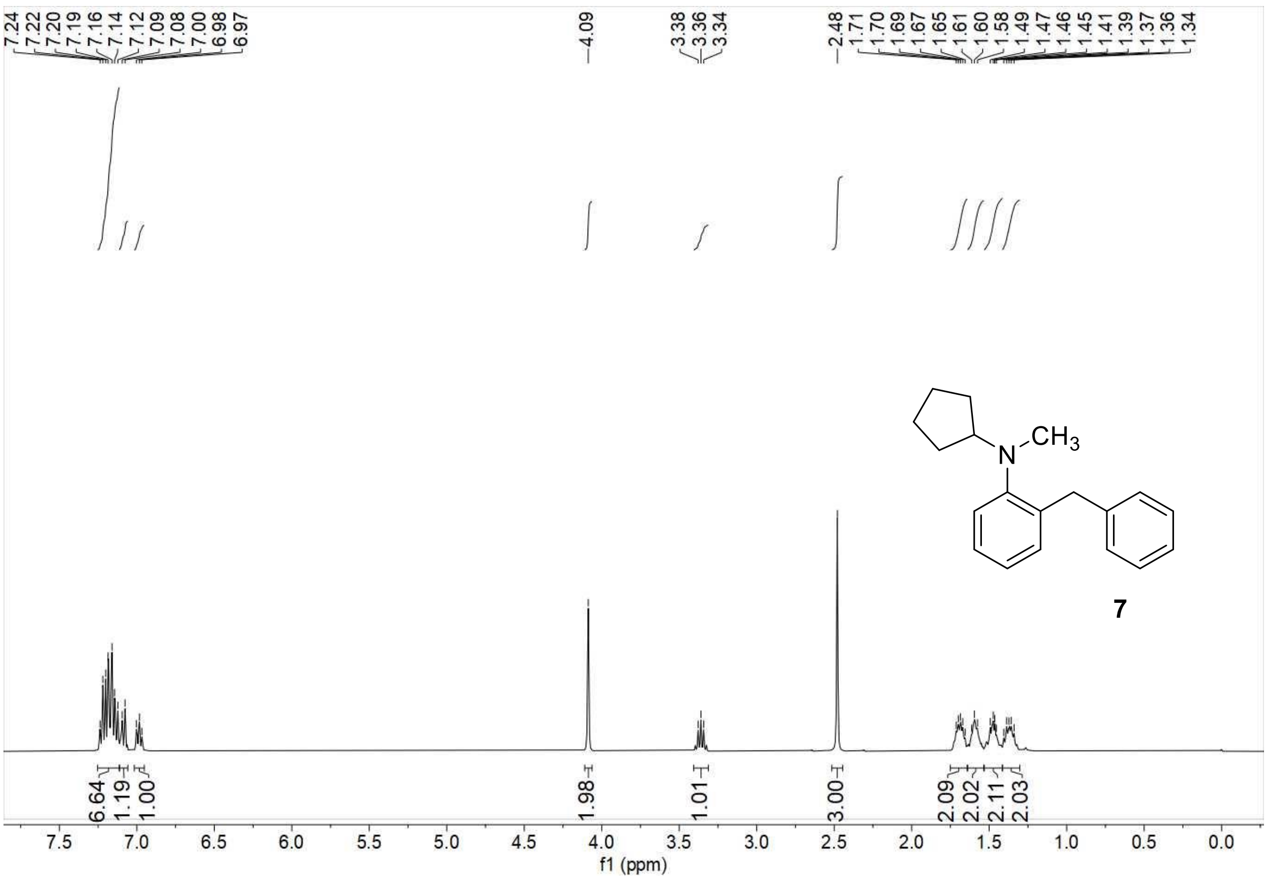
7.24 7.22 7.20 7.19 7.16 7.14 7.12 7.09 7.08 7.00 6.98 6.97
4.09
3.38 3.36 3.34
2.48 1.71 1.70 1.69 1.67 1.65 1.61 1.60 1.58 1.49 1.47 1.46 1.45 1.41 1.39 1.37 1.36 1.34
CH_3
N
7
6.64 1.19 1.00 1.98 1.01 3.00 2.09 2.02 2.11 2.03
7.5 7.0 6.5 6.0 5.5 5.0 4.5 4.0 3.5 3.0 2.5 2.0 1.5 1.0 0.5 0.0
f1 (ppm)

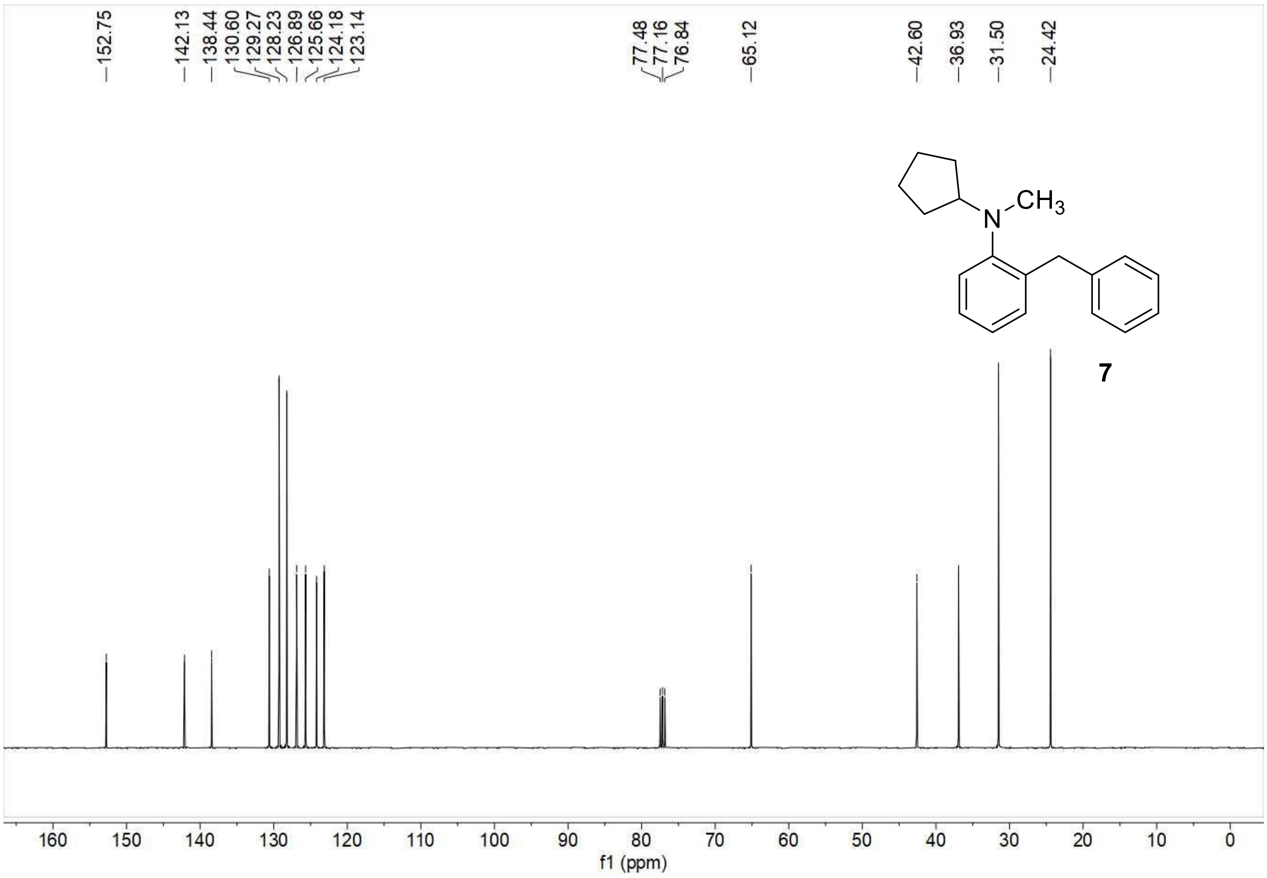
152.75
142.13 138.44 130.60 129.27 128.23 126.89 125.66 124.18 123.14
77.48 77.16 76.84
65.12
42.60 36.93 31.50 24.42
CH_3
N
7
160 150 140 130 120 110 100 90 80 70 60 50 40 30 20 10 0
f1 (ppm)
